# Supplementary material for: Global scenarios of resource and emission savings from material efficiency in residential buildings and cars
Source: Nat Commun. 2021 Aug 24;12:5097. doi: 10.1038/s41467-021-25300-4 (PMC8385048; doi:10.1038/s41467-021-25300-4)
Supplement: Supplementary file 1 — Supplementary Information [file 41467_2021_25300_MOESM1_ESM.pdf]

July 2021

Global Scenarios of Resource and Emission Savings from Material Efficiency in Residential Buildings and Passenger Vehicles

Supplementary material

Stefan Pauliuk, Niko Heeren, Peter Berrill, Tomer Fishman, Andrea Nistad, Qingshi Tu, Paul Wolfram, and Edgar G Hertwich

Content

(1) Relation of this research to already published works and advancement beyond the state of the art
(2) Additional material: introduction and method
(3) Scenarios and model framework (short description)
(4) RECC 2.4 database and scenario drivers
(5) Additional Results and Discussion

1. Relation of this research to already published works and advancement beyond the state of the art

1.1. Advancement over previous work and relation to own preparatory work

The advancement presented here was made possible through a large effort on data collection, scenario development, model development and result analysis.
Compared to previous work of colleagues (see sub-section below), the following major advances apply to the ODYM-RECC model framework. Detailed research and modelling gaps in previous work are reported in detail further down:

- **Consistency:** We do not consider industry as an end-use sector and calculate industry output (material production, construction, vehicle manufacturing, and waste management) so that it is consistent with what happens in the use phase of the end-use sectors industry and buildings. By doing so, we quantify the different steps of the stock-flow-service nexus [1], and parts of the Energy Service Cascade [2] step-by step, where once step is consistent with the next one and all calculations are physically consistent (i.e., mass- and energy-balanced and described by explicit parameter relations).
- **Material cycle and product life cycle perspective:** With the consistent bio-physical modelling of energy and material flows in the system, we can depict both: the technical material cycles supporting service provision (and the recycling potential and resource and energy demand in them) and the product life cycles with their supply chains, use phase, and end-of-life management.
- **Strategy detail:** We explicitly model, with dedicated energy and material parameters, ten different material efficiency strategies, covering product design, manufacturing, use phase, consumer demand, and the waste management industries.
- **Emphasis on demand-side solutions**: Edelenbosch et al. [3] recently published highlighting the lack of attention towards demand-side solutions in integrated assessment models (IAMs), noting that there is great uncertainty in future energy/cap, and pointing out the potential for energy efficiency in transport and buildings in particular.

Not only through our assessment of ME efficiency strategies here, which as noted is absent from the IAM framework, we also contribute important knowledge with respect to future drivers of service and energy demand per capita, responding to Edelenbosch et al.'s [3] "challenge [for modelers] to better understand drivers of future energy efficiency and service demand, that contribute to the projected energy demand".

The data gathering, scenario development, model development, and analysis work we build upon here is very comprehensive and involves a number of disciplinary advances that deserve separate publication. Moreover, the modular nature of our research approach means that different elements, like the scenarios and the model framework, can be used in different settings and will then need specific references to publications with high level of detail. From

our own experience and exchange will colleagues, we know that it is often frustrating to try to extract data, verify calculations, or even re-use model code from work that is presented in monolithic publications without much technical detail, and here, we want to facilitate cumulative research by documenting and discussing the different aspects of the work in dedicated publications. We directly build upon the following works:

Preparatory work:

- Detailed description of how the socioeconomic scenarios are constructed and rationales for the different parameter choices [4]. These scenarios are independent of the RECC modelling framework and part of the family of literature that details the SSP storylines to make them directly different for different end-use and intermediate sectors and services.
- Detailed description of the engineering approach taken for the energy-material-consistent vehicle archetype modelling for the six drive technologies, four car segments, and four vehicle material design choices [5]. This product-level work is independent of the RECC modelling framework and adds to the process modelling literature (focus here on passenger vehicles as end-use energy conversion process and their consistent physical description regarding use phase energy use and material composition).
- A detailed description of the engineering approach taken for the energy-material-consistent building archetype modelling for the three building types, four energy standards, and four building material design choices is in preparation. This modelling is comprehensive in that it consistently reflects the different thermal properties of material design choices in the building energy balance model and takes different designs (e.g., window size and orientation) and different regional climates into account.
- A detailed description of the underlying material flow analysis (MFA) software for multi-dimensional system descriptions and the data model that allows for efficient data exchange between the project's work packages [6]. This software was developed with the application in the RECC project in mind but is independent of it and contributes to the literature on open source software for environmental systems analysis and industrial ecology, in particular.

Complementary work:

- An introduction to the ODYM-RECC model framework [7] to explain in detail how the modules from the different scientific fields are integrated to construct the consistent multi-strategy scenarios for the in-use stocks and related material cycles and supply chains for the different services. This publication describes the model details that apply to all global and case studies made with the framework. They explain, for example, how the archetype inventories are scale up, how the in-use stock and its turnover is calculated, or how energy demand and environmental pressure indicators are calculated.
- The work for the International Resource Panel [8] mainly covers the G7 countries in great detail, plus China and India as reference cases. It does not have a global scope and captures the service demand of ca. half of the world's population only. Moreover, it focusses exclusively on GHG metrics and not on the equally relevant material and energy indicators that also can be extracted from the framework.

Advancement of the current approach:
The research reported here expands our knowledge on the climate implications of material efficiency along several dimensions. Compared to own previous work, two major advances apply:

- **Global coverage:** Unlike the work for the International Resource Panel [8], which covered the G7 countries plus China and India and thus ca. half of the world's population only, this work was expanded considerably in scope and now covers the entire world with a resolution of 20 countries/regions. Next to putting more emphasis on the developing regions of the planet, where the potential for material efficiency is higher than for the high-income regions with mature in-use stocks, it allows for estimating the potential climate benefits of a global scale-up of material efficiency in the two end-use sectors. Thus, we can now quantify the contribution of a wide bundle of ME strategies to reducing the global carbon budget overshoot by residual emissions in a low carbon energy future for these two sectors.

- **Expanding the scope of research questions:** Next to quantifying the strategy-specific energy and climate implications of the different ME strategies, individually and as a bundle and by region and global, this work puts more focus on (i) the material cycle implications, by reporting future primary and secondary production volumes, (ii) contraction and convergence of per capita stocks across regions, and (iii) the context, by juxtaposing the ME-induced emissions reduction with those obtained from efficiency and low carbon energy supply for both Global North and Global South.

1.2. Comparison to other studies on the system-wide potential and implications of material efficiency

Comparison of the low energy demand (LED) study material efficiency scope compared to the ME scope of this study.

The LED work [9] has energy as its focus and therefore includes energy savings mediated by material efficiency, and demand-side measures, in particular.

The table below shows the differences in ME strategy coverage between the two studies (Supplementary Table 1). The information was taken from the extensive available documentation on the LED scenarios and the data and assumptions that went into them.

Supplementary Table 1: The ten material efficiency strategies considered in ODYM-RECC V2.4 and their coverage by the LED study [9] as far as documented and supplied with underlying data and assumptions for the two sectors covered by our study: passenger vehicles and residential buildings. The references in the table below point to the supplementary material of Grubler et al. (2018).

**Strategy**	**Buildings (residential and non-residential)**	**Vehicles**
Using less material by light-weighting through improved design and/or downsizing, **ULD**	**Included but not explicit**, described implicitly, via a 'dematerialisation multiplier'. No consistent material life cycle perspective in the sense that material demand/material cycle flows are consistently calculated as a result of the in-use stock turnover for the different demand-side measures.	**Included but not explicit**, described implicitly, via a 'dematerialisation multiplier'. No consistent material life cycle perspective in the sense that material demand/material cycle flows are consistently calculated as a result of the in-use stock turnover for the different demand-side measures.
Less material through light-weighting by material substitution, **MSu**	**Impact on material demand is described implicitly**, via a "material efficiency" term, which includes (mostly manufacturing side) strategies such as longer product lifetimes, altered product designs, or remanufacturing/reuse of existing components. This aggregate factor is quantified based on previous work, justified by expert judgement, and cross-checked with back-of-the-envelope calculations against the product demand in the end-use sectors like vehicles and buildings.	
Fabrication yield improvement, **FYI**	**Impact on material demand is described implicitly**, via a "material efficiency" term, which includes (mostly manufacturing side) strategies such as longer product lifetimes, altered product designs, or remanufacturing/reuse of existing components. This aggregate factor is quantified based on previous work, justified by expert judgement, and cross-checked with back-of-the-envelope calculations against the product demand in the end-use sectors like vehicles and buildings.	
End-of-life recovery rate improvement, **EoL**	**Impact on material production energy consumption is described implicitly**, via changes in specific energy needs per unit of material produced (GJ/ton).	
Fabrication scrap diversion, **FSD**	**Impact on material demand is described implicitly**, via a "material efficiency" term, which includes (mostly manufacturing side) strategies such as longer product lifetimes, altered product designs, or remanufacturing/reuse of existing components. This aggregate factor is quantified based on previous work, justified by expert judgement, and cross-checked with back-of-the-envelope calculations against the product demand in the end-use sectors like vehicles and buildings.	
Car-sharing, **CaS**	**Not explicit:** "Shared modes, higher load factors, constraints on car ownership and parking in dense urban areas, combine to yield significant reductions in the size of the global vehicle stock." Implemented implicitly via utilization factor passenger-km/vehicle (Suppl. Table 16).<br><br>Impact on material demand is described implicitly, via a 'dematerialisation multiplier' quantified by expert judgement and cross-checked with back-of-the-envelope calculations against the product demand in the end-use sectors like vehicles and buildings.	
Ride-sharing, **RiS**	**Not explicit:** "Shared modes, higher load factors, constraints on car ownership and parking in dense urban areas, combine to yield significant reductions in the size of the	

	global vehicle stock." Implemented implicitly via utilization factor passenger-km/vehicle (Suppl. Table 16)<br><br>Impact on material demand is described implicitly, via a 'dematerialisation multiplier' quantified by expert judgement and cross-checked with back-of-the-envelope calculations against the product demand in the end-use sectors like vehicles and buildings.
More intensive use, **MIU**	**For residential buildings: via per capita floor space reduction** (Suppl. Table 3)<br><br>**For passenger vehicles: via utilization factor passenger-km/vehicle** (Suppl. Table 16)<br><br>Impact on material demand is described implicitly, via a 'dematerialisation multiplier' quantified by expert judgement and cross-checked with back-of-the-envelope calculations against the product demand in the end-use sectors like vehicles and buildings.
Product lifetime extension, **LTE**	**Impact on material demand is described implicitly,** via a "material efficiency" term, which includes (mostly manufacturing side) strategies such as longer product lifetimes, altered product designs, or remanufacturing/reuse of existing components. This aggregate factor is quantified based on previous work, justified by expert judgement, and cross-checked with back-of-the-envelope calculations against the product demand in the end-use sectors like vehicles and buildings.
Recovery, remanufacturing, and reuse of components, **ReU**	**Impact on material demand is described implicitly,** via a "material efficiency" term, which includes (mostly manufacturing side) strategies such as longer product lifetimes, altered product designs, or remanufacturing/reuse of existing components. This aggregate factor is quantified based on previous work, justified by expert judgement, and cross-checked with back-of-the-envelope calculations against the product demand in the end-use sectors like vehicles and buildings.

Summary: Grubler et al. (2018) include many material efficiency strategies, but only one is represented by changing a strategy-specific parameter: the more intense use of buildings (via lower m^2/capita) and vehicles (via higher passenger-km per vehicle). All other strategies are included implicitly by a change in an aggregate 'dematerialisation multiplier' and a 'material efficiency' term, both representing factors to scale down total material production demand from its baseline. Changes in the waste management and recycling industries are included as one reason behind the assumed changes in specific energy needs per unit of material produced (GJ/ton).
This approximate representation of material efficiency gives a roughly consistent estimation of future energy demand for material production for the different end-use sectors, which is suitable for the purpose of the LED study, which is the compilation of bottom-up scenarios for energy demand estimates across sectors. Here, it is sufficient to represent the material cycle industries in a broad-brush fashion, as they are one of many sectors considered and the feedback of lower end-use service demand on material production is included with some rough estimates.

For a detailed assessment of the material efficiency-climate change linkage, however, this approach is not suitable, as our detailed reasoning for the three dimensions with substantial advancement beyond the state of the art shows:

Consistency: The LED approach to the service-material linkage does, however, not solve the problem that industry output, and with it material production, is modelled as end-use sector, independently from the demand in the intermediate (like freight transport and commercial buildings) and final demand sectors (like passenger vehicles or residential buildings). Changes in the material cycle industries are not consistent with the changes in the end use sectors, because the parameters that can establish such consistency (product lifetime and material composition, scrap recovery efficiencies, re-use shares, …) are not present and dealt with implicitly by changes in more aggregate parameters.

Material cycle and product life cycle perspective: For the same reason as above (lack of detailed parameters in the material part of the LED study), a detailed mass-balanced depiction of the in-use stocks and material cycles is not possible in LED.

Strategy detail: For the same reason as above (lack of detailed parameters in the material part of the LED study), a detailed depiction of most individual ME strategies in the material cycle is not possible in LED.

Comparison of our work to the Material Economics 'Circular Economy' material efficiency scope compared to the ME scope of this study.

In contrast to Grübler et al, the gray literature Circular Economy report published by Material Economics (https://materialeconomics.com/publications/the-circular-economy) has materials as its focus, and does not quantify changes to energy demand and energy related emissions. Nevertheless, with its particular focus on material efficiency/circular economy strategies applied to buildings and passenger vehicles, it shares some common scope and approach with this study. The table below shows the differences in ME strategy coverage between the two studies (Supplementary Table 2). The information was taken from the description of approaches detailed in the Circular Economy report.

Supplementary Table 2: The ten material efficiency strategies considered in ODYM-RECC V2.4 and their coverage by the Circular Economy report [https://materialeconomics.com/publications/the-circular-economy] as far as documented and supplied with underlying data and assumptions for the two sectors covered by our study: passenger vehicles and residential buildings.

**Strategy**	**Buildings (residential and non-residential)**	**Vehicles**
Using less material by light-weighting through improved design and/or downsizing, **ULD**	**Included**, based on explicit assumption that material intensity of construction reduces from 2.45 to 1.92 t/m2, to which reduction of over-specification of construction materials contributes a reduction of 0.22 t/m2. Other contributions to material intensity reductions are described below. Scope-wise, they consider non-residential building in addition to residential buildings	**Included**, based on explicit assumption that fleet-average material content per vehicle reduces from 1.3t to 0.9t. Scope-wise, they consider only passenger cars, not SUVs and light trucks, which are included in ODYM-RECC
**MSu**	**Excluded**	

Fabrication yield improvement, **FYI**	**Excluded**. The supply of scrap is discussed at length, but improved FYI is not modelled. A related strategy is considered in the buildings sector, i.e. waste reduction on construction sites. This is assumed to reduce from 15% to 5%. This strategy (Reducing On-site Construction Waste) is not considered in ODYM-RECC	
End-of-life recovery rate improvement, **EoL**	**Excluded**. EoL is not considered in its own right, but increased re-use is modeled, see strategy ReU	
Fabrication scrap diversion, **FSD**	**Excluded**. The supply of scrap is discussed at length, but improved FSD is not modelled.	
Car-sharing, **CaS**	**Included**. Both car-sharing and ride-sharing are represented in an aggregate way by assuming that average occupancy per car increases from 1.63 to 1.91<br><br>This contrasts to our approach where model car-sharing as independent from ride-sharing. Car-sharing does not change vehicle occupancy but the annual vehicle kilometrage, leading to a more intense use of vehicles, thus a smaller car fleet but also a resulting shorter lifetime and faster introduction of new and/or more efficient drive technologies.	
Ride-sharing, **RiS**	**Included**. Both car-sharing and ride-sharing are represented in an aggregate way by assuming that average occupancy per car increases from 1.63 to 1.91<br><br>This contrasts to our approach where model car-sharing as independent from ride-sharing. In our model ride-sharing is modeled as the share of total passenger-km delivered by the vehicle fleet, for which the occupancy rate changes by +1 compared to the base value (one additional passenger on average). The share of passenger-km provided by ride sharing is modelled with an implementation curve, which scales the +1 passenger, with for example 0% in 2016 to 100% in 2060 in the LED scenario.	
More intensive use, **MIU**	**Included.** Floor area requirements among all building types (excl. Industrial) are assumed to reduce by 5%, from 52 m2/cap to 49 m2/cap. The inclusion of some non-residential buildings is a feature of the Circular Economy study which is not included in ODYM-RECC v2.4. Our study contains more detail on variation in m2/cap per country/region, and in trajectories of m2/cap by scenario.	**Included,** by assuming that average occupancy per car increases from 1.63 to 1.91. There is also more intense use of each vehicle, see LTE
Product lifetime extension, **LTE**	**Included.** Average lifetime of buildings is assumed to increase by 40% (from 65 -> 91 yrs)	**Included.** Average lifetime (i.e. lifetime km driven) per vehicle increases from 280,000 km to 450,000 km.
Recovery, remanufacturing, and reuse of components, **ReU**	**Included.** 15% of structural building components are assumed to be reused. It is not explicitly stated, but based on discussion elsewhere, this is likely and increased from 5% for steel components, and from 0 for other materials.	**Included.** The share of new materials per vehicle is assumed to fall from 95% to 89% (recycled content approac)

Summary: Material Economics include several material efficiency strategies in their Circular Economy report. The most prominent difference between that report and this study is that they only consider material related emissions, while we consider both material and use-phase (energy consumption) related emissions. On the treatment of the material efficiency strategies, we consider more strategies, and even for most of the strategies considered by Material Economics, we have greater detail, for instance greater variation of MIU in buildings by region and scenario.

To summarize, more at a meta-level, compared to the Material Economics study, our work is

- open, transparent, and reproducible
- corresponds to and interfaces with well-known scenarios (SSP and LED)
- has multiple baseline scenarios
- has high regional differentiation with equal focus on all regions including developing ones

Whereas the Material Economics study

- has only one baseline scenario
- covers varying regions per material or end use: 11 world regions for steel, 9 for plastics, five for aluminum..., yet present most results only for the EU and the world
- includes estimates of economic costs and co-benefits

Detailed summary of Material Economics Circular Economy report

Product materials efficiency: Refers to reducing the "total materials input to key products", by

- reducing the amount of materials lost in production (c.f. RECC strategies Fabrication yield improvement & Fabrication scrap diversion)
- "use more advanced materials and construction techniques, such as high-strength steel that can cut materials use by 30%", and "reduce over-specification, such as the near 100% overuse of steel in buildings relative to what is strictly required to meet design specifications" (c.f. RECC strategy Light-weighting)
- New circular business models in mobility and buildings: Refers to increasing utilization of non-residential buildings, and vehicles. "Sharing enables much more intensive use". For vehicles, this means an increase of vehicle lifetime kilometers, higher utilization (through managed car/ride-sharing and automation), use of lower carbon energy (through increased electrification) and less material use (through smaller vehicle stocks due to higher lifetime km per vehicle?). "In a circular scenario, the materials input to mobility falls by 75%."- Don't know yet if this is an 'expert assumption' or a modelling outcome.

Mobility Chapter 5: Focus on passenger cars. "In a scenario where professionally managed, shared vehicle fleets account for two-thirds of travel, materials requirements could fall by as much as 75%, reducing annual CO_2 emissions from materials production by 43 Mt by 2050." "Higher utilization justifies much more investment in upfront costs, from the higher cost of

electric-vehicle drivetrains, to more advanced automation technology, or higher-performance materials." "The average car would be smaller, requiring far less material, and be far more durable and better maintained."

Reuse & Remanufacturing: Share of new materials per vehicle falls from 95% to 89%

Light-weighting: Average mass per car reduced from 1.3t to 0.9t

Longer lifetime: Average lifetime (i.e. lifetime km driven) per vehicle increases from 280,000 km to 450,000 km.

Sharing: Average occupancy increases from 1.63 to 1.91 passengers per car.

Buildings Chapter 6: Cement Recycling: Basically an assumption on reduction of the carbon intensity of cement production, attributed to an increase in cement recycling. "Cement Recycling becomes widespread, cutting CO2-intensity from 0.62 to 0.48 t CO2/t cement". It seems like they are assuming an increase in the share of secondary cement in cement production. We have something similar, judging by the cement box of Figure 3, by including the re-use of modular concrete building components. RECC cannot currently quantify the effect of increased secondary cement production on the GHG intensity of overall cement production.

Waste Reduction: "waste during construction is reduced to 5%" (unclear from what baseline, but probably ~15% based on earlier text on page 150)

Re-use of building materials: 15% of structural building components are reused. It is not explicitly stated, but based on discussion elsewhere, this is likely and increased from 5% for steel components, and from 0 for other materials.

Materials Efficiency: Reduction in material intensity of construction, from 2.45 t/m2 to 1.92 t/m2. Steel and cement use falls by 20-30% as a result of reduced over-specification and use of higher-quality materials.

Sharing (a.k.a. circular business models): Sharing reduces floor area requirements by 5% (from 52 -> 49 m2/cap) across all building categories, except industrial (so residential and commercial). Average lifetime of buildings increases by 40% (65 -> 91 yrs)

Comparison of our work to the IEA's efforts on capturing material efficiency in their energy future models, compared to the ME scope of this study.

Material efficiency strategies have been systematically added to the International Energy Agency's (IEA) energy supply and demand modelling suite. The 2015 World Energy Outlook by the IEA contains a material efficiency scenario [10], with the following central findings. First, on P26: "Changing product design, re-use and recycling ("material efficiency") also offers huge potential for energy saving; for energy-intensive products such as steel, cement, plastics or aluminium, efficient use and re-use of materials can save more than twice as much energy as can be saved by efficiency measures in the production process to 2040." And on P387: "Achieving greater efficiency in the use of materials through light-weighting, longer life products, re-use and recycling, is an important complementary strategy to energy efficiency in energy-intensive industries, as the potential for energy savings is about twice as large."

In a later report "Material efficiency in clean energy transitions" [https://www.iea.org/reports/material-efficiency-in-clean-energy-transitions], the IEA summarizes that "Clean energy transitions require decoupling of economic growth from material demand." The modelling approach focusses on steel, cement, and aluminium in the buildings/construction and vehicle value chains.
The table below shows the differences in ME strategy coverage between our and the IEA's latest study on material efficiency (Supplementary Table 3). The information was taken from the main report "Material efficiency in clean energy transitions".

Supplementary Table 3: The ten material efficiency strategies considered in ODYM-RECC V2.4 and their coverage by the IEA report "Material efficiency in clean energy transitions" [https://www.iea.org/reports/material-efficiency-in-clean-energy-transitions] as far as documented and supplied with underlying data and assumptions for the two sectors covered by our study: passenger vehicles and residential buildings.

**Strategy**	**Buildings (residential and non-residential)**	**Vehicles**
Using less material by light-weighting through improved design and/or downsizing, **ULD**	**Included**, as light-weighting of vehicles and improved building design and construction: Switch to composite frames, optimize steel and other frames. Values: Tables 8 and 9.	**Included**, as light-weighting of vehicles, combined with material substitution (Table 10).
Less material through light-weighting by material substitution, **MSu**	**Included**, as light-weighting of vehicles, combined with material substitution (Table 10), and improved building design and construction. Building: Use of high-strength steel and lower cement content modelled, see Tables 8 and 9.	
Fabrication yield improvement, **FYI**	**Included**. The strategy is split into one for semi-manufacturing yield improvement (home scrap reduction) and one for product manufacturing yield improvements (fabrication or new scrap reduction). Yield increases shown in Tables 4 and 6.	
End-of-life recovery rate improvement, **EoL**	**Included**. The material efficiency scenarios applies a 98% collection rate, relative to 85% currently. (Table 8).	
Fabrication scrap	**Excluded**.	

diversion, **FSD**		
Car-sharing, **CaS**	**Included**. Both car-sharing and ride-sharing are represented in an aggregate way by modeling a 'changes in activity' factor.	
Ride-sharing, **RiS**	**Included**. Both car-sharing and ride-sharing are represented in an aggregate way by modeling a 'changes in activity' factor.	
More intensive use, **MIU**	**Not included.**	**Included**. Both car-sharing and ride-sharing are represented in an aggregate way by modeling a 'changes in activity' factor.
Product lifetime extension, **LTE**	**Included.** Annual retrofit rate of 2-3% of the buildings stock and extension of new commercial buildings lifetime to 50-70 years.	**Implicitly included.** Re-use rates for vehicle materials are quite high, can be partly attributed to re-use/lifetime extension.
Recovery, remanufacturing, and reuse of components, **ReU**	**Included.** Values provided in Tables 5, 7 and 9, re-use rates rise until 50% for ship steel in the material efficiency scenarios, up to 13% for buildings, 8% for infrastructure. 10% re-use of precast concrete elements.	**Included.** Values provided in Tables 5 and 7, re-use rates rise until 50% for ship steel in the material efficiency scenarios, up to 13% for buildings, 8% for infrastructure.

Summary: The IEA material has a scope similar to the one of our work and is partly built on the same data. It is more detailed on the manufacturing side (including home scrap reduction) but its use phase material efficiency is not (at least not documented) modelled on material-energy consistent building and vehicle use phase modelling.

1.3. Comparison of our work to other reports on resource efficiency and partly the climate linkage at the science-policy level

In their report **"Quantifying the benefits of circular economy actions on the decarbonisation of EU economy"** prepared for the European Environment Agency in 2018, Trinomics consultants and colleagues [http://trinomics.eu/wp-content/uploads/2020/04/Trinomics-2018-Quantifying-the-benefits-of-circular-economy-actions-on-the-decarbonisation-of-EU-economy_final-report.pdf] perform a review. "The review focused on the non-energy elements of circular action and showed that such actions can make modest, yet valuable impacts on GHG abatement throughout sectors and throughout the different lifecycle stages of products in Europe." They develop options and recommendations for the development of a methodological framework for calculating GHG impacts of Circular economy activities, which include:

- Methodology must be comprehensive, detailed, robust, easily aggregated, synergistic and building on a limited set of existing data. (some of these characteristics may be in conflict with each other)
- A combination of a detailed analysis (such as LCA) with macro-economic modelling offers the most promise regarding the method requirements, but further work is needed to facilitate the exchange of data between the techniques.

A recent OECD report performs a similar review and comes to the conclusion that there is a lack of global forward-looking material cycle models available for detailed CE assessments. More detail and better connection between technology-detail ('bottom-up') and aggregated macroeconomic ('top-down') representations is needed [11].

In the report "**THE DECARBONISATION BENEFITS OF SECTORAL CIRCULAR ECONOMY ACTIONS**", prepared for the European Environment Agency in 2020, Rambøll, Fraunhofer ISI, and ecologic institute consultants and colleagues [https://de.ramboll.com/-/media/files/rm/rapporter/methodology-and-analysis-of-decarbonization-benefits-of-sectoral-circular-economy-actions-17032020-f.pdf?la=de] provide "an approach to how to select and assess circular economy actions (in the following, "CE actions") and their impact on the mitigation of climate change". "It integrates results from lifecycle analysis (LCA) and material flow modelling to develop a generic methodology for conducting ex ante quantifications of the CO_2 emission reduction potential of CE actions (Part 1 of the report)" and was "designed to be applicable to a broad range of economic sectors."

Starting with a scoping to identify the most relevant CE action for a given sector, the authors propose to apply LCA to estimate the savings potential of individual relevant strategies and also estimate the rebound effect and then an MFA to estimate the effects on material cycles, followed by a scale-up to quantify the overall potential. It is proposed that 'circular economy rebound' effect are factored into the potential savings estimate by reducing the original LCA-based potential by a certain percentage. In this pilot study, no rebound effects are taken into account, mostly because of the lack of their assessment.

The report provides a first example of their methodology, focusing on cement with the following RE/CE strategies:

- Reduce cement content of buildings by cutting over-specification in volume and in strength class.
- Use other types of cement and by-products from other industries
- Use pre-cast concrete elements
- Use timber instead of cement
- Optimize use of building space
- Renovate rather than demolish
- Reuse concrete elements
- Recycle cement from waste concrete
- Design buildings for disassembly

For steel, reducing design over-specification and the reuse of structural steel are included.

Global-scale resource use outlooks and economy-wide modelling of material use in general
Global system wide repercussions of resource efficiency have been captured by a number of general equilibrium approaches. A list and review of recent approaches is given in Winning et al. [12]. It includes the work with computable general equilibrium models (CGE) of Böhringer and Rutherford [13,14], the EllenMcArthur Foundation and McKinsey, EXIOMOD, and GINFORS as approaches to assess resource efficiency in a GCE framework, as well as the econometric model E3ME and the mixed model framework GIAM/GTEM-C. Recent major additions to the literature are the Global Resource Outlooks by the OEDC and the UN IRP, both published in 2019 and built upon a CGE framework [15,16]. For such works, CGE-based macro-economic models, such as GTEM-C are combined with physical accounts or physical sectoral models [17,18], including MEFISTO stock and flow framework [19]. The CGE model frameworks consider materials only in a stylistic manner, by adding external factors of 'satellite' accounts of raw material use by economic sector output. For sectors where no physical 'foreground model' is present, these factors are then assumed to change by a certain percentage per year, based either on historic trends (business-as-usual) or decline more sharply (resource-efficient scenario), but the underlying physical relationships of that change or material use in general are not explored in detail or cross-check against physical models of these sectors.

A recent model review by the OECD (McCarthy et al., 2018) found that the material cycle processes relevant for quantifying the economy-wide impacts of material efficiency in a detailed manner are not described by these models, hence, such assessments can only give a rough estimate of future material use. They can neither be checked for physical correctness (do the service-providing products actually need that many materials for their production?), nor can the savings potential of the many different material efficiency strategies and policy options be assessed. Clearly, improvements are needed.
Physical detail needs to be added to macro-economic models. Winning et al. [12] and Schuhmacher and Sands [20] amend CGE models by adding detail about steelmaking, e.g., by disaggregating the steel sector into the primary and secondary production route. Cooper et al. [21] use an MRIO approach to study the linkage between circular economy strategies, energy use, and emissions. They do not capture material flows and cycles themselves, as these are not covered by monetary IO models.

2. Additional material: introduction and method

Introduction: the share of passenger vehicles and residential buildings in total final energy consumption was determined as follows:

For passenger vehicles: Directly from IEA statistics: https://www.iea.org/data-and-statistics?country=WORLD&fuel=Energy%20consumption&indicator=Share%20of%20total%20final%20consumption%20(TFC)%20by%20sector queried on September 15, 2020.

> "Explore energy data by category, indicator, country or region": Energy topic: Energy consumption. Indicator: Share of total final consumption (TFC) by sector. Region: world. Sector: Transport: Time: 2018, Value: **29%**
>
> Share of passenger vehicles' energy consumption in total Energy consumption by transport sector, 2012: **61%.**

https://www.eia.gov/outlooks/ieo/pdf/transportation.pdf U.S. Energy Information Administration | International Energy Outlook 2016, Chapter 8: Transportation sector energy consumption, p 126. Multiplying the tow value gives 17,69%, which is reported as 18% in the paper.

For residential buildings: Directly from IEA statistics: https://www.iea.org/data-and-statistics?country=WORLD&fuel=Energy%20consumption&indicator=Share%20of%20total%20final%20consumption%20(TFC)%20by%20sector queried on September 15, 2020.

> "Explore energy data by category, indicator, country or region": Energy topic: Energy consumption. Indicator: Share of total final consumption (TFC) by sector. Region: world. Sector: Residential: Time: 2018, Value: **21%**

Method: Gross domestic product (GDP) was deliberately not chosen as scenario driver nor model input to enable a high-resolution service-level framework that can be used to depict future low carbon lifestyles instead of aggregate demand modelling (e.g. modeling useful energy demand as aggregated function of GDP), for which there is little evidence for decoupling [22].

Only in rare cases where there is a long enough time series, GDP-based extrapolations for the building stock were used for determining future service level in the SSP2 scenario. This approach was used for the USA and Japan.

3. Scenarios and model framework (short descrption)

Services are linked to material cycles via the stock-flow-service nexus [1] (Supplementary Figure 1). The scheme starts with the energy service cascade to relate values to services to functions to products (and their operation) [2], stock-driven modelling to translate product in-use stock demand into production of new and recycling of old products [23], new and old products to material flows via dynamic material flow analysis (MFA) [24], and the material flows to the energy demand and related GHG emissions via environmental extensions as done in previous work [25,26].

Supplementary Figure 1 (next page): Calculation scheme for the use phase (here shown as 'product stocks'). Stock levels are determined from historic stocks and scenarios following different storylines. The stock-driven model then determines the age-cohort decomposition of the in-use stock as well as product inflows and outflows and the associated material content. With the total stock broken down into different age-cohorts by the stock-driven model, the function and energy flows of the use phase can then be determined (cf. below) by applying the following parameters in turn: intensity of operation and intensity of use (for service flows) and energy intensity and energy carrier split (for energy use of the use phase). The indices are as follows (cf. RECC config table and RECC index table): t: time, c: age-cohort, r: region, g: good/commodity/product, S: scenario (SSP, RCP, and/or RE), V: service category, n: energy carrier, t_0: starting time of prospective assessment (2015).The red section of this figure is our interpretation and implementation of the energy service cascade [2].

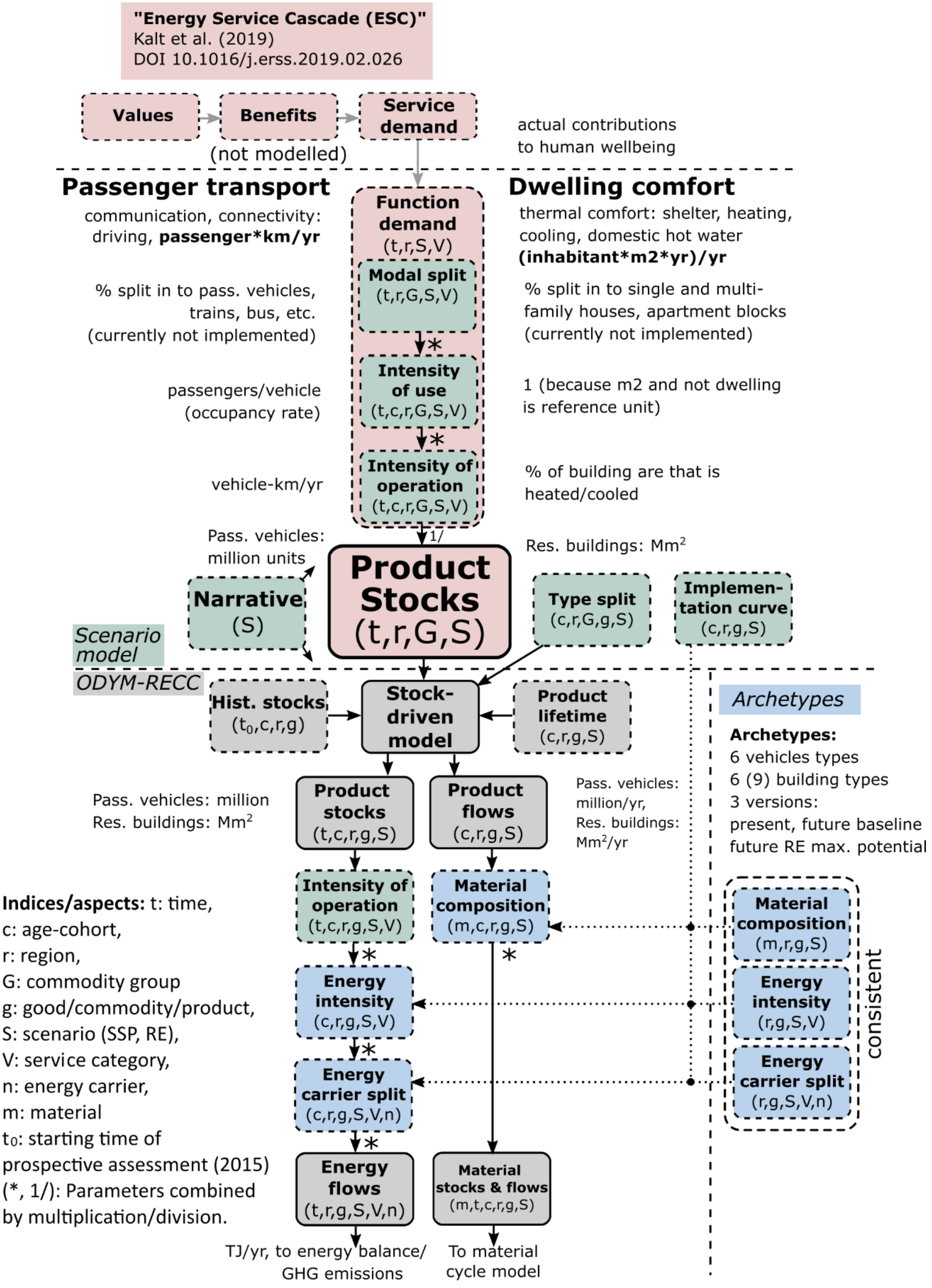

Supplementary Figure 1: Implementation of the energy service cascade and the material cycle model.

3.1. ODYM-RECC model

The ODYM-RECC model (open dynamic material systems model for the resource efficiency and climate change mitigation project) is a modular depiction of major end-use sectors and the material cycles for the climate-relevant bulk materials [6] (https://github.com/YaleCIE/RECC-ODYM). Its system definition (Supplementary Figure 2) comprises the use phase of materials (in products) and the material cycle stages mining, primary production, manufacturing, waste management and scrap recovery, and remelting/recycling as well as an energy supply scenario.

ODYM-RECC generates a set of what-if scenarios [27] for the climate-relevant end-use sectors and bulk material cycles against different socioeconomic, technology deployment, and climate policy backgrounds. It does so by applying a mass-balanced framework for the material cycles [24]. It allows us to study the impacts of a broad spectrum of sustainable development strategies on the material cycles and identify trade-offs and constraints. It does not assess the likelihood of realisation of any of the scenarios studied but checks if mass balance constraints (e.g. by long product lifetimes or limited scrap supply) render some scenarios unfeasible from a material cycle point of view.

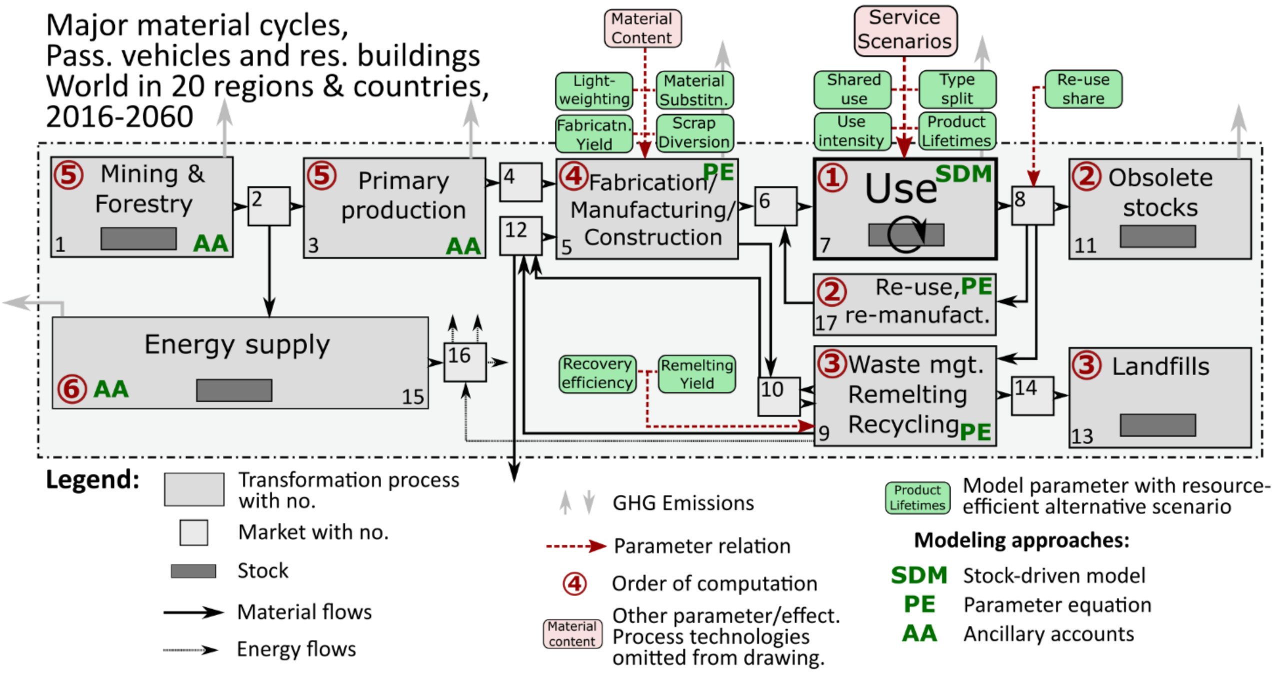

Supplementary Figure 2: ODYM-RECC v2.4 system definition

ODYM-RECC is a multi-layer model depicting products, materials, chemical elements, energy flows, and emissions, with mass balance across all processes down to the individual chemical element. ODYM-RECC has six modules that quantify the system in Supplementary Figure 2 by translating a given service scenario into product stocks, inflows and outflows (module 'use phase UP', using stock-driven modelling [23], product outflows into scrap and recycled materials (module 'waste management and recycling WR', using parameter equations), product inflows

into material demand and fabrication scrap (module 'manufacturing MF' using parameter equations), material demand into primary production and related impacts (module 'primary production PP', using environmental extension factors), and by determining the chemical element composition of al stocks and flows (module 'material-element composition ME', using mass balance). Finally, the energy consumption and environmental pressure and impact indicators are calculated (module 'energy and extensions EX').

For the RECC project, 35 data aspects (time, age-cohort, process, material, chemical element, waste/scrap, environmental extension, socioeconomic scenario…) were defined and each of the 104 model parameters has a specific data model that links it to the data aspects. For example, the parameter for the product lifetime extension potential has the three aspects 'product', 'region', and 'scenario. The parameter for the future stock levels needed has the four aspects 'scenario', 'product', 'region', and 'time'. The resolution of each data aspect is defined in the model configuration file, a summary is given in Supplementary Table 4.

Supplementary Table 4: ODYM-RECC model and data resolution.

Model and data aspect	Resolution
Time	2016-2060 in steps of 1 year
Age-cohorts/Vintages	Vehicles: 1980-2060, residential and non-residential buildings: 1900-2060, appliances: 1971-2060, industry (electricity generation assets): 1986-2060.
Regions	For passenger vehicles and residential buildings: 20 countries and world regions, covering the entire world. For non-residential buildings and appliances: one aggregate global region. For industrial assets (electricity generation): 11 world regions.
Products	6 passenger vehicle types, 13 residential building types, 4 non-residential building types, 18 electricity generation technologies, and 12 types of appliances.
Engineering materials	construction grade steel, automotive steel, stainless steel, cast iron, wrought Al, cast Al, copper electric grade, plastics, wood and wood products, zinc, concrete
Waste and scrap types	heavy melt, plate, and structural steel scrap; steel shred; Al extrusion scrap, auto rims, clean; Al old sheet and construction waste; Al old cast; copper wire scrap; construction waste, concrete, bricks, tiles, ceramics
Chemical elements	C, Al, Cr, Fe, Cu, Zn, 'other'
Energy carriers	Electricity, coal, hard coal, diesel, gasoline, natural gas, hydrogen, fuel wood
Service categories	Driving (vehicles), heating, cooling, domestic hot water (residential and non-residential buildings)
Scenarios	Socioeconomic: Low energy demand (LED), SSP1, SSP2<br>Climate policy: No policy after 2020 (reference scenario), 2 degrees Celsius (66%), corresponding to RCP2.6 forcing pathway.

The model parameters are linked to the system variables (stocks and flows shown in Supplementary Figure 2) via the model equations, which are grouped into the five ODYM-

RECC modules. The parameters are divided into three groups: socioeconomic parameters such as future population, service demand, or intensity of operation of stocks (e.g. vehicle-km per year), technology parameters like energy efficiency of stock operation of the future emissions intensity of energy supply, and resource efficiency parameters describing both the potential for resource efficiency at the different stages of the system (green boxes in Supplementary Figure 2), and the speed of implementation of these potentials under different socioeconomic and climate policy scenarios.

Each RE strategy can be implemented separately or as part of a cascade of strategies. The model allows for calculating the impact of one strategy at a time (sensitivity analysis) or a bundle of strategies in different orders of implementation, each for different socioeconomic and climate policy scenarios.

3.2. Overview table with main assumptions

The following table shows the main quantitative assumptions behind the RECC parameter datasets. For a complete overview, we refer to the RECC scenario publication [4] and database (https://doi.org/10.5281/zenodo.4671643).

The entire model and its complete input and result databases are released under a permissive license on https://github.com/YaleCIE/RECC-ODYM and on Zenodo:

RECC Global input database: https://doi.org/10.5281/zenodo.4671643

RECC Global results: https://zenodo.org/record/4698619

Supplementary Table 5. RECC Global main input data and assumptions

	**LED**	**SSP1**	**SSP2**
**2050 world population (Million)**	8937	8246	8937
**Global North average passenger-km/yr 2016/2050**	**2016:** 6360<br>**2050 :** 5990	**2016:** 6470<br>**2050 :** 8600	**2016:** 6570<br>**2050 :** 10800
**Global North average heated residential building m^2/cap 2016/2050**	**2016:** 27.3<br>**2050 (no ME):** 28.3<br>**2050 (full ME):** 28.3	**2016:** 27.5<br>**2050 (no ME):** 40<br>**2050 (full ME):** 31.5	**2016:** 27.7<br>**2050 (no ME):** 48.5<br>**2050 (full ME):** 39
**Global North average cooled residential building m^2/cap 2016/2050**	**2016:** 28.8<br>**2050 (no ME):** 19.4<br>**2050 (full ME):** 19.4	**2016:** 28.9<br>**2050 (no ME):** 30<br>**2050 (full ME):** 24.5	**2016:** 29<br>**2050 (no ME):** 42<br>**2050 (full ME):** 33.6
**Global South average passenger-km/yr 2016/2050**	**2016:** 970<br>**2050 :** 1610	**2016:** 1100<br>**2050 :** 4050	**2016:** 1200<br>**2050 :** 5900
**Global South average heated residential building m^2/cap 2016/2050**	**2016:** 11<br>**2050 (no ME):** 18<br>**2050 (full ME):** 18	**2016:** 11<br>**2050 (no ME):** 22<br>**2050 (full ME):** 18.6	**2016:** 11<br>**2050 (no ME):** 27.3<br>**2050 (full ME):** 22.1
**Global South average cooled residential building m^2/cap 2016/2050**	**2016:** 12.3<br>**2050 (no ME):** 13.8<br>**2050 (full ME):** 13.8	**2016:** 12.4<br>**2050 (no ME):** 22<br>**2050 (full ME):** 18.7	**2016:** 12.4<br>**2050 (no ME):** 33<br>**2050 (full ME):** 26.7
**Material efficiency: (I): Industrial material efficiency, (D): Demand-side material efficiency**			
**Material Efficiency Buildings**			
**End of life recovery (I)**	95% recovery of steel and aluminum, 93% copper, 70% plastics		
**Fabrication yield loss (I)**	Decrease to 10%		
**New scrap diversion (I)**	Up to 80% of all fabrication scrap is used without re-melting		
**Re-use at end of life (I)**	+29% steel reuse, +27% concrete reuse		
**Lifetime extension (D)**	lifetime extended by 90%		
**Material substitution (I)**	85% of new buildings	50% of new buildings	10% of new buildings
**Less material by design (I)**	85% of new buildings	55% of new buildings	35% of new buildings
**More intense use (D)**	None. (Baseline)	-20% of m^2/cap, ≥ LED	-20% of m^2/cap, ≥ LED
**Material Efficiency Vehicles**			
**End of life recovery (I)**	95% recovery of steel and aluminum, 82% copper, 70% plastics		
**Fabrication yield loss (I)**	Decrease to 10%		
**New scrap diversion (I)**	Up to 80% of all fabrication scrap is used without re-melting		
**Re-use at end of life (I)**	20–40% reuse	20–40% reuse	9–20% reuse
**Lifetime extension (D)**	Lifetime of PHEV, BEV, FCV extended by 20%		
**Material substitution (I)**	For 60% of new vehicles	For 60% of new vehicles	28–35% of new vehicles
**Downsizing (D)**	Share of microcars and passenger cars 80–96%	Share of microcars and passenger cars 70–95%	Share of microcars and passenger cars 65–94%
**Car-sharing (D)**	30% service demand through car sharing	25% service demand through car sharing	15% service demand through car sharing
**Ride-sharing (D)**	40% increase in occupancy rate		
**Climate policy parameters**			
**GHG intensity of electricity generation, global average**	241 g CO2-eq/kWh (No new Policy), 87 g CO2-eq/kWh (2°C climate policy)		
**Share of electricity and H_2, vehicles, 2016/2050**	2016: 0%, 2050: 6-7% (No new Policy), 39-40% (2°C climate policy)		
**Share of electricity and H_2, vehicles, 2016/2050**	2016: 33%, 2050: 64-66% (No new Policy), 67-70% (2°C climate policy)		

3.3. The ODYM-RECC Database

The ODYM-RECC v2.4 database contains 104 model parameters of two to six dimensions each. Parameters range from static values (direct emissions of combustion by MJ of energy carrier) to highly detailed highly uncertain datasets (e.g., the future energy carrier split of buildings by region, time, and operation mode (heating/cooling/hot water).

The ODYM-RECC database was compiled as a community effort involving a large number of experts. Data templates and project wide classifications were used to facilitate the compilation of the various types of information.

Depending on data availability, we applied several pathways of data compilation, which are listed and described in detail below.

- Extract mostly socioeconomic parameters from existing scenario models (scenario reference)
- Compile own plausible scenario estimates for socioeconomic parameters in line with the different scenario narratives where established model framework results are not available (group consensus scenarios)
- Extract process-, product, and material-specific data from the engineering and industrial ecology literature (bottom-up data)
- Extract quantitative estimates of resource efficiency strategy potentials, mostly related to prototypes and case studies, from the literature (strategy potentials)
- Simulate energy consumption and material composition of a number of building and vehicle archetypes with specialised software, which are then used as bottom-up product descriptions with and without implementation of RE strategies (archetype descriptions)

3.4. Scenario reference

For the socioeconomic parameters the Shared Socioeconomic Pathways (SSP) database and model results as well as available data from the World Energy Outlook and Energy Technology Perspectives models were used wherever possible, e.g., for future population, future GHG intensity of energy supply, or the drive technology mix for vehicles [28–32]. The data were extracted from available databases (like the SSP scenario database hosted at IIASA: https://www.iiasa.ac.at/web/home/research/researchPrograms/Energy/SSP_Scenario_Database.html) or shared by colleagues, then parsed and reviewed by the RECC team, then aggregated, disaggregated, and interpolated to fit the ODYM-RECC project-wide classification. For each parameter file the data gathering process is documented both in the respective template files in the RECC database (if only Excel was used), in custom scripts (for more comprehensive datasets) and in the data log files archived under https://github.com/YaleCIE/RECC-data.

Group consensus scenarios: For some parameters like the future stock levels or the split of residential buildings into different types no detailed SSP-consistent scenario calculation was available that we could refer to. Hence we assumed a set of plausible target values for a number of socioeconomic parameters in line with the storylines of the individual socioeconomic scenarios. This process is commonly used when translating broad storylines into high product and regional resolution and sector-specific parameters, cf. Riahi et al. [28] and Grübler et al. [9]. The target values for 2020, 2030, 2040, 2050, and 2060 chosen and the rationale for their choice are documented in scenario target tables, one for each parameter. From there, the target values are read, interpolated, smoothed with a moving average, and exported in ODYM format to be directly used in the ODYM-RECC model. The documentation for the individual parameters is archived in https://github.com/YaleCIE/RECC-data.

The open model and data framework allow for third parties to modify the scenario assumptions and to run calculations with custom parameters and storylines.

Bottom-up data: For the energy intensity, emissions intensity, and material composition of products and processes detailed but representative product or process descriptions were compiled from the literature and available databases. These data include the material composition and specific energy consumption of vehicles and buildings, e.g., [33–35], the loss and recovery rates for the manufacturing and waste management industries e.g., [36,37], and the specific energy consumption and process emissions for the manufacturing, waste management, and primary material production industries [31,32,38,39]. While the data can be regarded as representative of current average global technology, their main limitation is that they are static and no information on their change under different socioeconomic and climate policy scenarios, in particular, is given. To become more realistic a scenario reference was made wherever possible (cf. above), e.g., for the changing GHG intensity of the supply of different energy carriers, for which a combination of MESSAGE IAM results and IEA Energy Technology Perspective results was used. Also, for the average GHG intensity of primary metal production a scenario analysis based on ecoinvent was calculated to take into account scenario-dependent changes of the GHG intensity of electricity generation.

Resource efficiency **Strategy potentials:** For some parameters, including the improvement potentials for fabrication scrap, end-of-life recovery efficiency of scrap, re-use of steel components in buildings, or product lifetime extension, previous estimates can be used [25]. The other strategies were covered by the scenario formulation approach described above.

Archetype descriptions: Here, 'archetype' refers to an idealized description of the physical properties (energy intensity of operation and material composition) of a product with a certain functionality, assuming typical user behaviour in a given region.

For passenger vehicles, drive technology, segment (car size), and material design choice together determine the archetypes' material composition, and the three properties above plus the assumed driving cycle determine its specific operational energy consumption (specific = per km driven).

For residential buildings, building type, energy standard, material intensity (conventional or lightweight design), material design choice, and stylized climate conditions (heating and cooling degree days by region) together determine the archetypes' material composition and specific operational energy consumption (specific = per m^2).

The building and vehicle archetypes differ indeed by their characteristics and are being modelled very differently. The energy source is a discrete property of vehicles and (normally) cannot be changed during its lifetime, so it is considered a defining parameter. In the case of buildings, however, energy supply can be considered independent of a building as it is common for a building to change its thermal energy generator during its lifetime. This is the reason why we decided to use different approaches when modelling vehicle and building archetypes.

The building archetypes have the following discrete dimensions that are being modeled endogenously by the archetype model (BuildME, https://github.com/nheeren/BuildME):

- occupation (3 types: multi family, single-family, residential tower)
- energy standard (4 types: non-standard, standard, efficient, zero-energy)
- resource efficiency strategy (4 types: none, light-weighted, material substitution, combined)
- plus 1 informal type that is considered constant.

In addition to that, the archetypes are simulated for each climate zone individually. Building energy supply is considered exogenously and modeled by the main stock model (ODYM).

The vehicle archetype is mostly characterized by its drive-train technology and has 6 different types, therefore determining the energy demand endogenously. Here the vehicle-specific simulation tools BuildME GREET (https://greet.es.anl.gov/) and FASTSim (https://www.nrel.gov/transportation/fastsim.html) were used to model the archetype descriptions by deriving model estimates for both the material composition and energy intensity of operation for different vehicle configurations.

For each of the nine building and six vehicle types four archetypes, representing maximal potential for change, were simulated: a standard product without special consideration of material efficiency, downsizing, or material substitution, a downsized product, a product with ambitious material substitution, and a downsized material-substituted product.

The beauty of the bottom-up archetype modelling approach is that it can be used to explain very large and heterogeneous product systems. Other modelling approaches, such as top-down or statistical models, have a much lower degree of freedom when it comes to applying engineering strategies (e.g. effect of increased insulation material in different climates). As the reviewer mentions, the trick for the bottom-up approach is to strike a good balance between true representation of the system and model complexity.

In our case we decided to choose the most common technological systems today and what we consider the most hopeful technologies in the intermediate future. In case of the building

archetype model we are somewhat at the upper limit of model complexity. The archetypes represent 3 occupations, 4 energy standards, 4 Resource Efficiency strategies, and approx. 30 climate zones. This results in ca. 1400 manifestations. As we are using complex thermal simulation tools, model runtime is already substantial. Yet we decided to make this trade-off as we deemed all of the aforementioned parameters as important enough to account for them explicitly.

To summarize, for the building archetypes, we take the region-specific climate into account but no behavioural aspects, which is a limitation that we now describe in more detail in the manuscript (see quotation below). We could simulate different material choice scenarios on top of the concrete-wood-substitution that we already model. But this research topic/question would warrant a different paper, ideally, a detailed country-level case study, which the RECC model can also be used for.

For a detailed description and definition of all model aspects, the classifications used for them, the system variables and parameters, the model equation and their division into modules and the data compilation, (dis)aggregation and formatting process, we refer to the ODYM-RECC model documentation.

The ODYM-RECC database is formatted in standardised spreadsheets and archived on Zenodo (dataset DOI https://doi.org/10.5281/zenodo.4671643).

3.5. Model resolution

The information presented here is a summary only. The full info about the resolution of the RECC project is documented in the Master classification file, which is part of the project’s database: **RECC_Classifications_Master_V2.0.xlsx**

Time and age-cohort, dimension: Time:

- The time frame is 1900-2060, as some historic data reach back to 1900 and before. The Actual modelling period is 2016 to 2060, where usually, results until 2050 are extracted for reporting and publication.

Regions, dimension: Region:

- The two end-use sectors passenger vehicles and residential buildings are implemented for 20 countries and world regions: (cf. RECC_Classifications_Master_V2.0.xlsx for details), See Supplementary Table 6:

Supplementary Table 6: ODYM-RECC model global model regions and their aggregation.

ODYM-RECC region (20)	Description	Global North	Global South	EU28
R32CAN	Canada	**X**		
R32CHN	China	**X**		
R32EU12-M	"New" EU countries, medium income	**X**		**X**
R32IND	India		**X**	
R32JPN	Japan	**X**		
R32USA	USA	**X**		
France	France	**X**		**X**
Germany	Germany	**X**		**X**
Italy	Italy	**X**		**X**
Poland	Poland	**X**		**X**
Spain	Spain	**X**		**X**
UK	UK	**X**		**X (not in EU27)**
Oth_R32EU15	Other "old" EU countries,	**X**		**X**
Oth_R32EU12-H	Other "new EU countries, high income	**X**		**X**
R5.2OECD_Other	Other OECD countries	**X**		
R5.2REF_Other	Countries of the former USSR	**X**		
R5.2ASIA_Other	Other Asian countries		**X**	
R5.2MNF_Other	Middle East and Northern African Countries		**X**	
R5.2SSA_Other	Sub-Saharan Africa Country		**X**	
R5.2LAM_Other	Latin-American Countries		**X**	

Eningering_Materials, dimension: Material:

- Construction grade steel
- Automotive steel
- stainless steel
- Cast iron
- Wrought Al
- Cast Al
- Copper electric grade
- Plastics
- Cement
- Wood and wood products
- Zinc
- Concrete
- Concrete aggregates

UsePhase, dimension: Process:

- Cf. Products resolution

Products, dimension: Good_Product:

- **Passenger vehicles:**
  Internal Combustion Engine, gasoline (ICEG)
  Internal Combustion Engine, diesel (ICED)
  Hybrid Electric Vehicles (HEV)
  Plugin Hybrid Electric Vehicles (PHEV)
  Battery Electric Vehicles (BEV)
  Fuel Cell Vehicles (FCV)

EoL goods, dimension: Good_Product:

- Cf. Products resolution

Energy, dimension: Energy carriers:

- Electricity
- Coal, hard coal
- Diesel
- Gasoline
- Natural gas
- Hydrogen
- Fuel wood

SSP_Scenarios, dimension: Scenario:

- LED (low energy demand)
- SSP1 (Shared Socioeconomic Pathway 1)
- SSP2 (Shared Socioeconomic Pathway 2)

RCP_Scenarios, dimension: Scenario:

- RCP2.6, 2°C-pathway
- Baseline (no new climate policy after 2020)

Env. extensions, dimension: Extensions:

- CO_2 emisisons per main output
- CH_4 emissions per main output
- N_2O emissions per main output
- SF_6 emisisons per main output
- GHG emissions
- GHG emissions, supply chain

Env. midpoints, dimension: Extensions:

- GWP 20/100/500
- GTP 20/100/500

Chemical Elements, dimension: Element:

- C
- Al
- Cr
- Fe
- Cu
- Zn
- ‘Other’

MaterialProductionProcess, dimension: Process:

- One (average) primary production process for each material.

ManufacturingProcess, dimension: Process:

- One average manufacturing process for each product/good

Waste management process, dimension: Process:

- One waste mgt. (dismantling, shredding, sorting) process to convert each of the 15 products into waste/scrap at the end of life, one re-melting process for each scrap category

Waste/scrap, dimension: Material:

- Heavy melt, plate, and structural steel scrap
- Steel shred
- Al extrusion scrap, auto rims, clean
- Al old sheet and construction waste
- Al old cast
- Copper wire scrap
- Construction waste, concrete, bricks, tiles, ceramics
- Thermoplastic waste
- Used wood

Car segments, Good_Product:

- microcar
- passenger car
- minivan_SUV
- light truck

3.6. Figure creation and results log for RECC global, v2.4

- Reference Target Table: *RECC_scenario_target_tables_v2_4_RECC_Global_ ARCHIVE.xlsx*, 19.08.2020, 22:05. Archived on Zenodo under https://doi.org/10.5281/zenodo.4671643
- Interpolated target tables and other data and model parameters archived on Zenodo under https://doi.org/10.5281/zenodo.4671643
- RECC Database and results, generated from reference target table and other raw data: 19.08.2020 and later, results for global aggregate regions were run later, after script changes to construct no-efficiency scenarios. Model commit 9c93d9b.
- ODYM-RECC v2.4 global results from August-October of 2020 were evaluated with revised RECC v2.4 evaluation scripts, model commit 9c93d9b, April 2021.
- From Result evaluation run RECC_Results_6a52982d-6a01-48d5-94f3-fdf6876024e2
  - Fig1_select_GHG_pav_reb_SSP1_Gt_v2.png is taken as Fig. 1 of the main paper.
  - Global_pav_reb_GHG_Overview_rel_2050.png is taken as Fig. 2 of the main paper
  - Annual_GHG_2050_MtGlobal_pav_reb_suff_eff_bothcascades.png, Annual_GHG_2050_MtGlobal_North_pav_reb_suff_eff_bothcascades.png, and Annual_GHG_2050_MtGlobal_South_pav_reb_suff_eff_bothcascades.png are combined together to form Fig. 3 of the paper. (using GIMP)
  - Global_pav_reb_nrb_Materials_SSP1_RCP2_6_line_v2.png is combined with pC_Stocks_line_SSP1.png and the two legends, Legend_Matflows.png and Legend_Matstocks.png, to form Fig. 4 of the main paper. (using GIMP)

  - All numerical results are archived on Zenodo under https://zenodo.org/record/4698619

- Those number that are plotted or directly reported in the paper are archived in supplementary material 3 (Excel-File available from the journal homepage). This supplement contains the data that appear in the plots in the paper. The result evaluation run RECC_Results_6a52982d-6a01-48d5-94f3-fdf6876024e2 generated a number of .xls files, the following of which contain the data in the figures:
  - Sheet "Fig. 1": Data extracted from the export summary files GHG_Area_Data_pav[reb]_[Region].xls. Plotted values are highlighted red. Unit: Mt CO2-eq.
  - Sheet "Fig. 2": Data extracted from the export summary files GHG_Cascade_Data_pav_reb_Global.xls. Plotted values are highlighted red. Unit: Mt CO2-eq.
  - Sheet "Fig. 3": Data extracted from the export summary files ME_industry_demand_cascadeGlobal[North/South].xls. Plotted values are highlighted red. Unit: Mt CO2-eq.
  - Sheet "Fig. 4a": Data extracted from the export summary files PrimaryMaterial_pav_reb_Global.xls and SecondaryMaterial_pav_reb_Global.xls. Plotted values are highlighted red. Unit: Mt/yr.
  - Sheet "Fig. 4b": Data extracted from the export summary files pC_MatStocks_tonsPcap_pav_reb_Global[North/South].xls. Plotted values are highlighted red. Unit: ton/cap.

4. RECC 2.4 database and scenario drivers

Here, we list the changes in the main drivers and the country/region-level plots (Supplementary Figure 3).

2016-2060 relative change in main material cycle drivers

			LED	SSP1	SSP2
France	RCP2.6	Population	→ 0%	→ 0%	→ 0%
France	RCP2.6	per capita in-use stock, residential buildings	↘ -26%	→ 0%	→ 16%
France	RCP2.6	per capita in-use stock, passenger vehicles	↘ -10%	→ 0%	↗ 44%
Germany	RCP2.6	Population	→ 0%	→ 0%	→ 0%
Germany	RCP2.6	per capita in-use stock, residential buildings	↘ -28%	→ 0%	→ 18%
Germany	RCP2.6	per capita in-use stock, passenger vehicles	↘ -19%	→ 0%	↗ 48%
Italy	RCP2.6	Population	→ 0%	→ 0%	→ 0%
Italy	RCP2.6	per capita in-use stock, residential buildings	↘ -30%	→ 0%	→ 17%
Italy	RCP2.6	per capita in-use stock, passenger vehicles	↘ -20%	→ 0%	↗ 62%
Oth_R32EU12-H	RCP2.6	Population	↘ -8%	→ 0%	↘ -8%
Oth_R32EU12-H	RCP2.6	per capita in-use stock, residential buildings	↘ -21%	→ 0%	→ 18%
Oth_R32EU12-H	RCP2.6	per capita in-use stock, passenger vehicles	↘ -21%	→ 0%	↗ 31%
Oth_R32EU15	RCP2.6	Population	→ 0%	→ 0%	→ 0%
Oth_R32EU15	RCP2.6	per capita in-use stock, residential buildings	↘ -25%	→ 0%	→ 18%
Oth_R32EU15	RCP2.6	per capita in-use stock, passenger vehicles	↘ -14%	→ 0%	↗ 41%
Poland	RCP2.6	Population	→ 0%	→ 0%	→ 0%
Poland	RCP2.6	per capita in-use stock, residential buildings	↘ -21%	→ 0%	→ 18%
Poland	RCP2.6	per capita in-use stock, passenger vehicles	↘ -20%	→ 0%	→ 26%
R5.2ASIA_Other	RCP2.6	Population	→ 14%	→ 0%	→ 14%
R5.2ASIA_Other	RCP2.6	per capita in-use stock, residential buildings	↘ -14%	→ 0%	→ 14%
R5.2ASIA_Other	RCP2.6	per capita in-use stock, passenger vehicles	↓ -75%	→ 0%	↑ 99%
R5.2LAM_Other	RCP2.6	Population	→ 13%	→ 0%	→ 13%
R5.2LAM_Other	RCP2.6	per capita in-use stock, residential buildings	↘ -13%	→ 0%	↗ 31%
R5.2LAM_Other	RCP2.6	per capita in-use stock, passenger vehicles	↓ -75%	→ 0%	↑ 99%
R5.2MNF_Other	RCP2.6	Population	→ 15%	→ 0%	→ 15%
R5.2MNF_Other	RCP2.6	per capita in-use stock, residential buildings	↘ -25%	→ 0%	→ 13%
R5.2MNF_Other	RCP2.6	per capita in-use stock, passenger vehicles	↓ -53%	→ 0%	↑ 76%
R5.2OECD_Other	RCP2.6	Population	→ 8%	→ 0%	→ 8%
R5.2OECD_Other	RCP2.6	per capita in-use stock, residential buildings	↘ -25%	→ 0%	→ 13%
R5.2OECD_Other	RCP2.6	per capita in-use stock, passenger vehicles	↘ -38%	→ 0%	↗ 36%
R5.2REF_Other	RCP2.6	Population	→ 9%	→ 0%	→ 9%
R5.2REF_Other	RCP2.6	per capita in-use stock, residential buildings	↘ -25%	→ 0%	→ 13%
R5.2REF_Other	RCP2.6	per capita in-use stock, passenger vehicles	↘ -38%	→ 0%	↗ 36%
R5.2SSA_Other	RCP2.6	Population	→ 19%	→ 0%	→ 19%
R5.2SSA_Other	RCP2.6	per capita in-use stock, residential buildings	↘ -29%	→ 0%	→ 25%
R5.2SSA_Other	RCP2.6	per capita in-use stock, passenger vehicles	↓ -75%	→ 0%	↑ 99%
R32CAN	RCP2.6	Population	→ -3%	→ 0%	→ -3%
R32CAN	RCP2.6	per capita in-use stock, residential buildings	↓ -42%	→ 0%	→ 24%
R32CAN	RCP2.6	per capita in-use stock, passenger vehicles	↘ -26%	→ 0%	↗ 40%
R32CHN	RCP2.6	Population	→ 4%	→ 0%	→ 4%
R32CHN	RCP2.6	per capita in-use stock, residential buildings	↘ -25%	→ 0%	→ 25%
R32CHN	RCP2.6	per capita in-use stock, passenger vehicles	↘ -37%	→ 0%	↗ 63%
R32EU12-M	RCP2.6	Population	→ 4%	→ 0%	→ 4%
R32EU12-M	RCP2.6	per capita in-use stock, residential buildings	↘ -21%	→ 0%	→ 18%
R32EU12-M	RCP2.6	per capita in-use stock, passenger vehicles	↘ -18%	→ 0%	↗ 36%
R32IND	RCP2.6	Population	→ 16%	→ 0%	→ 16%
R32IND	RCP2.6	per capita in-use stock, residential buildings	↘ -13%	→ 0%	↗ 33%
R32IND	RCP2.6	per capita in-use stock, passenger vehicles	↓ -65%	→ 0%	↑ 93%
R32JPN	RCP2.6	Population	↘ -6%	→ 0%	↘ -6%
R32JPN	RCP2.6	per capita in-use stock, residential buildings	↘ -23%	→ 0%	→ 19%
R32JPN	RCP2.6	per capita in-use stock, passenger vehicles	↘ -28%	→ 0%	↑ 91%
Spain	RCP2.6	Population	→ 0%	→ 0%	→ 0%
Spain	RCP2.6	per capita in-use stock, residential buildings	↘ -21%	→ 0%	→ 19%
Spain	RCP2.6	per capita in-use stock, passenger vehicles	↘ -13%	→ 0%	↗ 37%
UK	RCP2.6	Population	→ 0%	→ 0%	→ 0%
UK	RCP2.6	per capita in-use stock, residential buildings	↘ -25%	→ 0%	→ 13%
UK	RCP2.6	per capita in-use stock, passenger vehicles	↘ -11%	→ 0%	↗ 43%
R32USA	RCP2.6	Population	→ -3%	→ 0%	→ -3%
R32USA	RCP2.6	per capita in-use stock, residential buildings	↓ -44%	→ 0%	↗ 30%
R32USA	RCP2.6	per capita in-use stock, passenger vehicles	↘ -19%	→ 0%	↑ 67%

Supplementary Figure 3: Overview of major material cycle drivers: cars, residential buildings, and population.

Supplementary Figures 4.1 –4.20 below show the temporal development of the main drivers and stock parameters by country/region for both the residential building and the passenger vehicle sectors.

Legend for all figures: First row: residential floor space: buildings in m²/cap; share of light-weighted buildings in new construction, unit: 1; share of material-substituted (timber-intensive) buildings in new construction, unit: 1. Second row: population, million; car ownership rate, cars per person; share of downsized (segment-shifted) cars in newly registered cars, unit: 1; share of material-substituted (light-weighted) cars in newly registered cars, unit: 1. Third row: OR: Vehicle occupancy rate, passengers per car; PKM: total passenger-km delivered by entire fleet, billion km; VKM: total vehicle-km delivered by entire fleet, billion km)

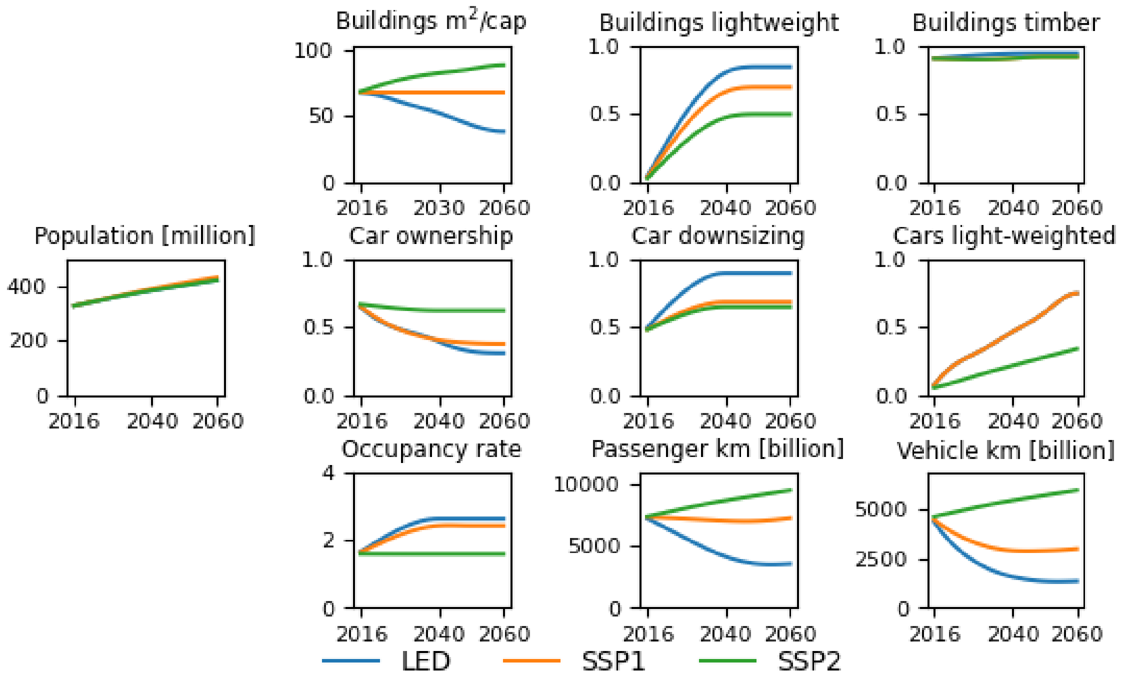

Supplementary Figure 4.1: Drivers and in-use stock parameters, USA.

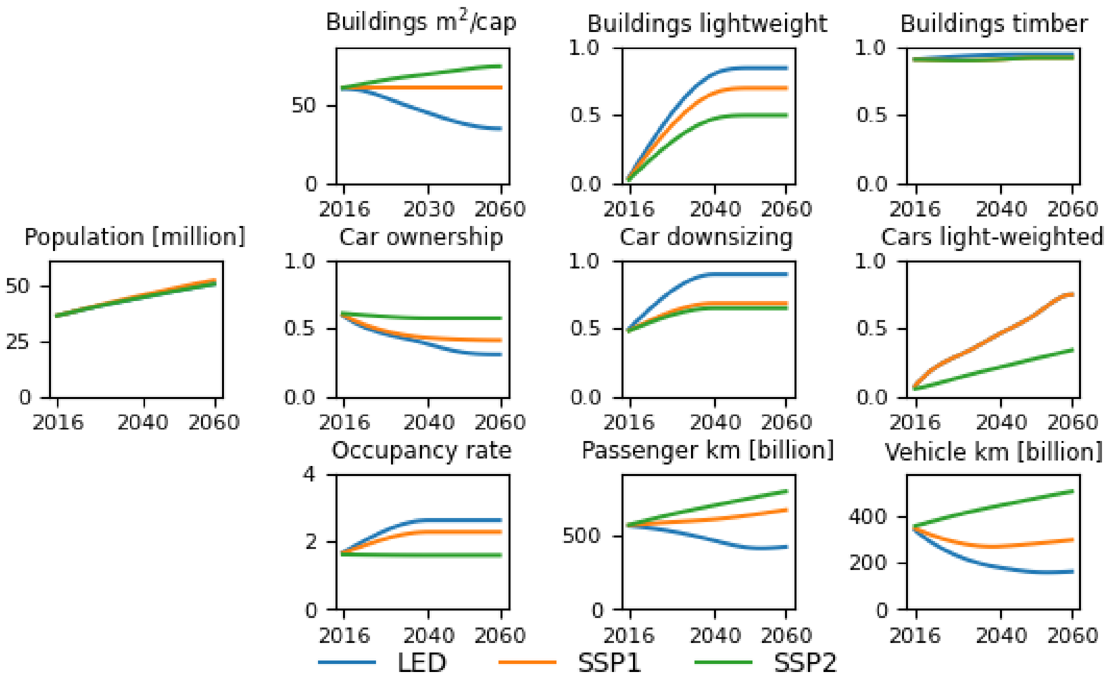

Supplementary Figure 4.2: Drivers and in-use stock parameters, Canada.

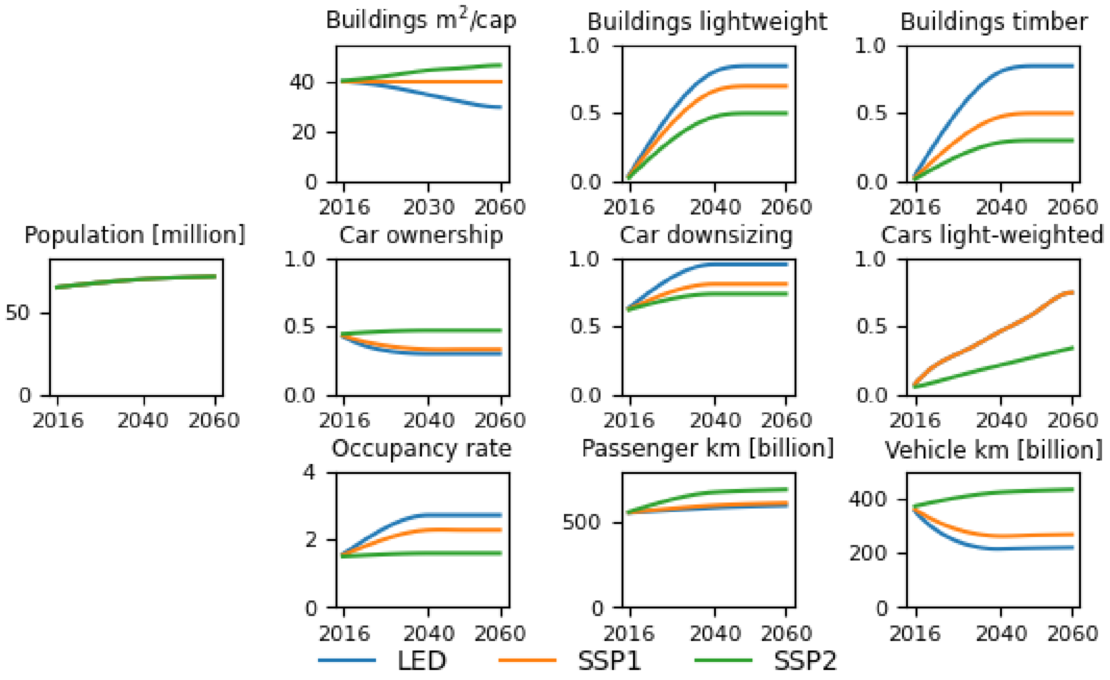

Supplementary Figure 4.3: Drivers and in-use stock parameters, France.

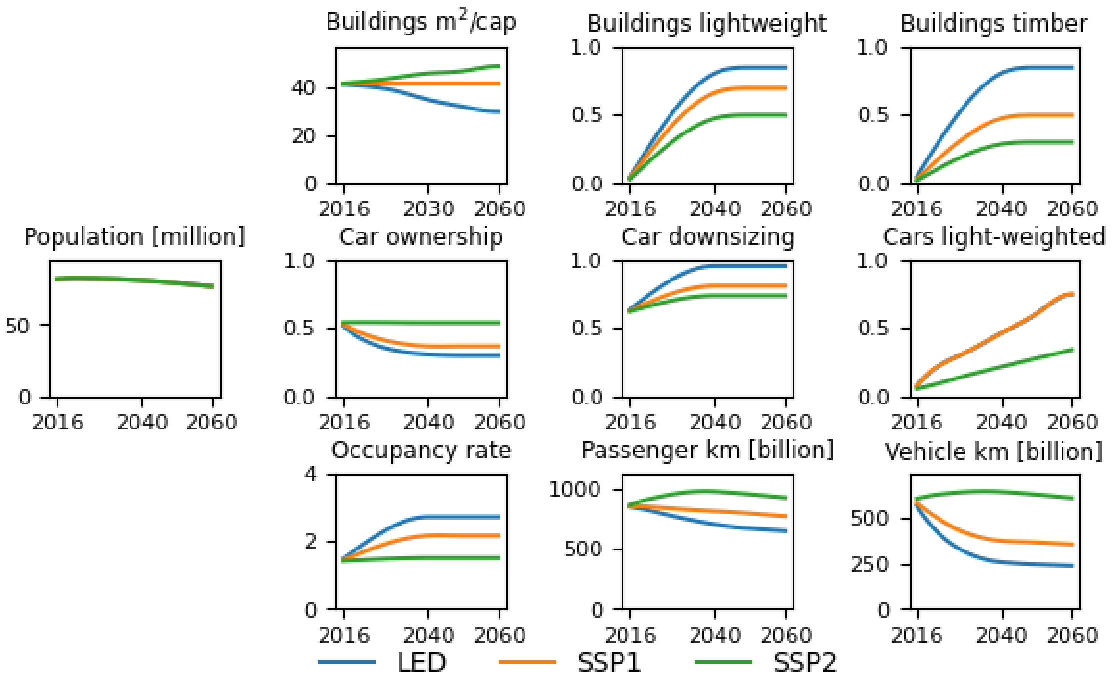

Supplementary Figure 4.4: Drivers and in-use stock parameters, Germany.

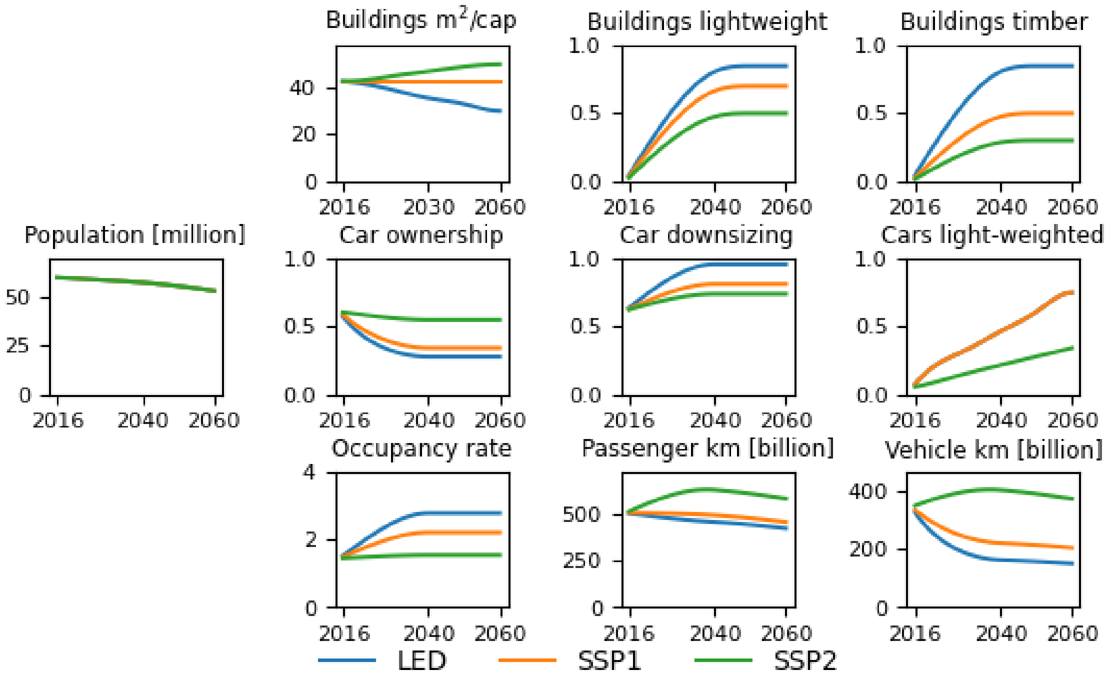

Supplementary Figure 4.5: Drivers and in-use stock parameters, Italy.

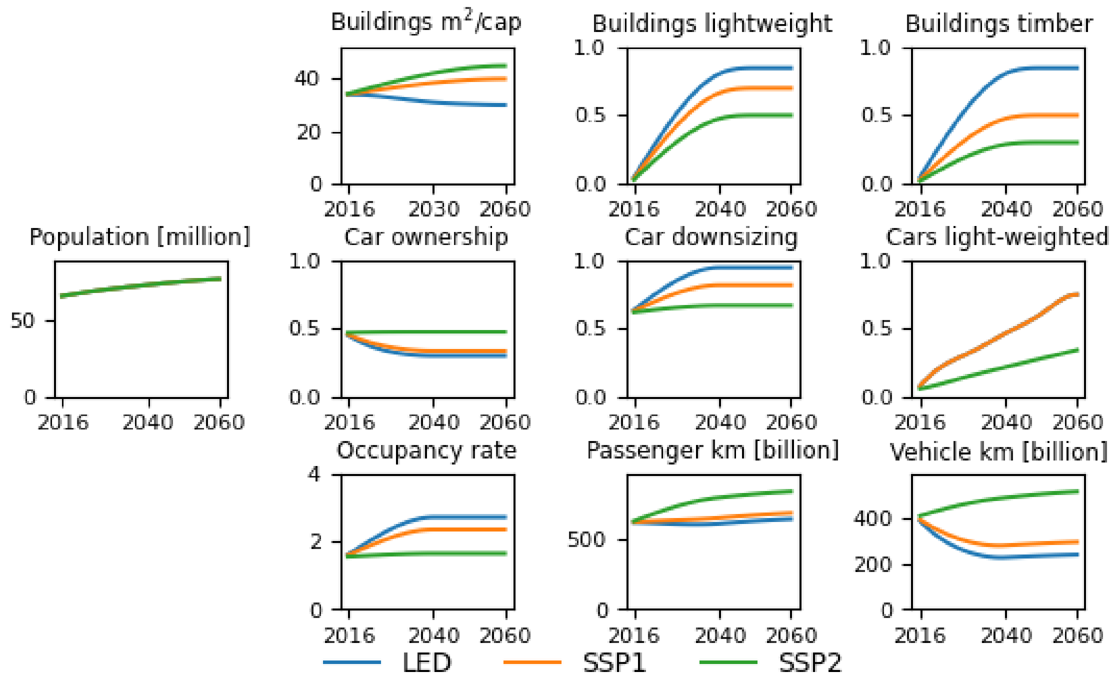

Supplementary Figure 4.6: Drivers and in-use stock parameters, UK.

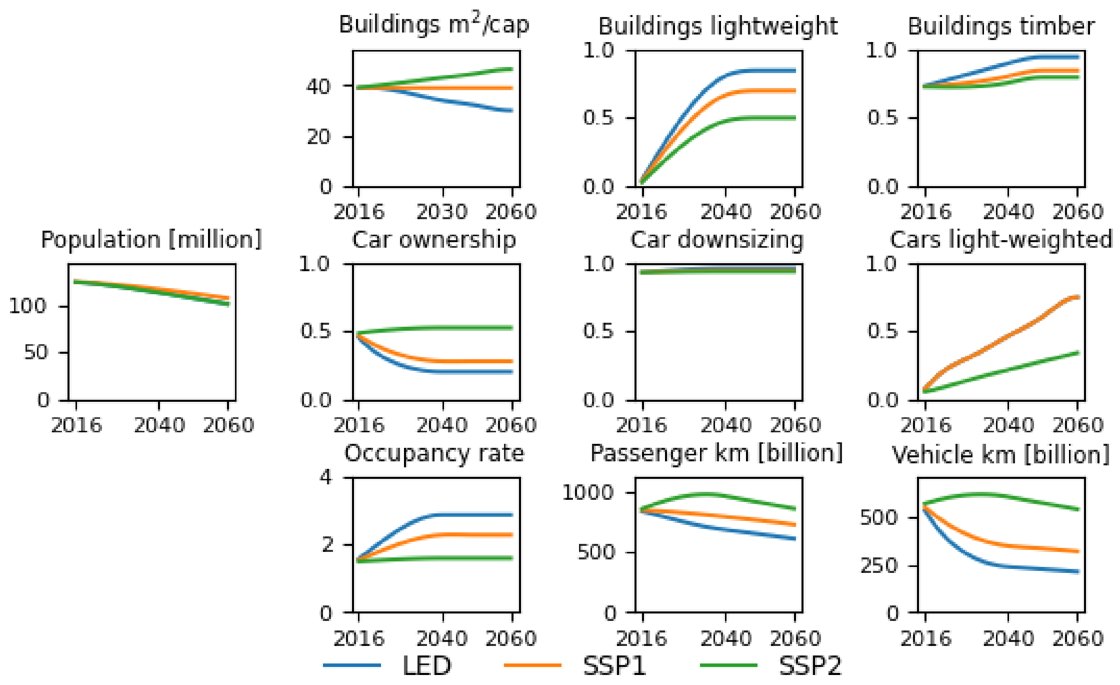

Supplementary Figure 4.7: Drivers and in-use stock parameters, Japan.

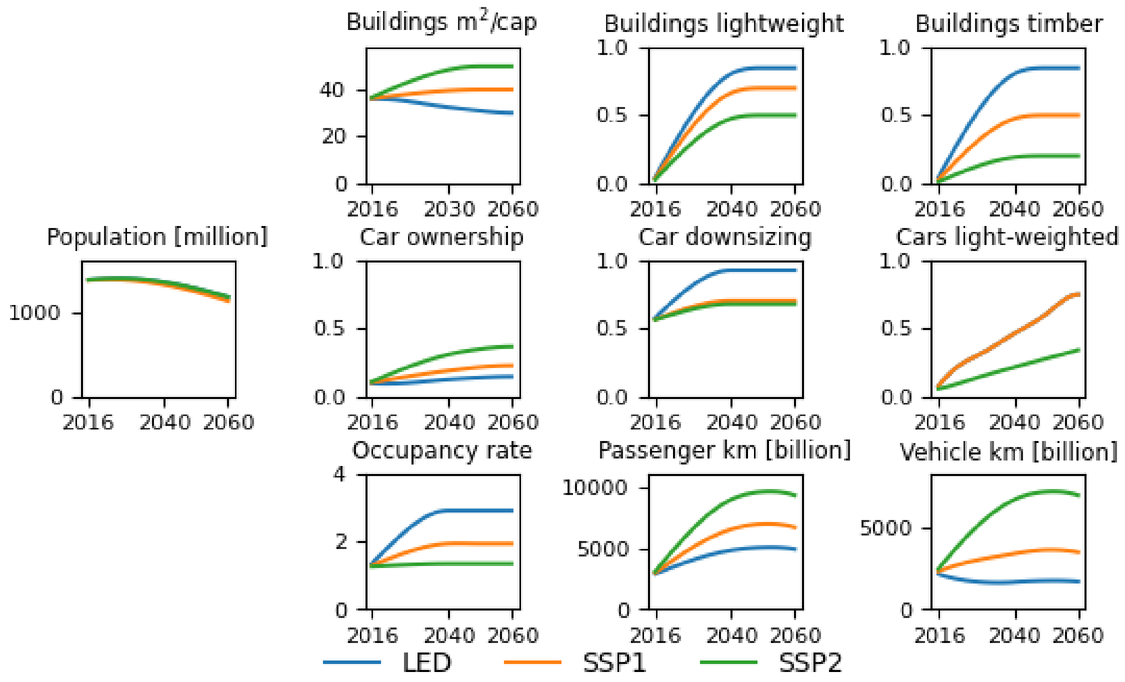

Supplementary Figure 4.8: Drivers and in-use stock parameters, China.

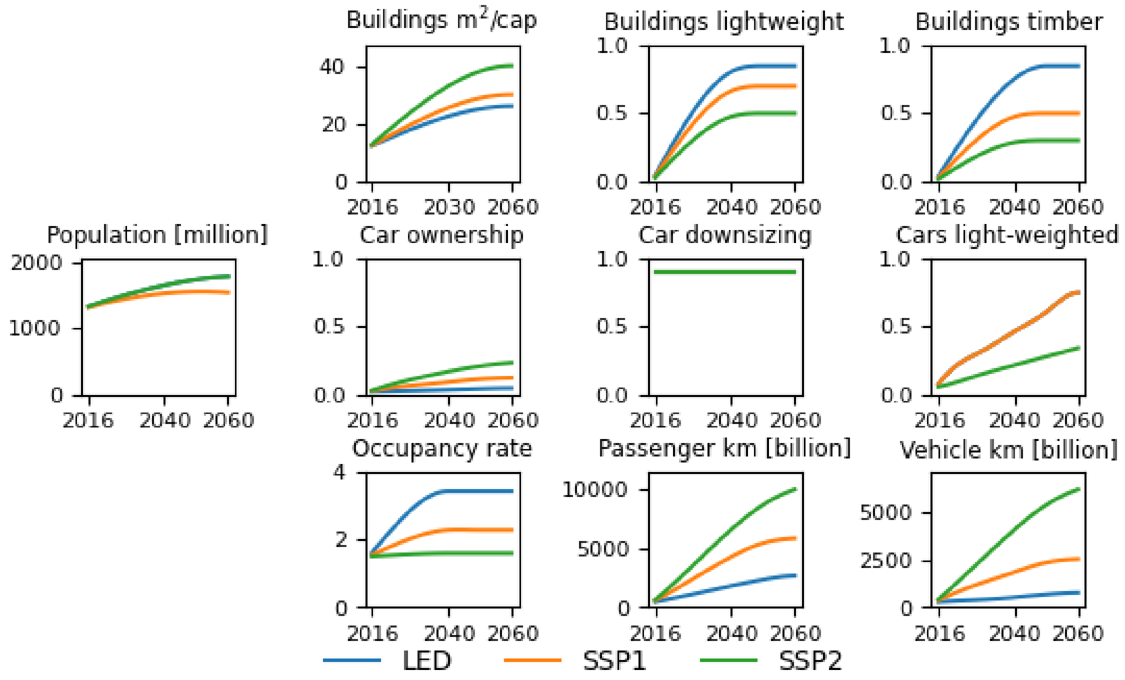

Supplementary Figure 4.9: Drivers and in-use stock parameters, India.

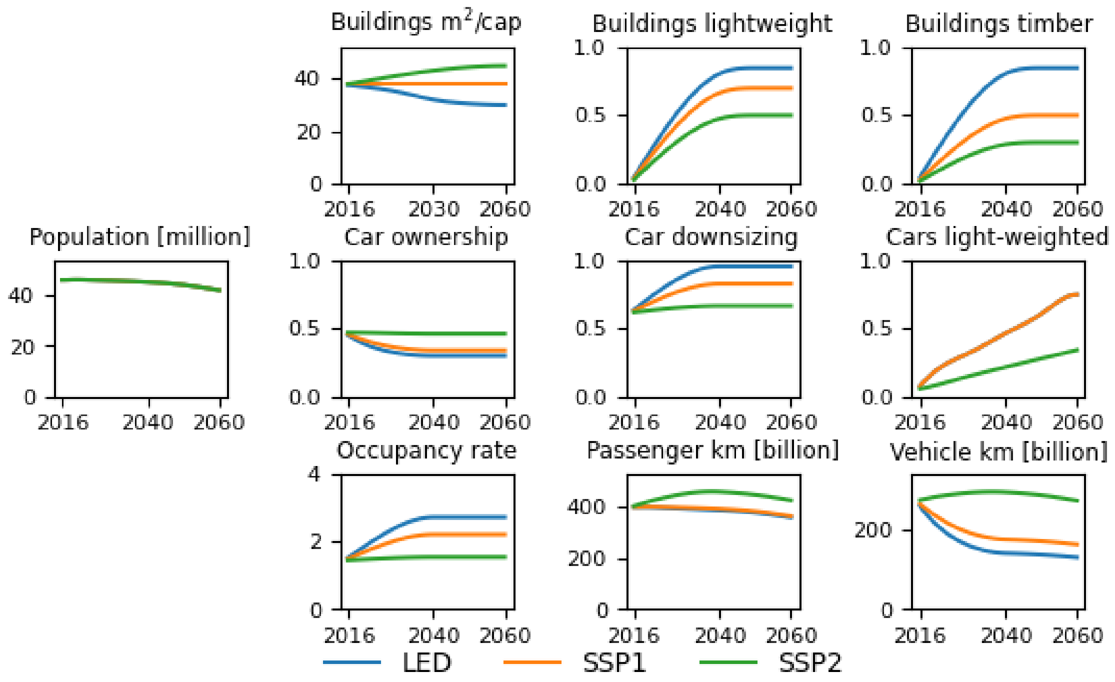

Supplementary Figure 4.10: Drivers and in-use stock parameters, Spain.

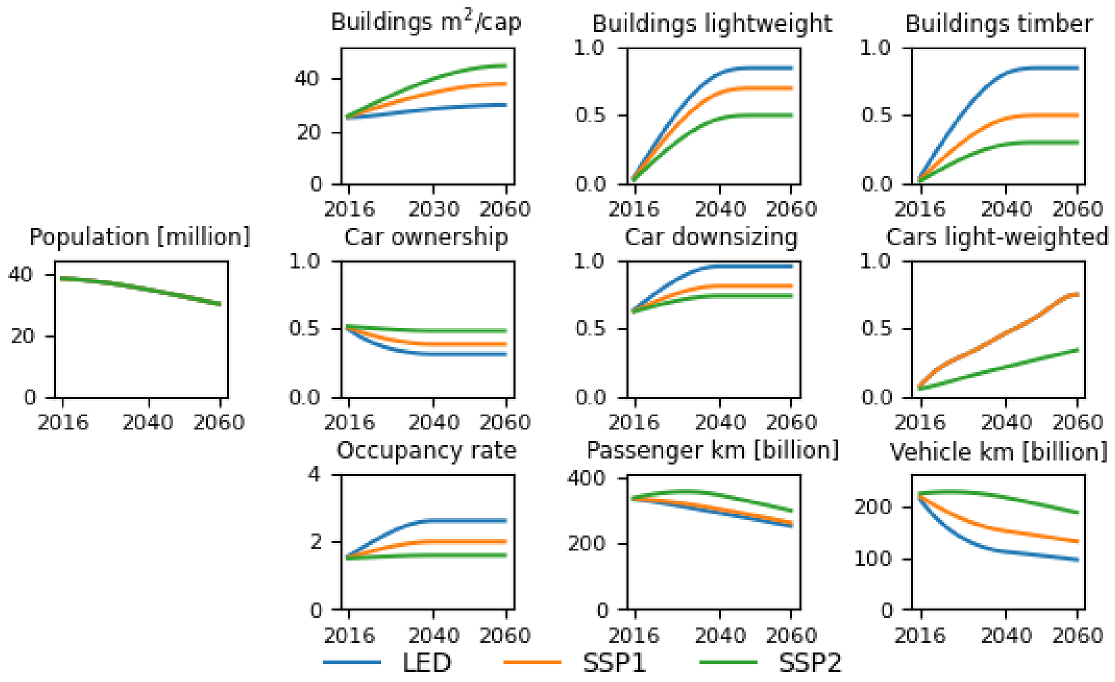

Supplementary Figure 4.11: Drivers and in-use stock parameters, Poland.

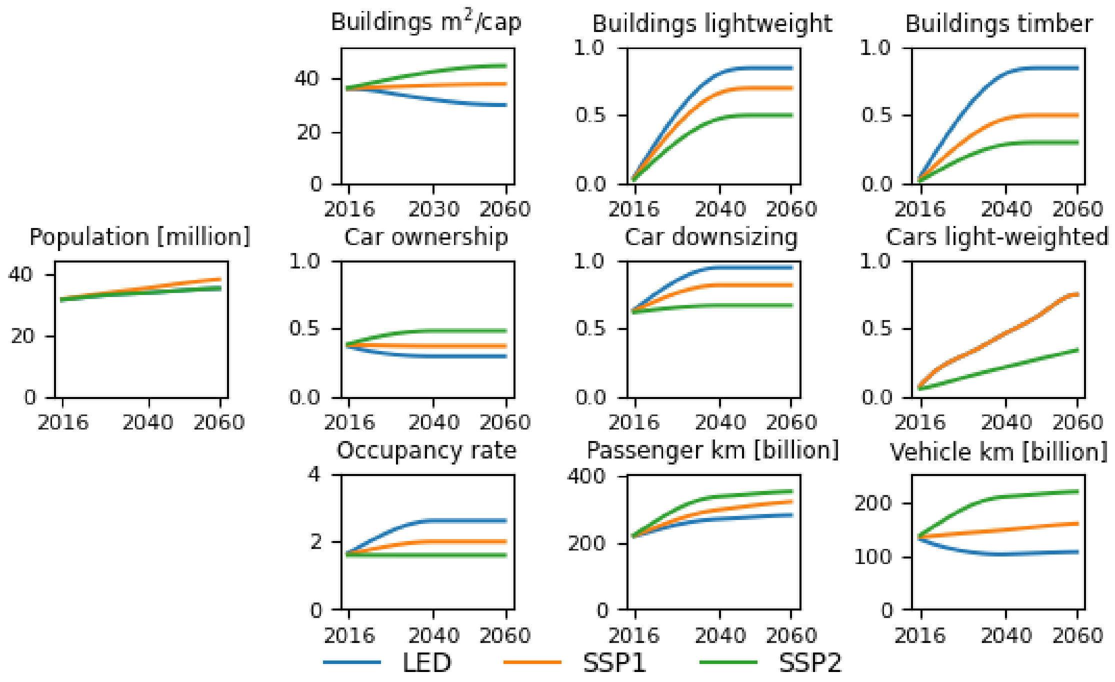

Supplementary Figure 4.12: Drivers and in-use stock parameters, R32EU12-H.

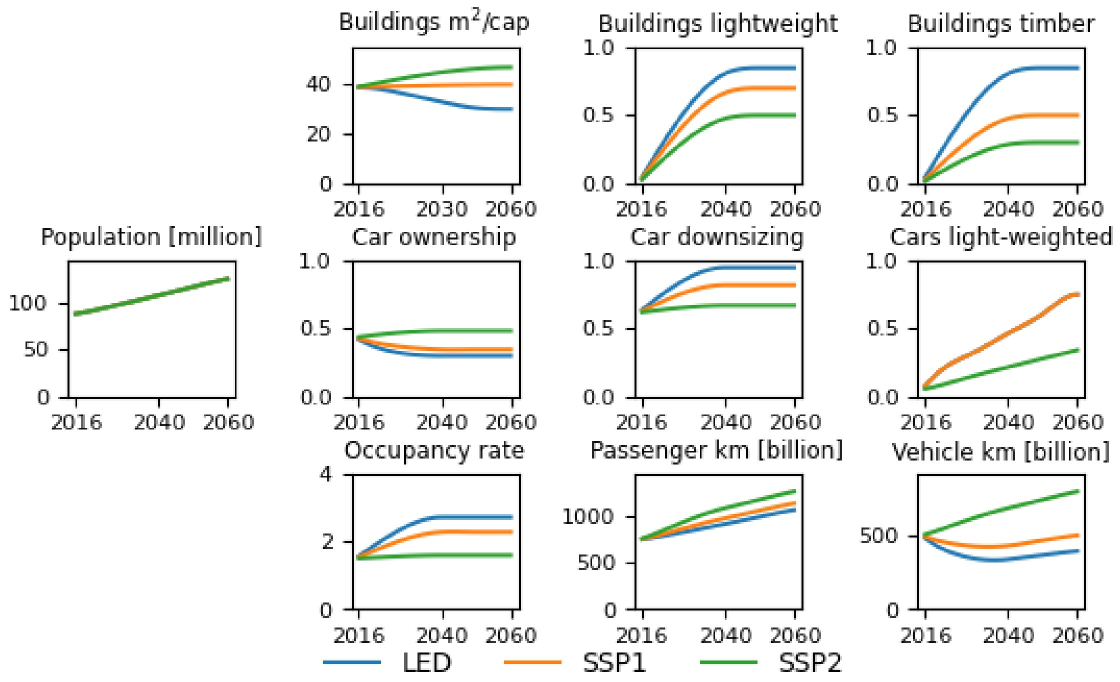

Supplementary Figure 4.13: Drivers and in-use stock parameters, R32EU15.

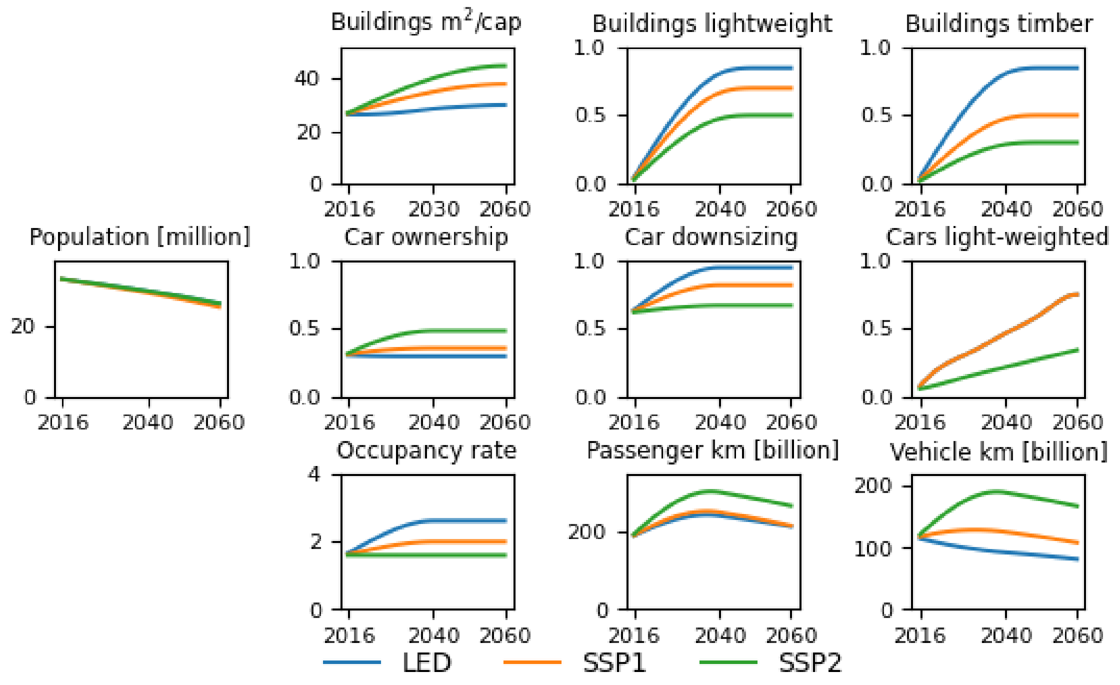

Supplementary Figure 4.14: Drivers and in-use stock parameters, R32EU12-M.

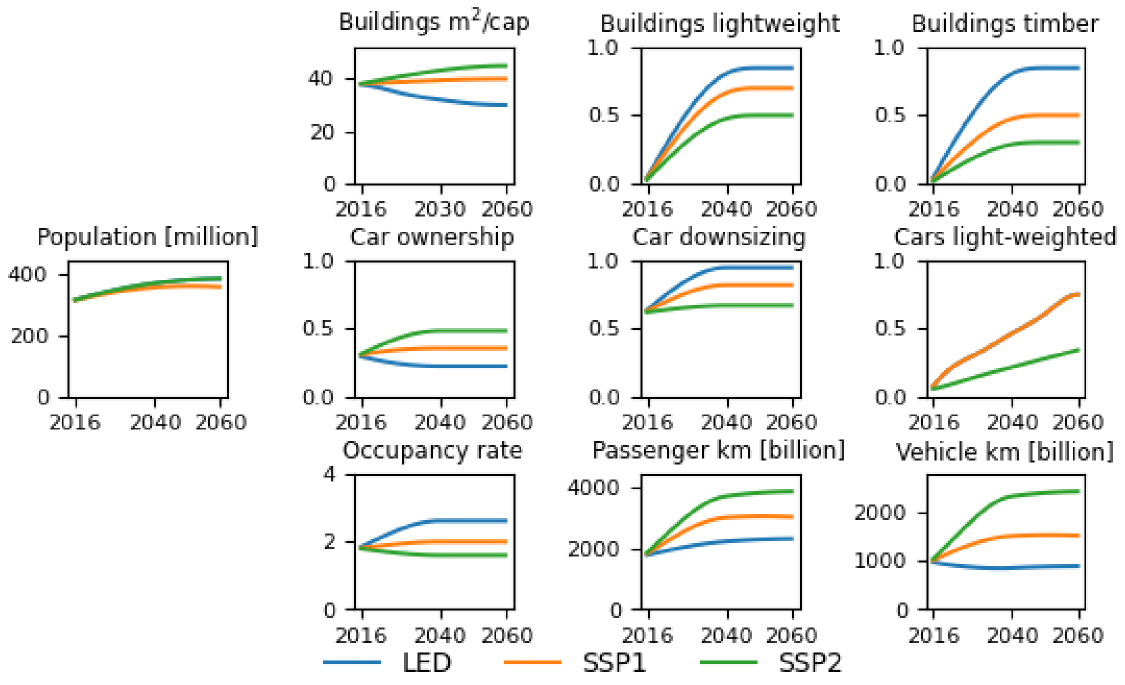

Supplementary Figure 4.15: Drivers and in-use stock parameters, R5.2OECD_Other.

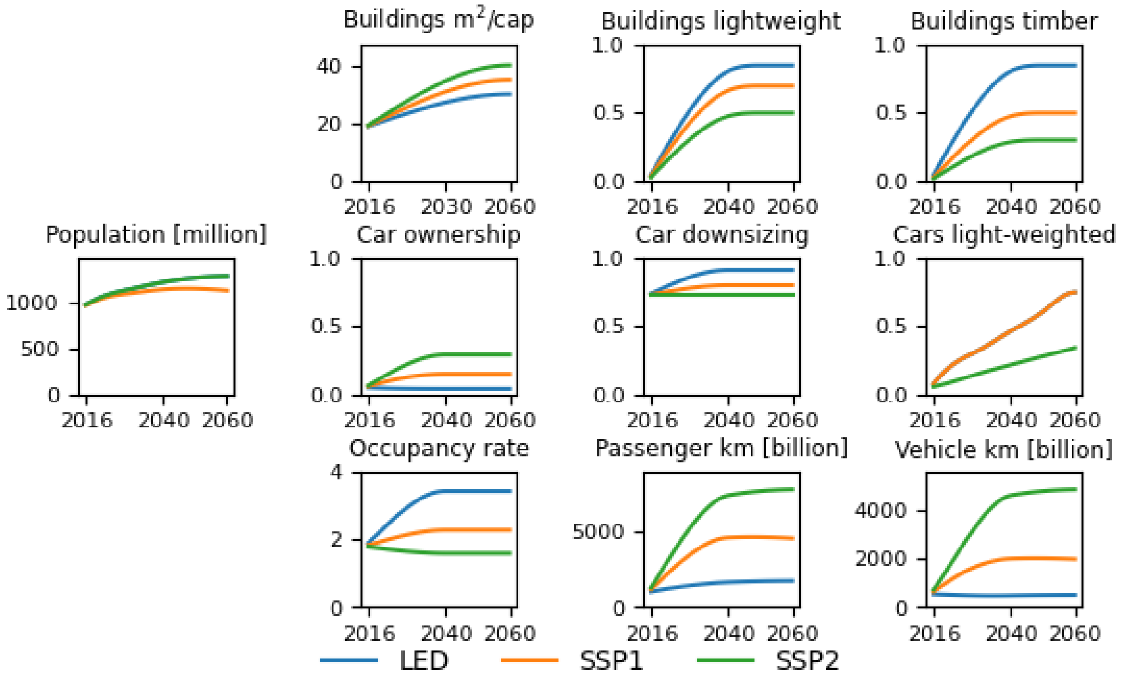

Supplementary Figure 4.16: Drivers and in-use stock parameters, R5.2Asia_Other.

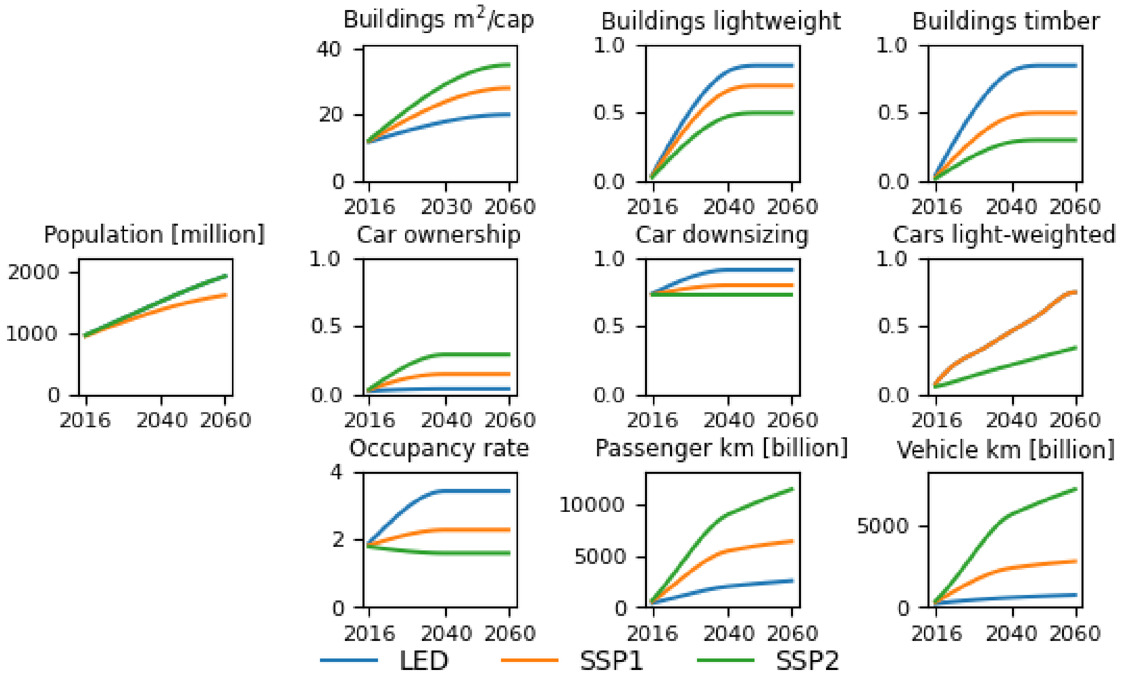

Supplementary Figure 4.17: Drivers and in-use stock parameters, R5.2SSA_Other.

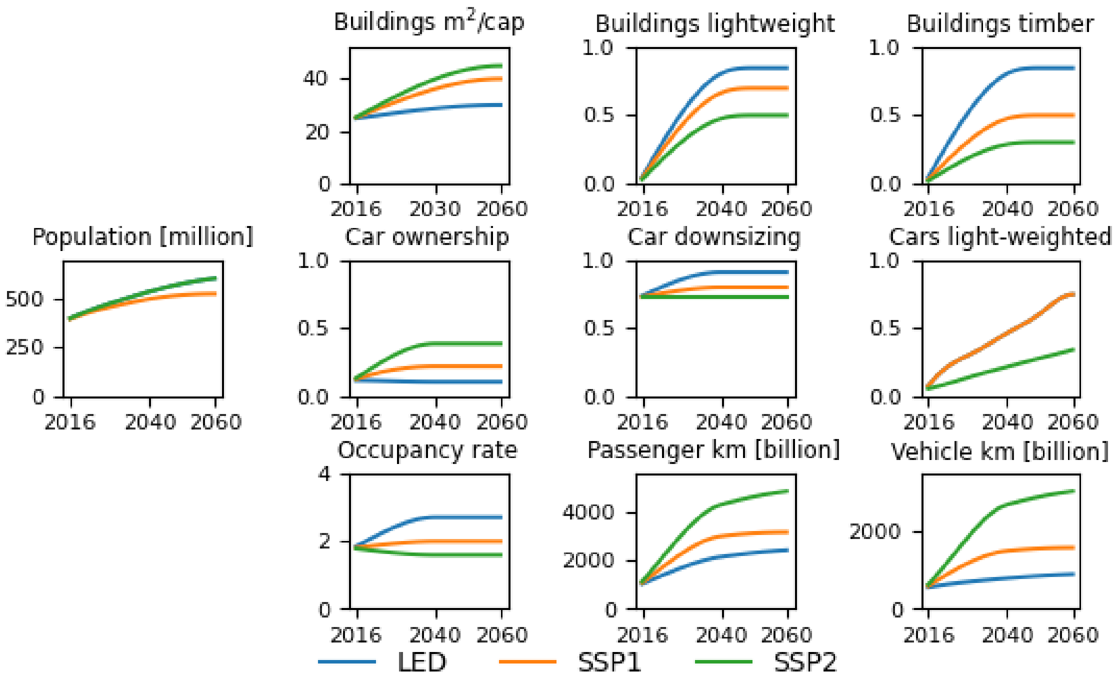

Supplementary Figure 4.18: Drivers and in-use stock parameters, R5.2MNF_Other.

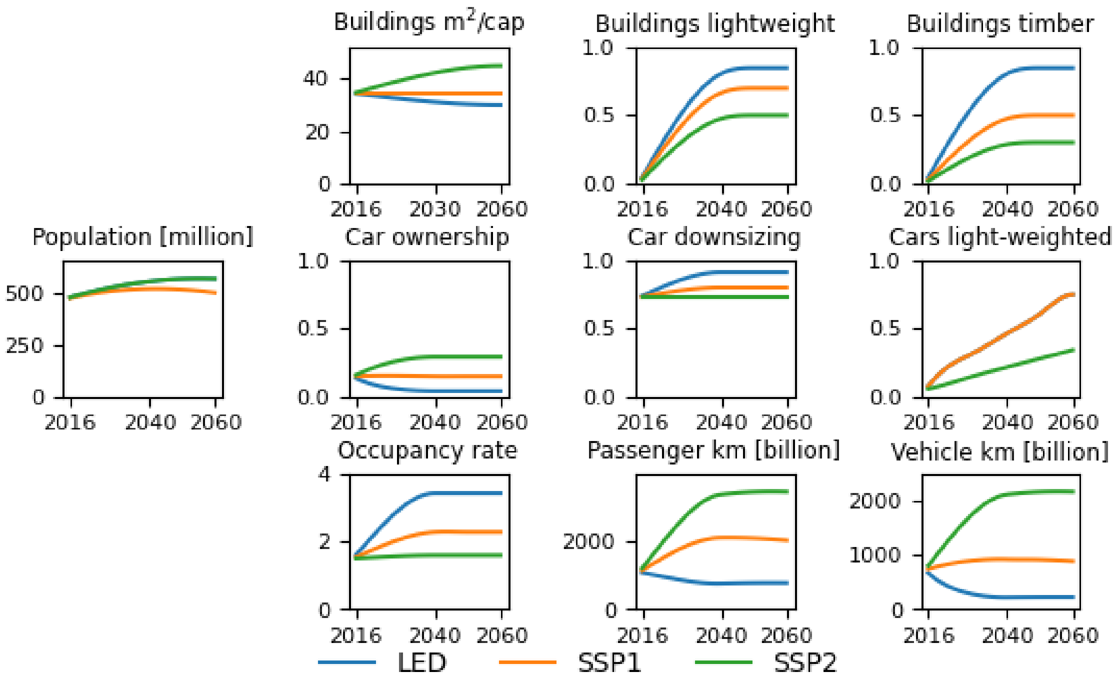

Supplementary Figure 4.19: Drivers and in-use stock parameters, R5.2LAM_Other.

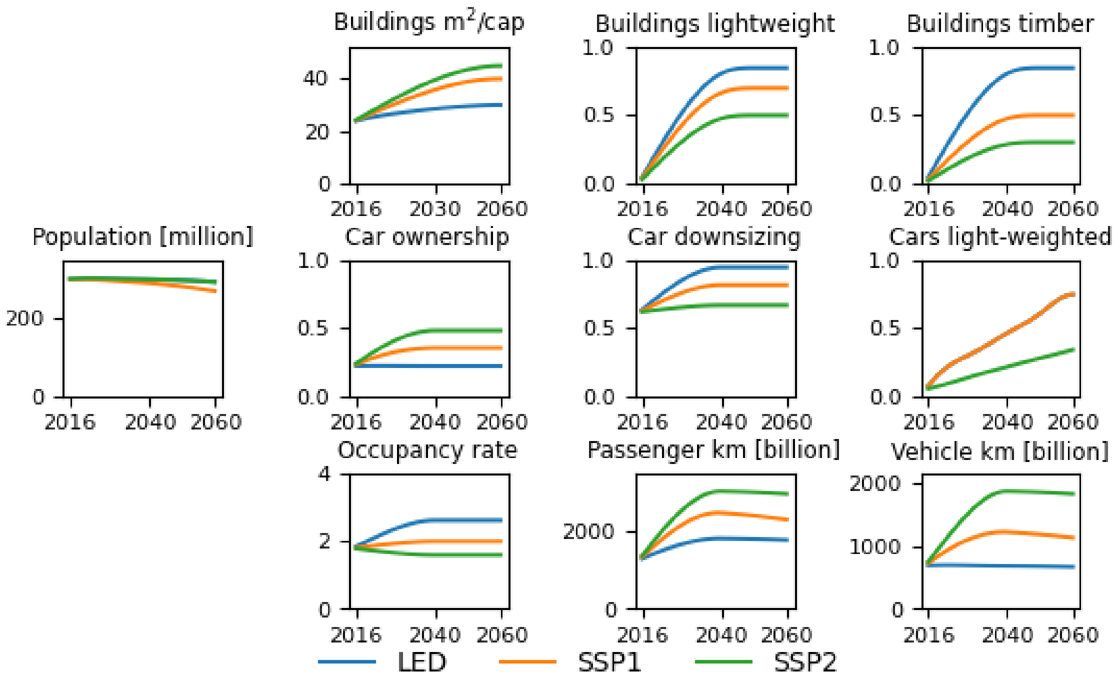

Supplementary Figure 4.20: Drivers and in-use stock parameters, R5.2REF_Other.

Main stock and service parameters, passenger vehicles, by country/region. Supplementary Figures 5.1 – 5.20 below show the temporal development of the passenger vehicle fleet (total number of vehicles by drive technology, left y axis) and the annual change of the total fleet size (stock change, right y axis).

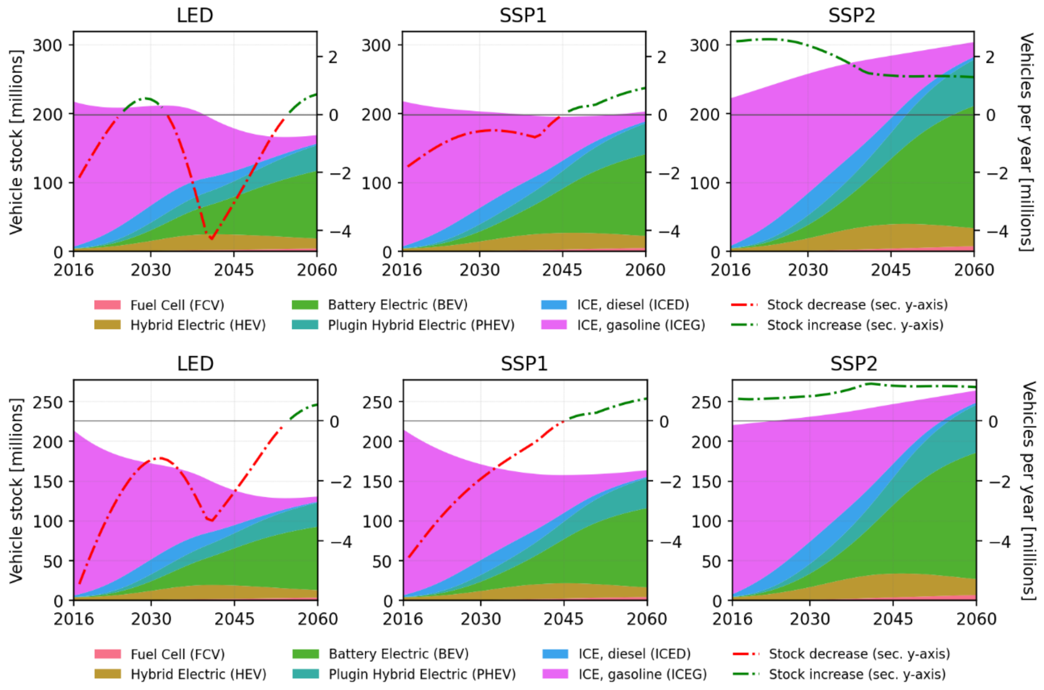

Supplementary Figure 5.1: Drivers and stock parameters, passenger vehicles, USA. Top row: no material efficiency strategies (MES) included, bottom row: full MES spectrum. Results are shown for the 2°C-compatible climate polity scenario.

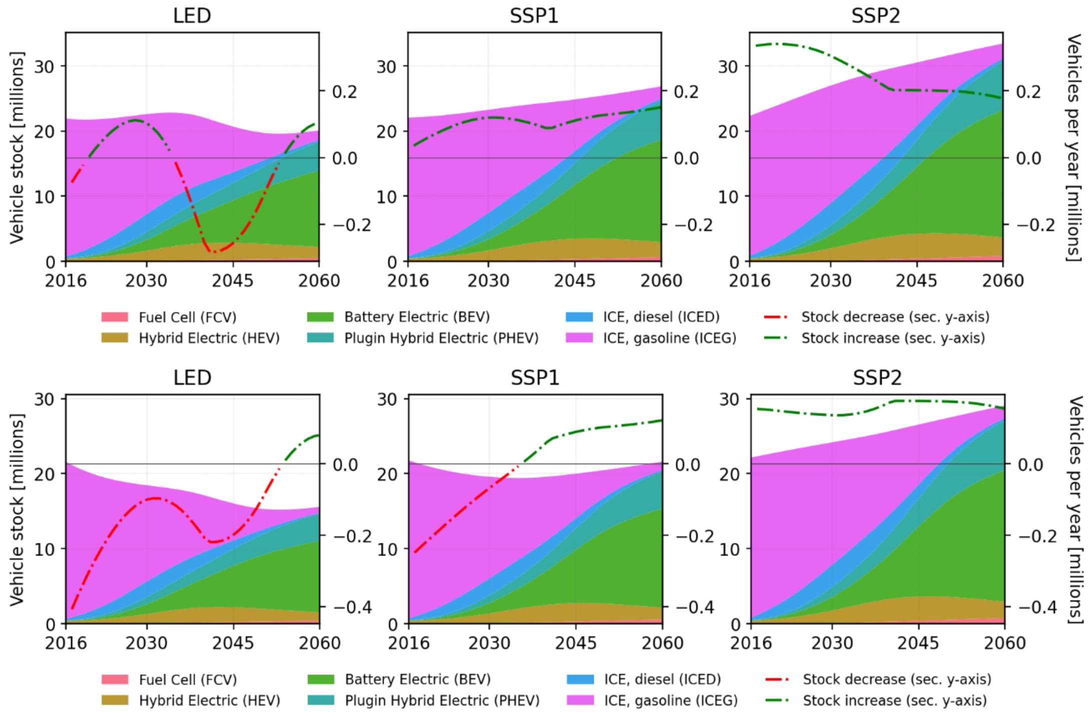

Supplementary Figure 5.2: Drivers and stock parameters, passenger vehicles, Canada. Top row: no material efficiency strategies (MES) included, bottom row: full MES spectrum. Results are shown for the 2°C-compatible climate polity scenario.

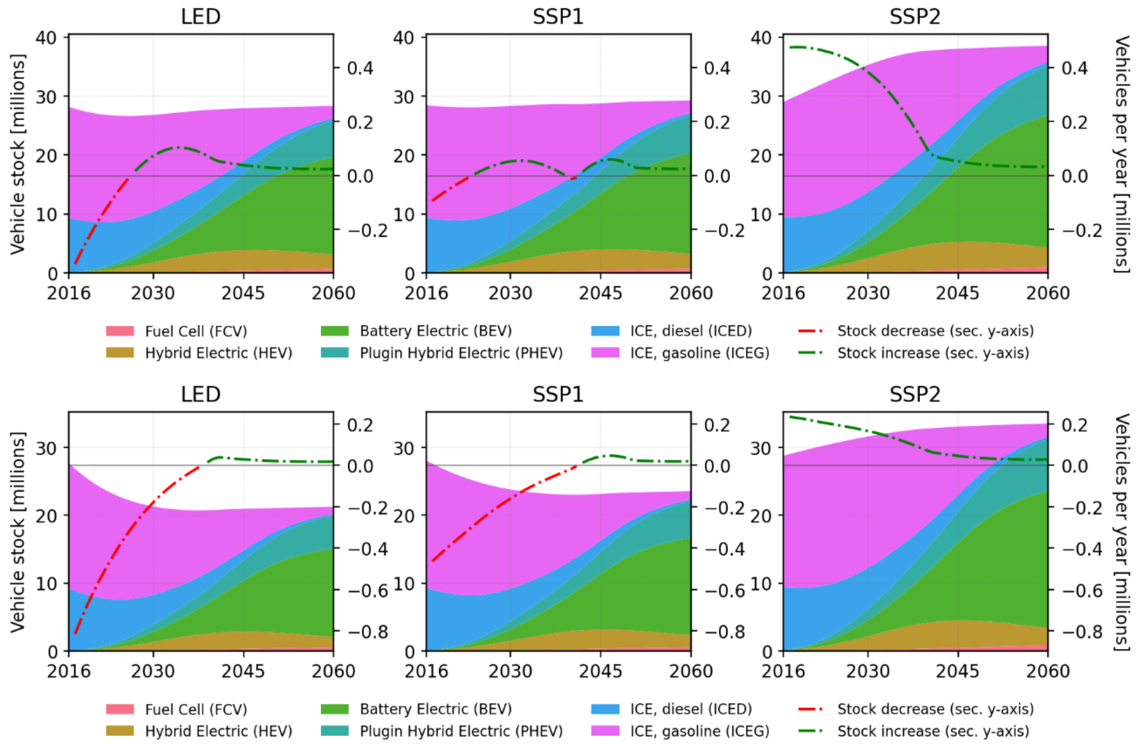

Supplementary Figure 5.3: Drivers and stock parameters, passenger vehicles, France. Top row: no material efficiency strategies (MES) included, bottom row: full MES spectrum. Results are shown for the 2°C-compatible climate polity scenario.

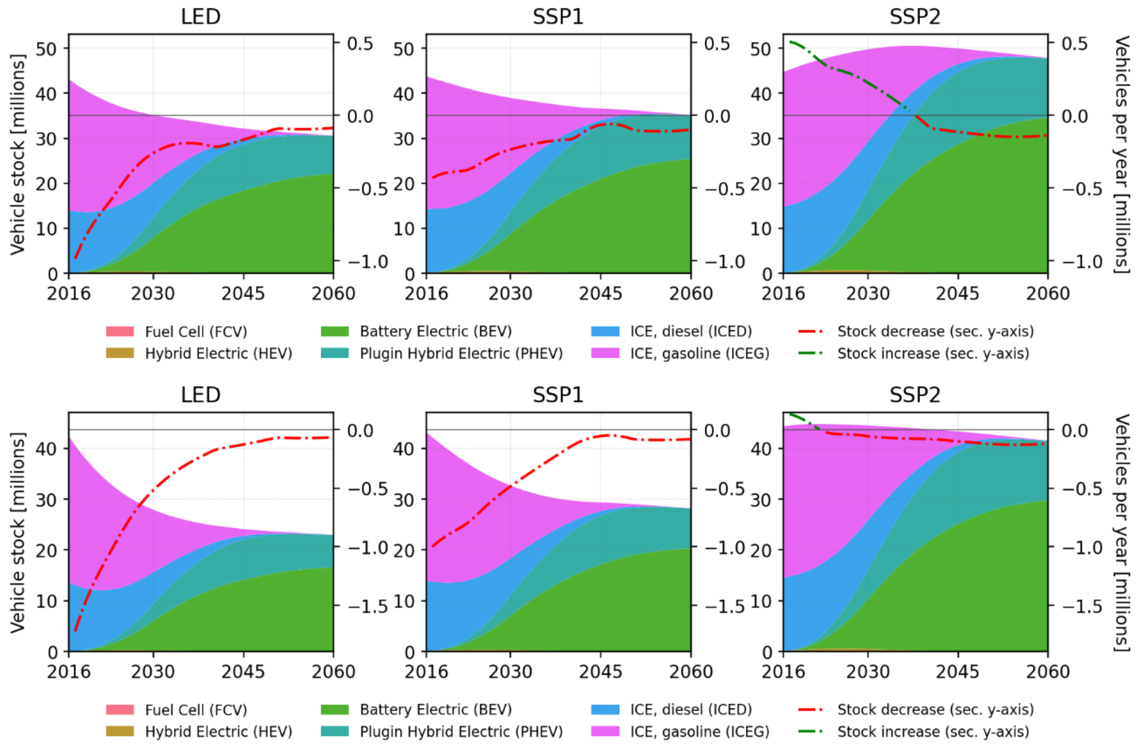

Supplementary Figure 5.4: Drivers and stock parameters, passenger vehicles, Germany. Top row: no material efficiency strategies (MES) included, bottom row: full MES spectrum. Results are shown for the 2°C-compatible climate polity scenario.

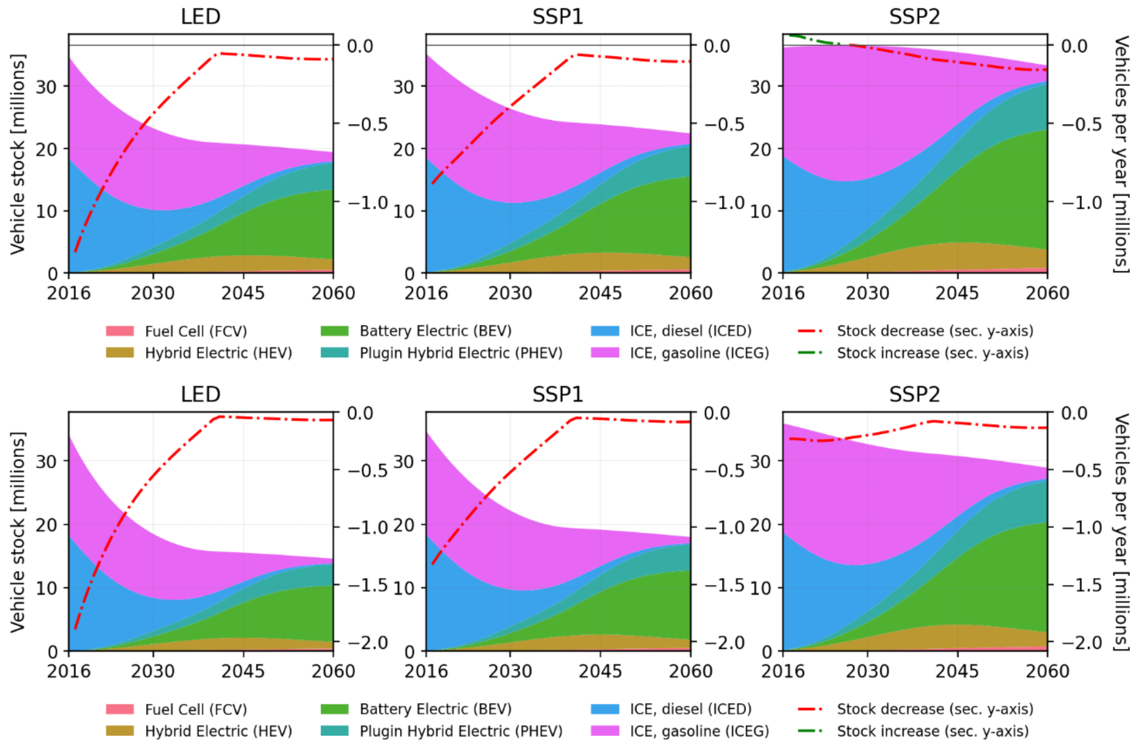

Supplementary Figure 5.5: Drivers and stock parameters, passenger vehicles, Italy. Top row: no material efficiency strategies (MES) included, bottom row: full MES spectrum. Results are shown for the 2°C-compatible climate polity scenario.

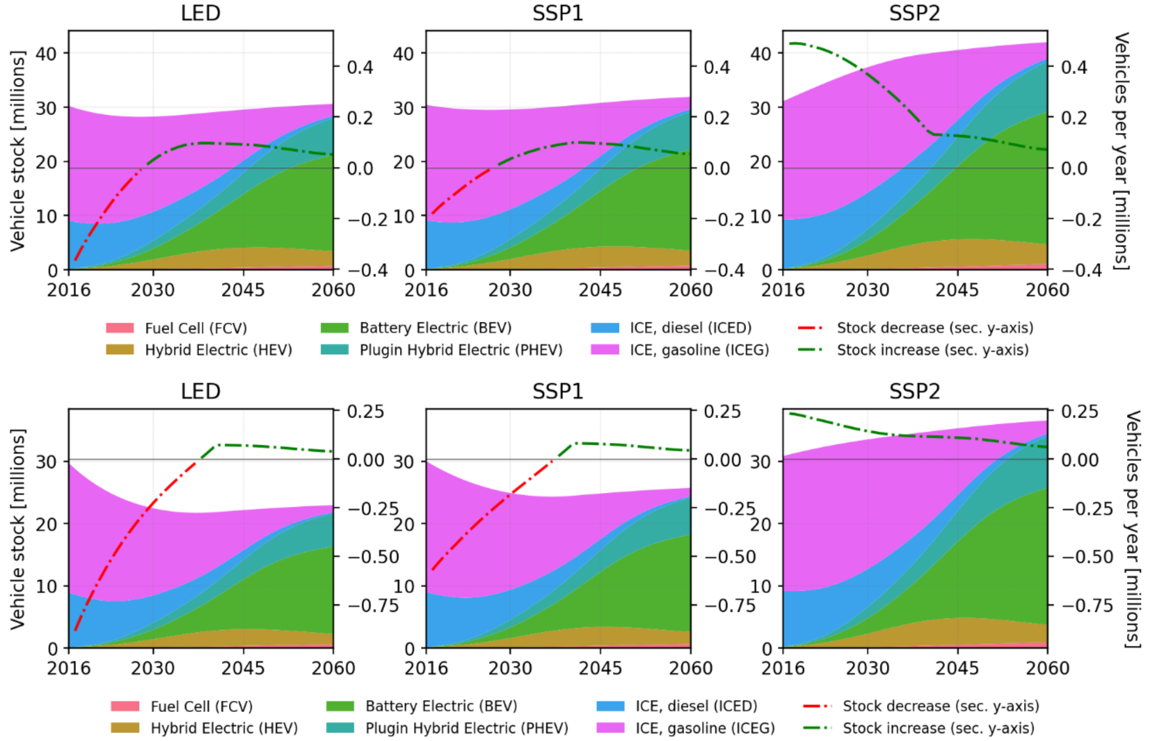

Supplementary Figure 5.6: Drivers and stock parameters, passenger vehicles, UK. Top row: no material efficiency strategies (MES) included, bottom row: full MES spectrum. Results are shown for the 2°C-compatible climate polity scenario.

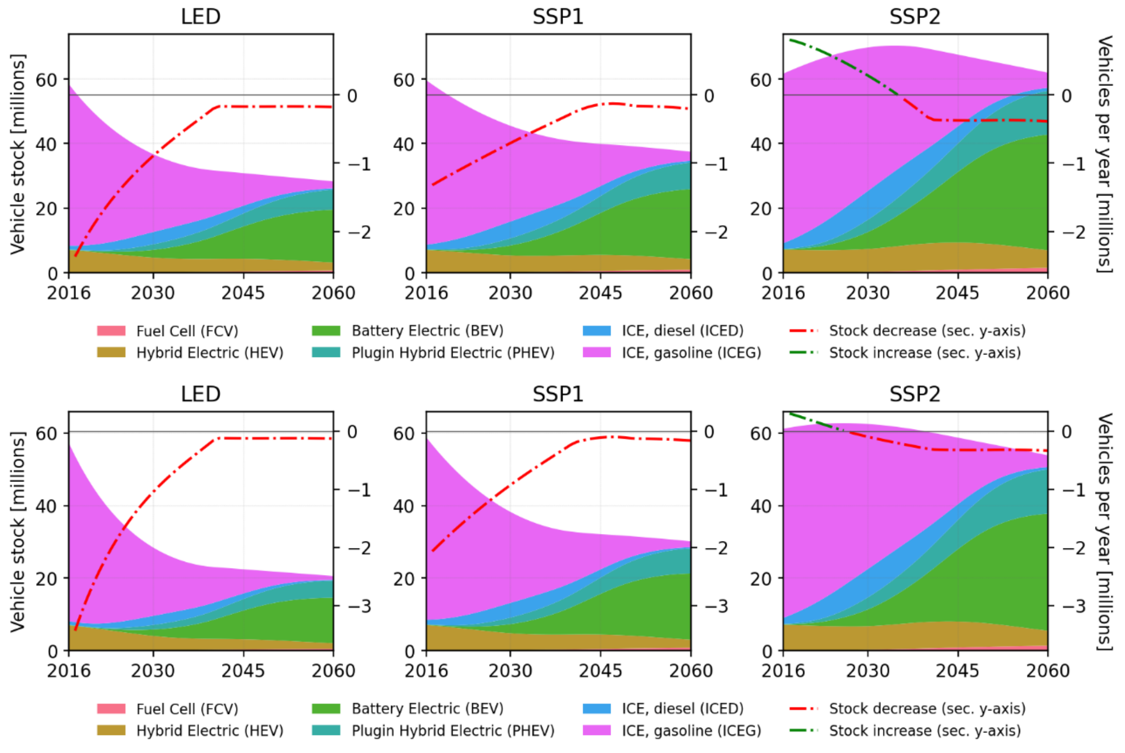

Supplementary Figure 5.7: Drivers and stock parameters, passenger vehicles, Japan. Top row: no material efficiency strategies (MES) included, bottom row: full MES spectrum. Results are shown for the 2°C-compatible climate polity scenario.

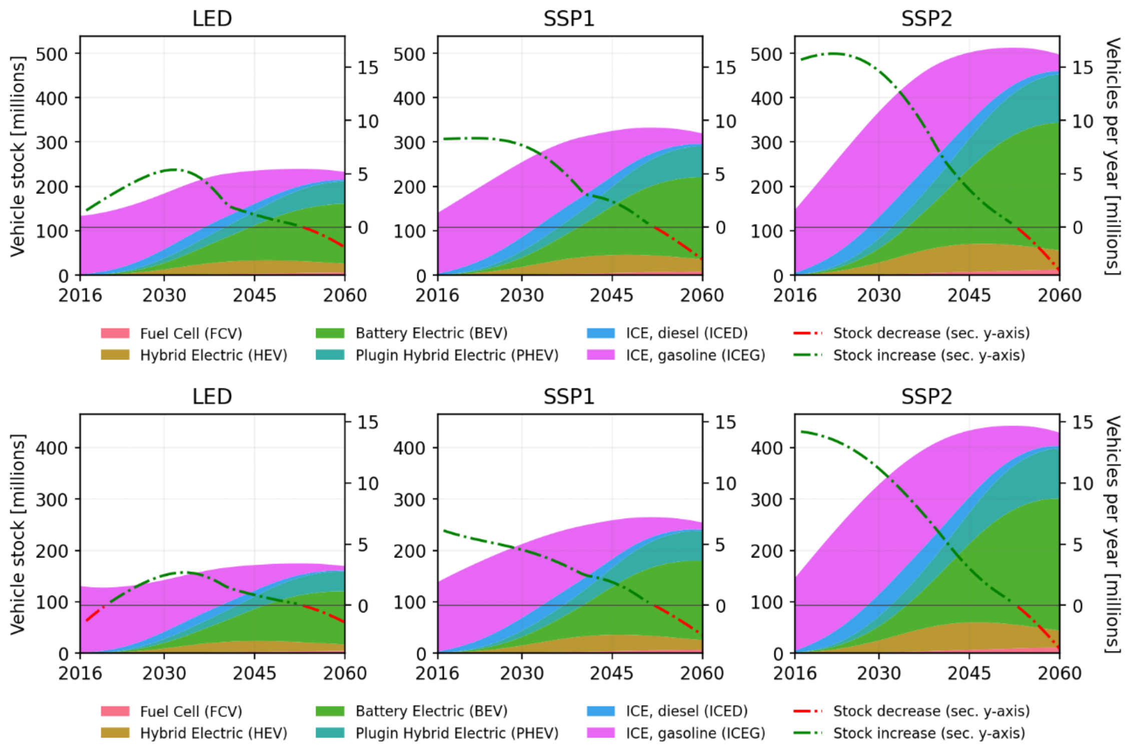

Supplementary Figure 5.8: Drivers and stock parameters, passenger vehicles, China. Top row: no material efficiency strategies (MES) included, bottom row: full MES spectrum. Results are shown for the 2°C-compatible climate polity scenario.

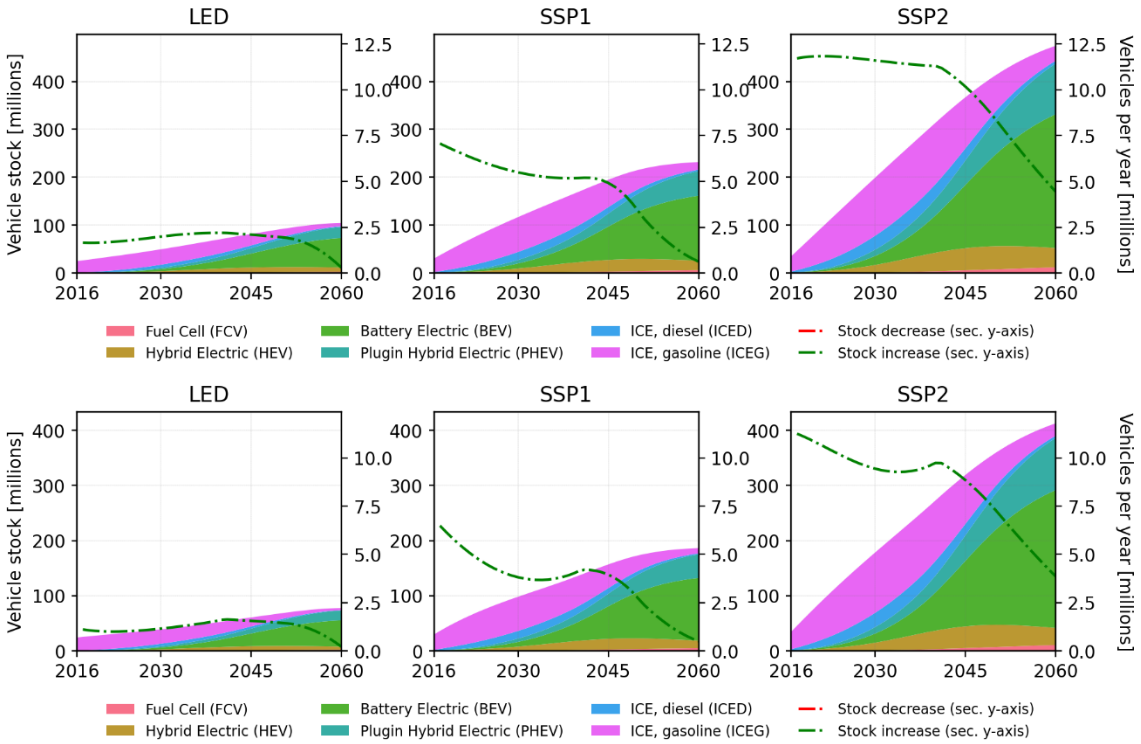

Supplementary Figure 5.9: Drivers and stock parameters, passenger vehicles, India. Top row: no material efficiency strategies (MES) included, bottom row: full MES spectrum. Results are shown for the 2°C-compatible climate polity scenario.

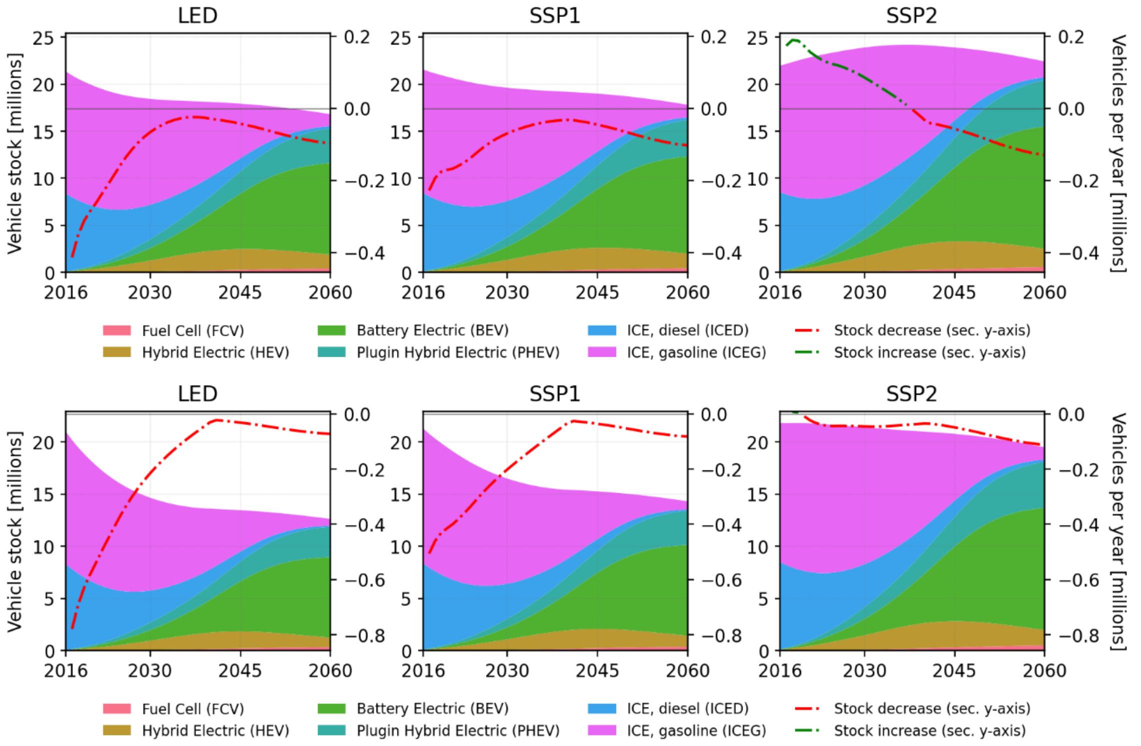

Supplementary Figure 5.10: Drivers and stock parameters, passenger vehicles, Spain. Top row: no material efficiency strategies (MES) included, bottom row: full MES spectrum. Results are shown for the 2°C-compatible climate polity scenario.

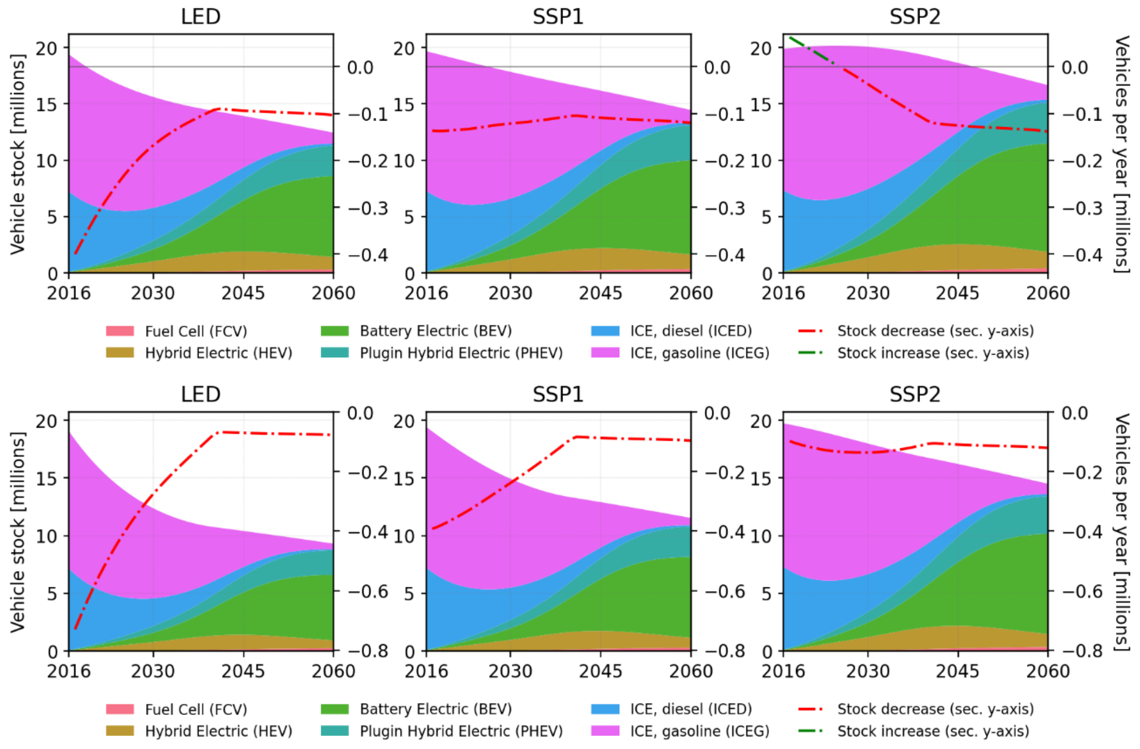

Supplementary Figure 5.11: Drivers and stock parameters, passenger vehicles, Poland. Top row: no material efficiency strategies (MES) included, bottom row: full MES spectrum. Results are shown for the 2°C-compatible climate polity scenario.

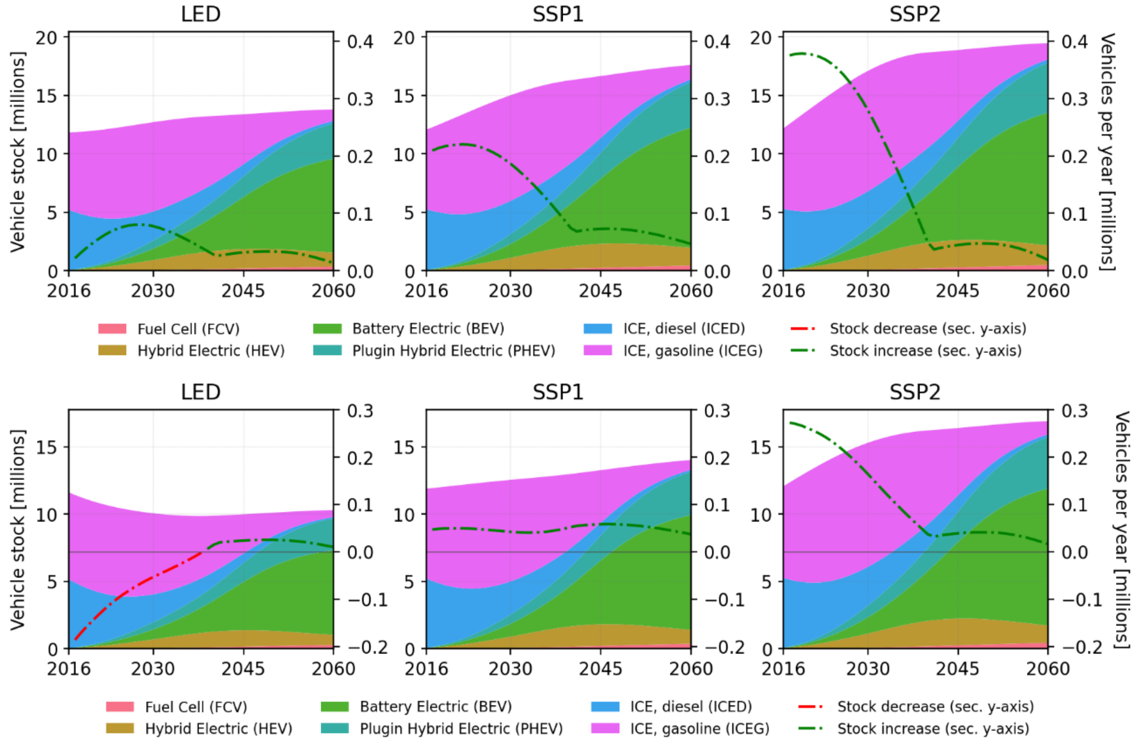

Supplementary Figure 5.12: Drivers and stock parameters, passenger vehicles, Oth_R32EU12-H. Top row: no material efficiency strategies (MES) included, bottom row: full MES spectrum. Results are shown for the 2°C-compatible climate polity scenario.

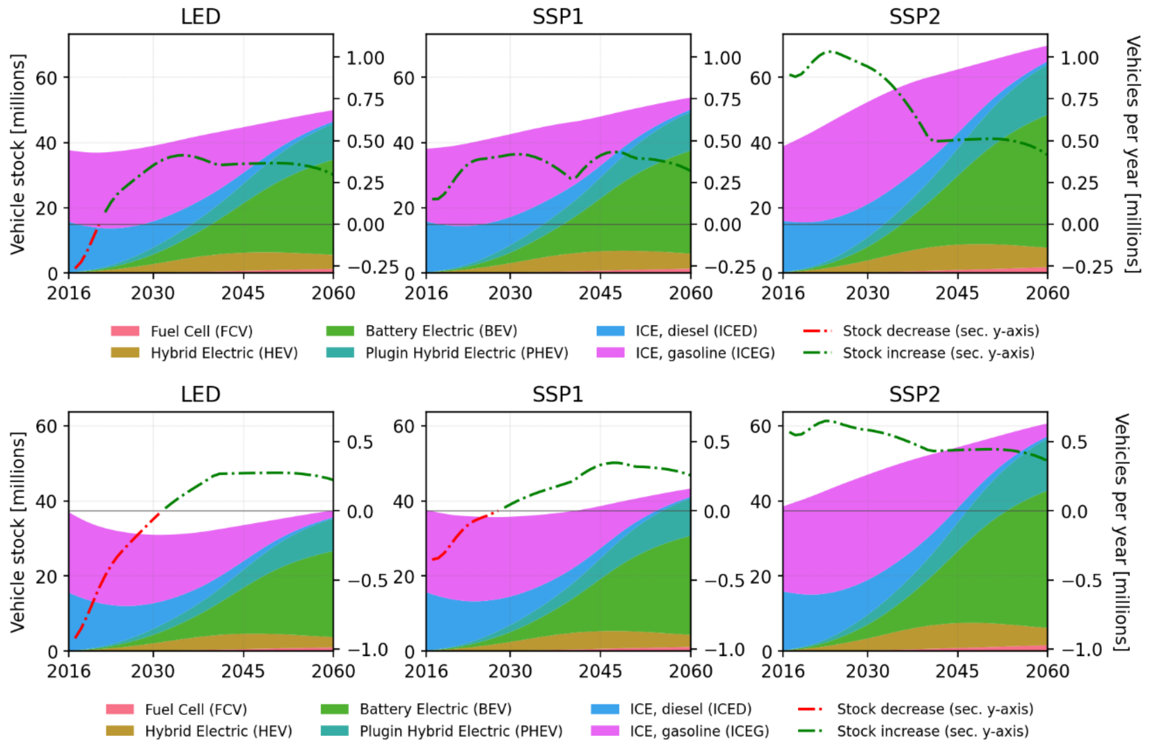

Supplementary Figure 5.13: Drivers and stock parameters, passenger vehicles, Oth_R32EU15. Top row: no material efficiency strategies (MES) included, bottom row: full MES spectrum. Results are shown for the 2°C-compatible climate polity scenario.

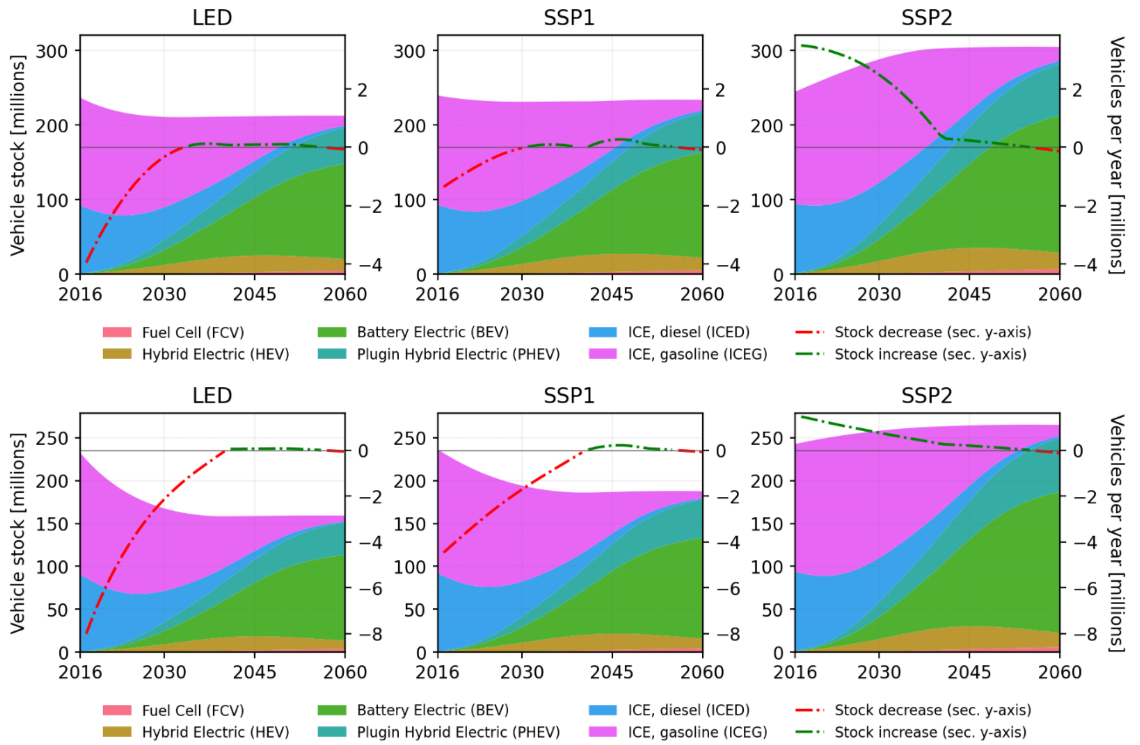

Supplementary Figure 5.14: Drivers and stock parameters, passenger vehicles, EU28. Top row: no material efficiency strategies (MES) included, bottom row: full MES spectrum. Results are shown for the 2°C-compatible climate polity scenario.

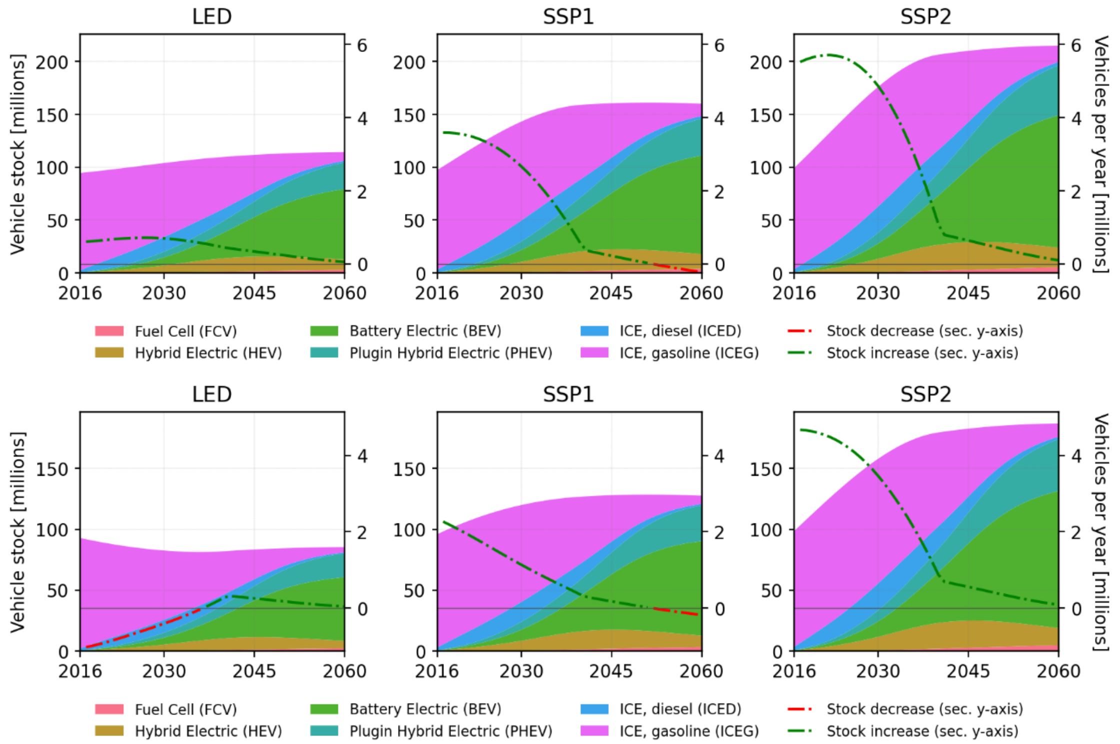

Supplementary Figure 5.15: Drivers and stock parameters, passenger vehicles, R5.2OECD_Other. Top row: no material efficiency strategies (MES) included, bottom row: full MES spectrum. Results are shown for the 2°C-compatible climate polity scenario.

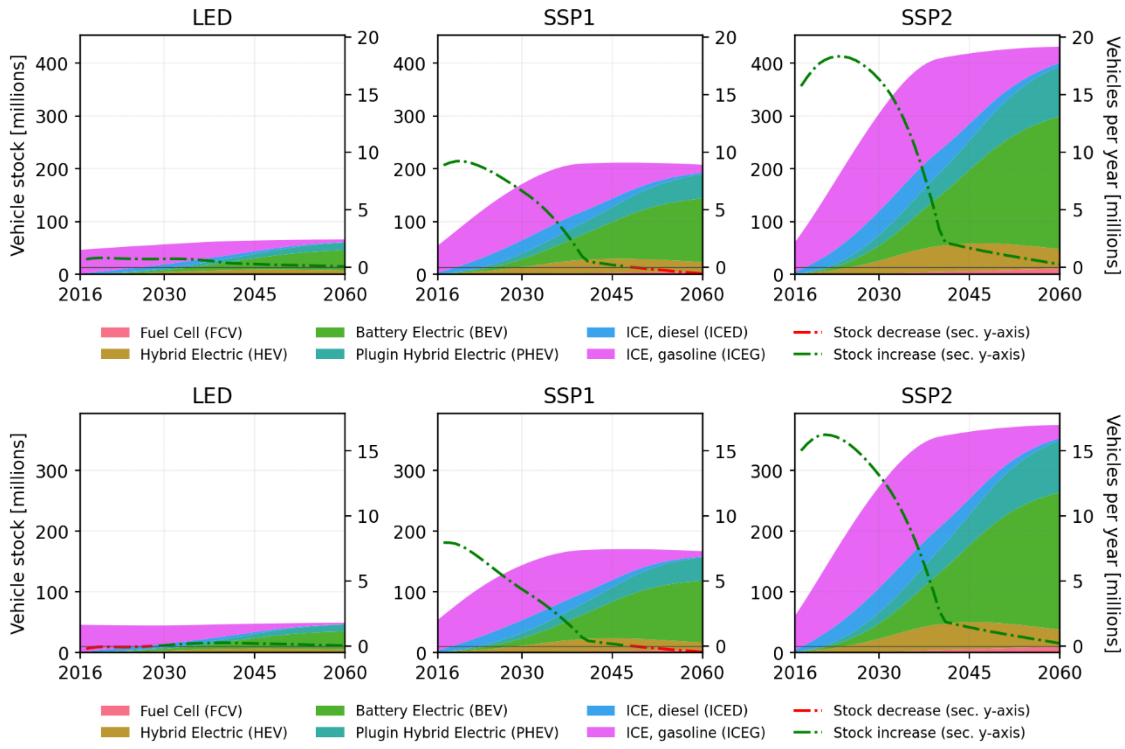

Supplementary Figure 5.16: Drivers and stock parameters, passenger vehicles, R5.2Asia_Other. Top row: no material efficiency strategies (MES) included, bottom row: full MES spectrum. Results are shown for the 2°C-compatible climate polity scenario.

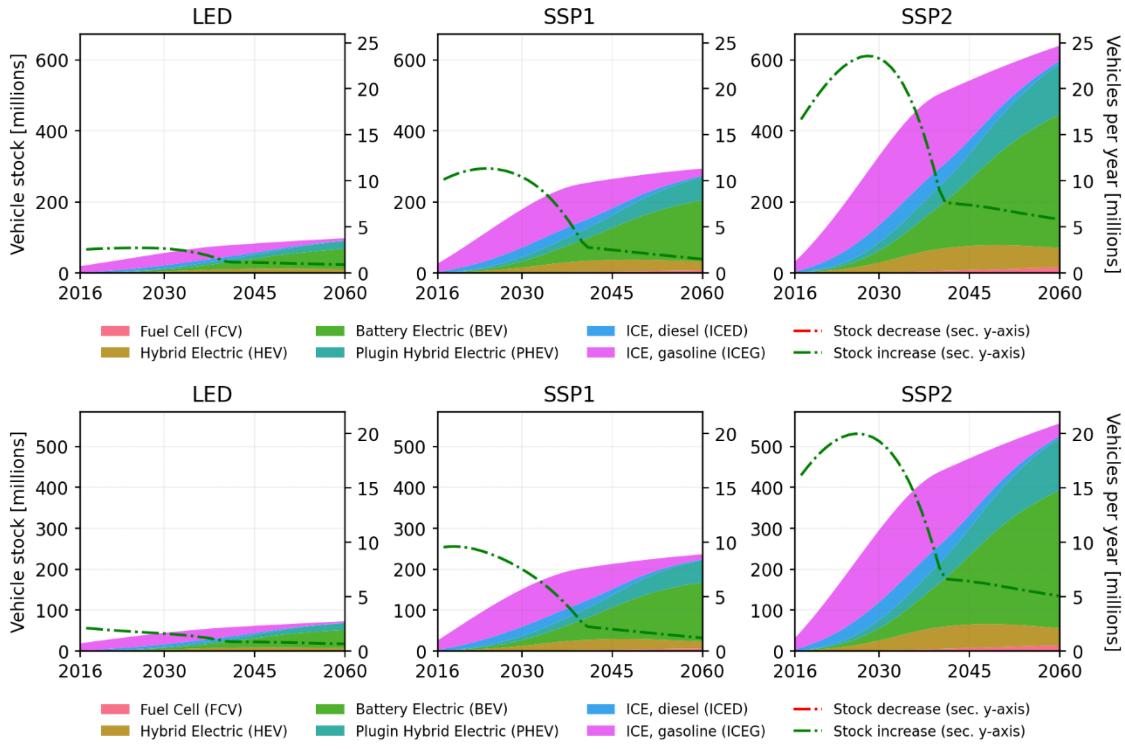

Supplementary Figure 5.17: Drivers and stock parameters, passenger vehicles, R5.2SSA_Other. Top row: no material efficiency strategies (MES) included, bottom row: full MES spectrum. Results are shown for the 2°C-compatible climate polity scenario.

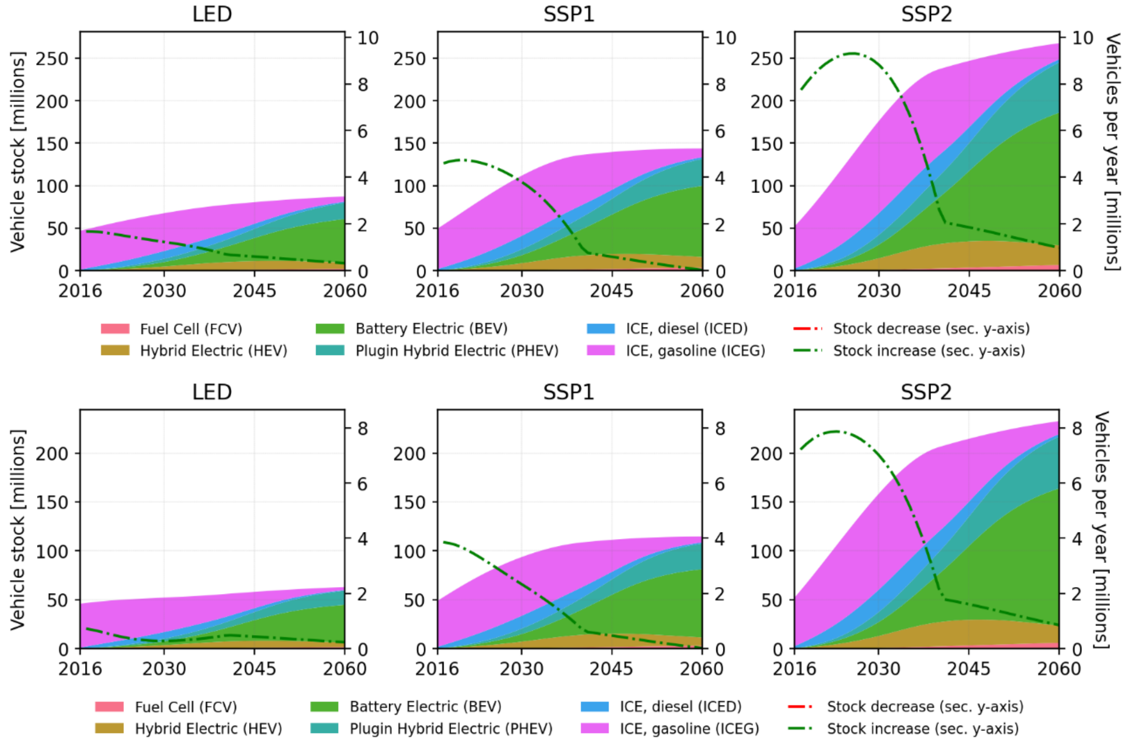

Supplementary Figure 5.18: Drivers and stock parameters, passenger vehicles, R5.2MNF_Other. Top row: no material efficiency strategies (MES) included, bottom row: full MES spectrum. Results are shown for the 2°C-compatible climate polity scenario.

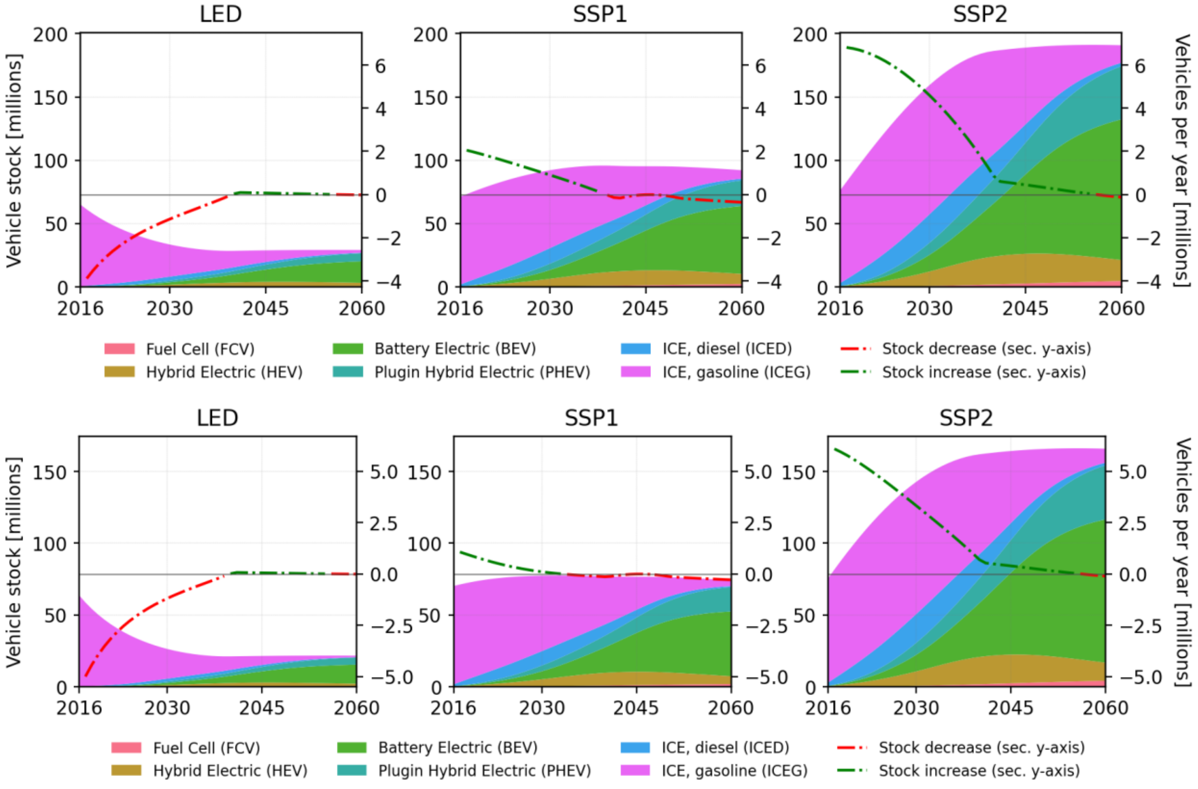

Supplementary Figure 5.19: Drivers and stock parameters, passenger vehicles, R5.2LAM_Other. Top row: no material efficiency strategies (MES) included, bottom row: full MES spectrum. Results are shown for the 2°C-compatible climate polity scenario.

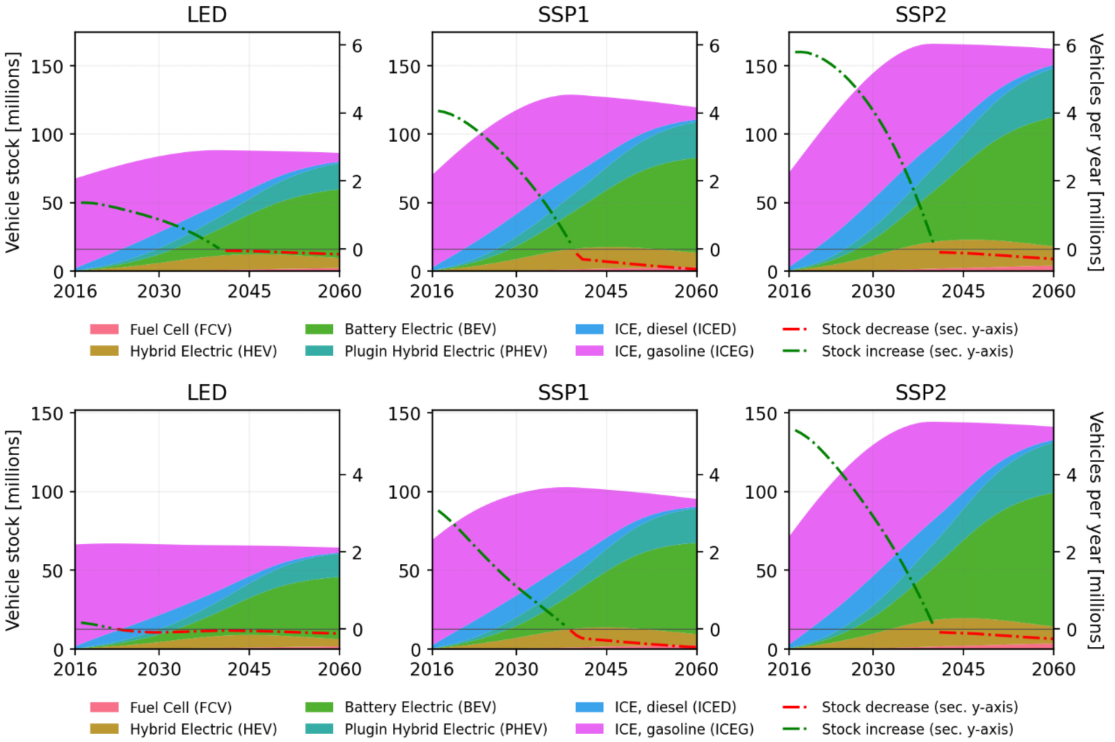

Supplementary Figure 5.20: Drivers and stock parameters, passenger vehicles, R5.2REF_Other. Top row: no material efficiency strategies (MES) included, bottom row: full MES spectrum. Results are shown for the 2°C-compatible climate polity scenario.

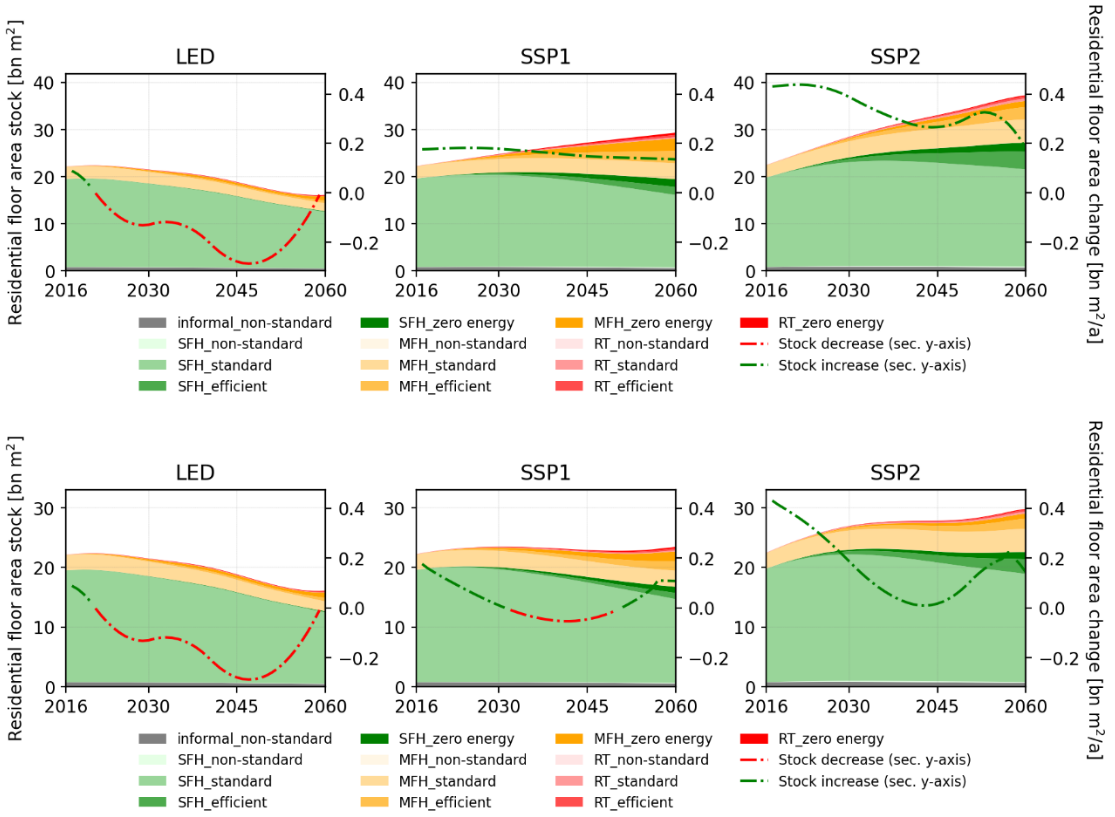

Supplementary Figure 6.1: Drivers and stock parameters, residential buildings, USA. Top row: no material efficiency strategies (MES) included, bottom row: full MES spectrum. Results are shown for the 2°C-compatible climate polity scenario.

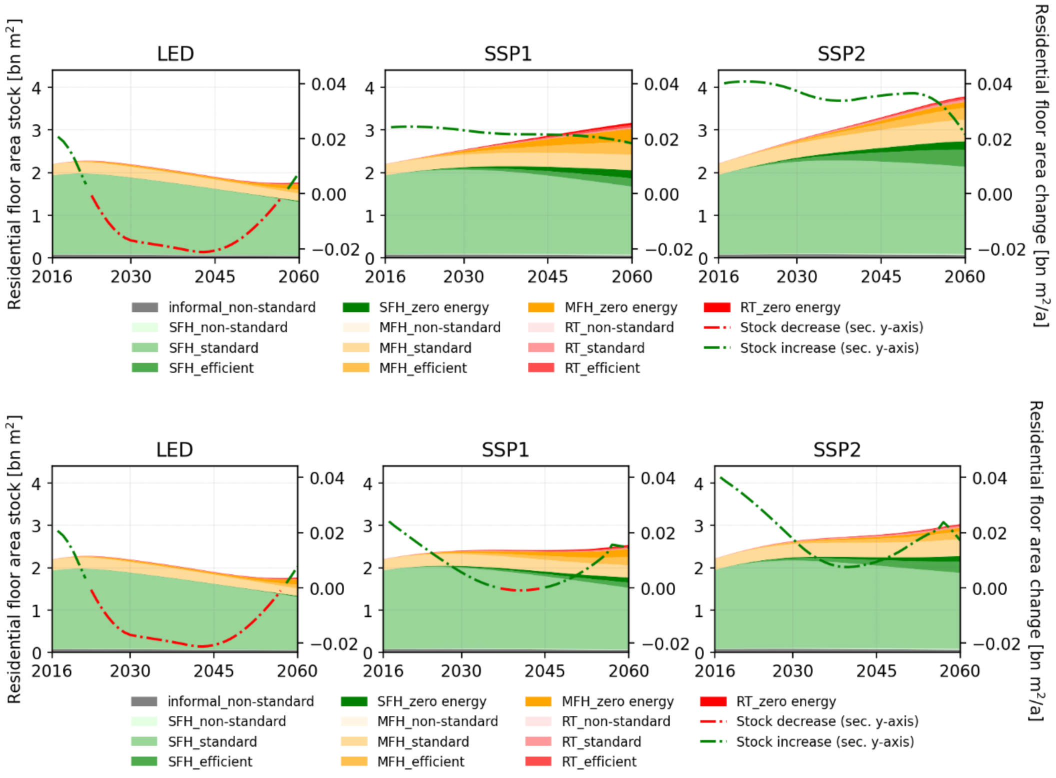

Supplementary Figure 6.2: Drivers and stock parameters, residential buildings, Canada. Top row: no material efficiency strategies (MES) included, bottom row: full MES spectrum. Results are shown for the 2°C-compatible climate polity scenario.

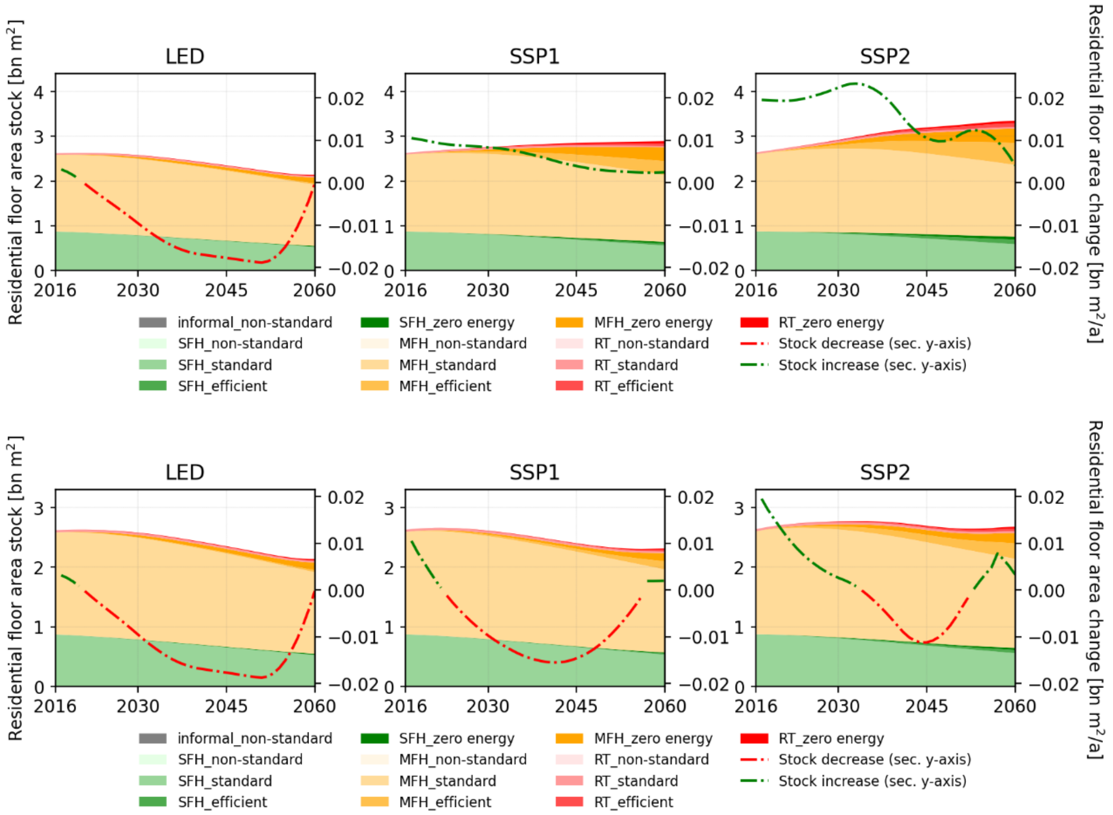

Supplementary Figure 6.3: Drivers and stock parameters, residential buildings, France. Top row: no material efficiency strategies (MES) included, bottom row: full MES spectrum. Results are shown for the 2°C-compatible climate polity scenario.

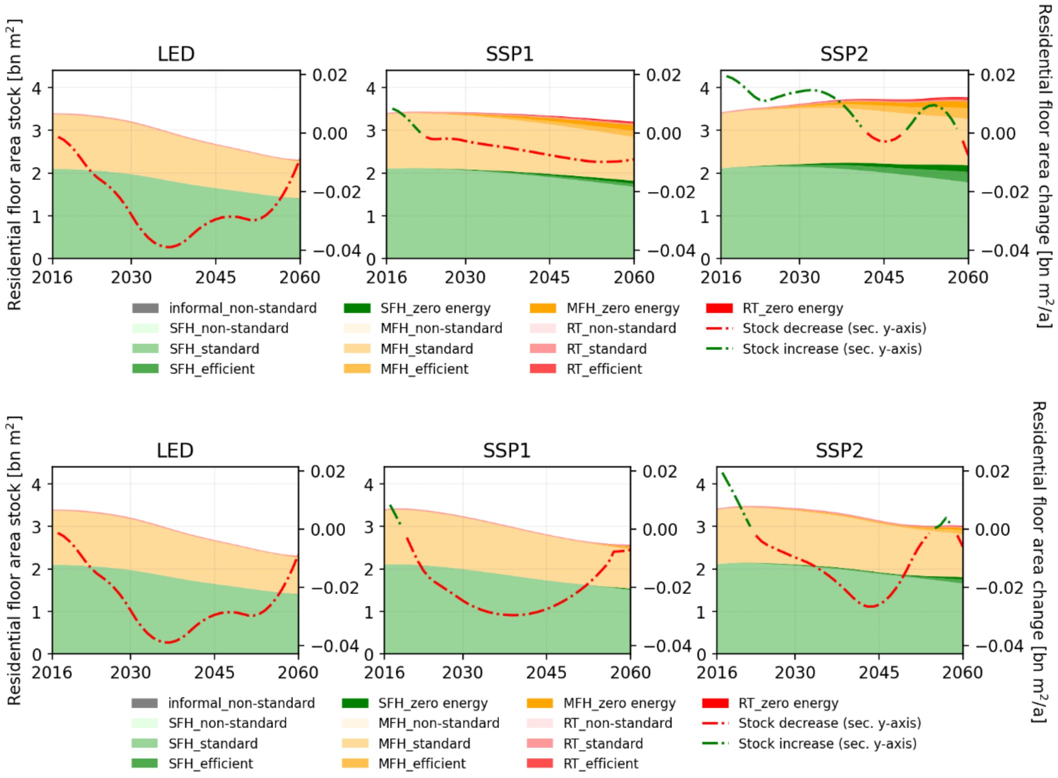

Supplementary Figure 6.4: Drivers and stock parameters, residential buildings, Germany. Top row: no material efficiency strategies (MES) included, bottom row: full MES spectrum. Results are shown for the 2°C-compatible climate polity scenario.

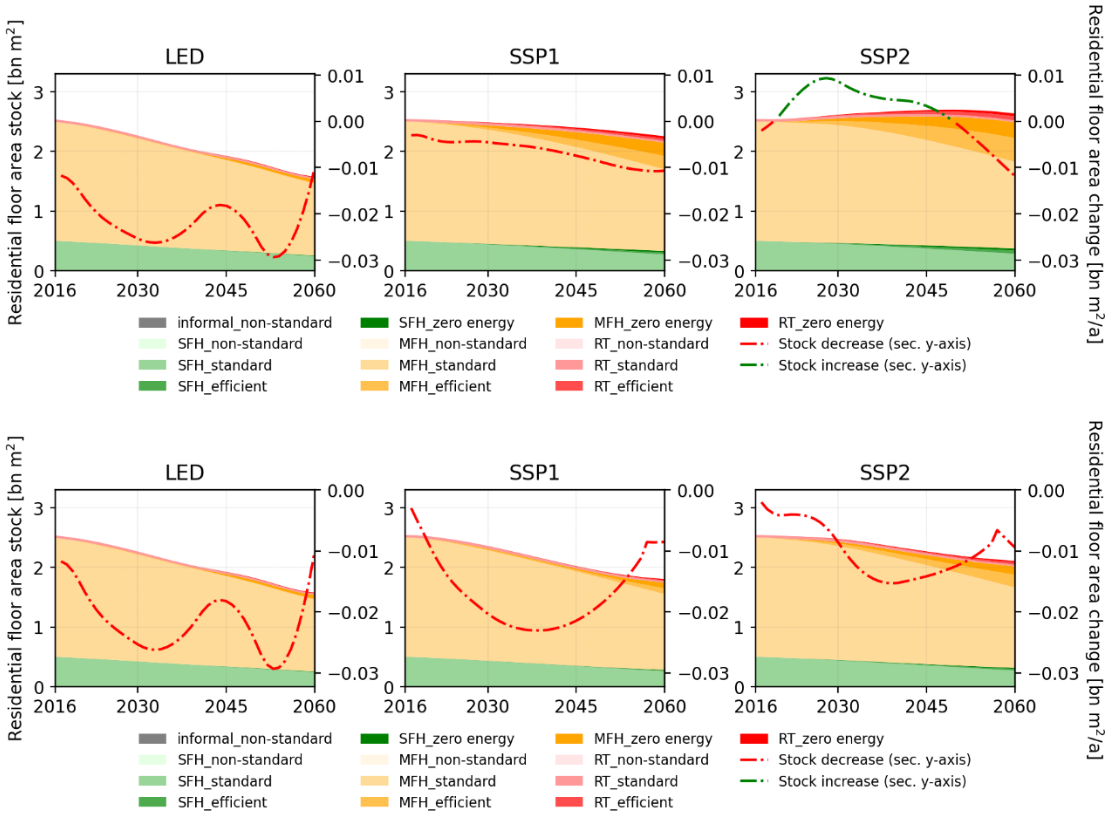

Supplementary Figure 6.5: Drivers and stock parameters, residential buildings, Italy. Top row: no material efficiency strategies (MES) included, bottom row: full MES spectrum. Results are shown for the 2°C-compatible climate polity scenario.

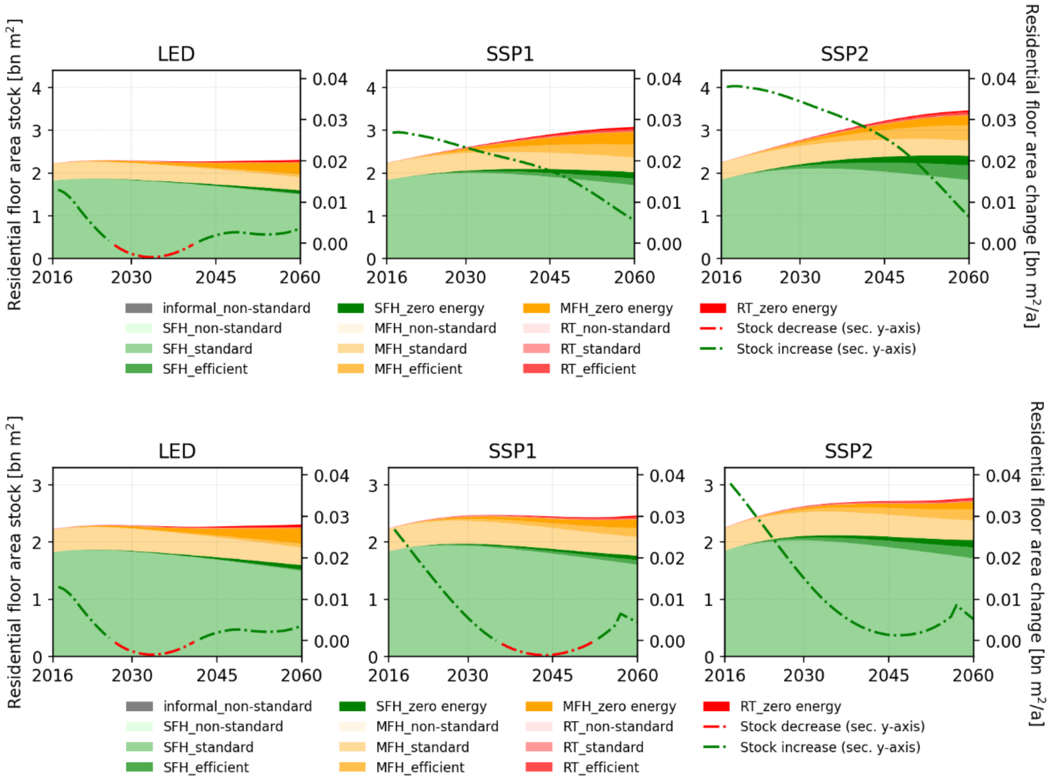

Supplementary Figure 6.6: Drivers and stock parameters, residential buildings, UK. Top row: no material efficiency strategies (MES) included, bottom row: full MES spectrum. Results are shown for the 2°C-compatible climate polity scenario.

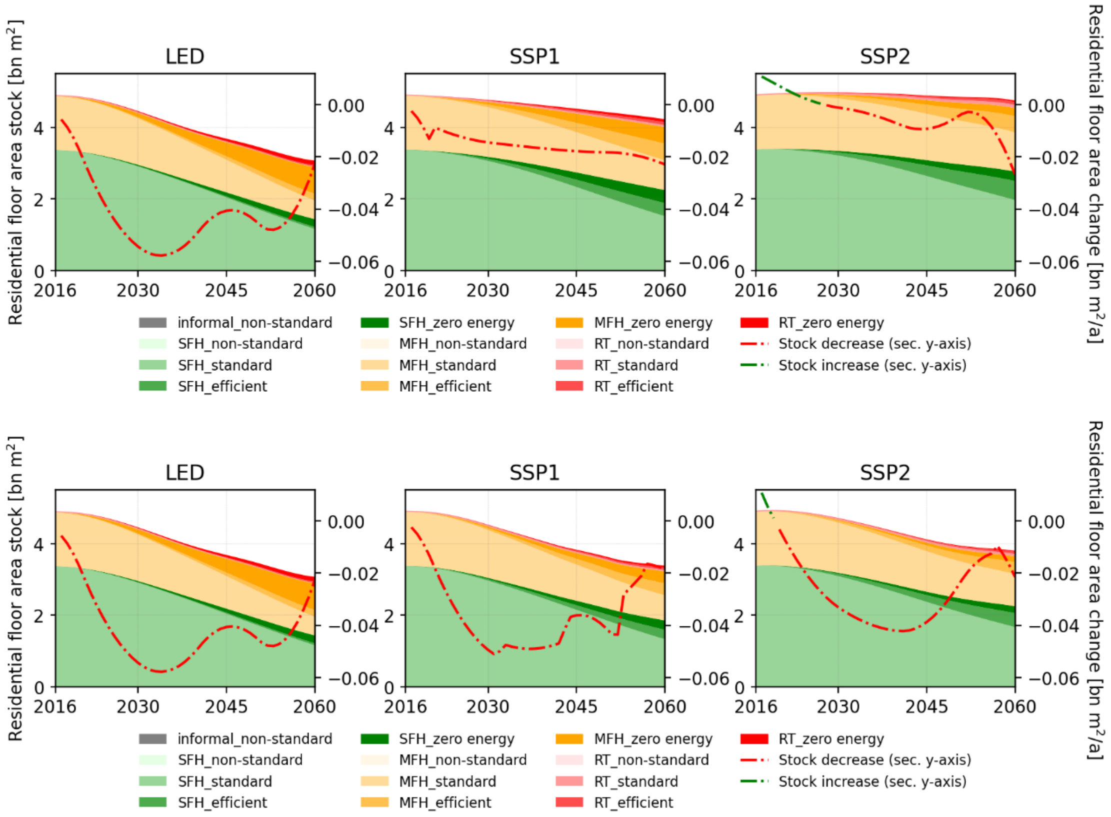

Supplementary Figure 6.7: Drivers and stock parameters, residential buildings, Japan. Top row: no material efficiency strategies (MES) included, bottom row: full MES spectrum. Results are shown for the 2°C-compatible climate polity scenario.

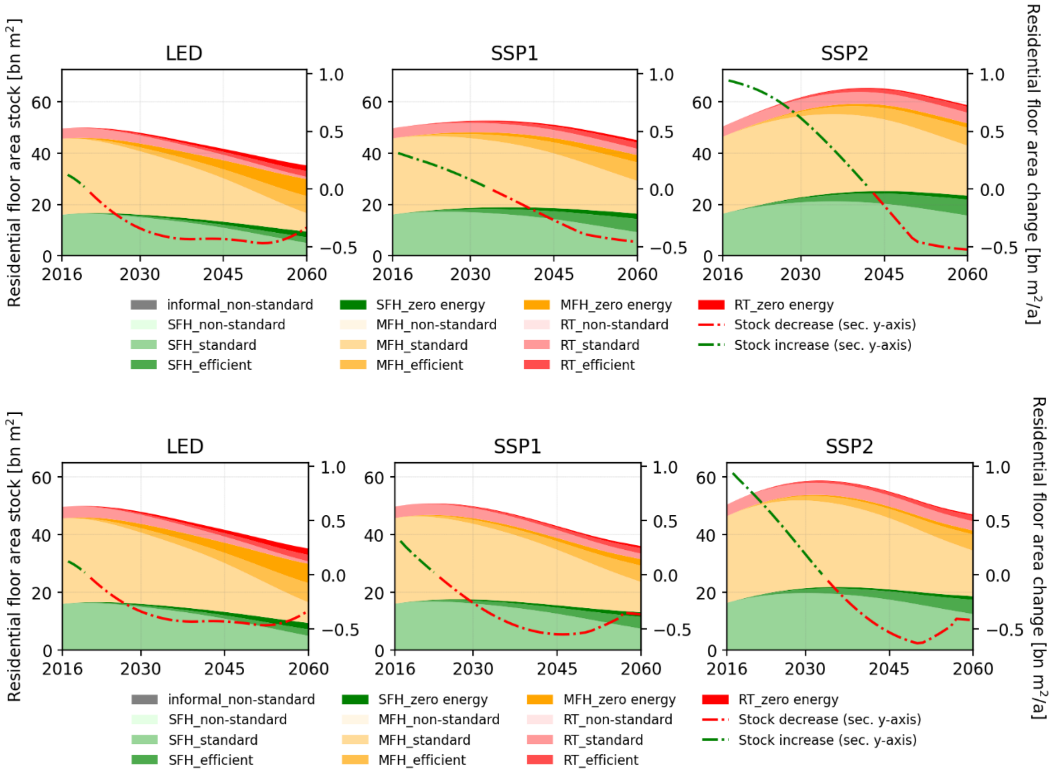

Supplementary Figure 6.8: Drivers and stock parameters, residential buildings, China. Top row: no material efficiency strategies (MES) included, bottom row: full MES spectrum. Results are shown for the 2°C-compatible climate polity scenario.

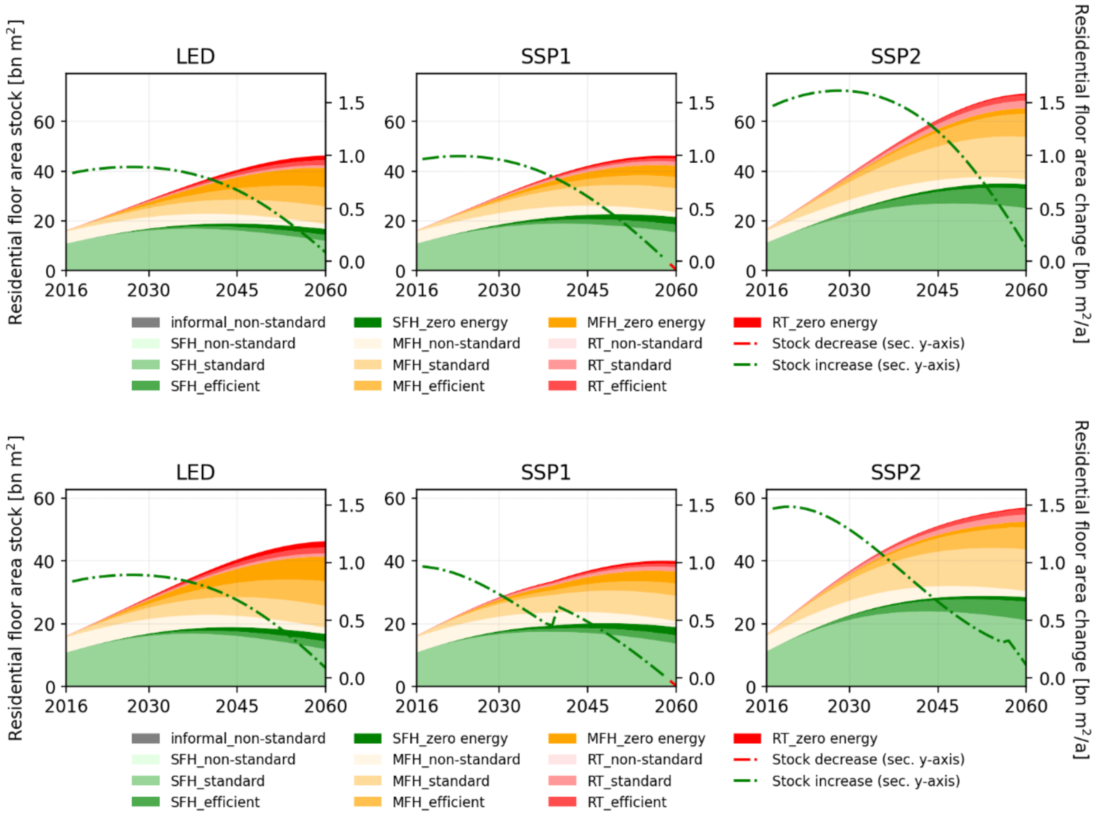

Supplementary Figure 6.9: Drivers and stock parameters, residential buildings, India. Top row: no material efficiency strategies (MES) included, bottom row: full MES spectrum. Results are shown for the 2°C-compatible climate polity scenario.

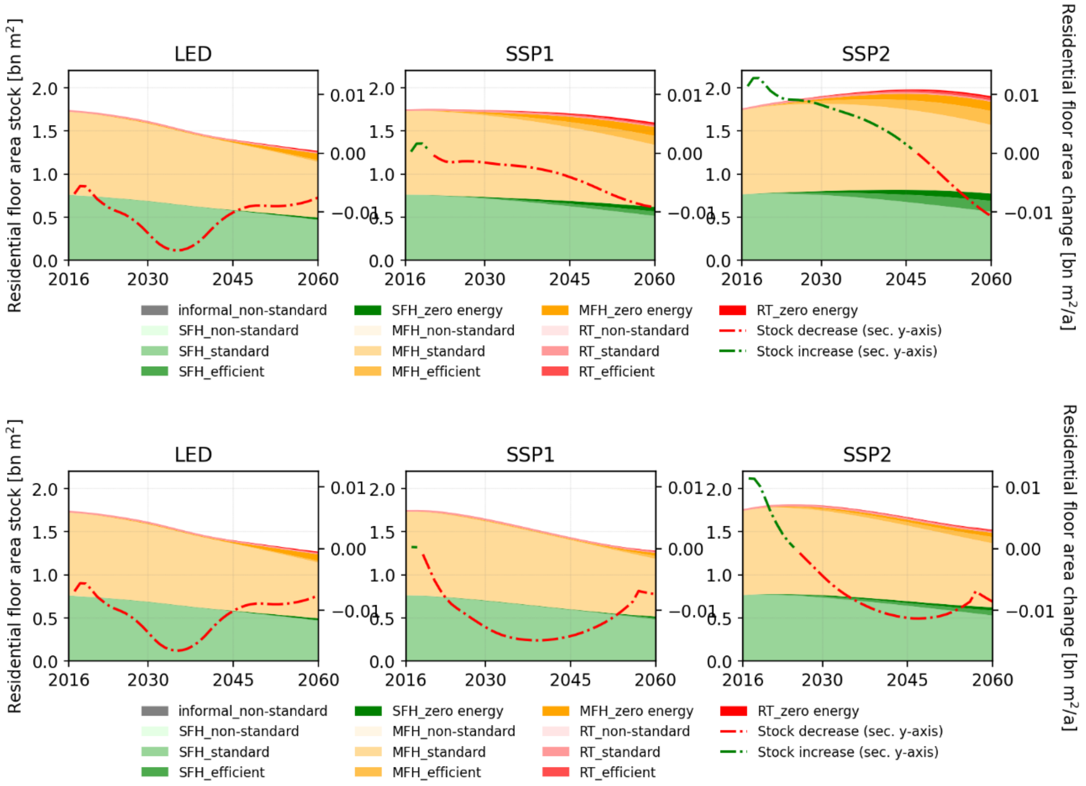

Supplementary Figure 6.10: Drivers and stock parameters, residential buildings, Spain. Top row: no material efficiency strategies (MES) included, bottom row: full MES spectrum. Results are shown for the 2°C-compatible climate polity scenario.

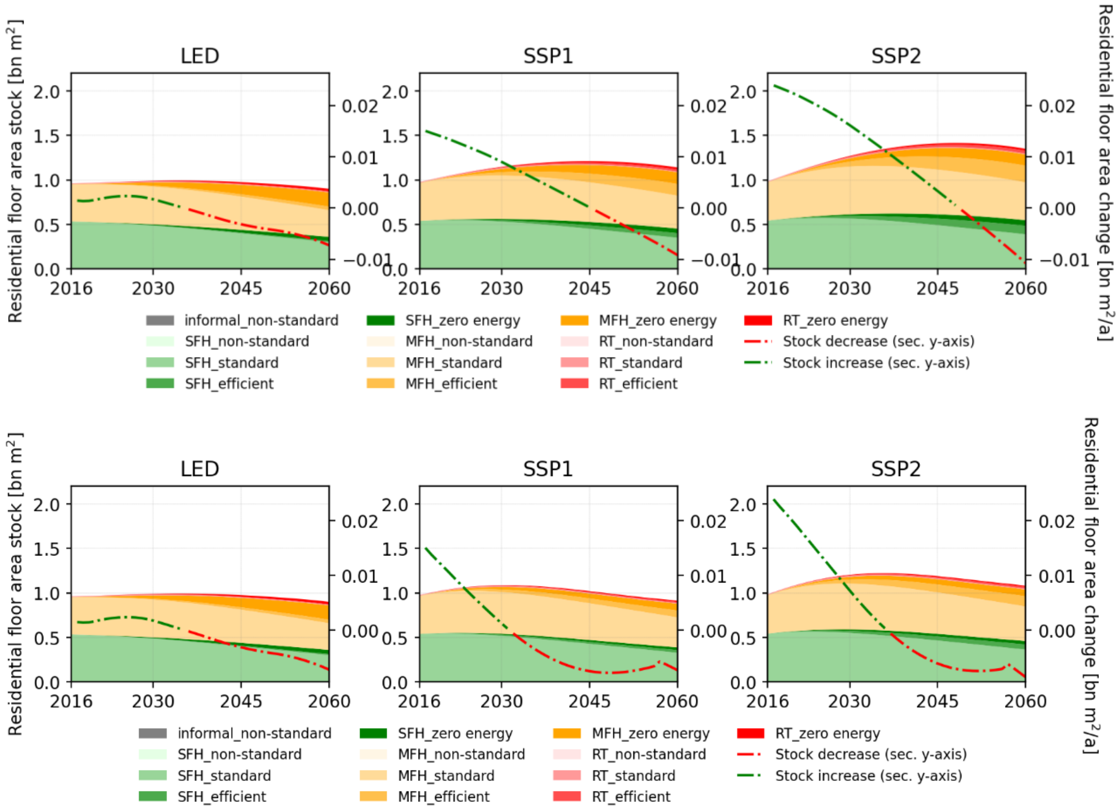

Supplementary Figure 6.11: Drivers and stock parameters, residential buildings, Poland. Top row: no material efficiency strategies (MES) included, bottom row: full MES spectrum. Results are shown for the 2°C-compatible climate polity scenario.

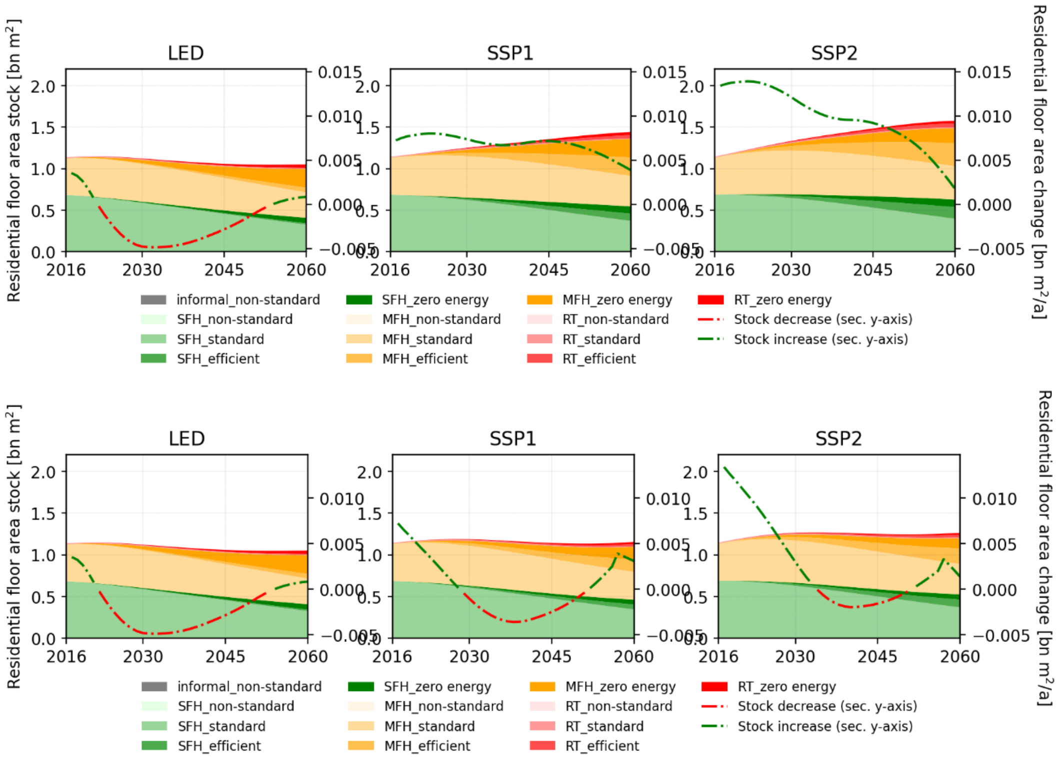

Supplementary Figure 6.12: Drivers and stock parameters, residential buildings, Oth_R32EU12-H. Top row: no material efficiency strategies (MES) included, bottom row: full MES spectrum. Results are shown for the 2°C-compatible climate polity scenario.

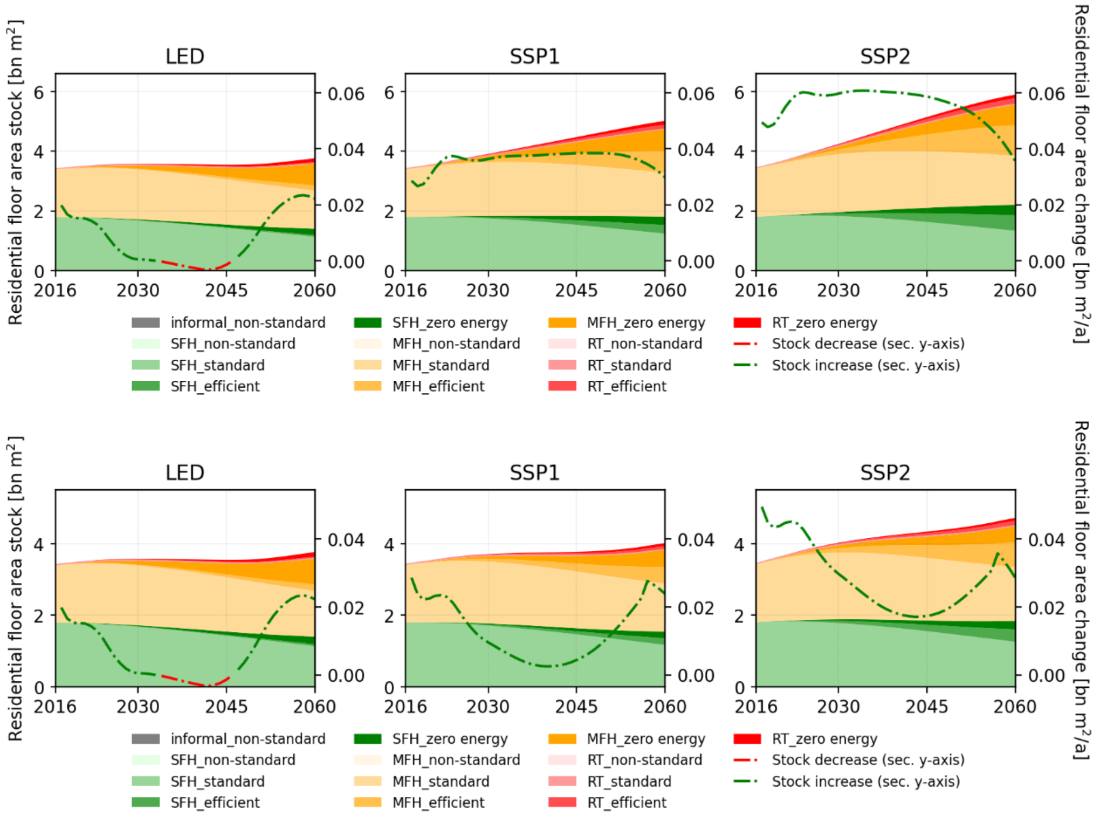

Supplementary Figure 6.13: Drivers and stock parameters, residential buildings, Oth_R32EU15. Top row: no material efficiency strategies (MES) included, bottom row: full MES spectrum. Results are shown for the 2°C-compatible climate polity scenario.

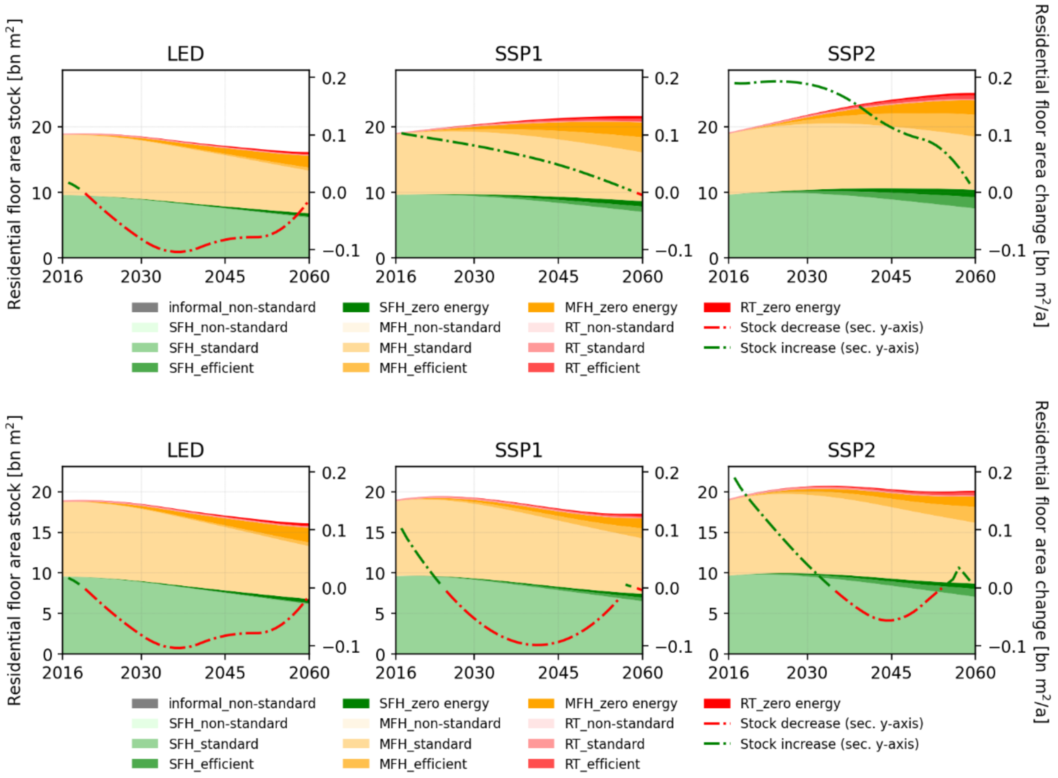

Supplementary Figure 6.14: Drivers and stock parameters, residential buildings, EU28. Top row: no material efficiency strategies (MES) included, bottom row: full MES spectrum. Results are shown for the 2°C-compatible climate polity scenario.

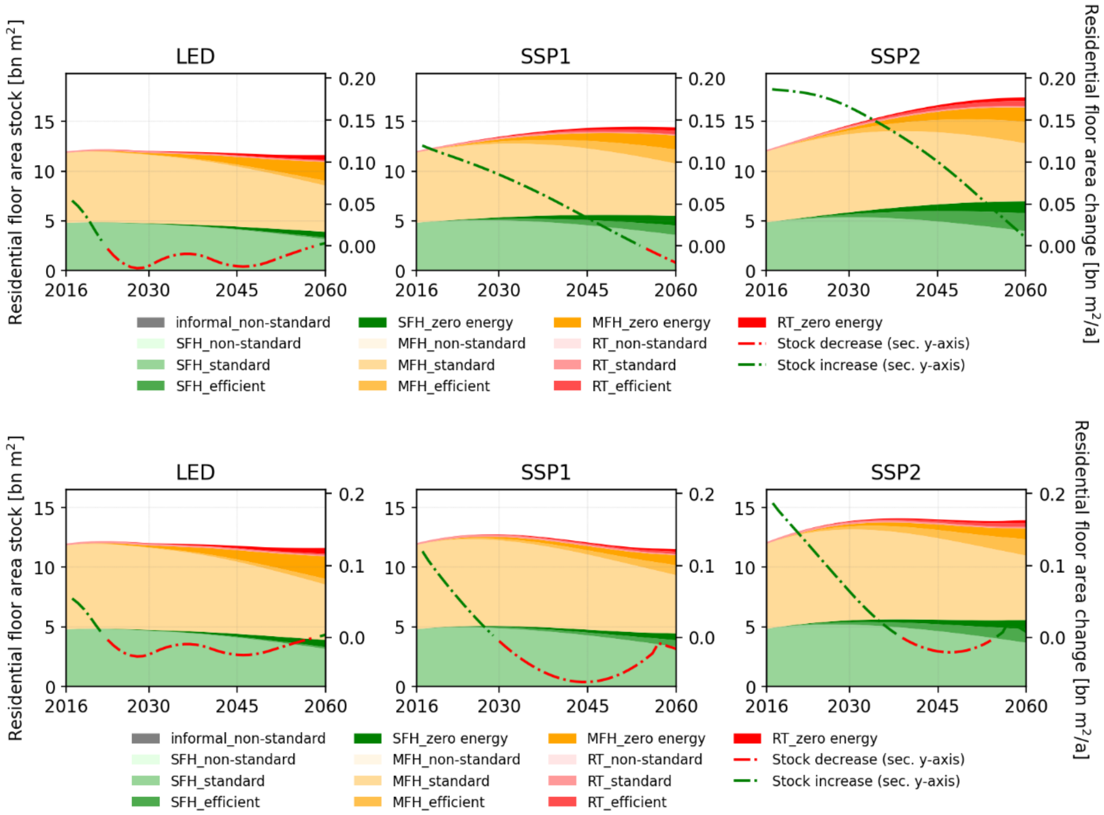

Supplementary Figure 6.15: Drivers and stock parameters, residential buildings, R5.2OECD_Other. Top row: no material efficiency strategies (MES) included, bottom row: full MES spectrum. Results are shown for the 2°C-compatible climate polity scenario.

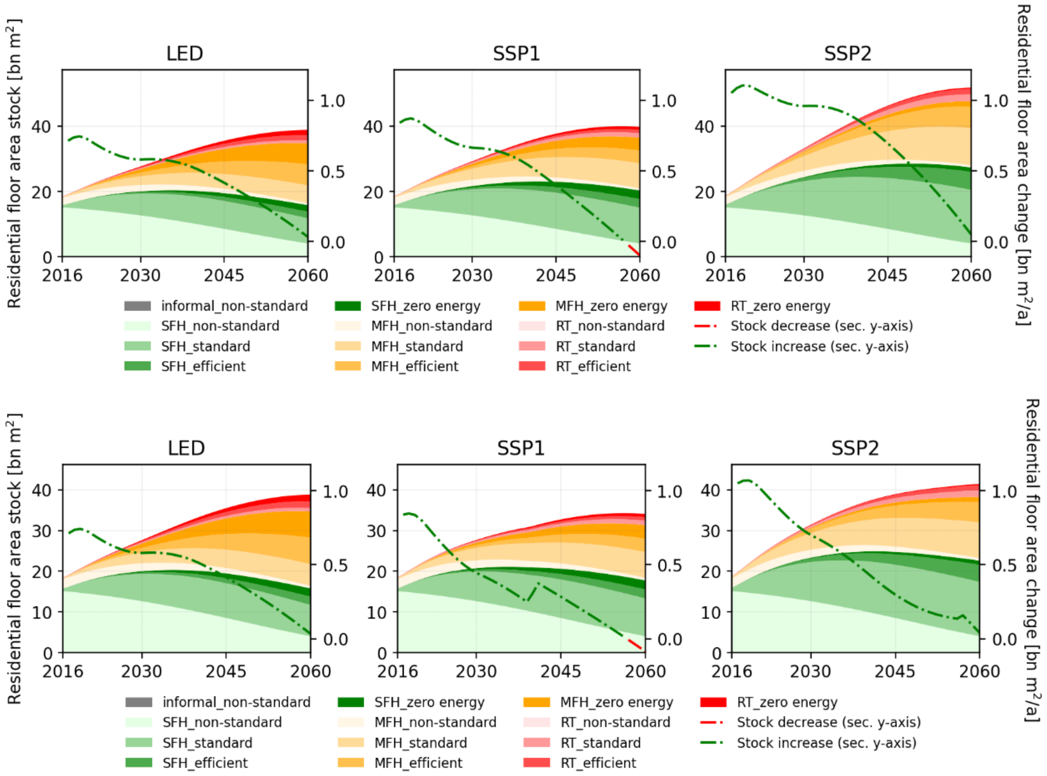

Supplementary Figure 6.16: Drivers and stock parameters, residential buildings, R5.2Asia_Other. Top row: no material efficiency strategies (MES) included, bottom row: full MES spectrum. Results are shown for the 2°C-compatible climate polity scenario.

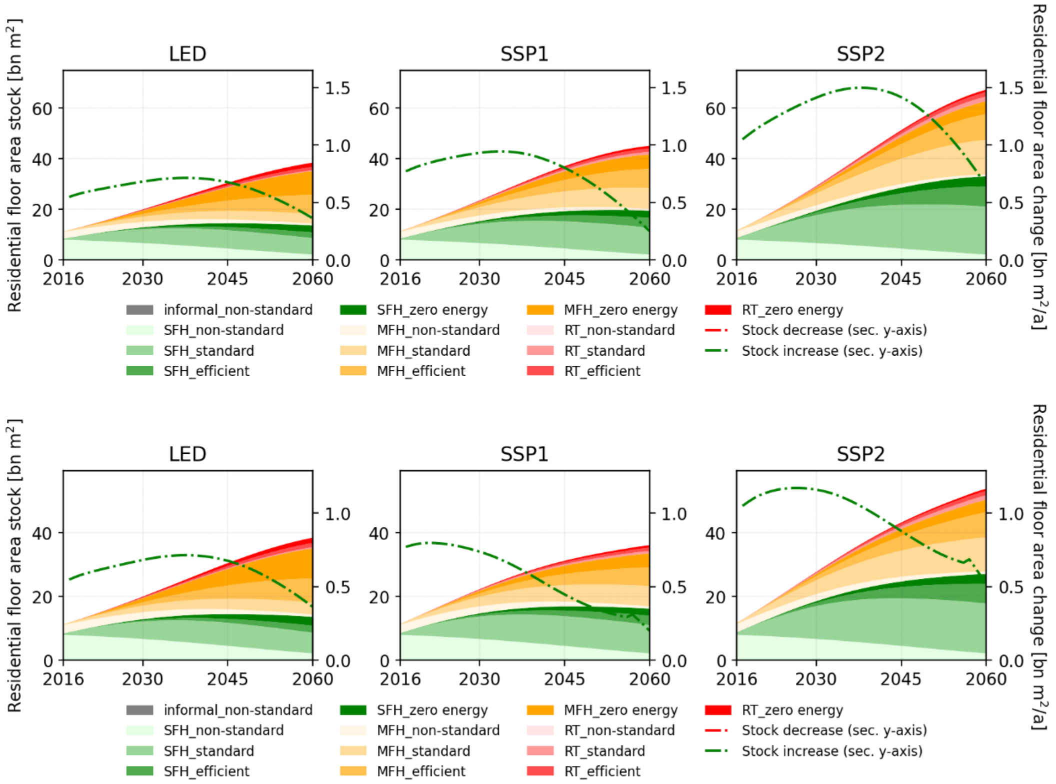

Supplementary Figure 6.17: Drivers and stock parameters, residential buildings, R5.2SSA_Other. Top row: no material efficiency strategies (MES) included, bottom row: full MES spectrum. Results are shown for the 2°C-compatible climate polity scenario.

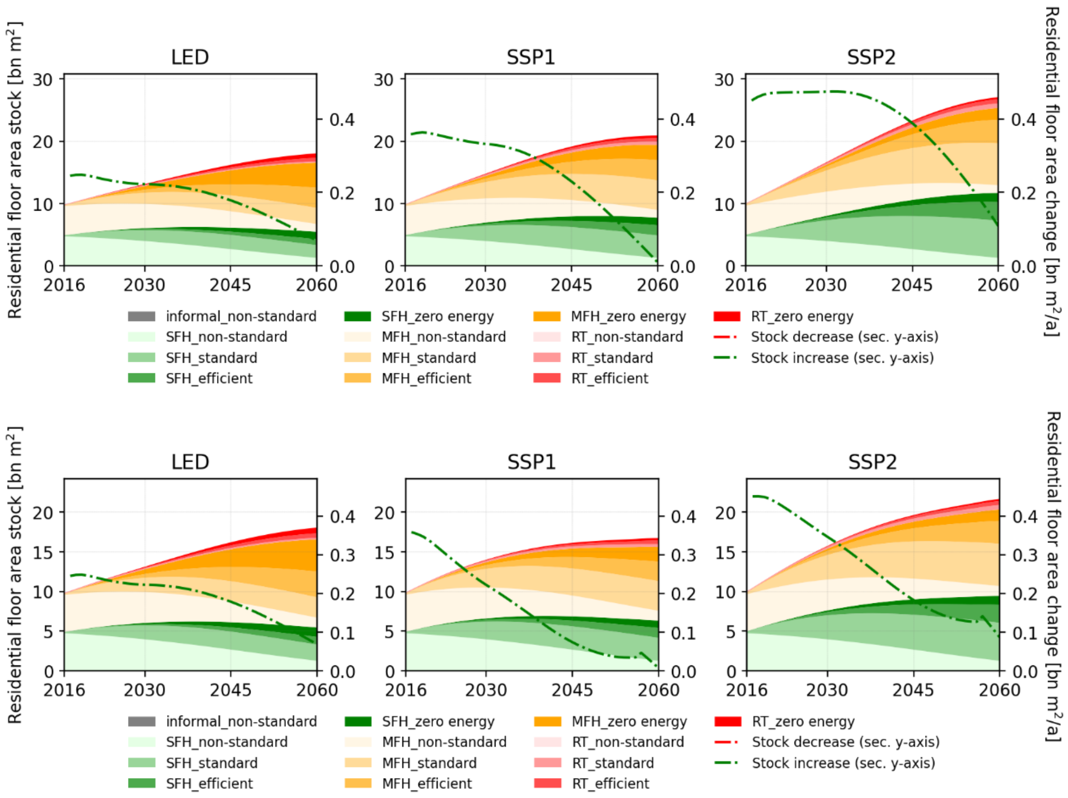

Supplementary Figure 6.18: Drivers and stock parameters, residential buildings, R5.2MNF_Other. Top row: no material efficiency strategies (MES) included, bottom row: full MES spectrum. Results are shown for the 2°C-compatible climate polity scenario.

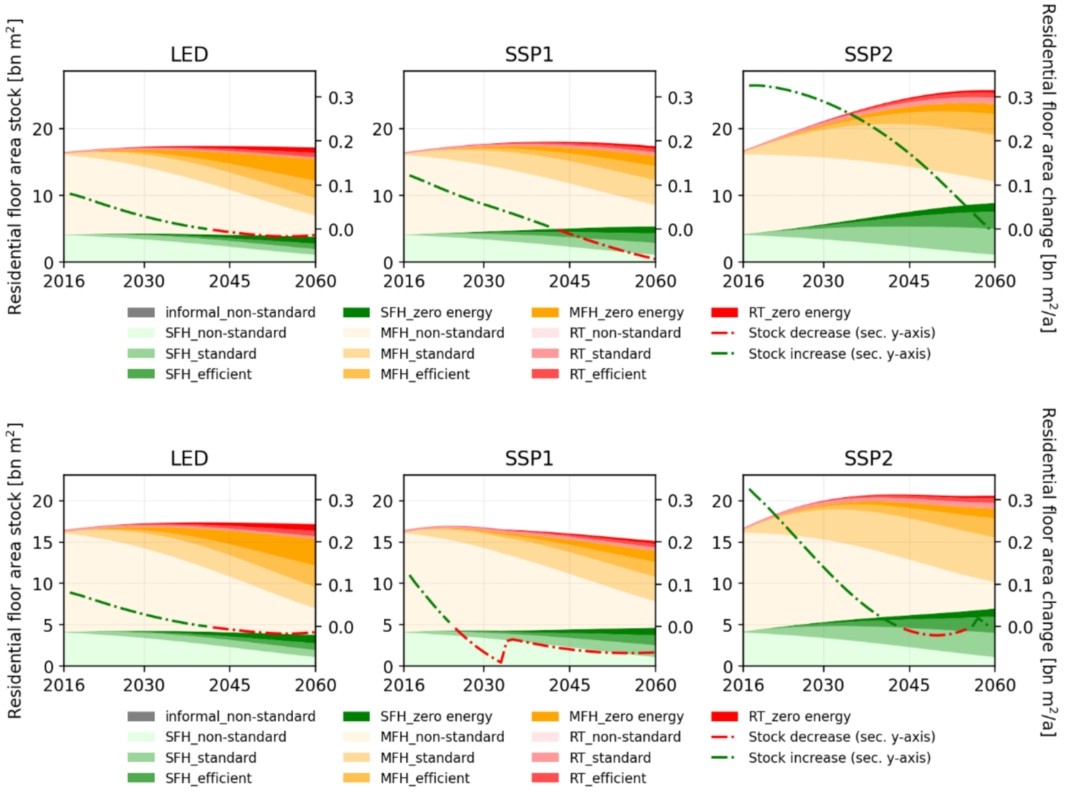

Supplementary Figure 6.19: Drivers and stock parameters, residential buildings, R5.2LAM_Other. Top row: no material efficiency strategies (MES) included, bottom row: full MES spectrum. Results are shown for the 2°C-compatible climate polity scenario.

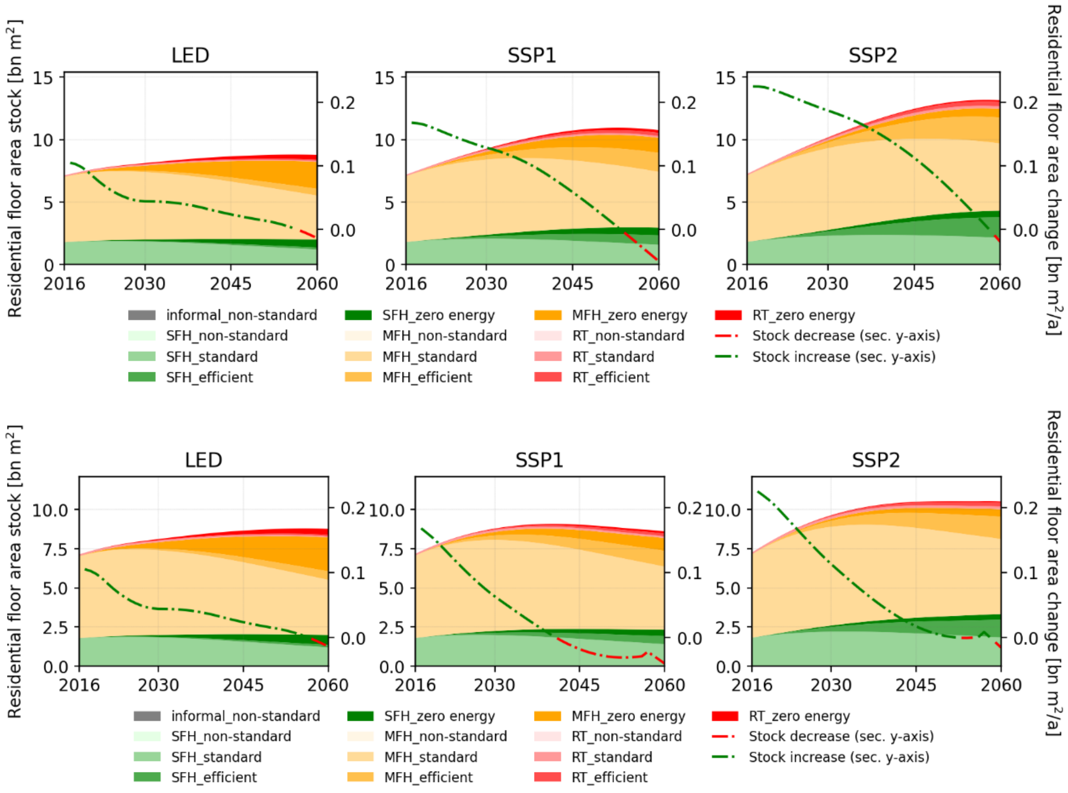

Supplementary Figure 6.20: Drivers and stock parameters, residential buildings, R5.2REF_Other. Top row: no material efficiency strategies (MES) included, bottom row: full MES spectrum. Results are shown for the 2°C-compatible climate polity scenario.

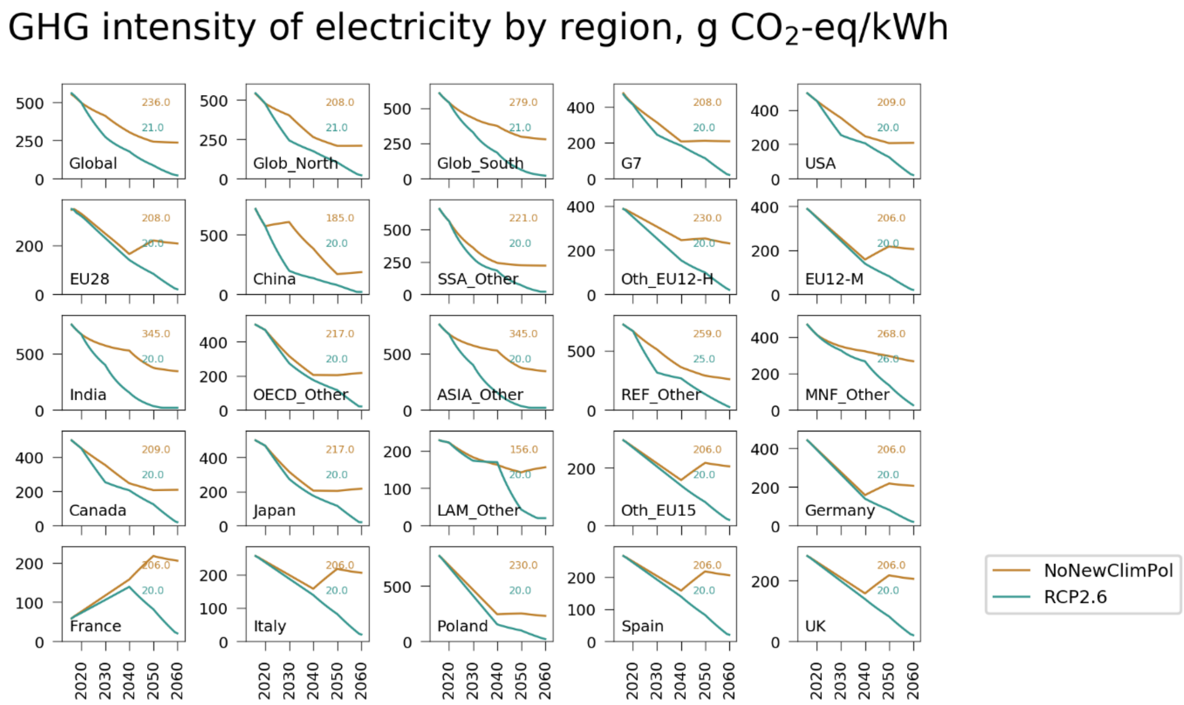

Supplementary Figure 7: GHG (CO_2, CH_4, and N_2O) intensity of electricity generation by region. The numbers shown indicate the values for 2060.

5. Additional Results and Discussion

Detailed description of Figure 2 in the paper:

Passenger vehicles, top row:

The striking difference in development is that emissions in the Global North and its constituting regions decline steadily over time, in all scenarios. In the Global South, they tend to peak between 2030 and 2040 (2°C scenario) or continue to increase (NoNewClimPol scenario). This behavior is a direct consequence of contraction and convergence of service levels: Global North service levels decline and are provided more efficiently, allowing for absolute decoupling of GHG emissions. Global South service levels increase substantially in most regions (current car ownership rate in Sub-Saharan Africa is ca. 18 cars per 1000 people), and fuel shift, low carbon energy supply, and ME can counter this strong growth trend only in the 2°C scenario and only after 2030. For China, the trend reversal can happen earlier, between 2025 and 2030.

In the SSP1 (easy mitigation and adaptation) scenario, the relative impact of full ME is similar in both climate policy scenarios, because both non-ME baselines are material-intensive, with a 15 year product lifetime and substantial improvement potential for end-of-life scrap recovery especially for plastics but also for the other materials. The shift to smaller segments and light cars and the car- and ride-sharing strategies are important contributors as well.

Residential buildings, bottom row:

Here, all regions show a huge GHG emissions reduction potential, as – despite strong growth in the regions of the Global South – the emissions reductions from increased energy efficiency in buildings outpace all assumed growth. Still, especially in the NoNewClimPol scenario, 2050 emissions without ME are nowhere near carbon neutrality, which is an aspiration for the easy-to-mitigate residential building sector in some strategy portfolios (zero emission buildings). Here, the ME strategies can prove particularly effective in achieving deep emissions cuts: In some regions, including the global total, Global South, China, India, and Sub-Saharan Africa, The 2°C-plus-ME GHG emissions are only a fraction of the 2°C-no-ME emissions, and for some regions (for SSP1: Global South, Other Asia, Middle East-Northern Africa, and India), forest carbon uptake associated with residential building timber use even leads to total negative emissions.

Regarding the forest regrowth modelling: There are of course some big assumptions here: that sustainable harvest exists and is possible at the scale required, that forests would not have added carbon to storage without timber removal, and there is no opportunity cost of the timber no longer being available for other carbon-saving measures.

Actual development 2016-2021:

We have not yet investigated in detail the question on which GHG emissions trajectory the two studied sectors are currently on. An update will be necessary in the coming 2-3 years to trace the actual development, assess the effect of existing policy, and adapt the scenario assumptions. Between 2015 (base year of the RECC model) and 2020, urban expansion continued and related GHG emissions rose in most world regions, and the actual development roughly followed the SSP1 and SSP2 trajectories. The LED scenario is a stark deviation from current growth patterns and its service levels would only be attainable in a world with very strong climate policy, combined with widespread lifestyle changes. The COVID 19 pandemic will have an impact on both growth (economic development) and lifestyle in the different regions, which will be visible in the emissions patterns together with the effect of climate policy.

More details for Figure 4 in the paper:

Secondary material production is determined from scrap supply (all available scrap is recycled) and may be exported to other sectors if excessive. Primary production is determined to satisfy demand for stock expansion and for high quality material.

Around 2020, material demand for the two sectors is mainly fuelled by primary material production from natural resources. This is because of the still expanding in-use stock, the lack of concrete re-use, and the demand for high-quality primary material especially for vehicles. Over time, the situation changes as stocks grow further, start saturating eventually, and more end-of-life material becomes available. Globally, for the two sectors combined, secondary material availability (determined largely from historic consumption) can overtake primary material demand, meaning that the average recycled content of new buildings and vehicles will exceed 50%. Depending on the degree of ME, this is the case for steel (2025-2048), aluminium (ca. 2025), copper (before 2020), and plastics (ca. 2035 for full ME). Still, some amounts, albeit low, of primary material production are needed even in high ME futures to allow for stock expansion and compensation of dissipative and irreversible losses.

5.1. Emissions by region, sector, and scenario

In all plots below, 'pav' stands for passenger vehicles and 'reb' for residential buildings.

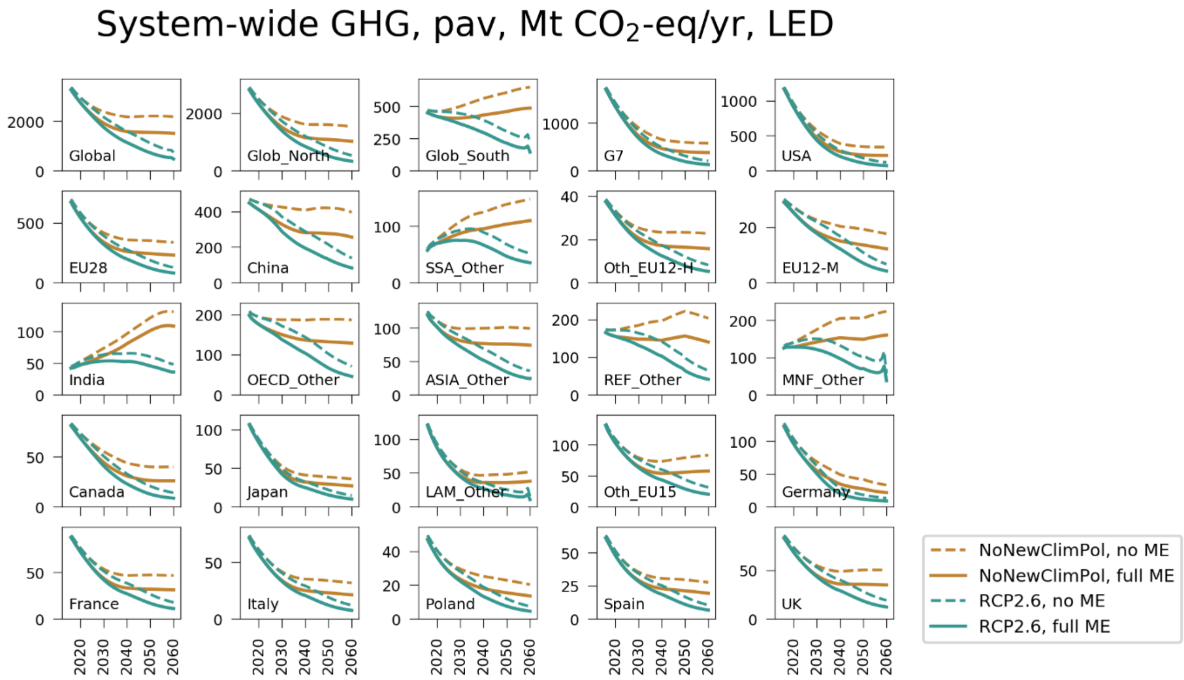

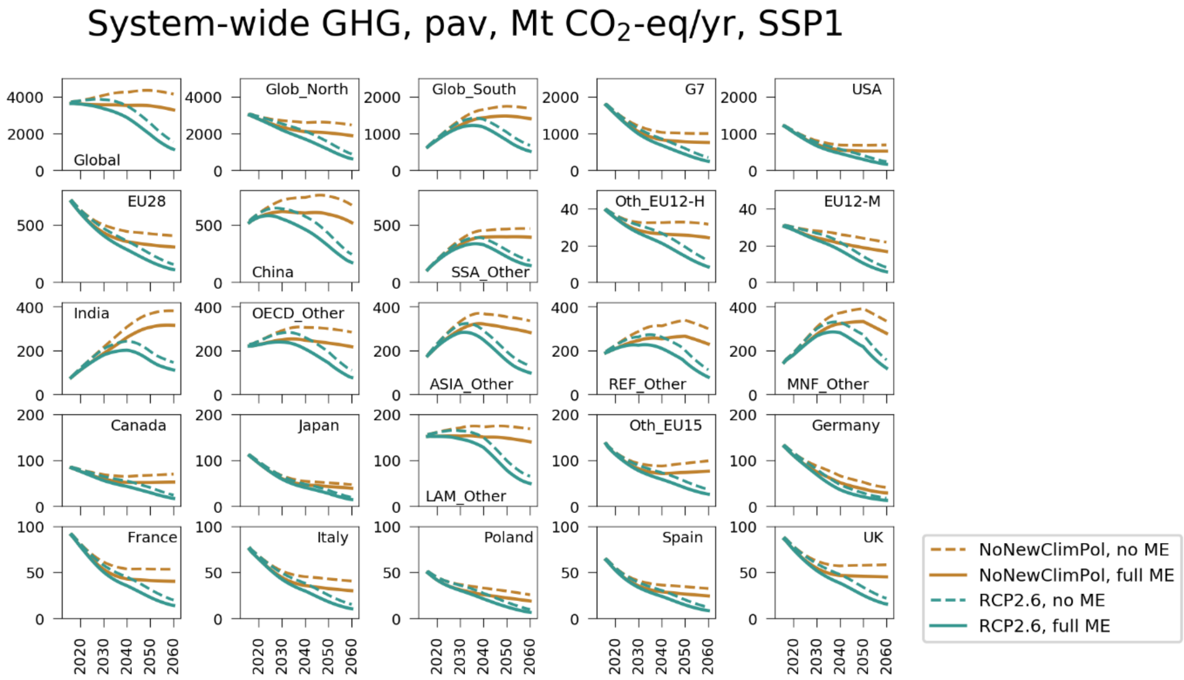

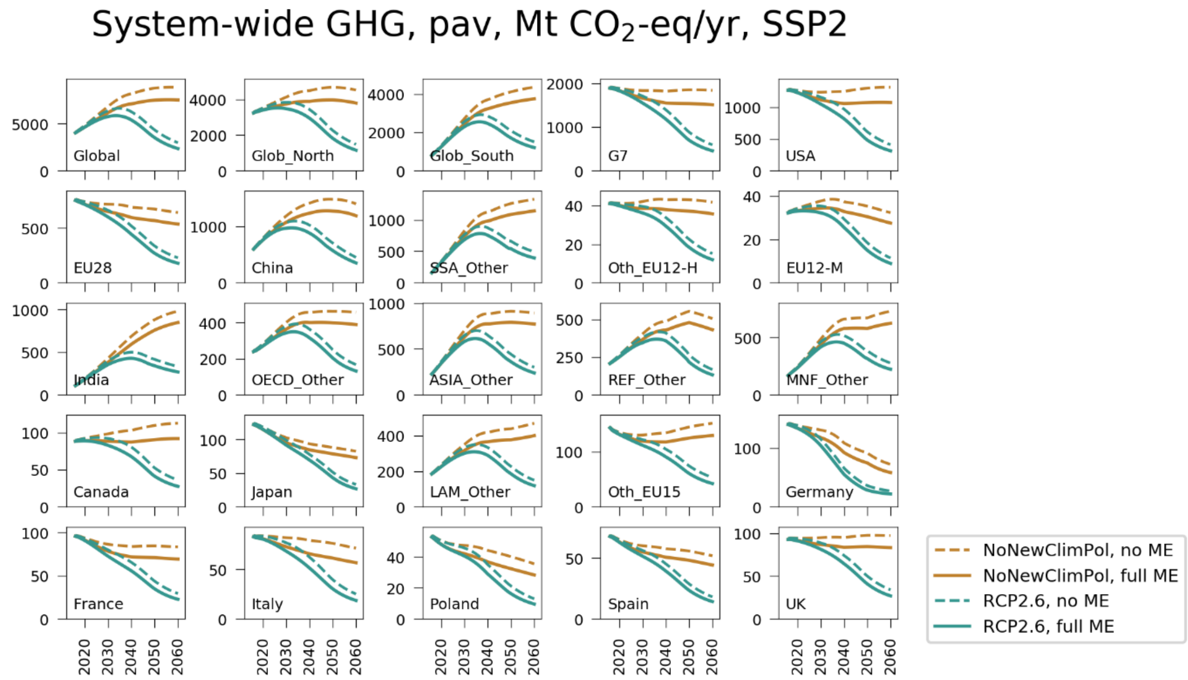

Supplementary Figure 8: Emissions by region and socioeconomic/policy scenario plus RES on/off.

Passenger vehicles (pav). In the plots above, 'pav' stands for passenger vehicles.

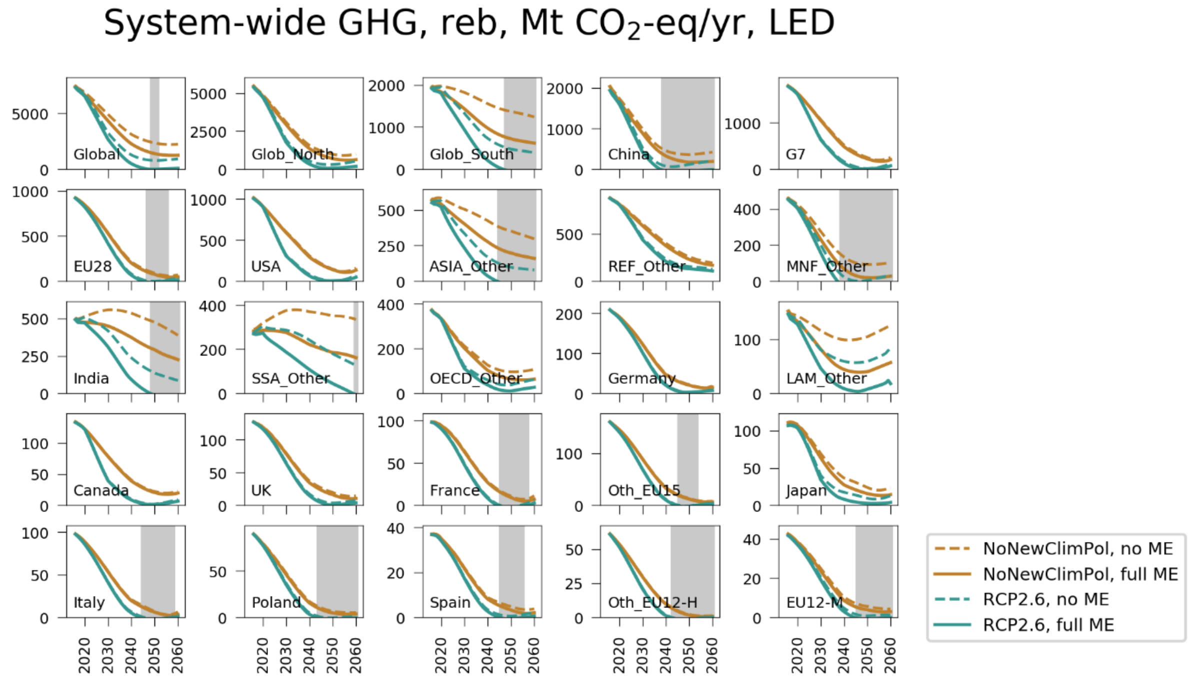

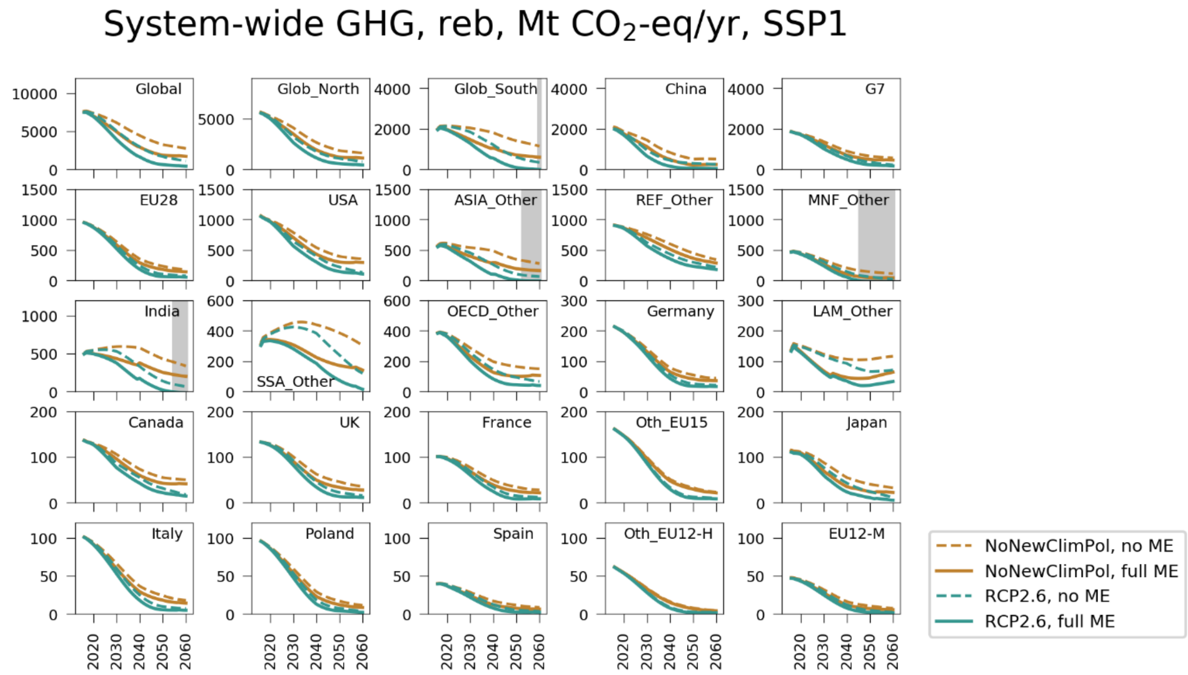

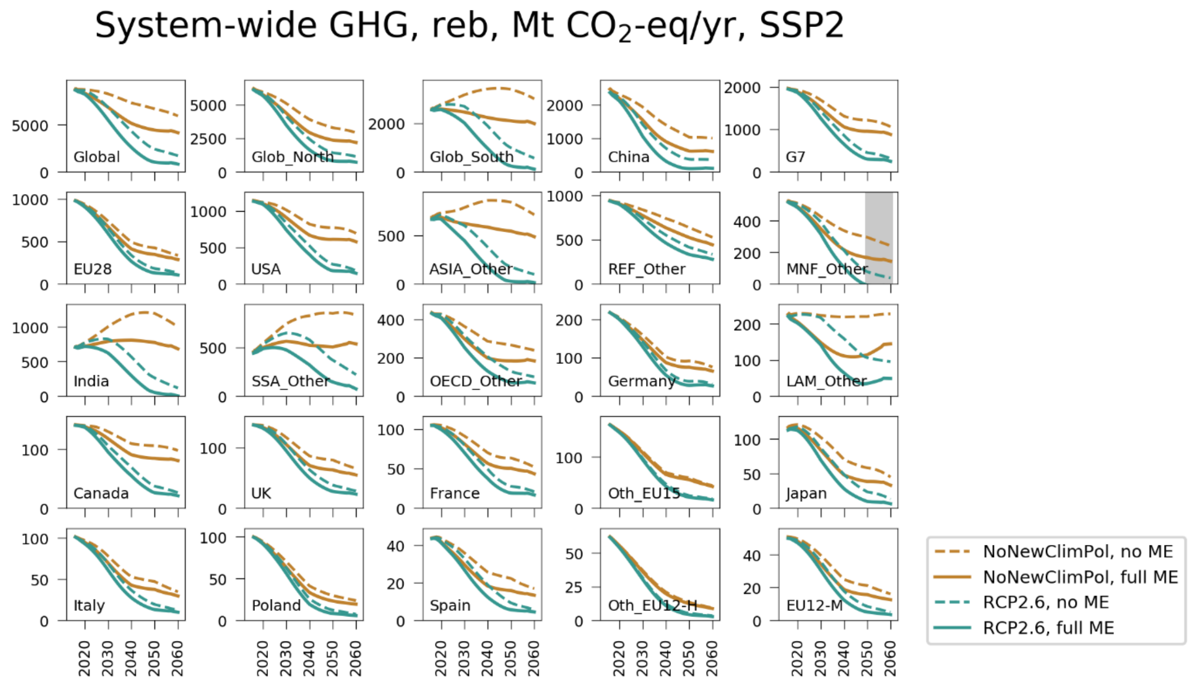

Supplementary Figure 9: Emissions by region and socioeconomic/policy scenario plus RES on/off. Residential buildings (reb). Grey shaded areas highlight periods of regional carbon negativity, which is due to forest carbon uptake as a consequence of previous timber harvesting and regrowth.

5.2. Cumulative emissions savings 2016-2060 for the different resource efficiency strategies (RES), regions, socioeconomic scenarios, and sectors

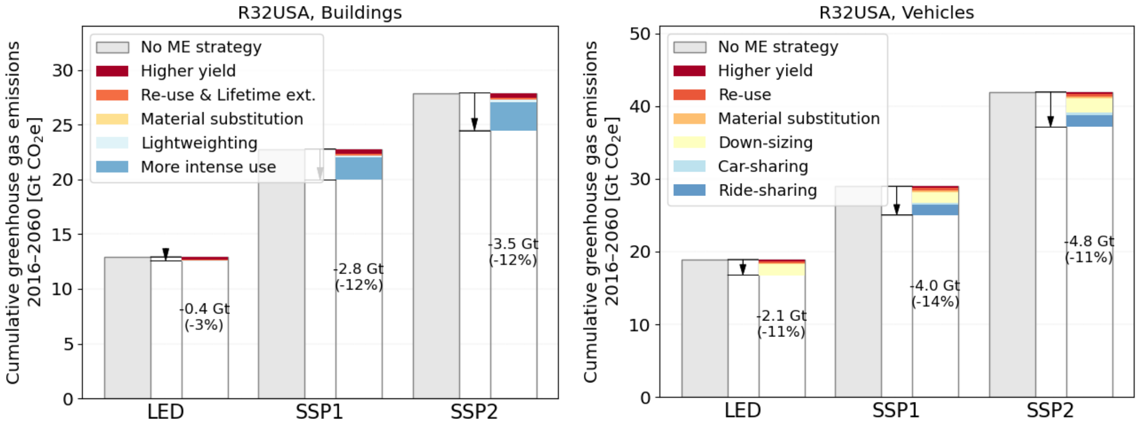

Supplementary Figure 10.1: RES impacts on cumulative emissions, 2016-2060, passenger vehicles and residential buildings, USA. For RCP2.6.

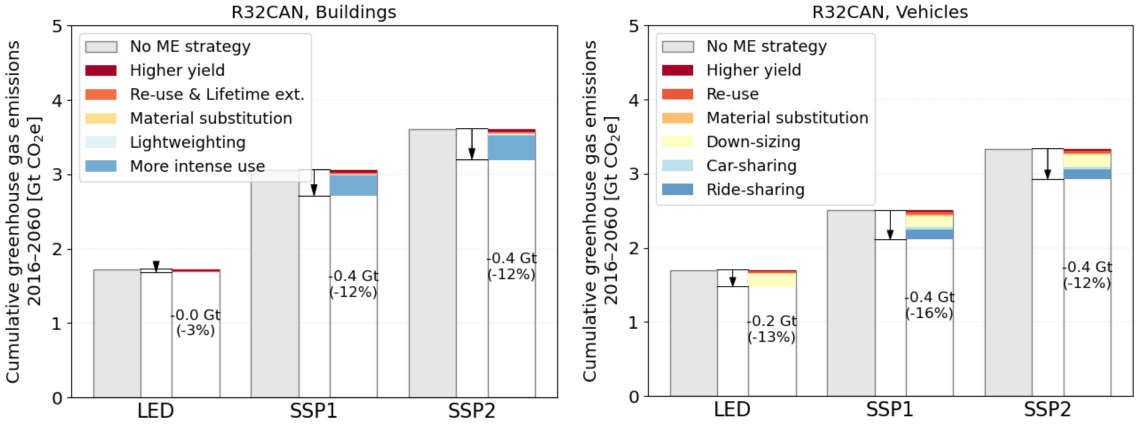

Supplementary Figure 10.2: RES impacts on cumulative emissions, 2016-2060, passenger vehicles and residential buildings, Canada. For RCP2.6.

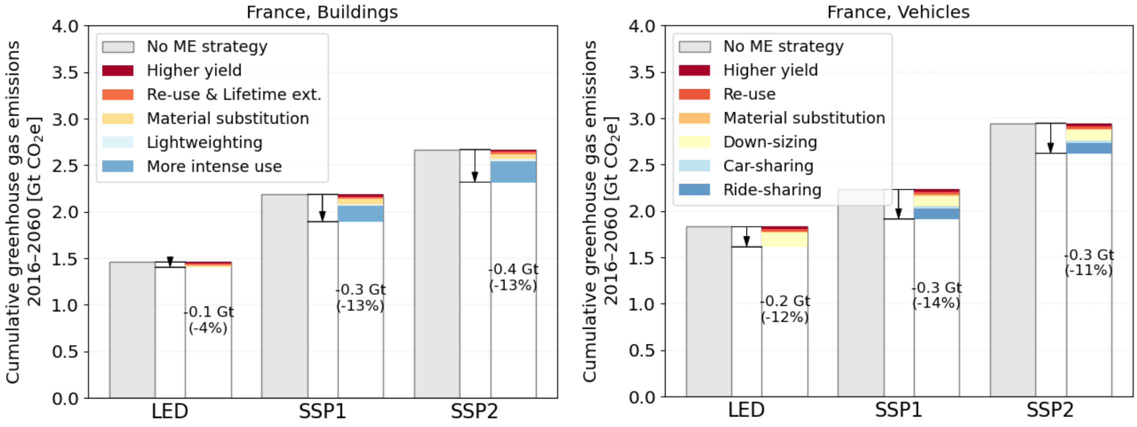

Supplementary Figure 10.3: RES impacts on cumulative emissions, 2016-2060, passenger vehicles and residential buildings, France. For RCP2.6

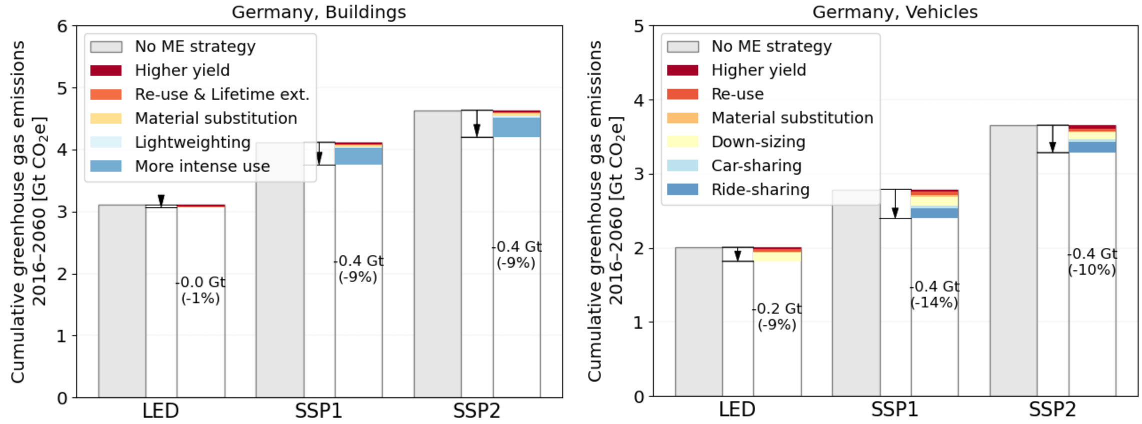

Supplementary Figure 10.4: RES impacts on cumulative emissions, 2016-2060, passenger vehicles and residential buildings, Germany. For RCP2.6

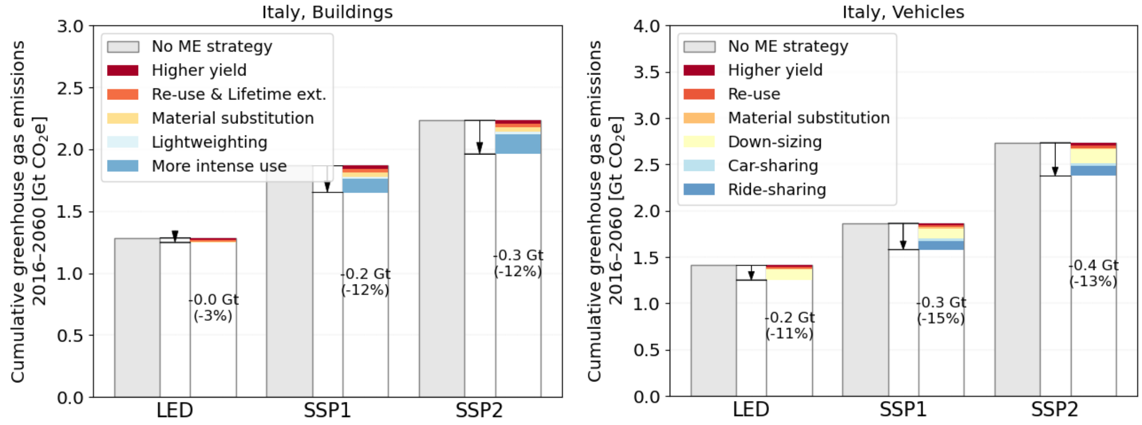

Supplementary Figure 10.5: RES impacts on cumulative emissions, 2016-2060, passenger vehicles and residential buildings, Italy. For RCP2.6

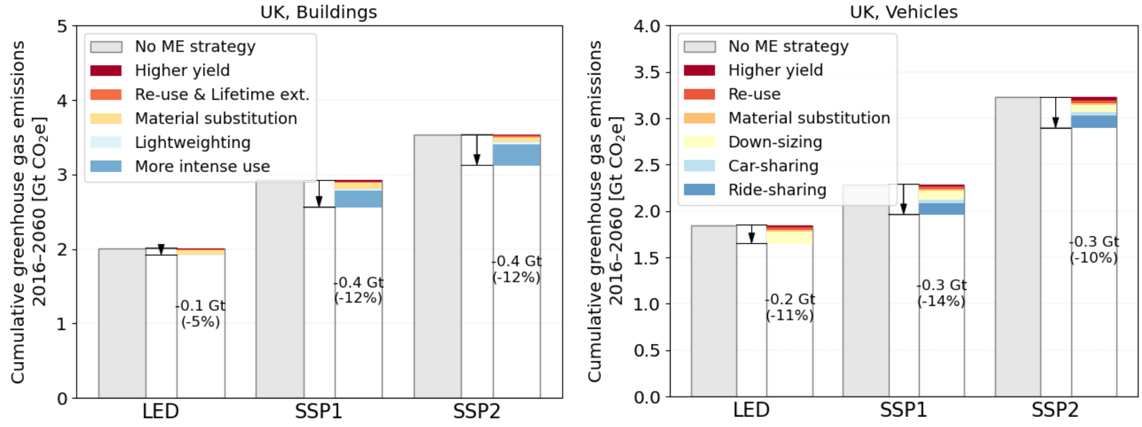

Supplementary Figure 10.6: RES impacts on cumulative emissions, 2016-2060, passenger vehicles and residential buildings, UK. For RCP2.6.

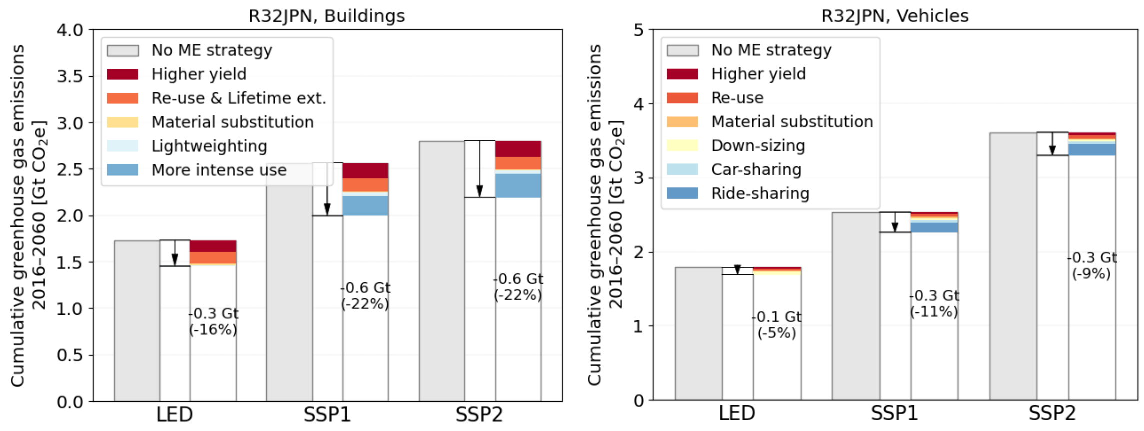

Supplementary Figure 10.7: RES impacts on cumulative emissions, 2016-2060, passenger vehicles and residential buildings, Japan. For RCP2.6.

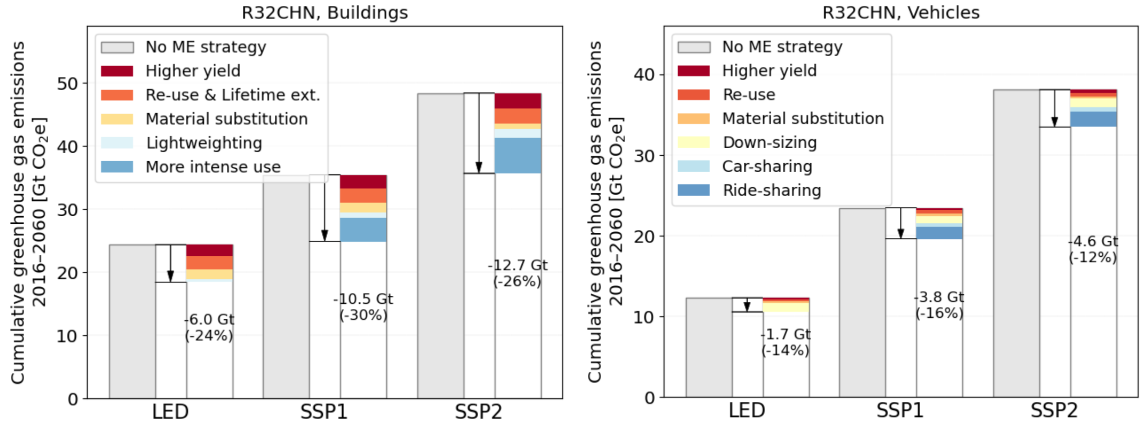

Supplementary Figure 10.8: RES impacts on cumulative emissions, 2016-2060, passenger vehicles and residential buildings, China. For RCP2.6.

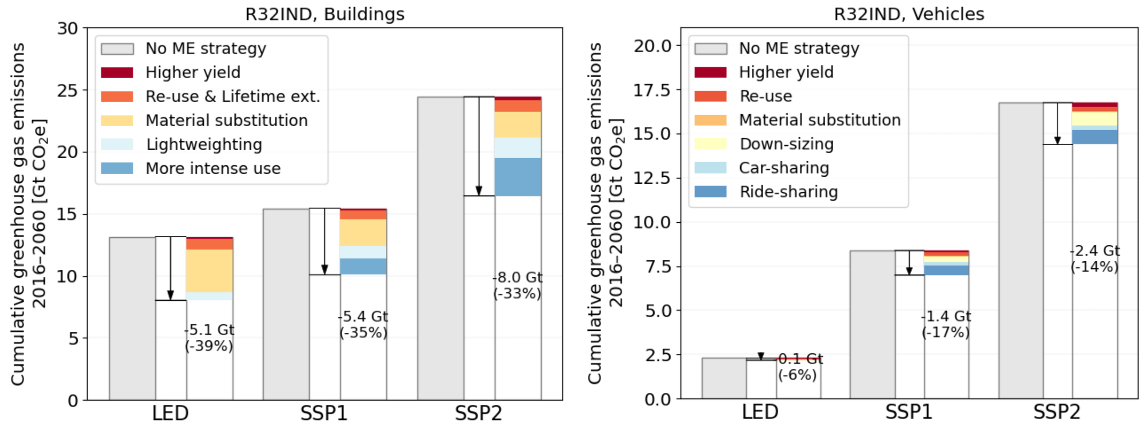

Supplementary Figure 10.9: RES impacts on cumulative emissions, 2016-2060, passenger vehicles and residential buildings, India. For RCP2.6.

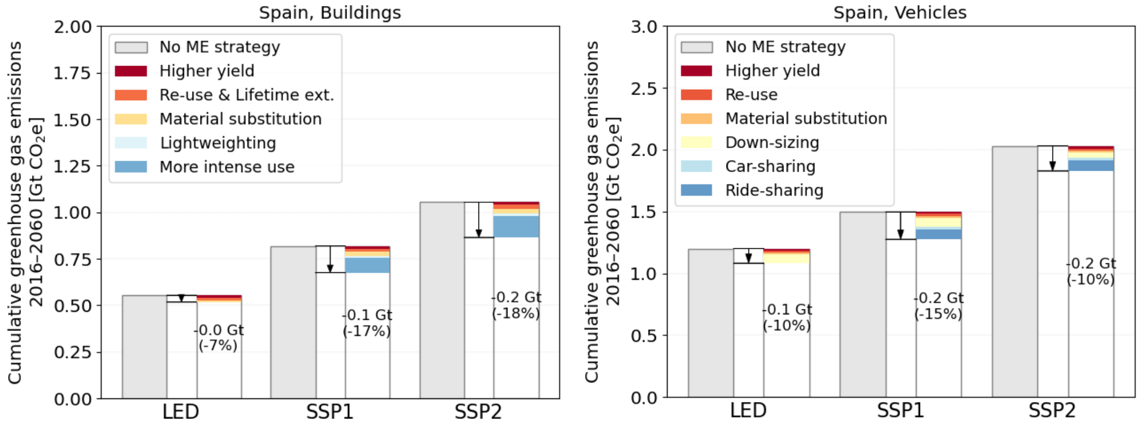

Supplementary Figure 10.10: RES impacts on cumulative emissions, 2016-2060, passenger vehicles and residential buildings, Spain. For RCP2.6

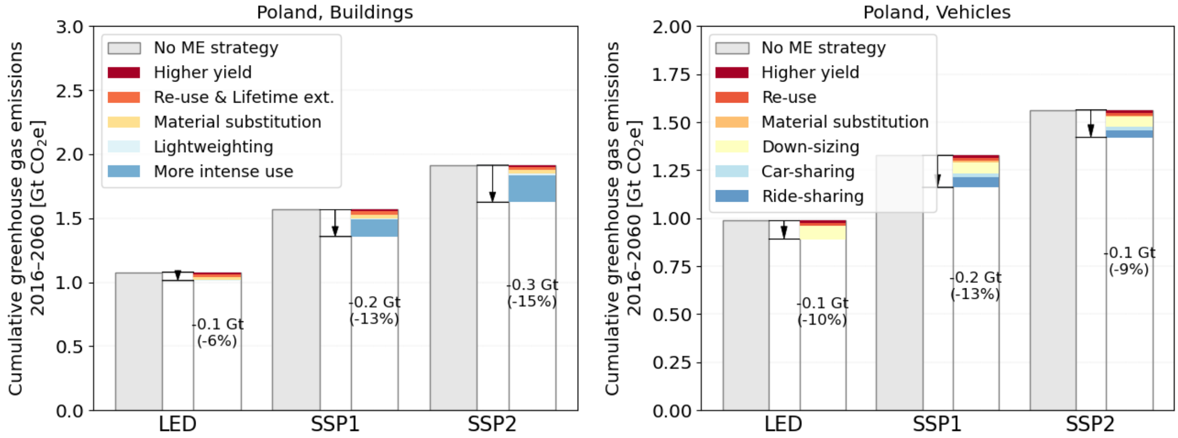

Supplementary Figure 10.11: RES impacts on cumulative emissions, 2016-2060, passenger vehicles and residential buildings, Poland. For RCP2.6.

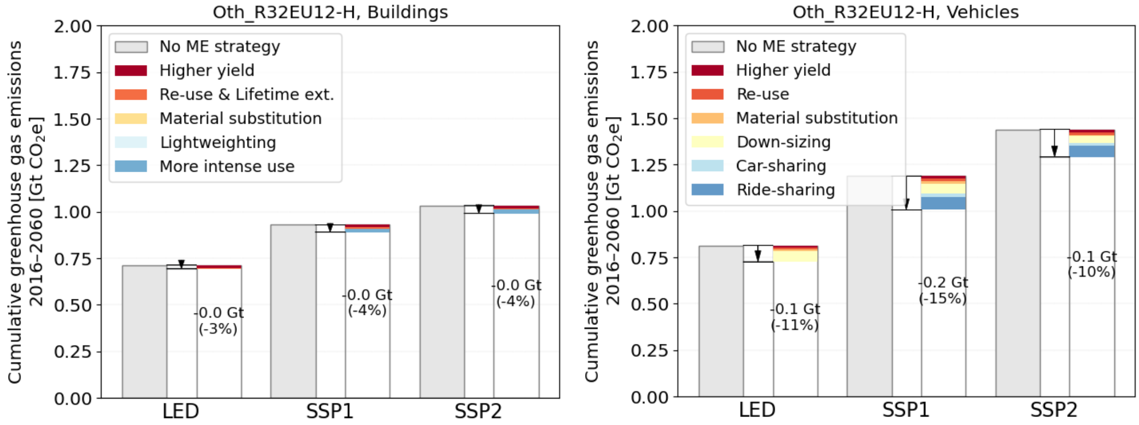

Supplementary Figure 10.12: RES impacts on cumulative emissions, 2016-2060, passenger vehicles and residential buildings, Oth_R32EU12-H. For RCP2.6.

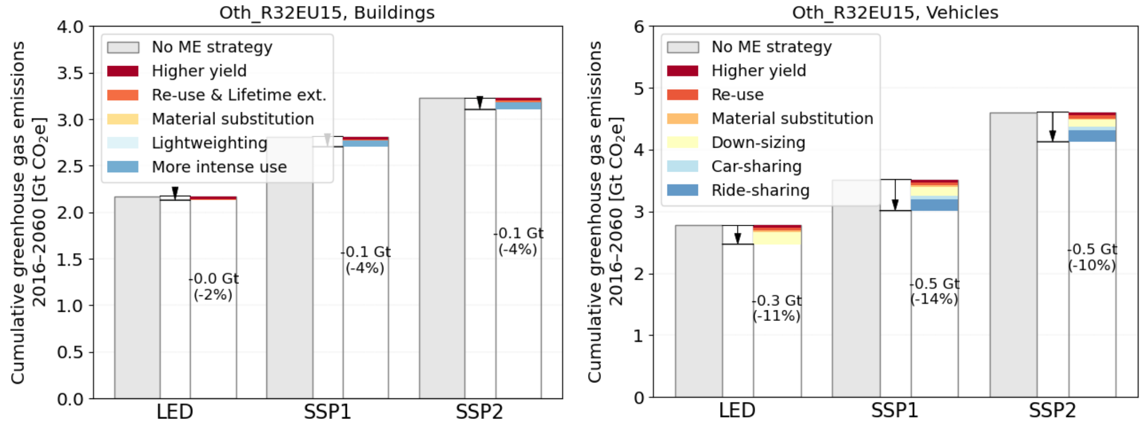

Supplementary Figure 10.13: RES impacts on cumulative emissions, 2016-2060, passenger vehicles and residential buildings, Oth_R32EU15. For RCP2.6.

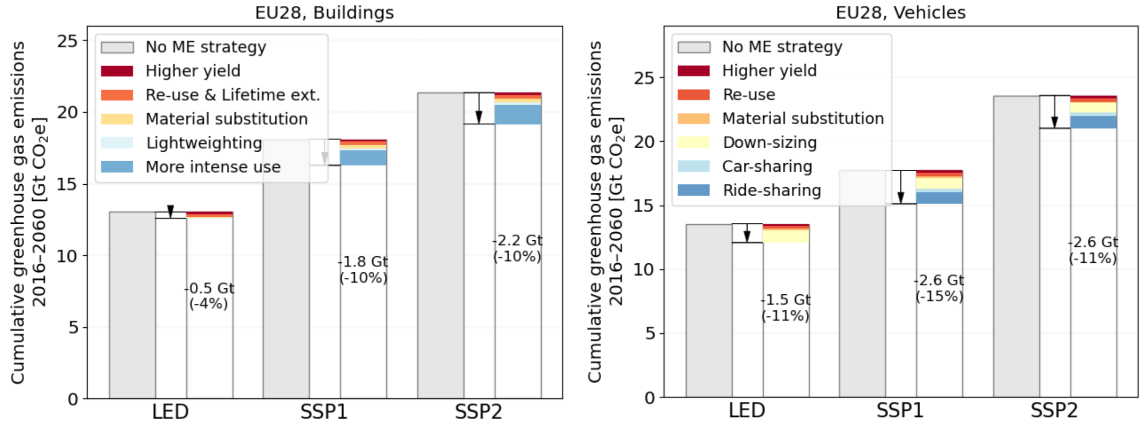

Supplementary Figure 10.14: RES impacts on cumulative emissions, 2016-2060, passenger vehicles and residential buildings, EU28. For RCP2.6.

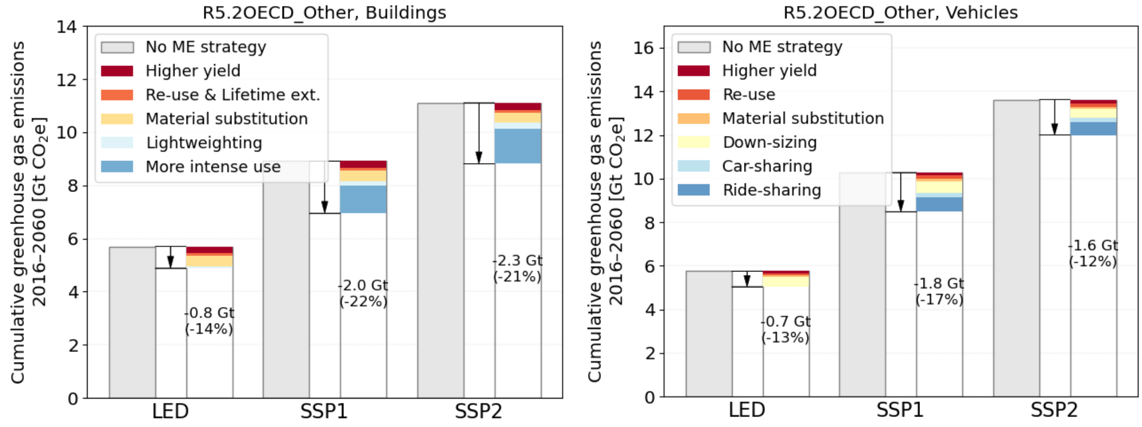

Supplementary Figure 10.15: RES impacts on cumulative emissions, 2016-2060, passenger vehicles and residential buildings, R5.2OECD_Other. For RCP2.6.

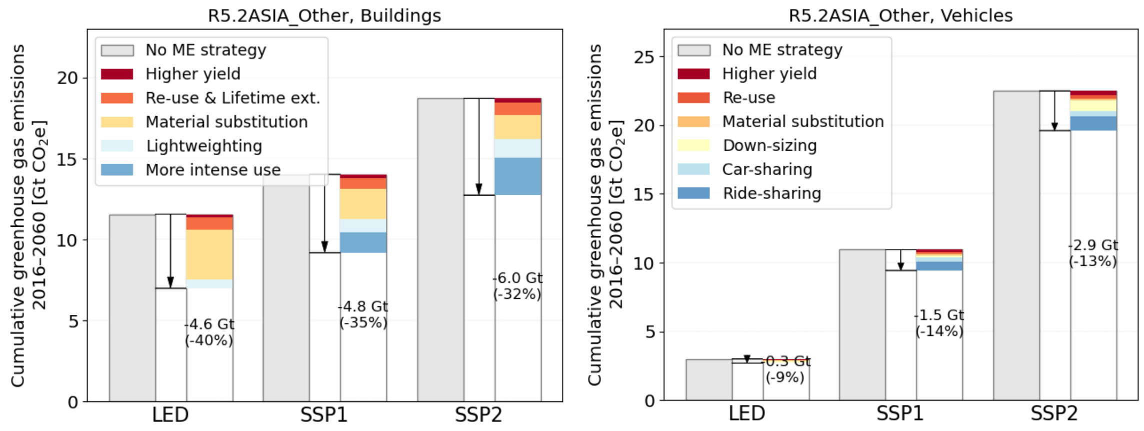

Supplementary Figure 10.16: RES impacts on cumulative emissions, 2016-2060, passenger vehicles and residential buildings, Asia_Other. For RCP2.6.

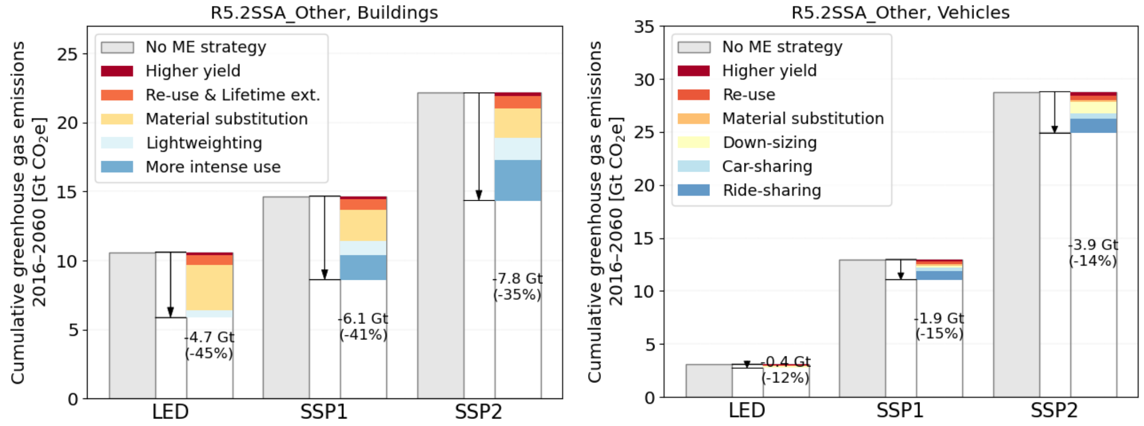

Supplementary Figure 10.17: RES impacts on cumulative emissions, 2016-2060, passenger vehicles and residential buildings, R5.2SSA_Other. For RCP2.6.

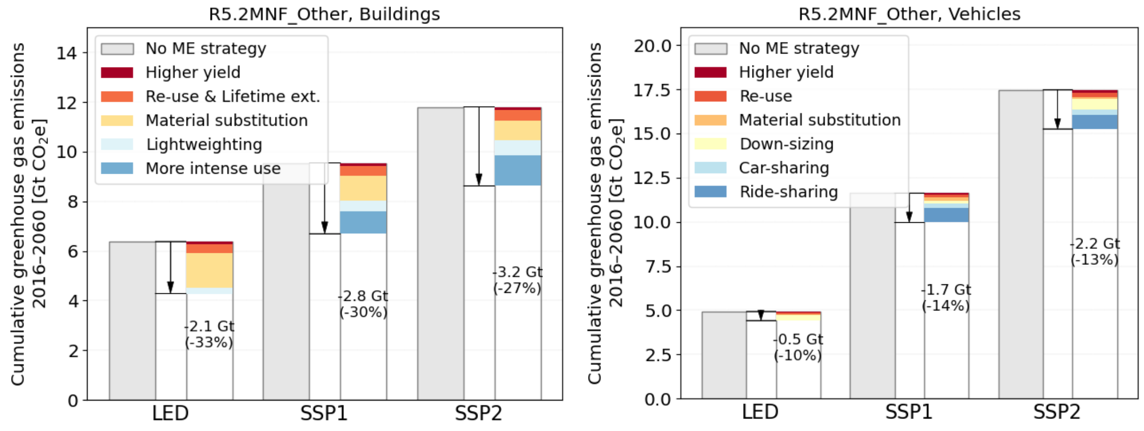

Supplementary Figure 10.18: RES impacts on cumulative emissions, 2016-2060, passenger vehicles and residential buildings, R5.2MNF_Other. For RCP2.6.

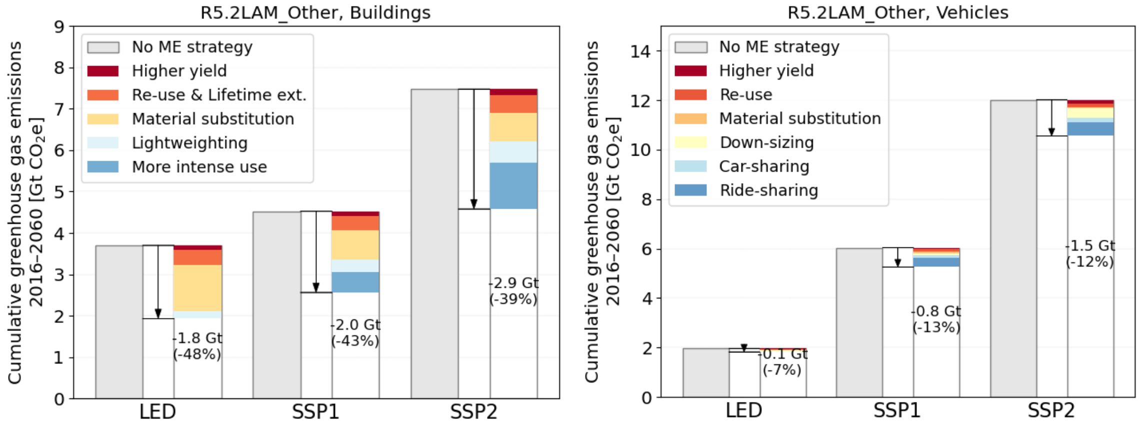

Supplementary Figure 10.19: RES impacts on cumulative emissions, 2016-2060, passenger vehicles and residential buildings, R5.2LAM_Other. For RCP2.6.

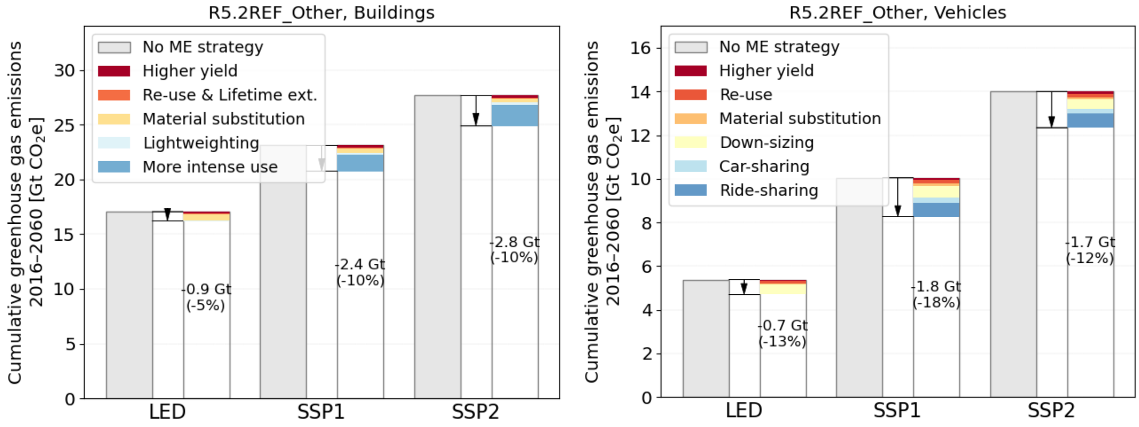

Supplementary Figure 10.20: RES impacts on cumulative emissions, 2016-2060, passenger vehicles and residential buildings, R5.2REF_Other. For RCP2.6.

5.3. Impact of material efficiency (ME) on the difficult-to-mitigate emissions in material production for the passenger vehicle ('pav') and residential building ('reb') sectors

Colors may deviate from legend colors due to overlap of RES wedges

no new climate policy (LEFT) RCP2.6 climate policy (RIGHT)

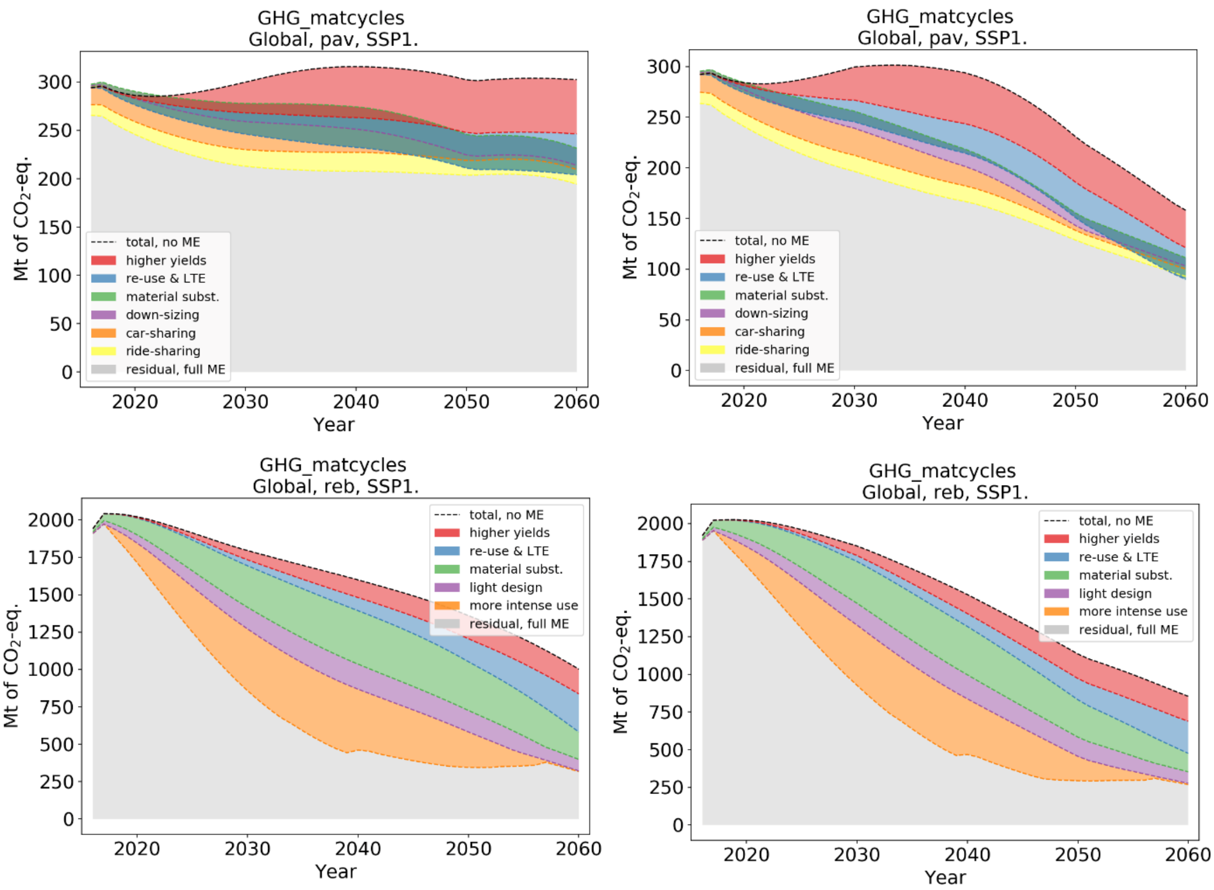

Supplementary Figure 11: Breakdown of the possible reduction of material production GHG for no new climate policy (LEFT) and an RCP2.6 climate policy (RIGHT). Passenger vehicles (top) and residential buildings (bottom) for SSP1. Colors may deviate from legend colors due to overlap of RES wedges.

Significant difference for Passenger vehicles (top) and residential buildings (bottom): Vehicles show large trade-offs to use phase (aluminium phase in, here: low-carbon energy can help (top right). Buildings have huge potential that occurs a bit earlier and larger for RCP2.6.

5.4. Impact of material efficiency (ME) on material cycle GHG emissions over time

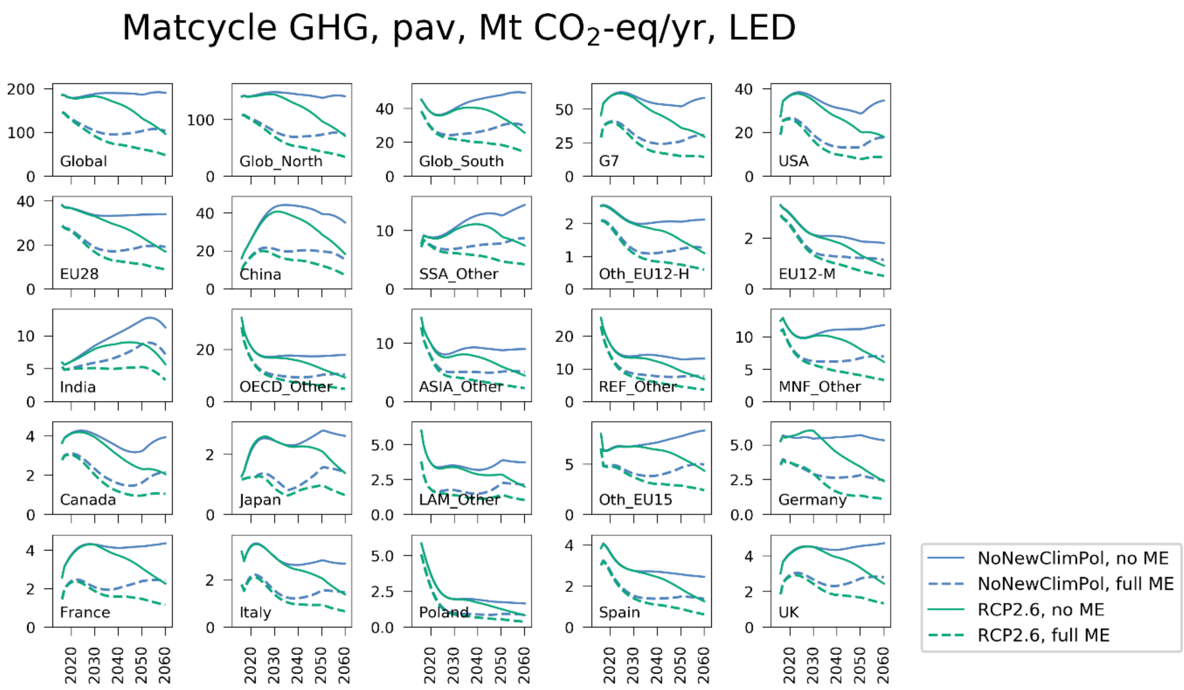

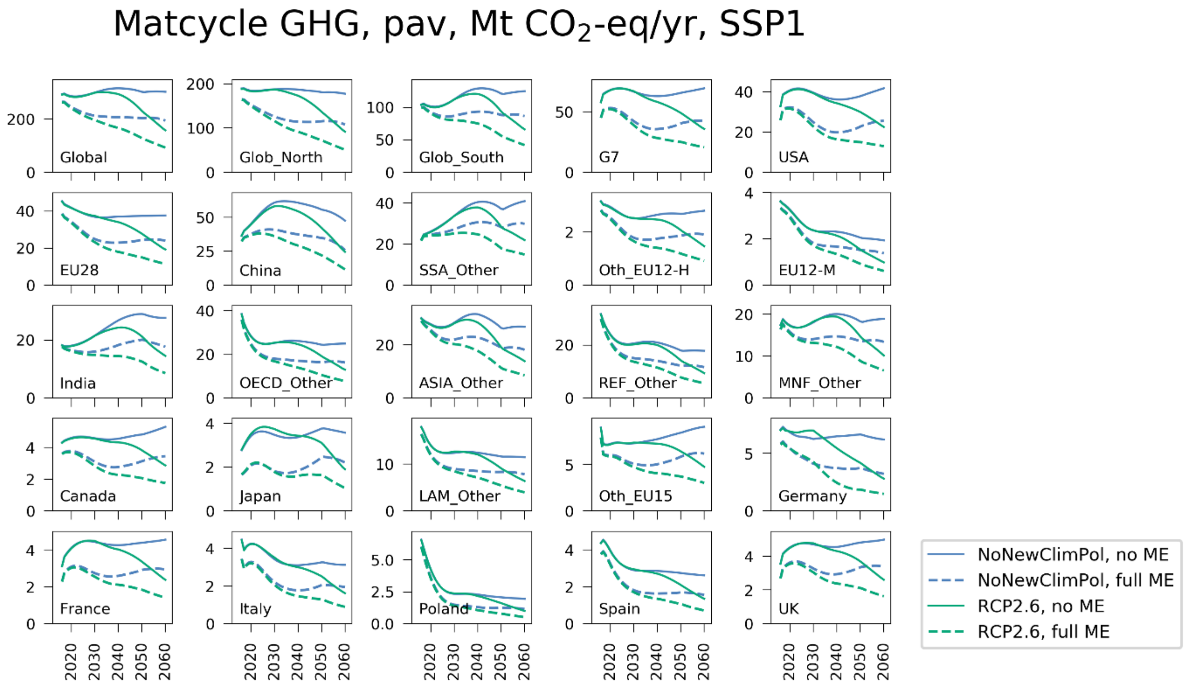

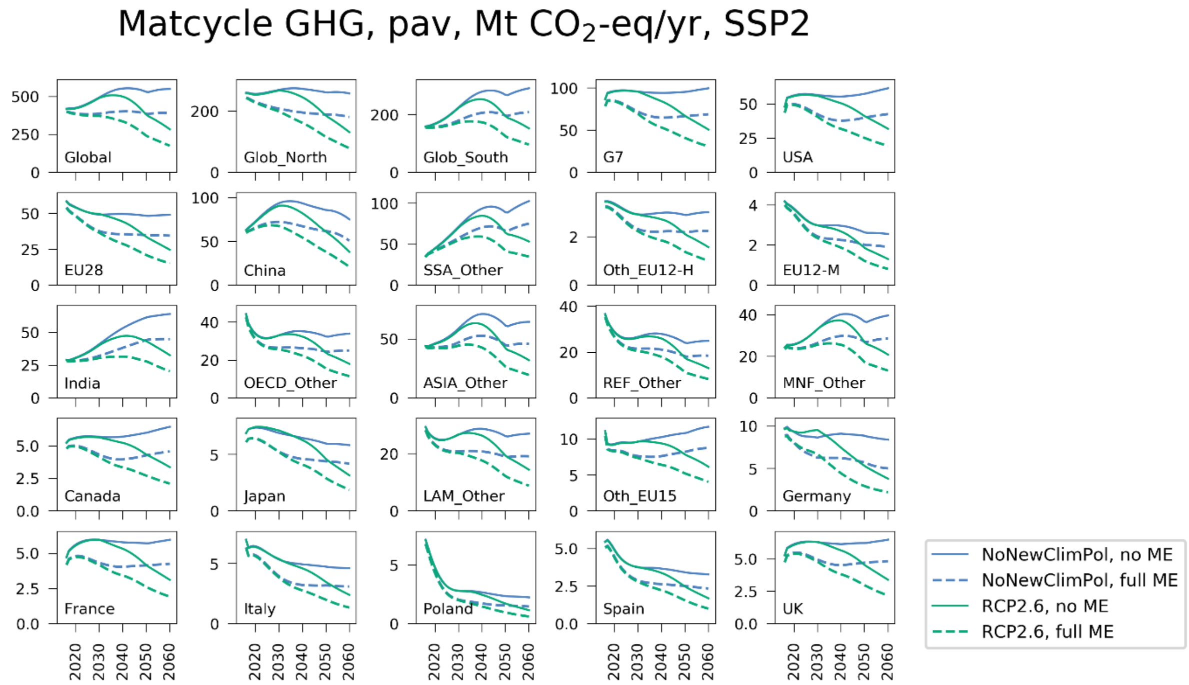

Supplementary Figure 12: Material cycle GHG emissions (for all materials combined), by region and climate policy/RES scenario, for passenger vehicles (pav).

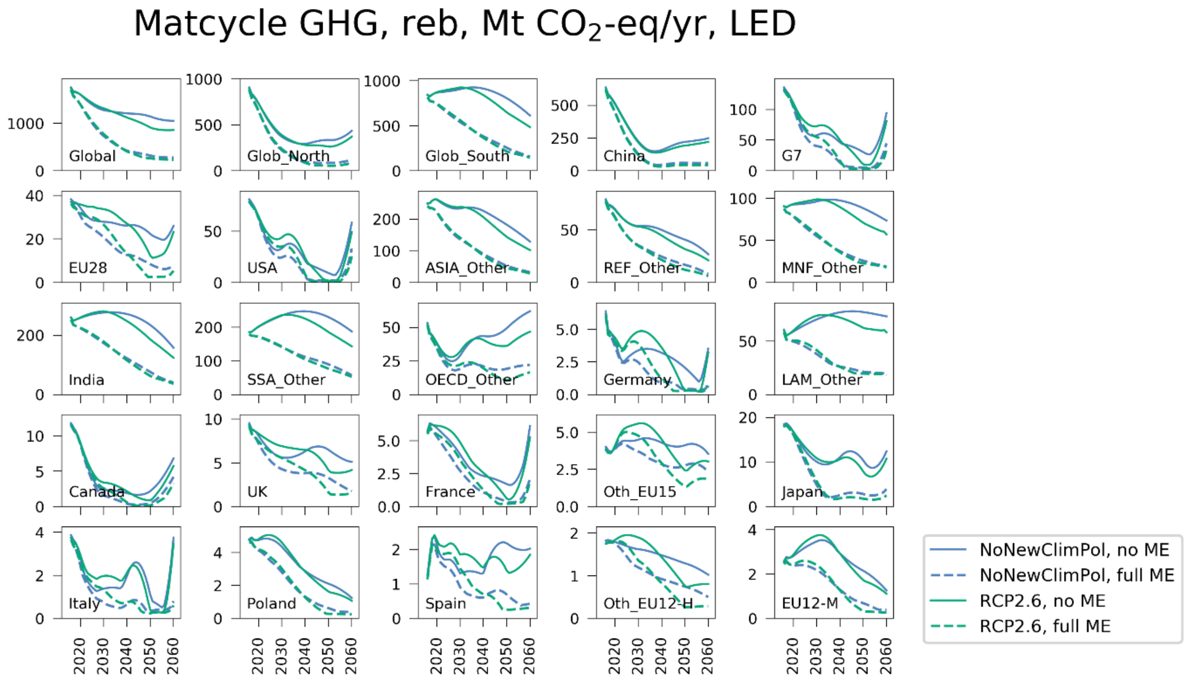

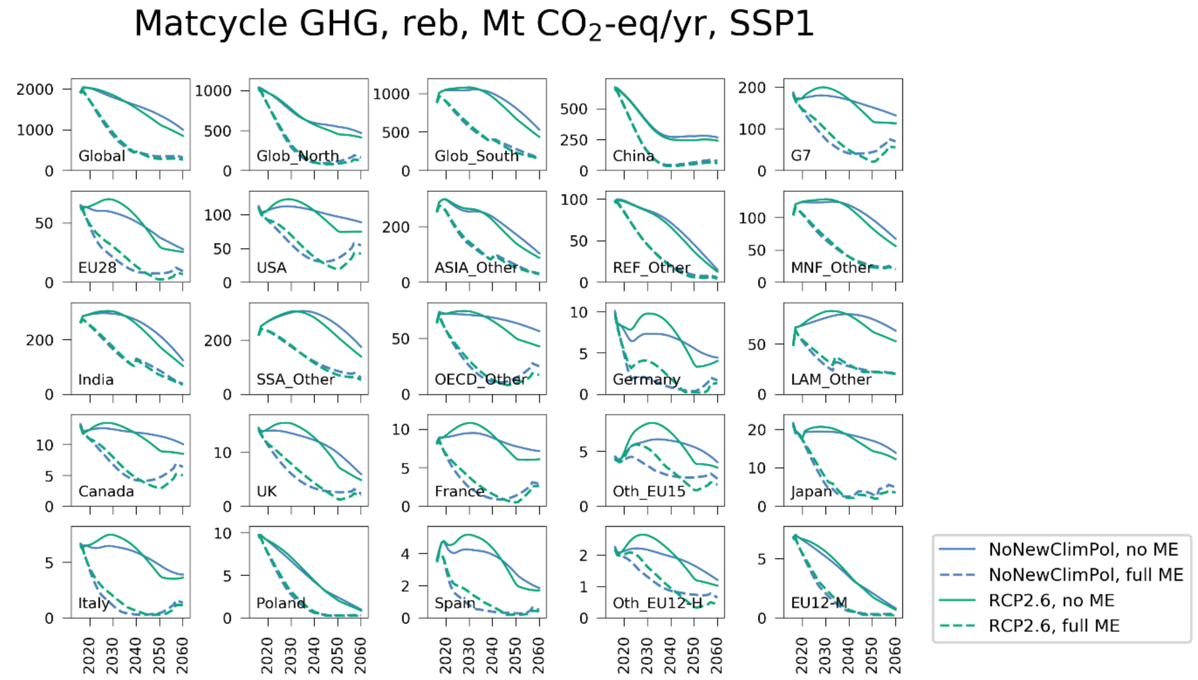

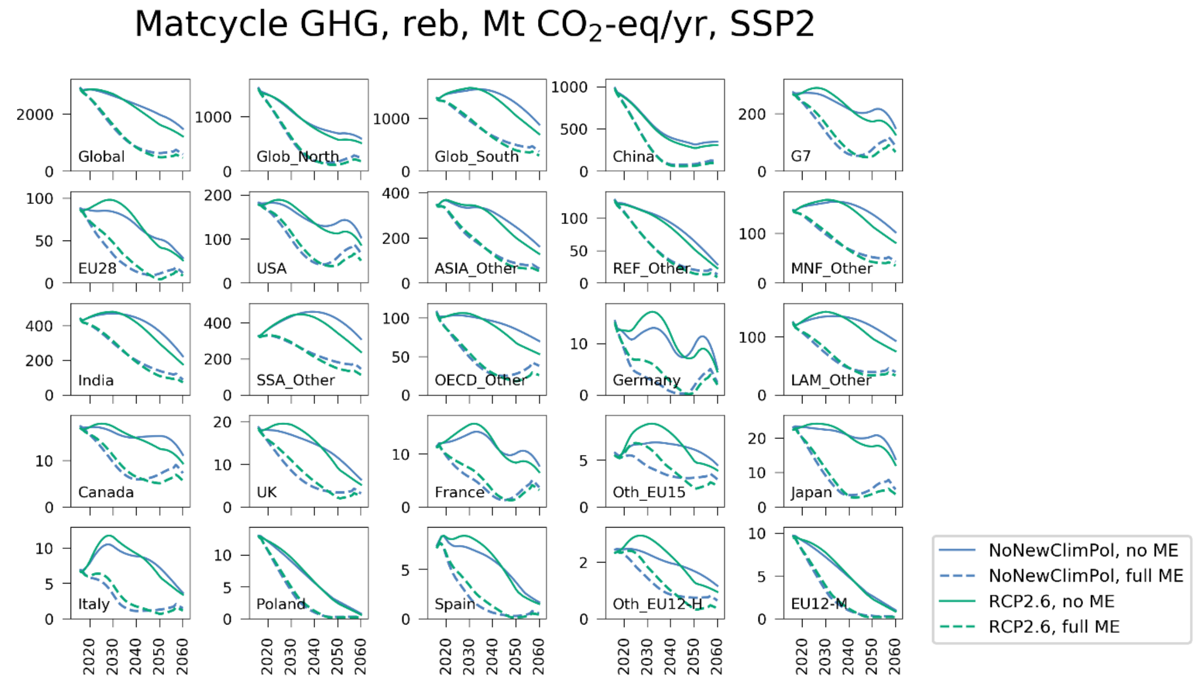

Supplementary Figure 13: Material cycle GHG emissions (for all materials combined), by region and climate policy/RES scenario, for residential buildings (reb).

5.5. Impact of ME on primary and secondary material production over time

Total primary material, pav, Mt/yr, LED

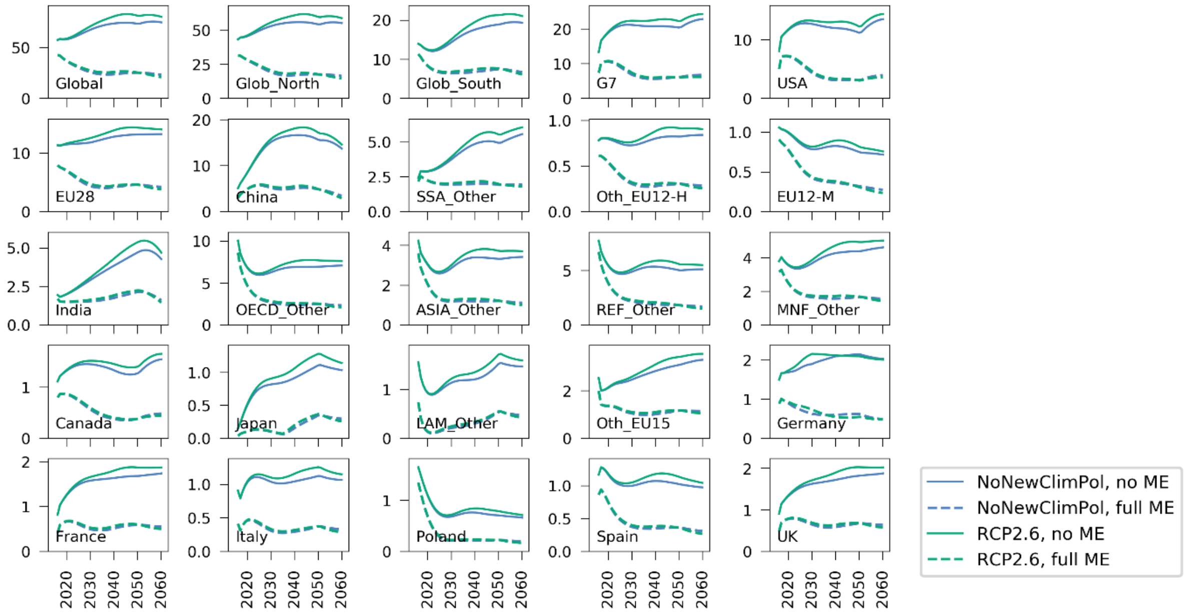

Total primary material, pav, Mt/yr, SSP1

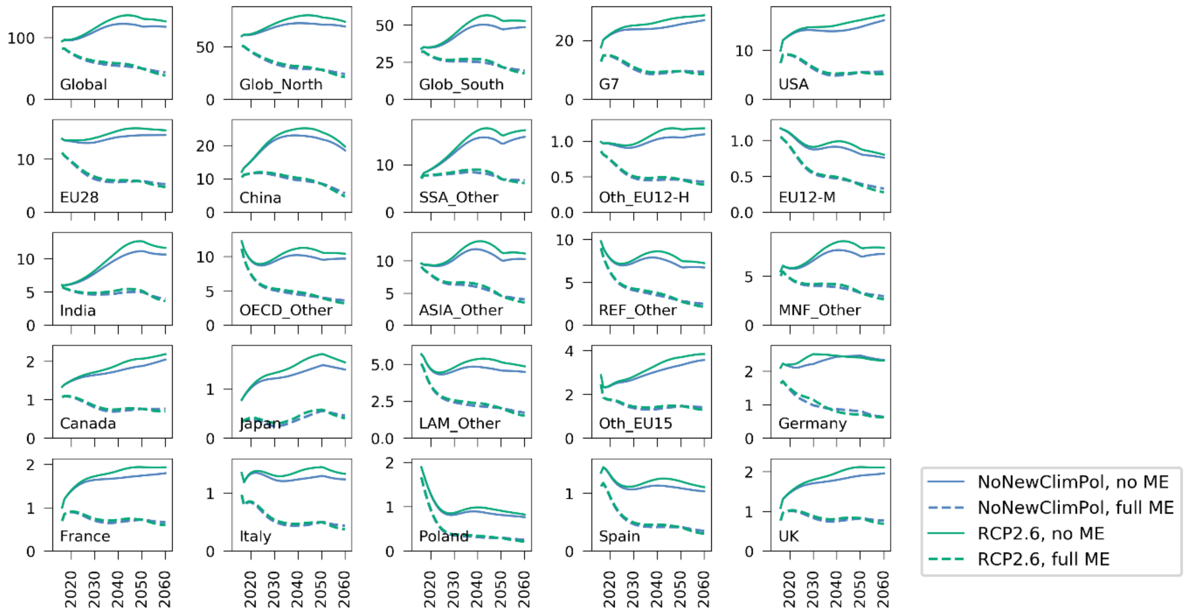

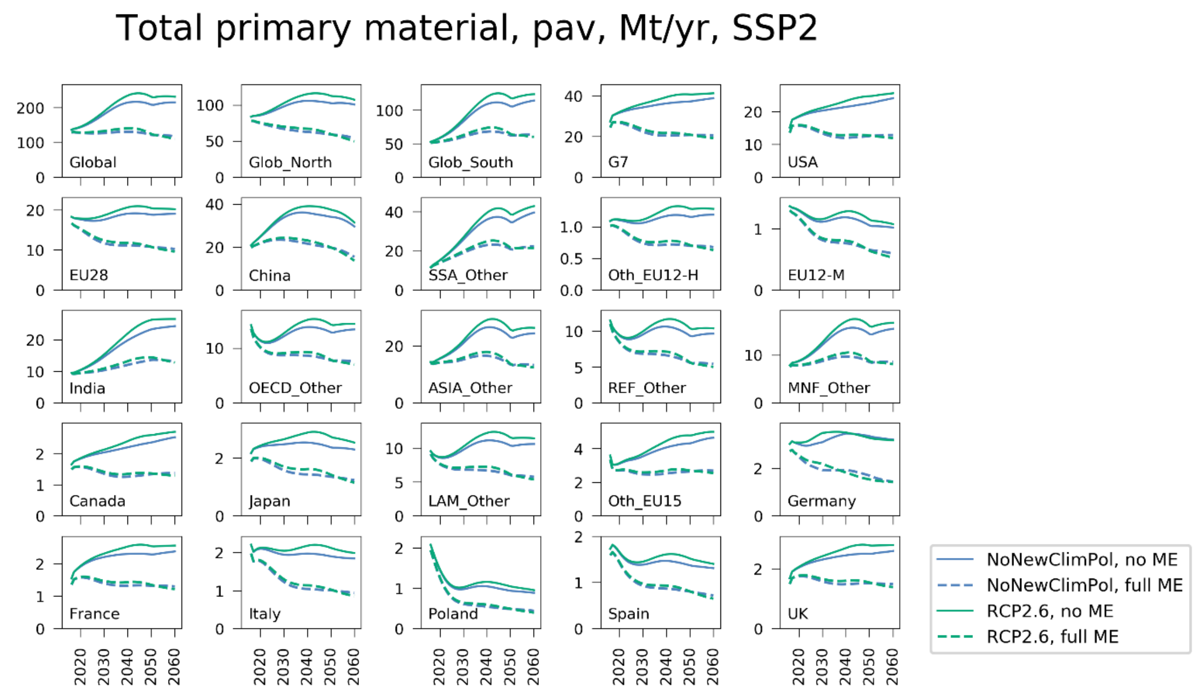

Supplementary Figure 14: Total primary material (all materials added up), by region and climate policy/RES scenario, for passenger vehicles (pav).

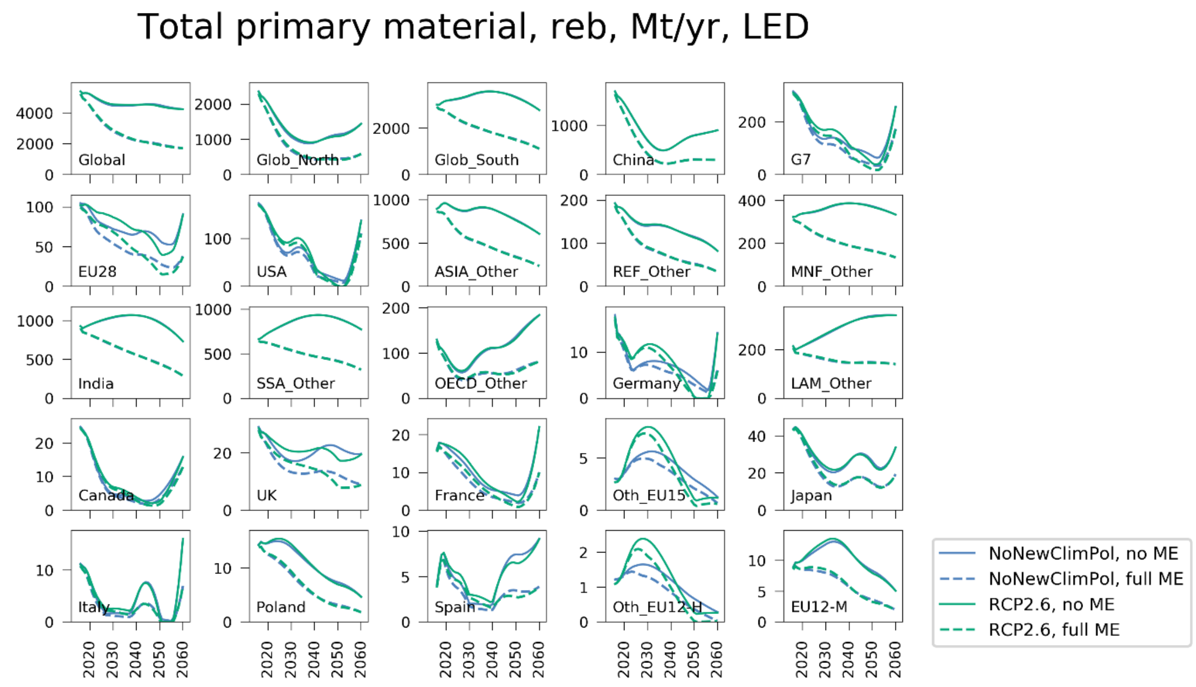

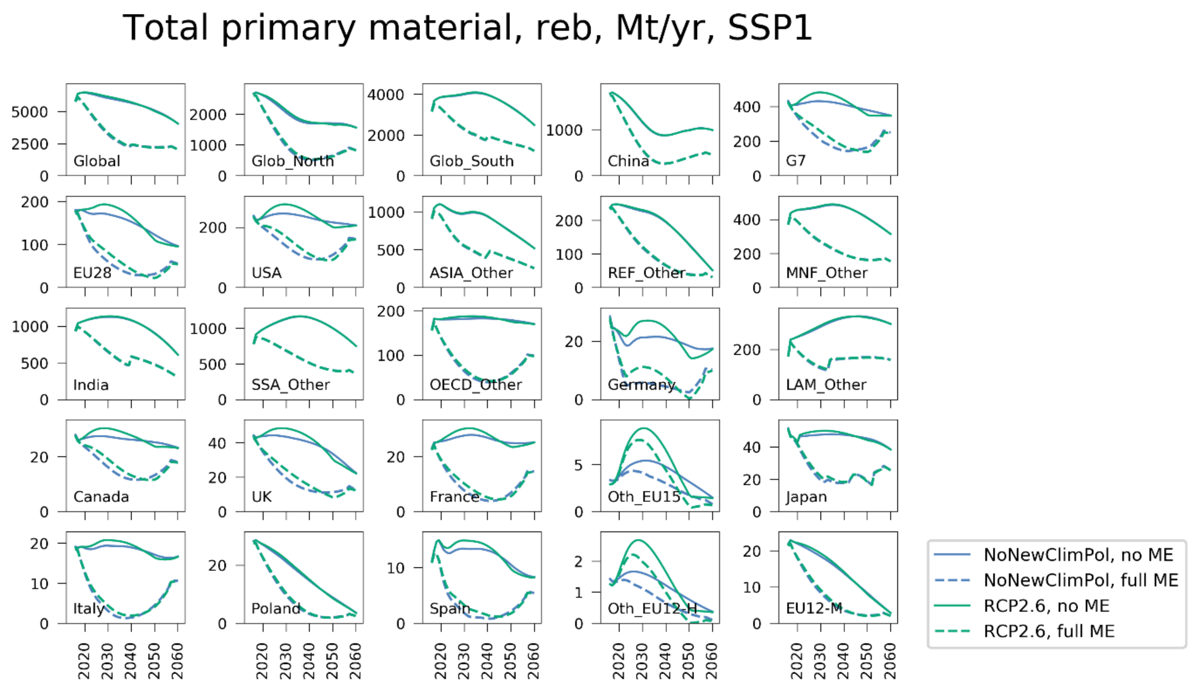

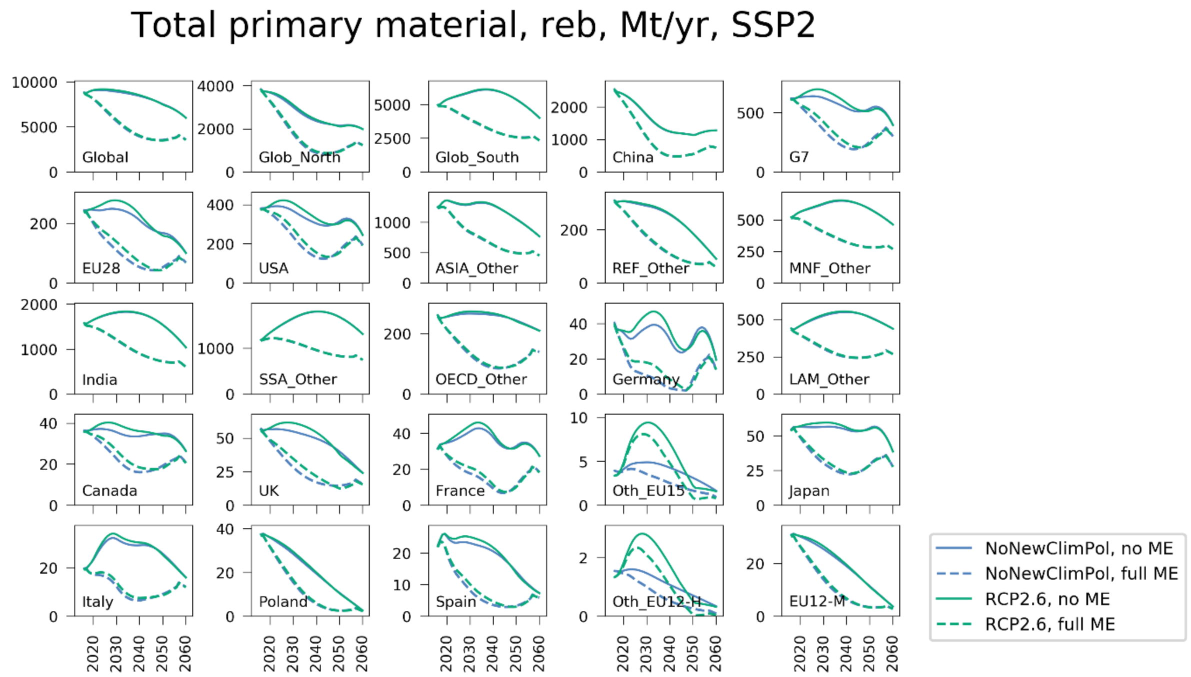

Supplementary Figure 15: Total primary material (all materials added up), by region and climate policy/RES scenario, for residential buildings (reb).

Total secondary material, pav, Mt/yr, LED

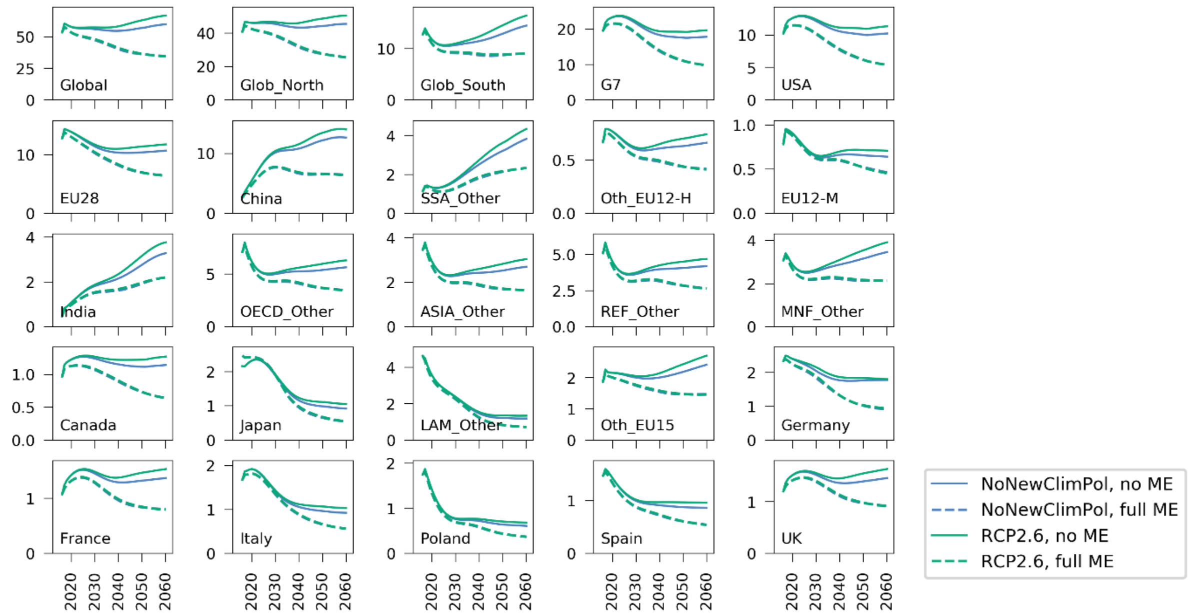

Total secondary material, pav, Mt/yr, SSP1

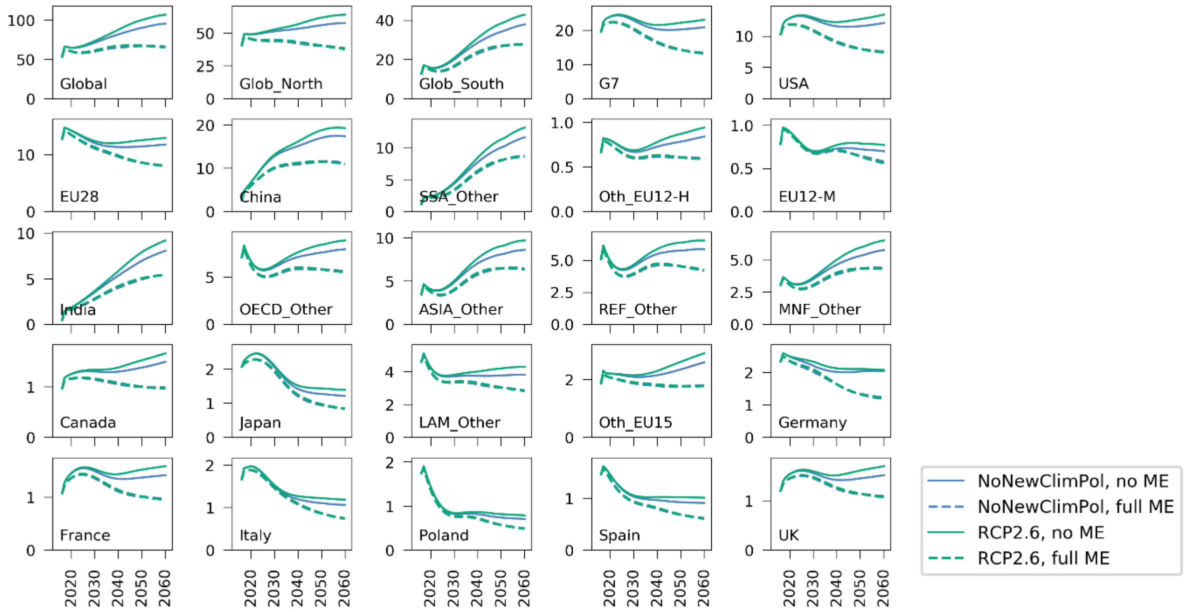

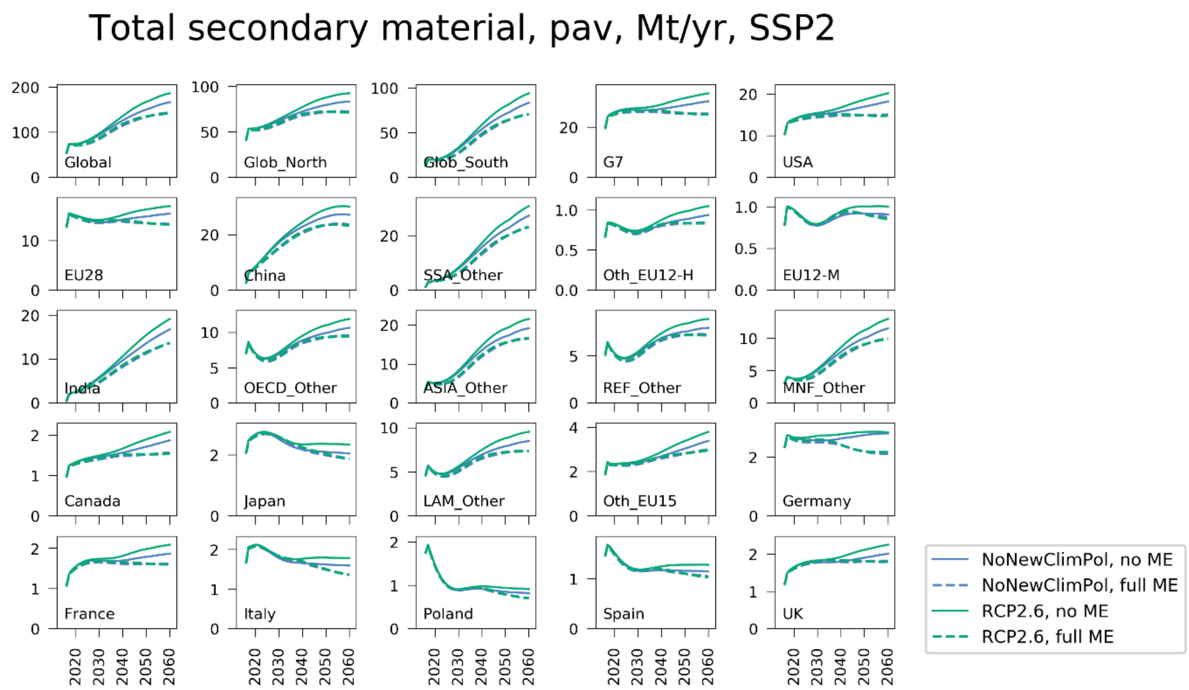

Supplementary Figure 16: Total secondary material (all materials added up), by region and climate policy/RES scenario, for passenger vehicles (pav).

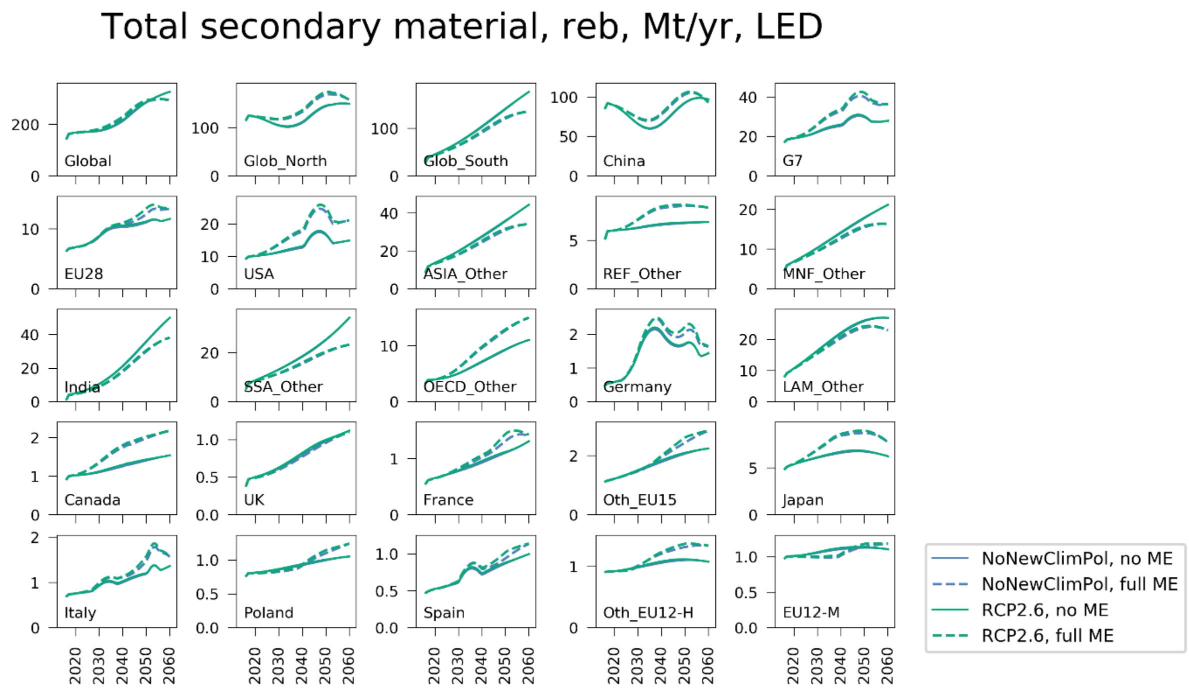

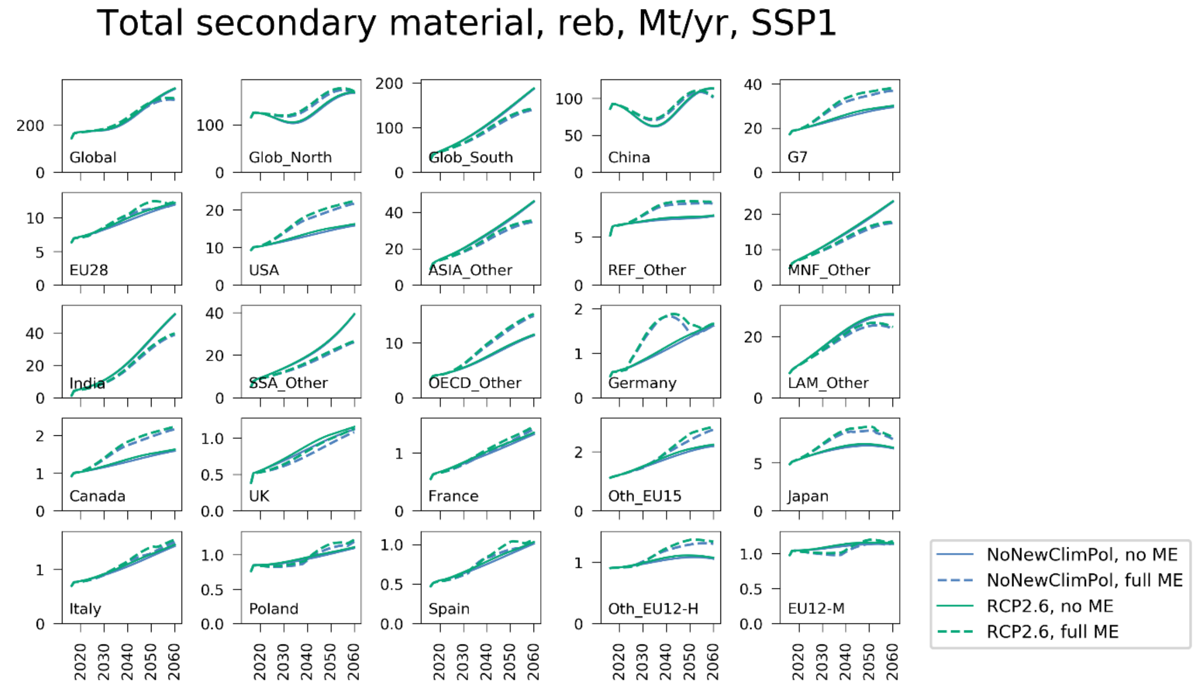

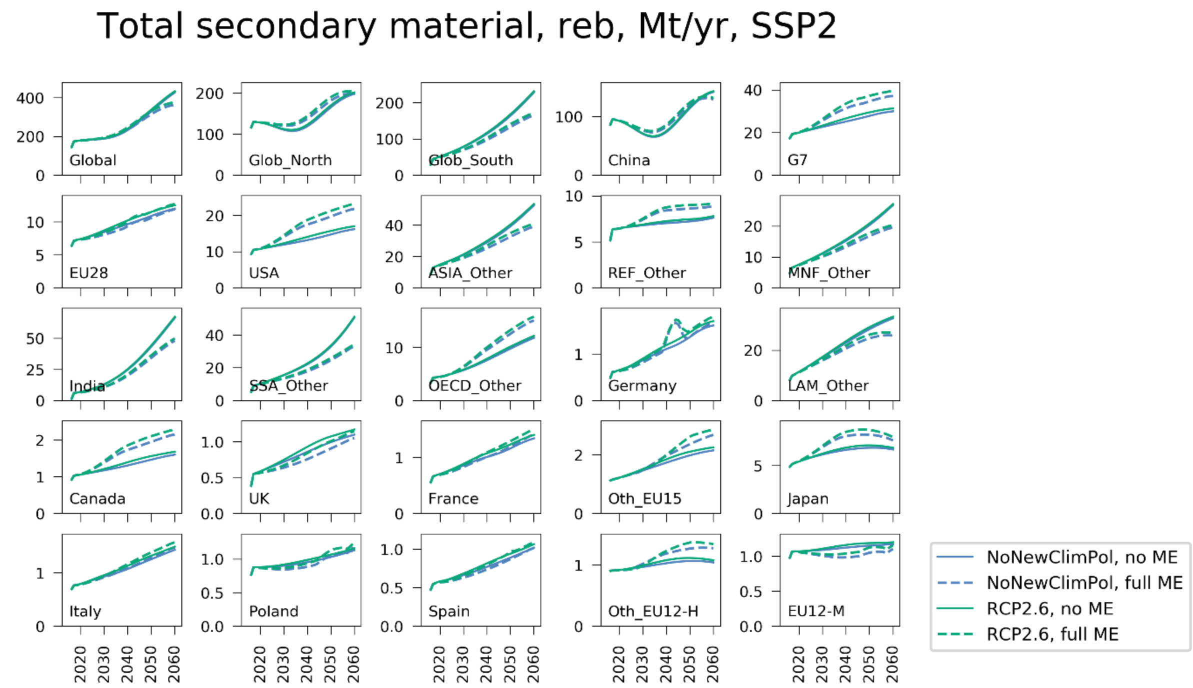

Supplementary Figure 17: Total secondary material (all materials added up), by region and climate policy/RES scenario, for residential buildings (reb).

5.6. Impact of ME on use phase energy consumption (final energy) over time

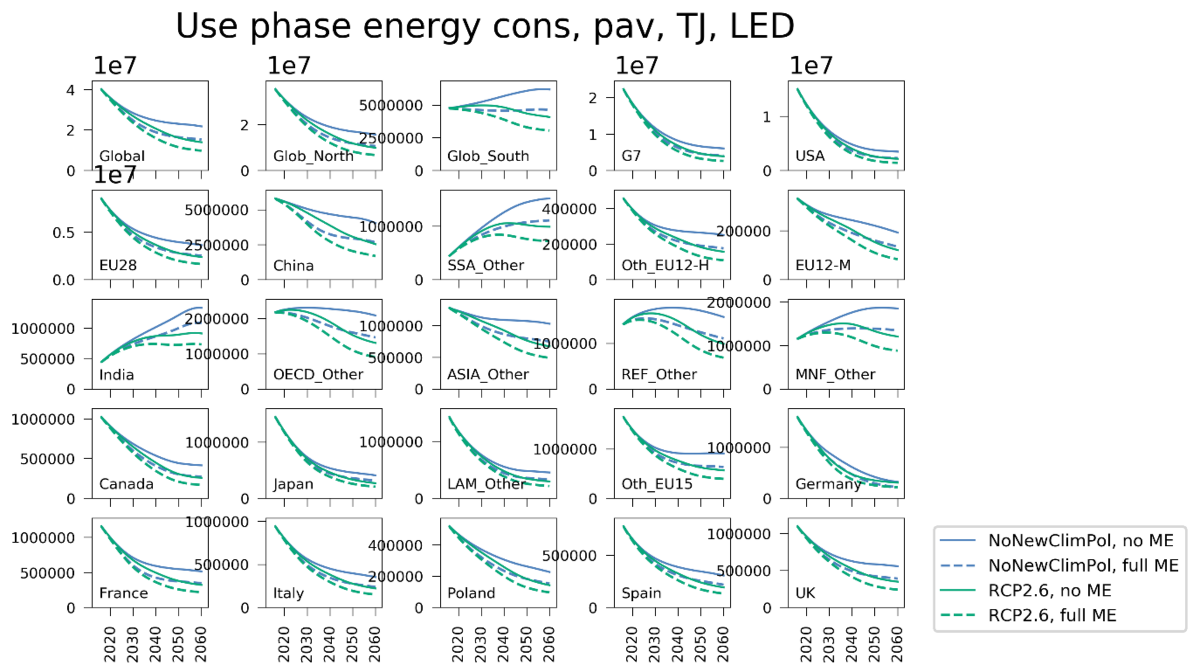

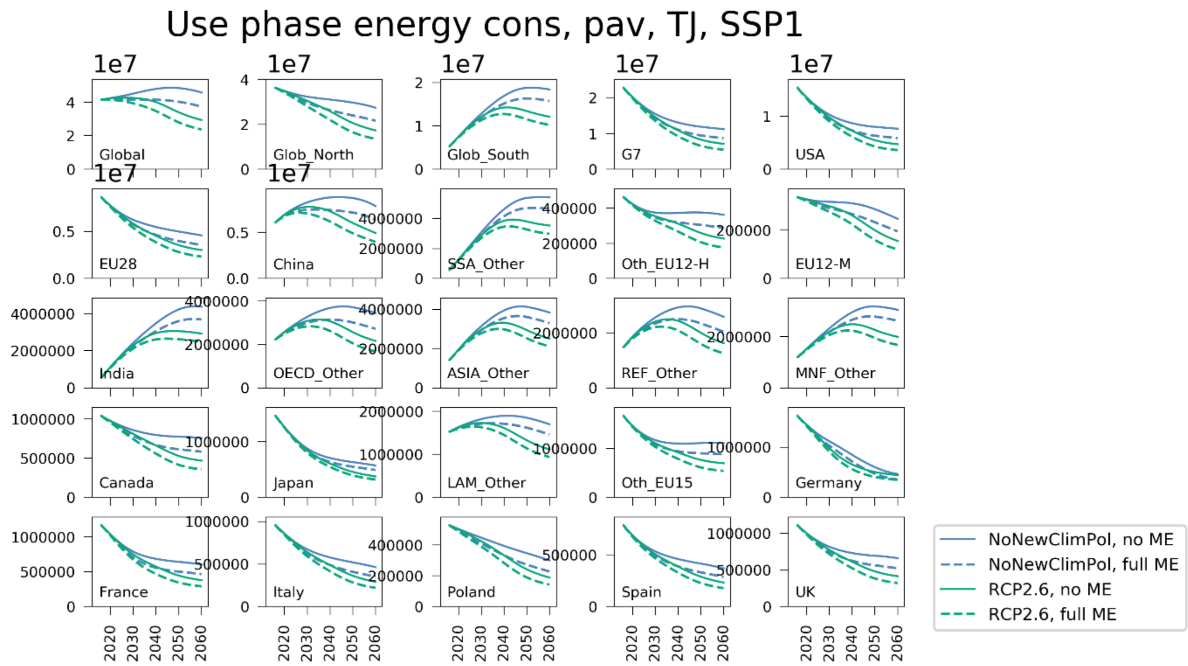

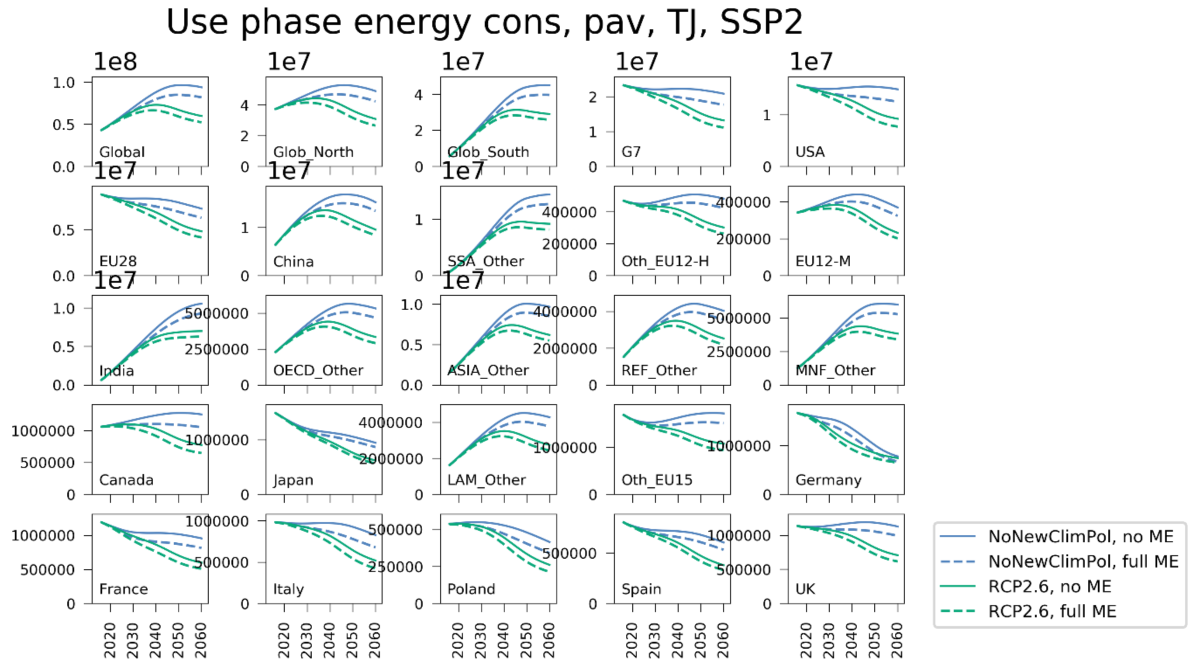

Supplementary Figure 18: Use phase energy consumption (final energy), by region and climate policy/RES scenario, for passenger vehicles (pav).

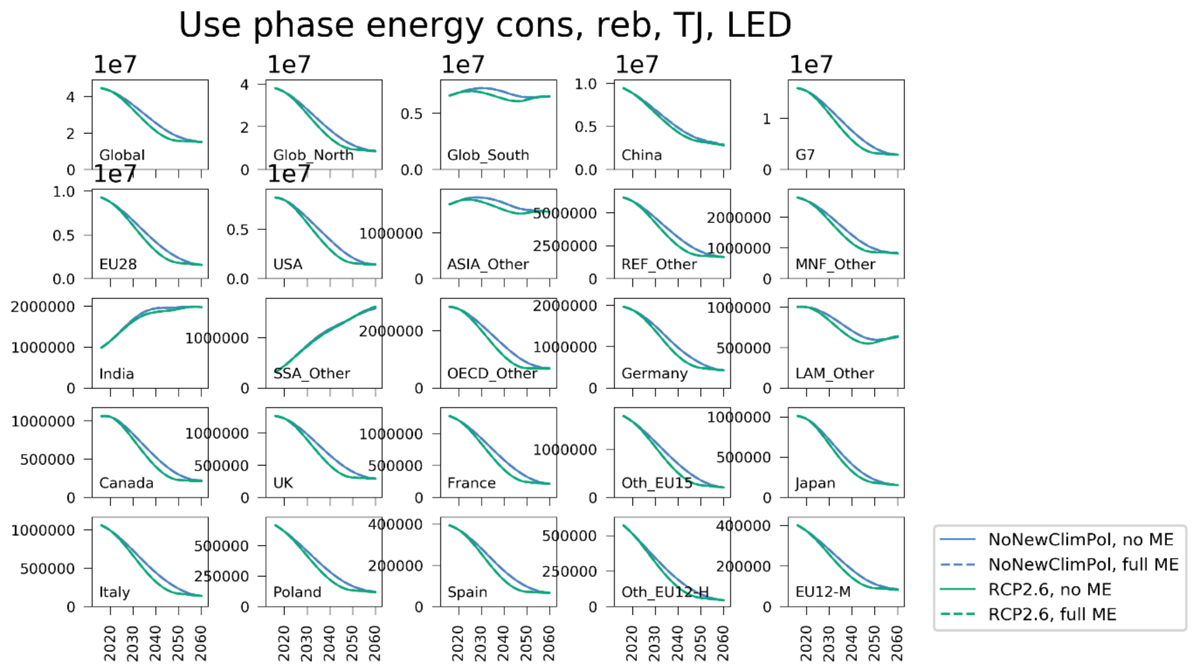

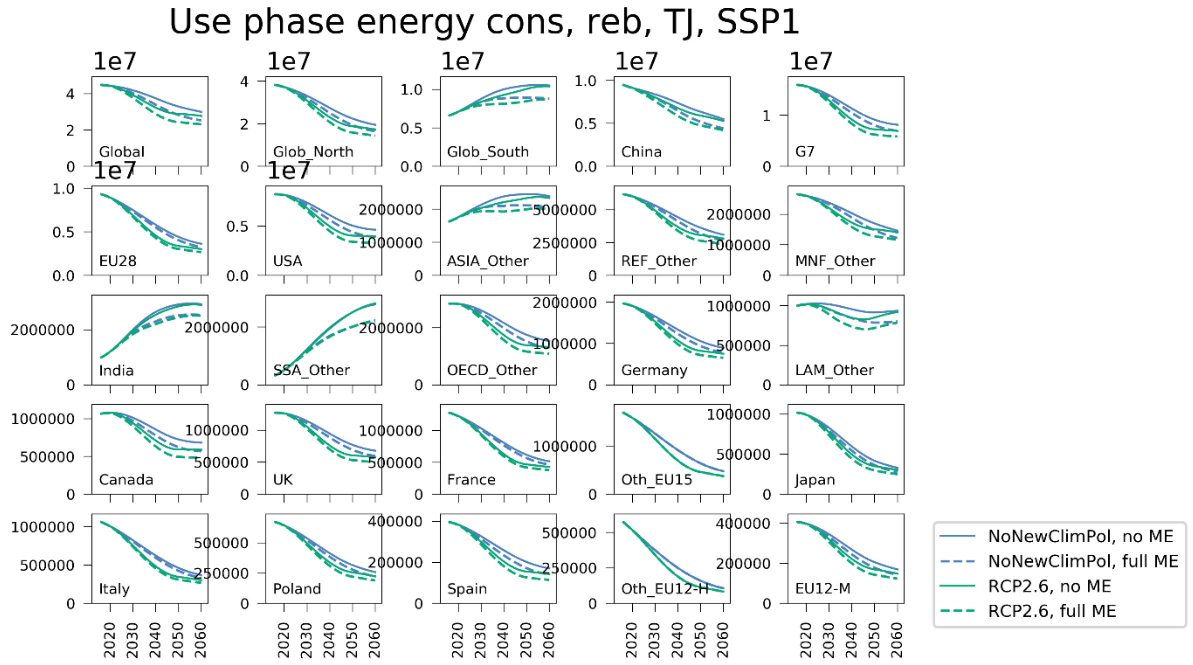

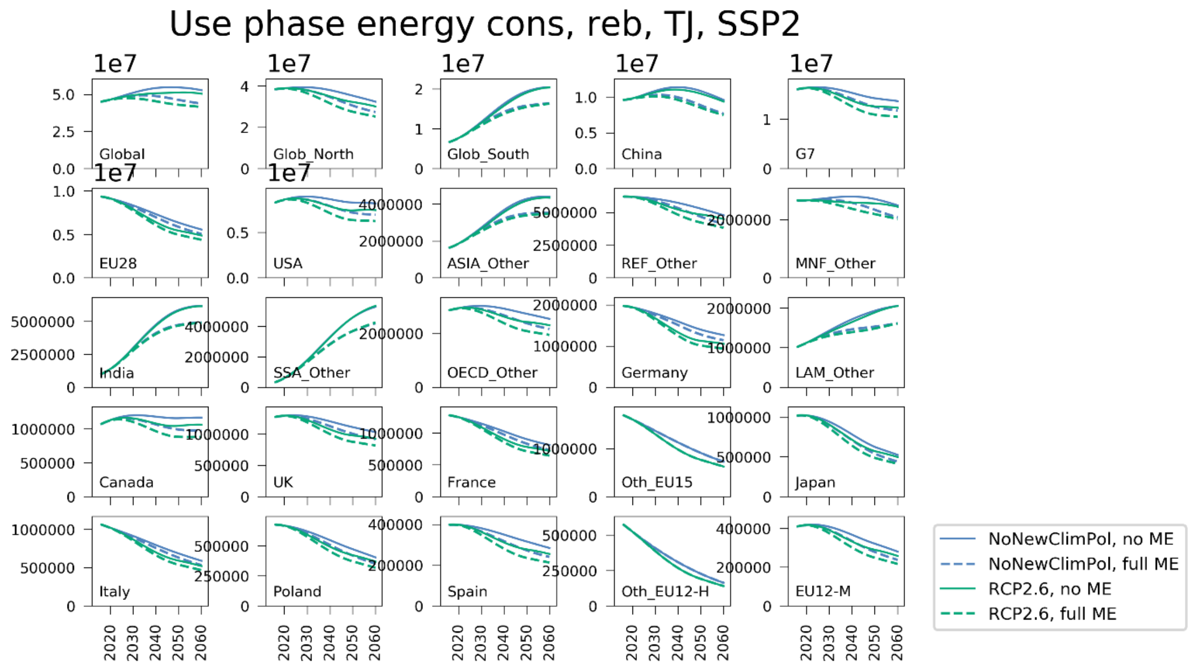

Supplementary Figure 19: Use phase energy consumption (final energy), by region and climate policy/RES scenario, for residential buildings (reb).

5.7. Share of electricity and hydrogen in use phase energy consumption over time

Share of El and H_2 in use phase en. cons, pav, 1, LED

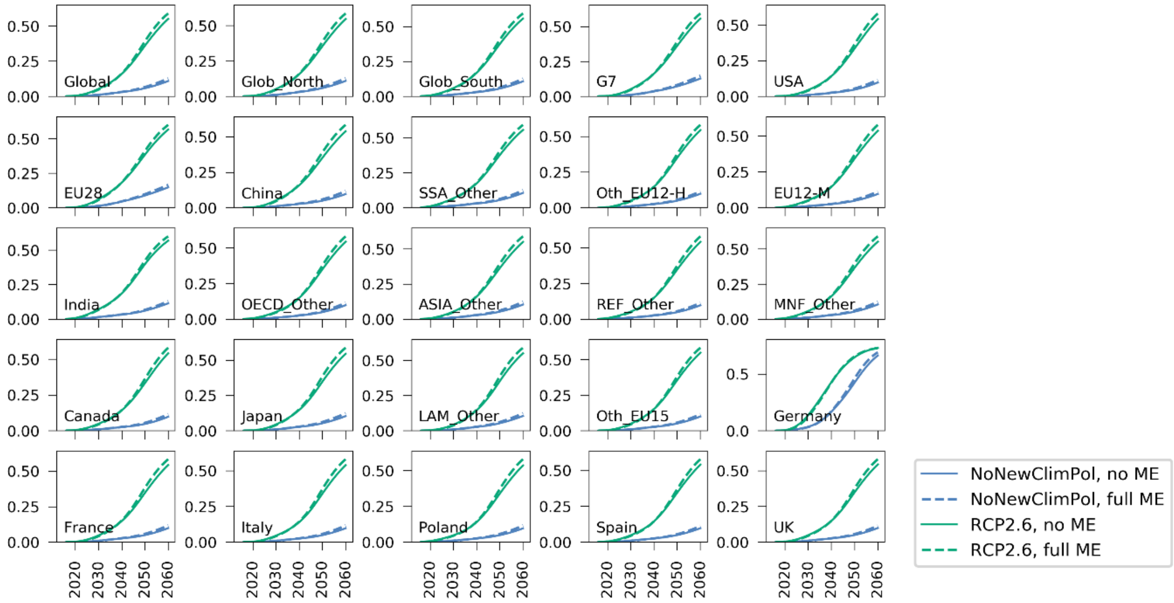

Share of El and H_2 in use phase en. cons, pav, 1, SSP1

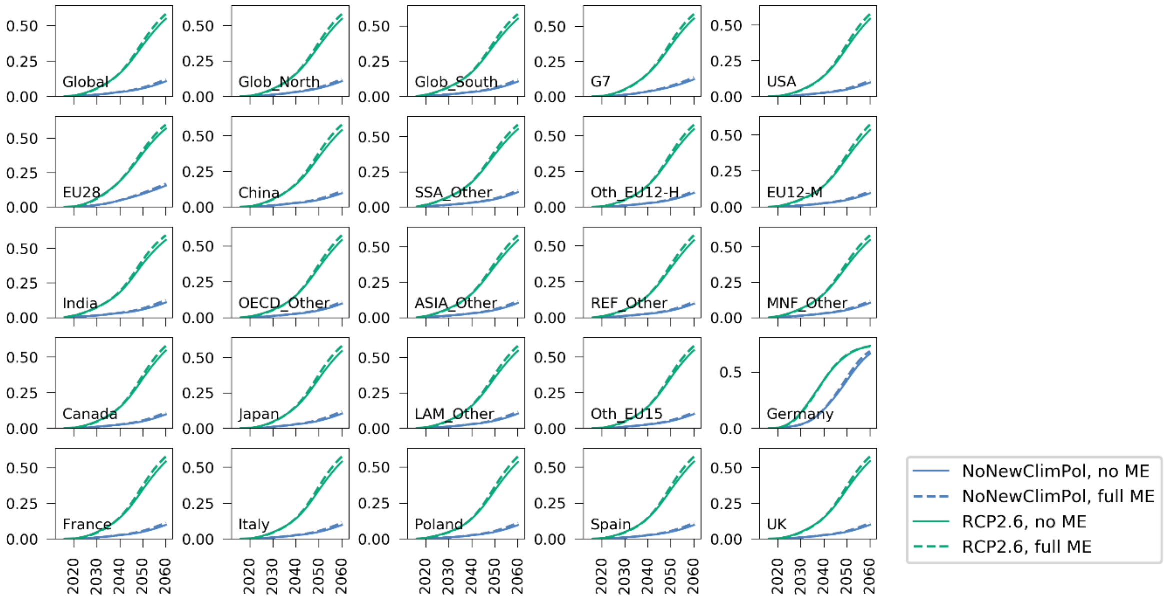

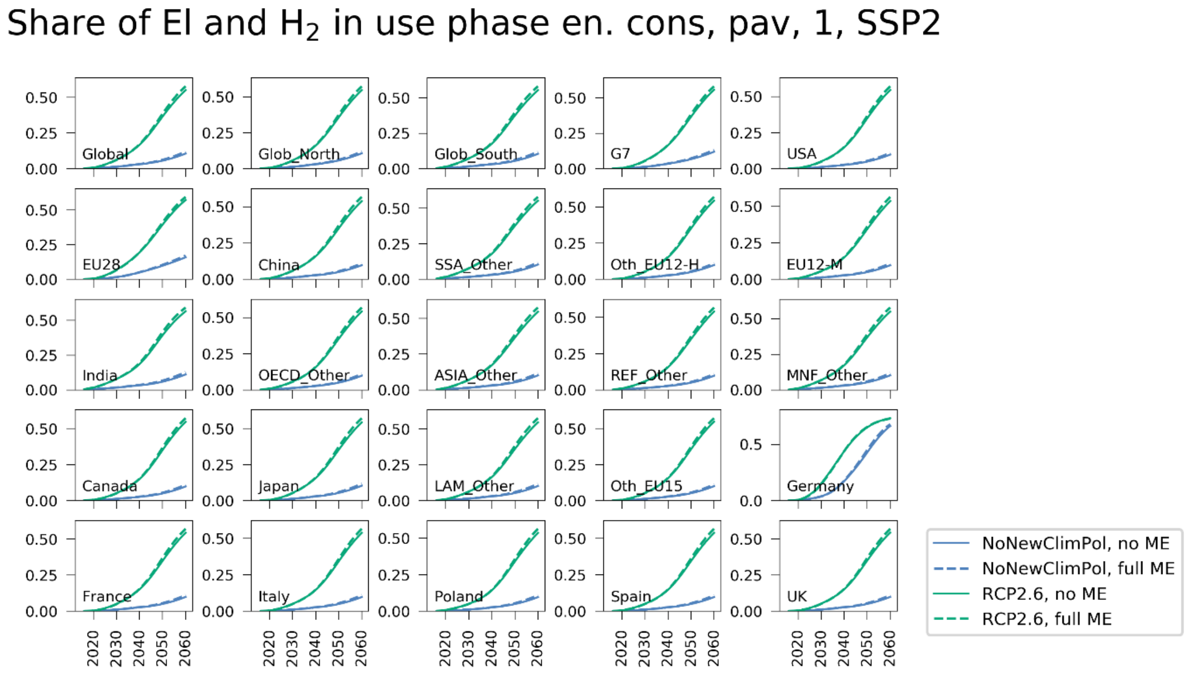

Supplementary Figure 20: Use phase share of electricity and H2 in total energy consumption, by region and climate policy/RES scenario, for passenger vehicles (pav).

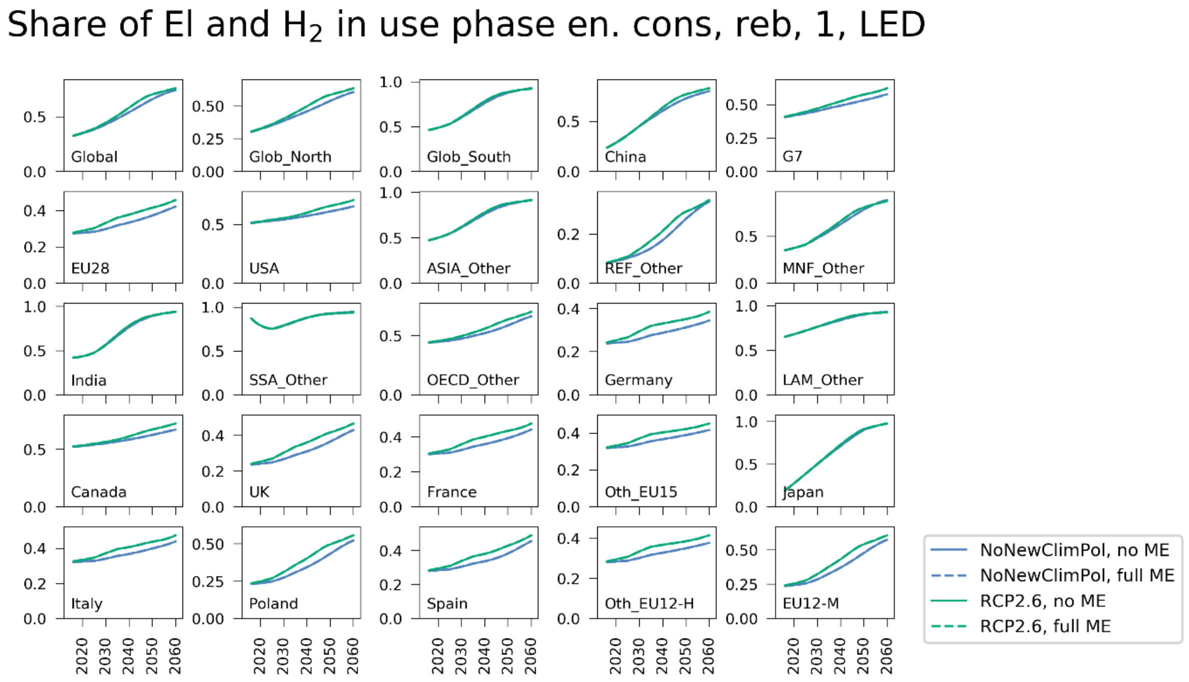

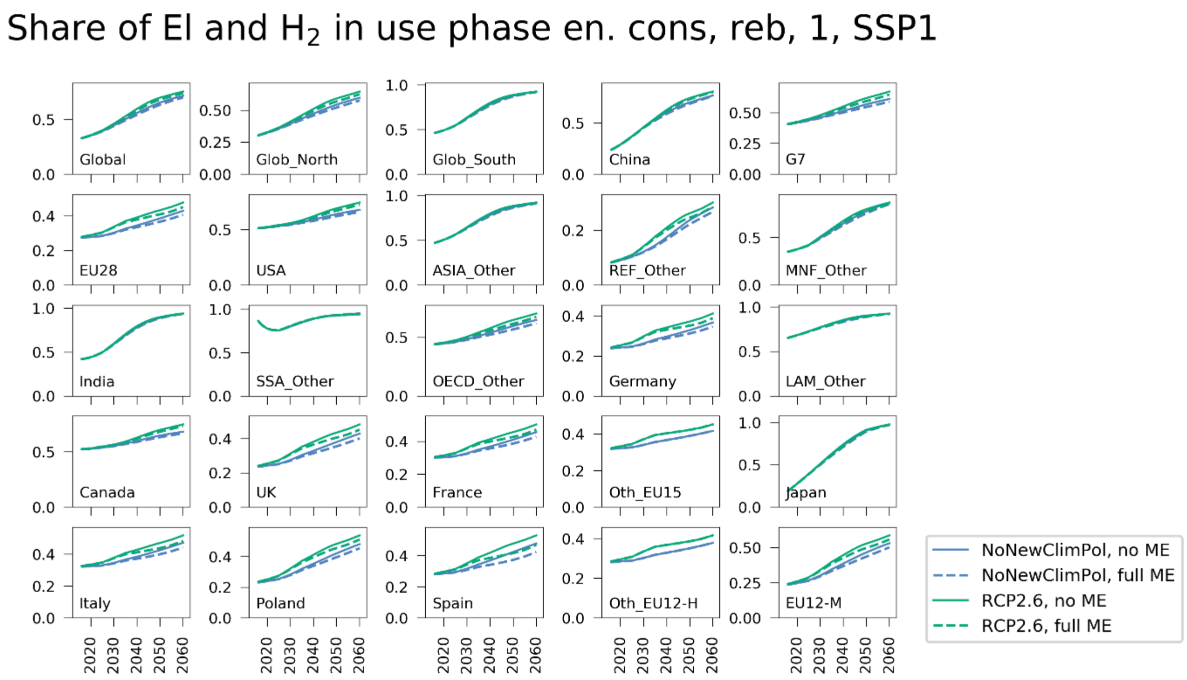

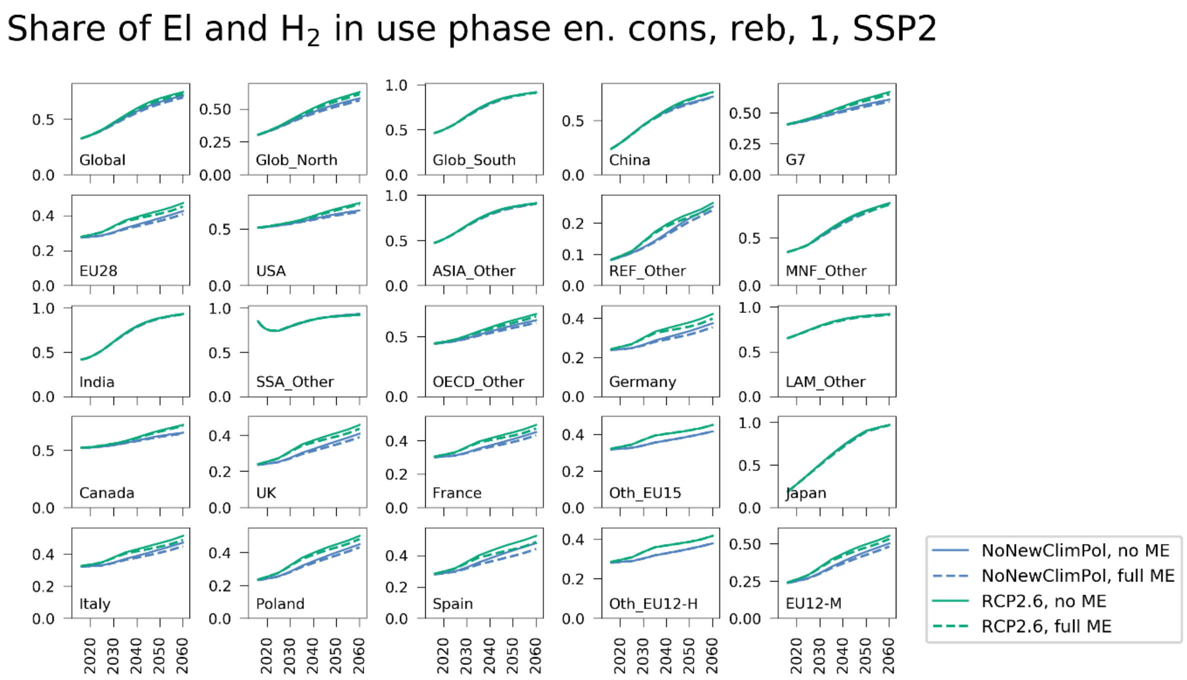

Supplementary Figure 21: Use phase share of electricity and H2 in total energy consumption, by region and climate policy/RES scenario, for residential buildings (reb).

5.8. Passenger-km are delivered over time

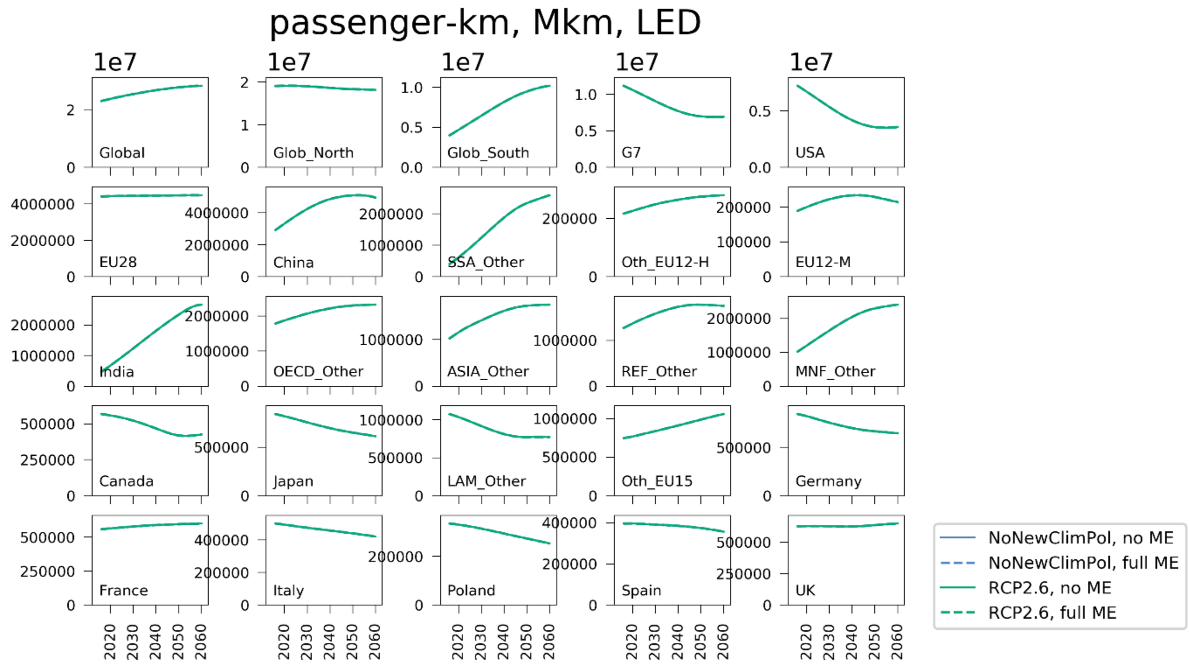

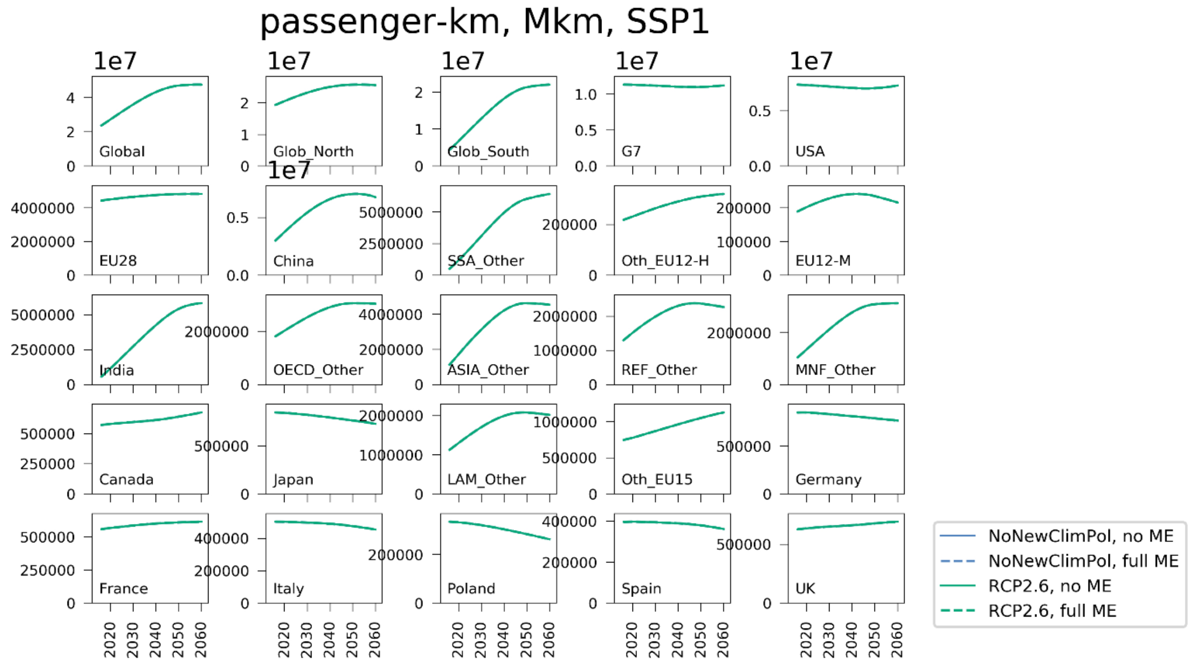

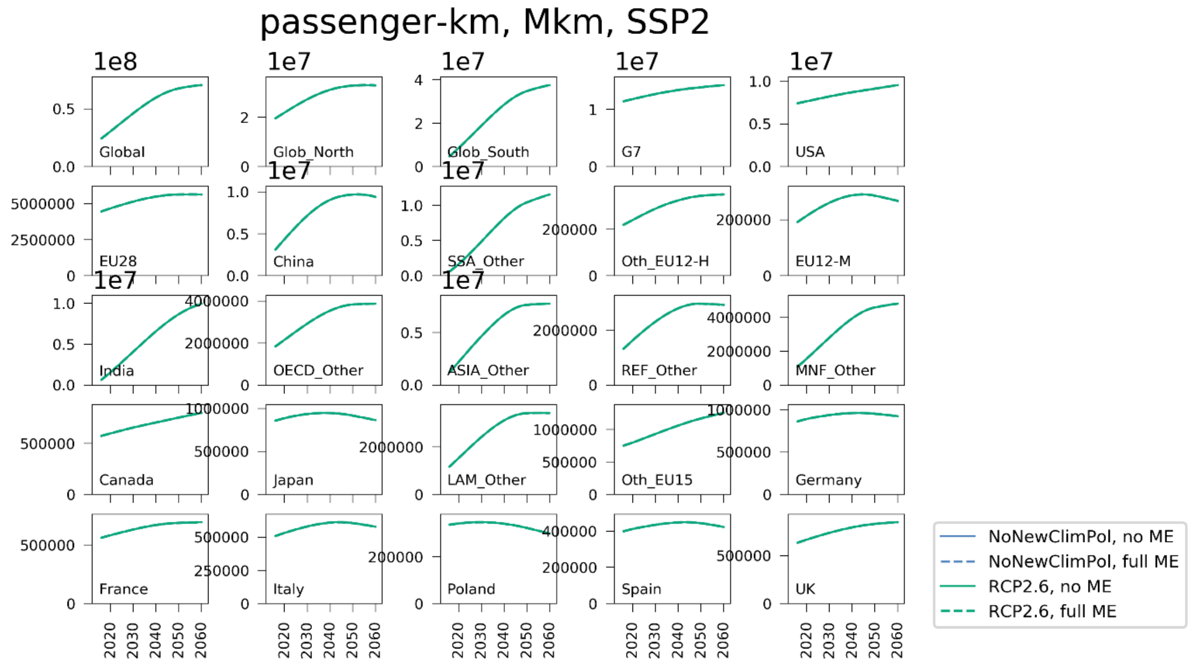

Supplementary Figure 22: passenger-km delivered (scenario driver), by region and climate policy/RES scenario, for passenger vehicles (pav).

5.9. 'Service efficiency', defined in passenger-km per total GHG and per total material stocks, over time

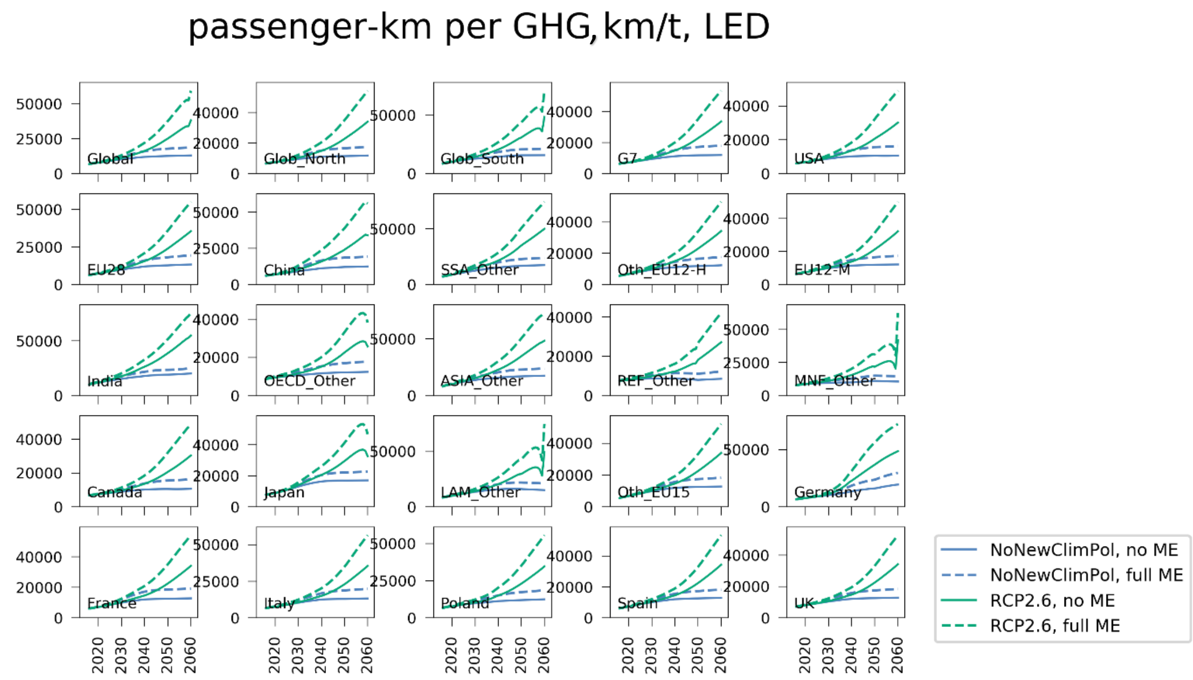

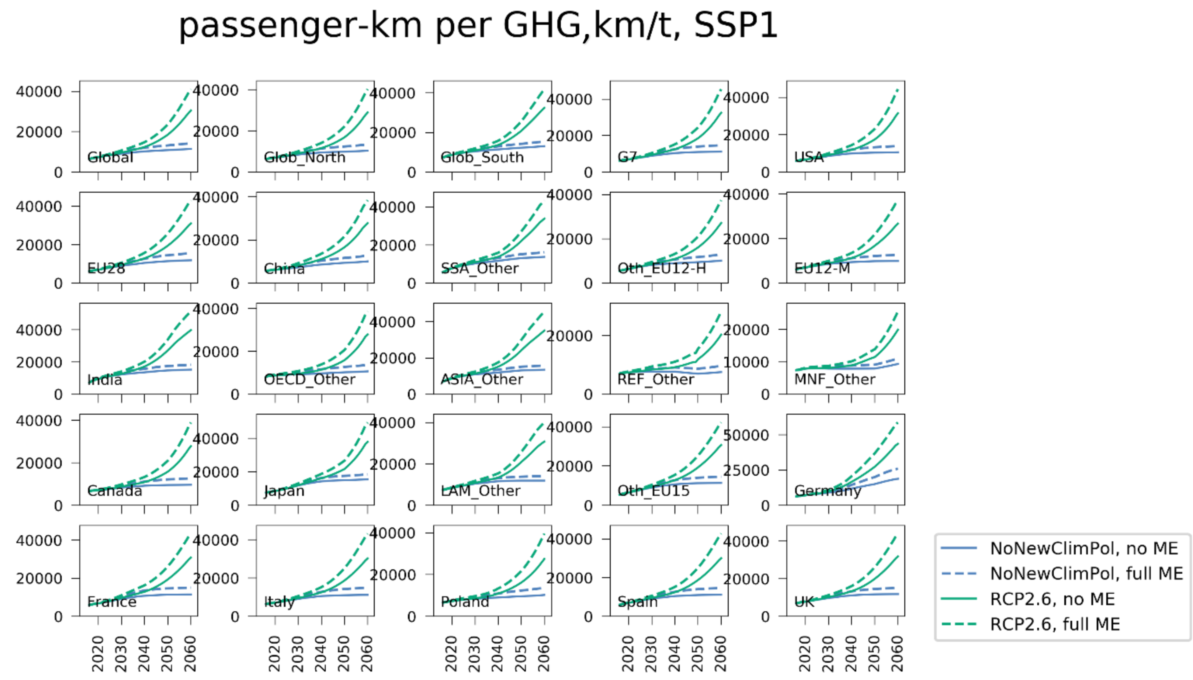

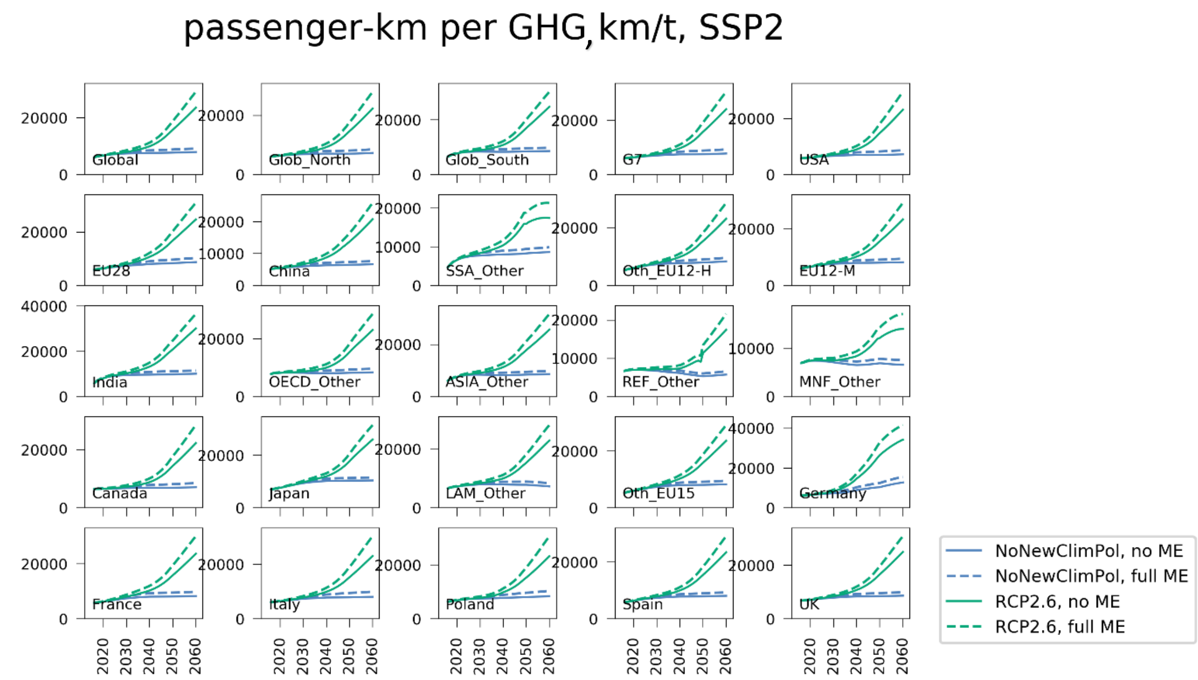

Supplementary Figure 23: passenger-km delivered (scenario driver) per system-wide GHG, by region and climate policy/RES scenario, for passenger vehices (pav).

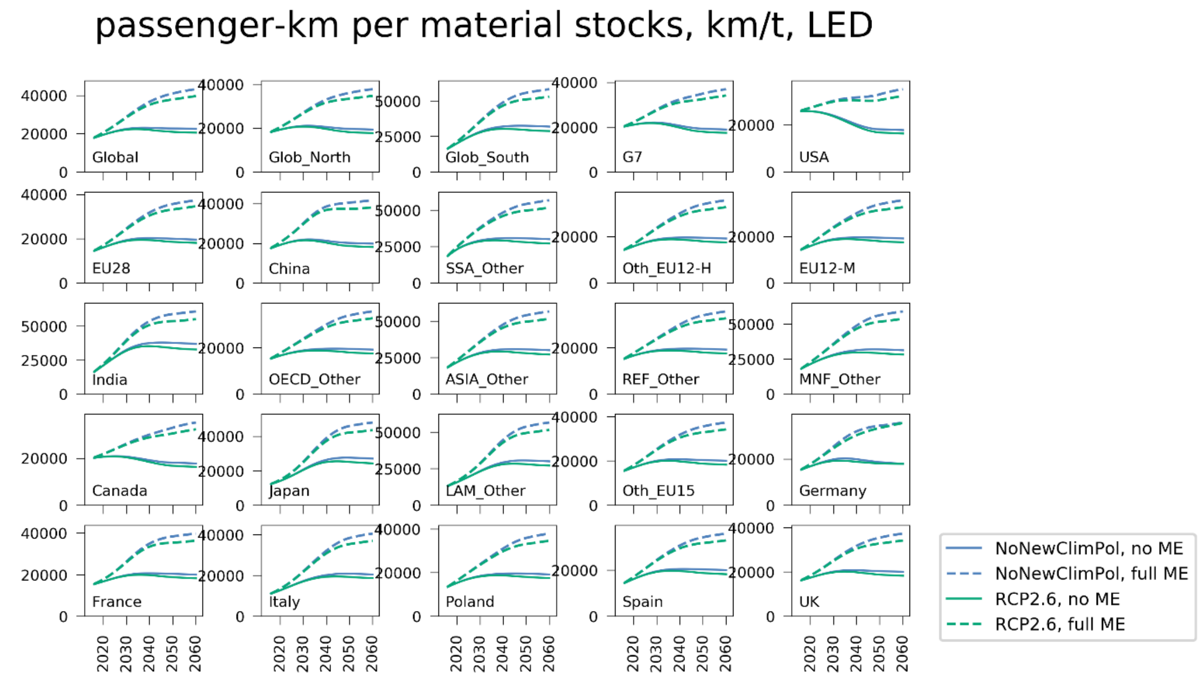

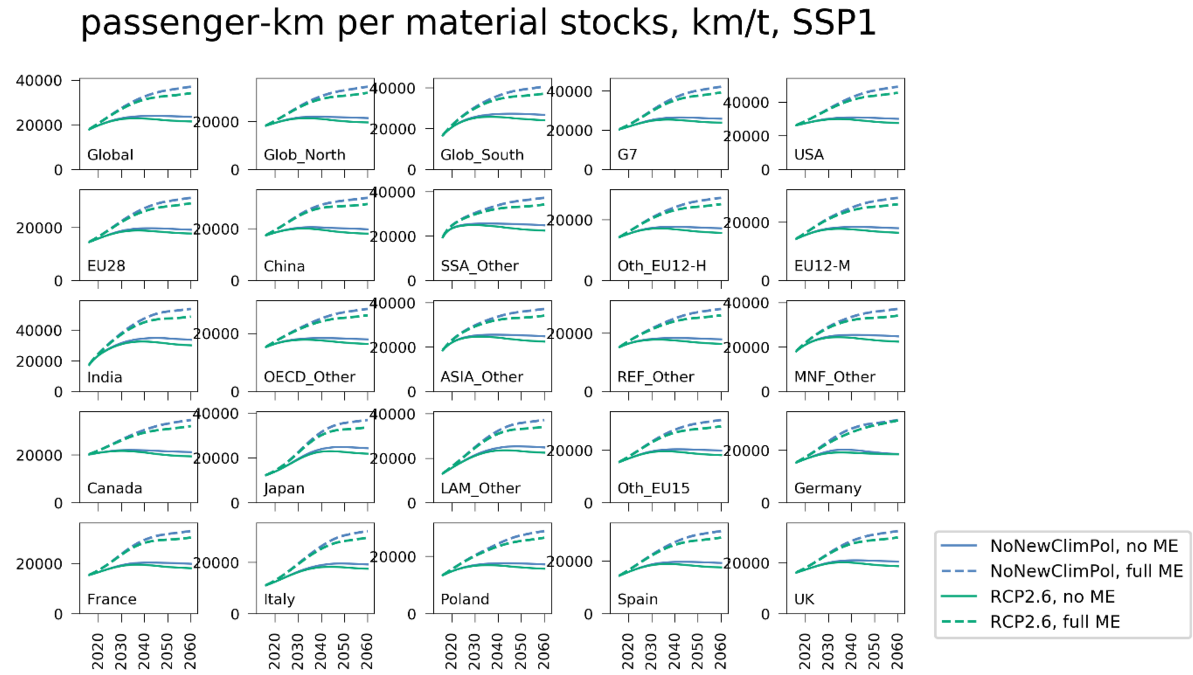

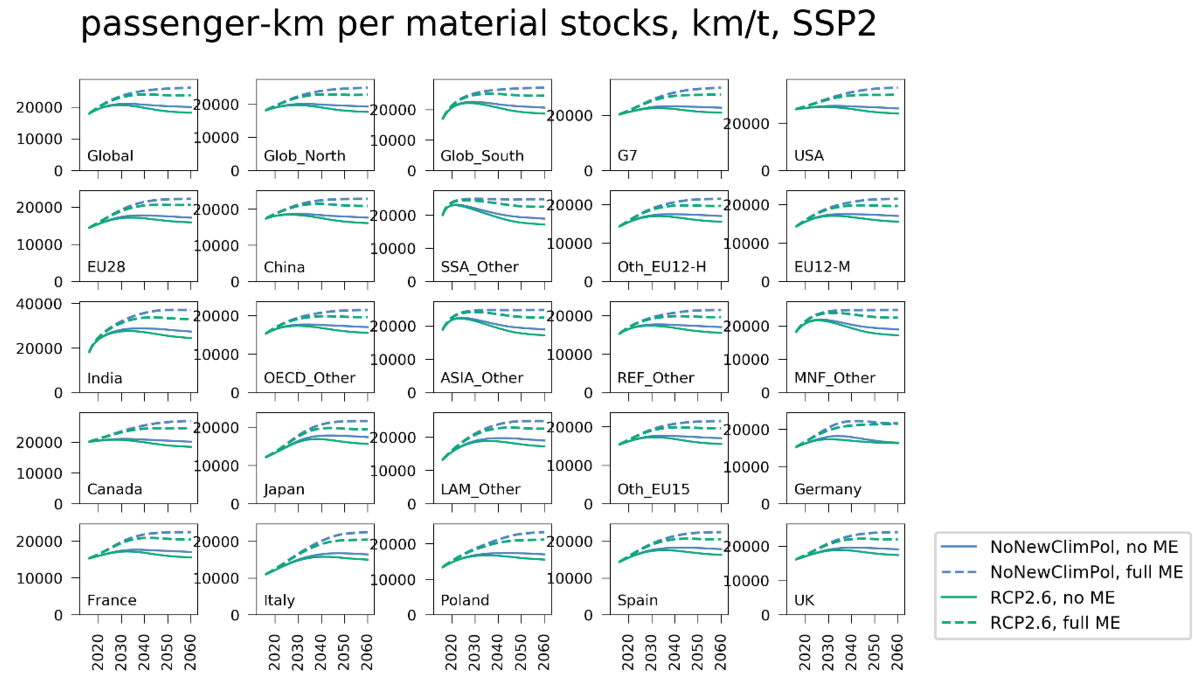

Supplementary Figure 24: passenger-km delivered (scenario driver) per total material stocks, in kilometer per ton (km/t), by region and climate policy/RES scenario, for passenger vehicles (pav).

5.10. Total global cumulative (top) and annual 2050 (bottom) emissions reductions of the technical potential of material efficiency strategies

Passenger vehicles:

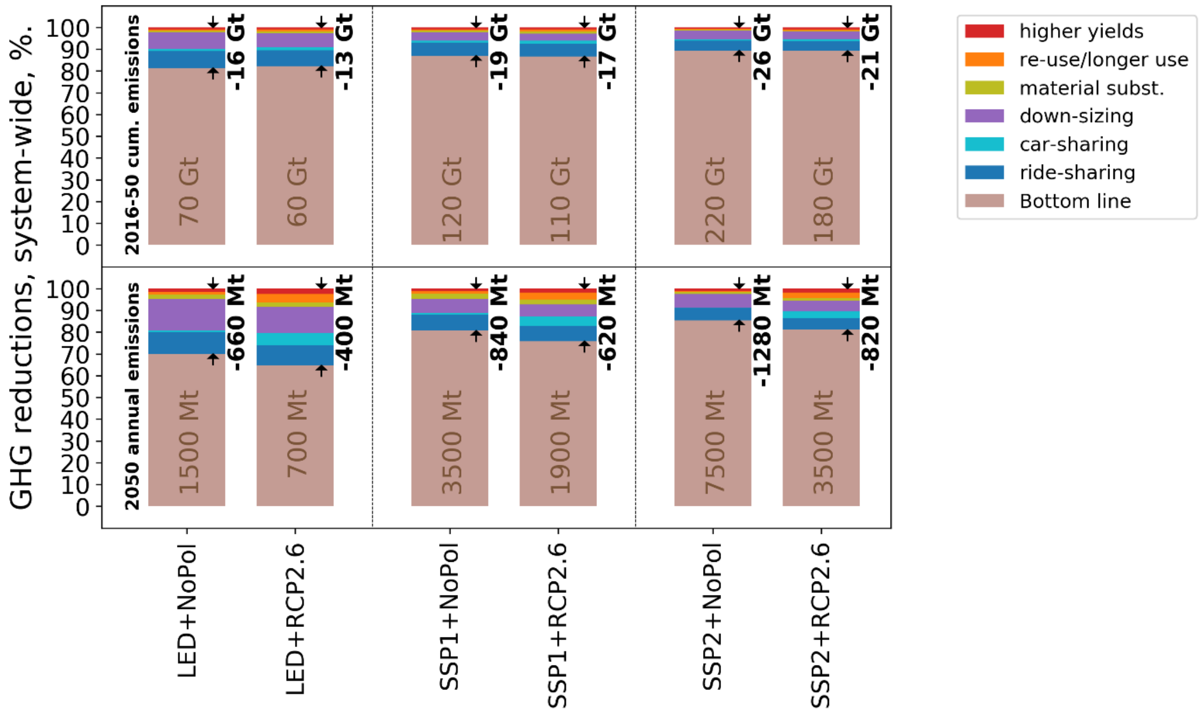

Residential buildings:

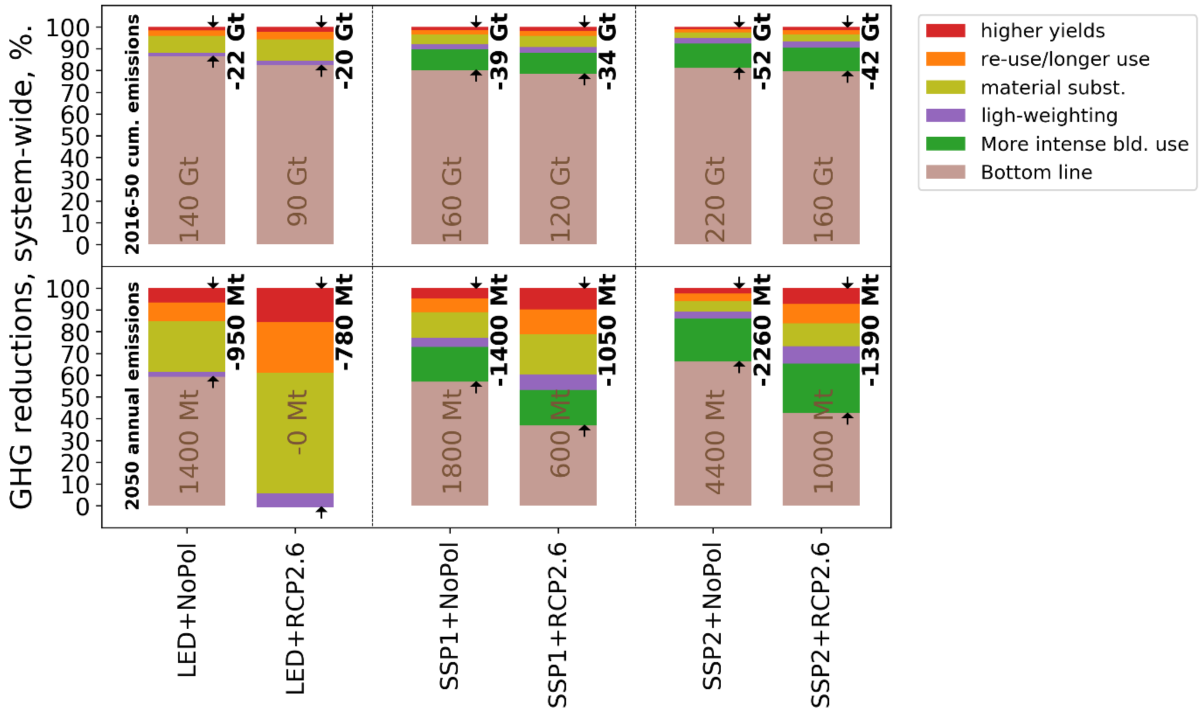

Passenger vehicles and residential buildings (Fig. 2 in the paper):

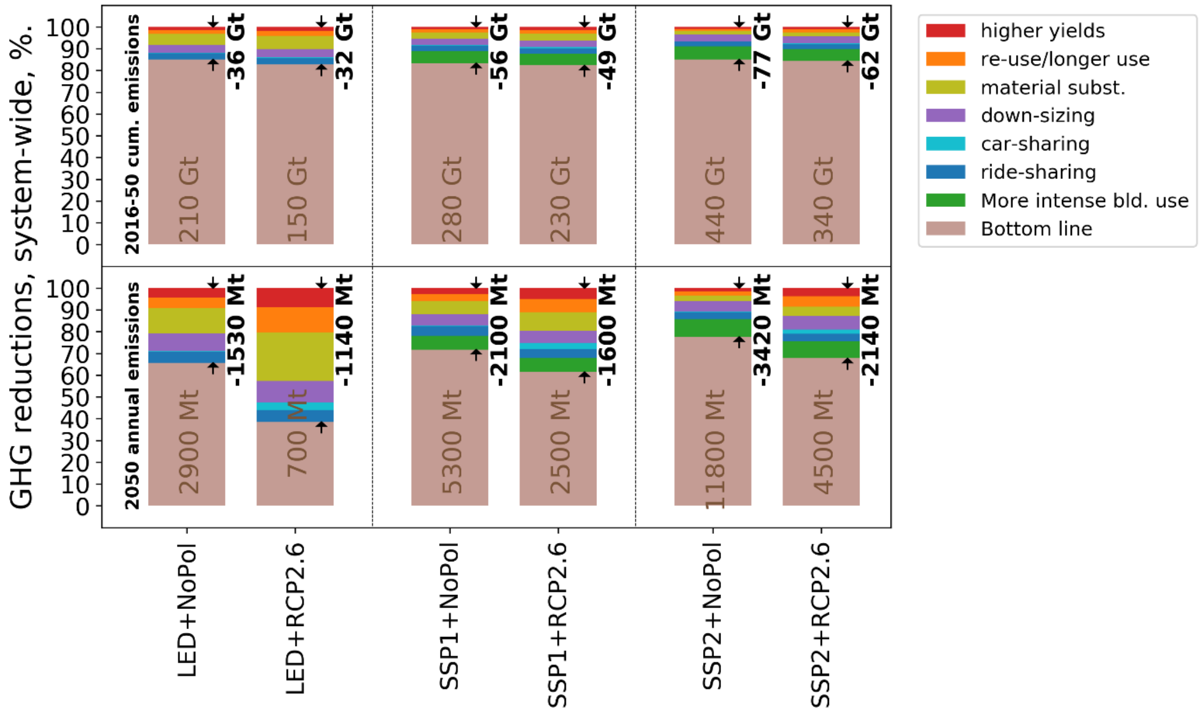

Supplementary Figure 25: Total global cumulative (top row) and annual *2050* (bottom row) emissions reductions of the technical potential of ten supply, demand- and sufficiency-based material efficiency strategies, by socioeconomic and climate policy scenario and ME strategy for the passenger vehicle (pav) and residential building (reb) sectors combined. Top: passenger vehicles, middle: residential buildings, bottom: pav and reb together.

Passenger vehicles:

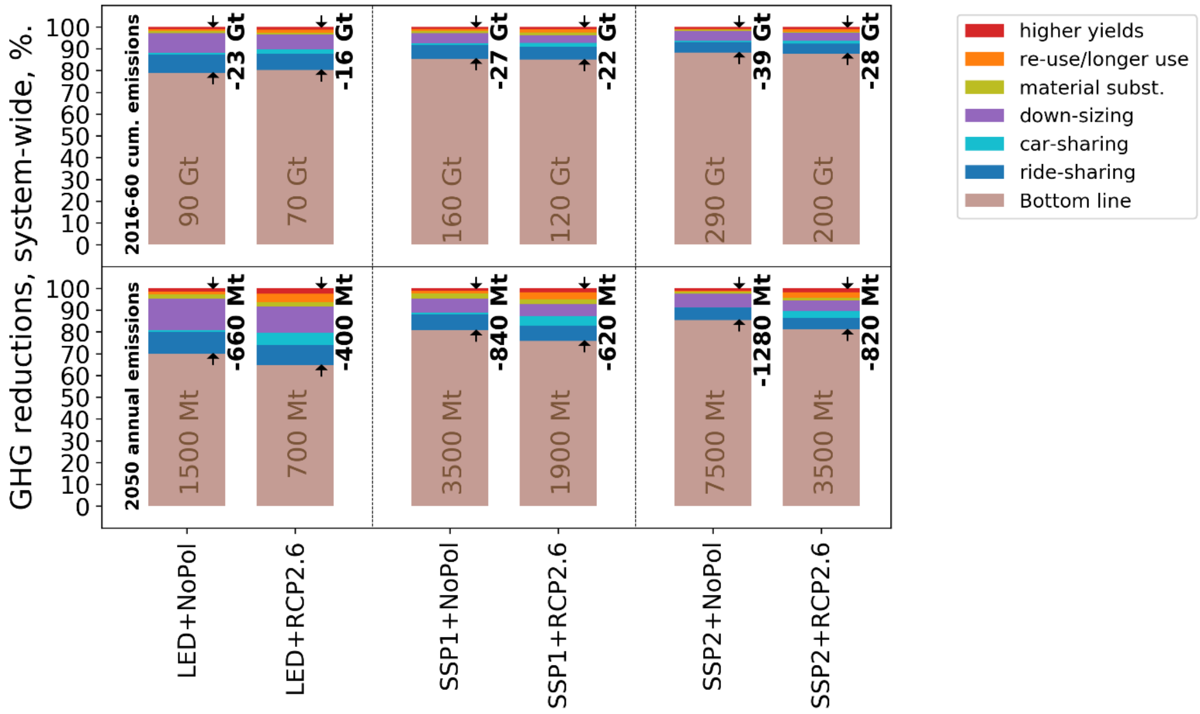

Residential buildings:

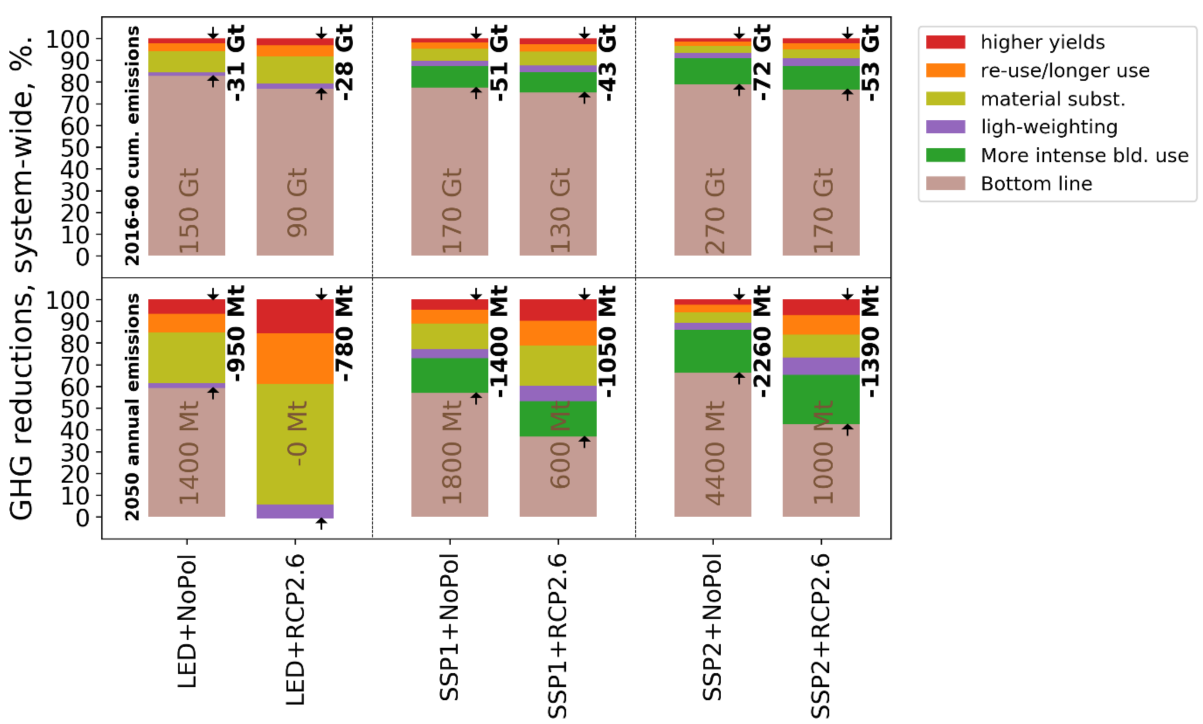

Passenger vehicles and residential buildings:

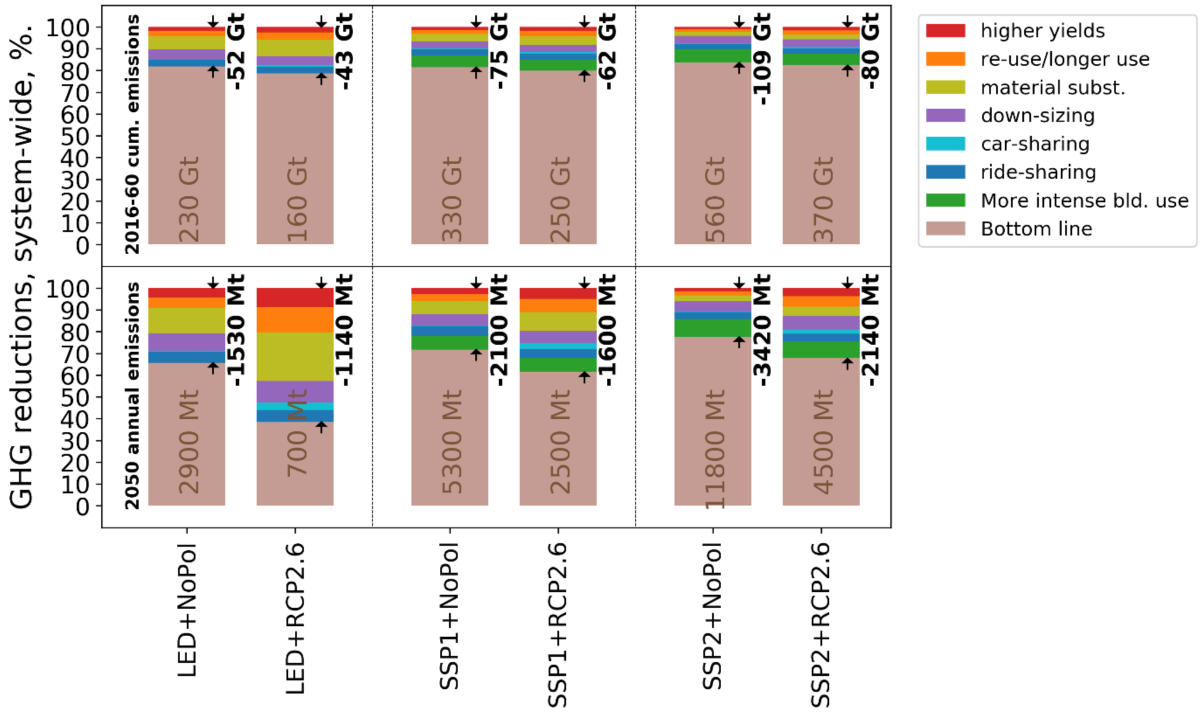

Supplementary Figure 26: Total global cumulative (top row) and annual *2060* (bottom row) emissions reductions of the technical potential of ten supply, demand- and sufficiency-based material efficiency strategies, by socioeconomic and climate policy scenario and ME strategy for the passenger vehicle (pav) and residential building (reb) sectors combined. Top: passenger vehicles, middle: residential buildings, bottom: pav and reb together.

Other supporting material

The second part of the supporting information below provides a detailed description of the ODYM-RECC v2.4 model as well as a summary of the input data. The data gathering is documented in the respective data templates of the 104 model parameters, which are available via https://doi.org/10.5281/zenodo.4671643.

The ODYM-RECC v2.4 model is available under a permissive license via https://github.com/YaleCIE/RECC-ODYM. The model results here were calculated in August and October 2020 by running the ODYM-RECC scripts of commit no. 7bd4e46 with the data in the archive linked above.

These results were evaluated in April 2021 with revised RECC v2.4 evaluation scripts, model commit 9c93d9b, April 2021, and are archived on https://zenodo.org/record/4698619.

Global Assessment of Resource Efficiency and Climate Change Mitigation (RECC)

Documentation of part IV of the RECC model framework: Open Dynamic Material Systems Model for the Resource Efficiency-Climate Change Nexus (ODYM-RECC), v2.4, GLOBAL version

Stefan Pauliuk*

with input from the entire RECC modelling team, in particular: Niko Heeren, Tomer Fishman, Qingshi Tu, Paul Wolfram, Peter Berrill, Andrea Nistad, and Edgar Hertwich

Freiburg (Breisgau), Germany, April 2021

This report contains the documentation of the ODYM-RECC model version 2.4, used to generate the RECC scenarios for the case study with global coverage

*) stefan.pauliuk@indecol.uni-freiburg.de
Faculty of Environment and Natural Resources, University of Freiburg
Tennenbacher Strasse 4
D-79106 Freiburg, Germany.

Link to related manuscript of journal article:
"Global Scenarios of Resource and Emissions Savings from Systemic Material Efficiency in Residential Buildings and Cars",
Preprint available via https://doi.org/10.21203/rs.3.rs-93217/v1

Table of Contents

Glossary 103
Summary 104
1. Background, introduction, and literature review 109
1.1. Literature review: Previous approaches to modelling material efficiency on the large scale 109
Economic modelling 109
Biophysical modelling of material efficiency 110
Combining economic and biophysical modelling 111
1.2. Research gap 111
1.3. General terms and definitions: 112
1.3.1. Our scoping of the term resource efficiency 112
1.3.2. Our scoping of the term material efficiency 113
1.4. Nomenclature and where to find what: 114
Project material 114
Model Framework 114
RECC database 115
2. Research questions, and project structure 116
ODYM_RECC research questions 116
Project structure 116
Model development prioritisation 118
3. System definition, model resolution, time frame 119
3.1. Project-wide system definition 119
3.2. Main project scoping: 120
3.3. Description of the aspects covered by ODYM-RECC 123
3.4. Resolution of model aspects. 124
4. Model calibration and scenario development 129
4.1. Scenario framing and model drivers 129
4.2. Scenario development mechanisms 131
4.3. Running and evaluating the scenarios, material efficiency cascade 133
4.4. Model calibration 136
5. Data needs and data gathering 138
5.1. Description of the data gathering process 139
5.2. ODYM-RECC parameters, complete list. 141
5.2.1. Socioeconomic parameters 141
5.2.2. Technology parameters 143
5.2.3. Resource efficiency parameters 147

5.3. Numerical data, units, and uncertainty in ODYM-RECC ... 150
5.4. ODYM-RECC parameter list, version numbers, and rationales ... 151
6. The ODYM-RECC model ... 166
6.1. Theoretical foundation of ODYM-RECC ... 166
6.2. Reference to methods and software used. ... 167
6.3. Basic ODYM-RECC modules and model equations ... 167
6.3.1. ODYM-RECC modules, overview ... 168
6.3.2. System variables ... 169
6.3.3. General description of resource efficiency strategies ... 173
6.3.4. The use phase module (UP) ... 174
6.3.5. The waste management and recycling module (WR) ... 182
6.3.6. Link to function provision, energy consumption, and environmental extensions/pressures (module EX) ... 183
6.3.7. Manufacturing (MF module) and the closure of the recycling loop ... 186
6.3.8. Link to material composition of products and materials (ME module) ... 186
6.3.9. The primary material production (PP module) ... 187
6.3.10. Mining industries (MR module) ... 188
6.3.11. Socioeconomic impacts ... 188
6.4. Sensitivity analysis and scenarios ... 189
7. Modelling environment, work flow, and interfaces ... 190
7.1. Modelling environment: Software, database, and sharing ... 190
7.2. Running the ODYM-RECC model ... 191
7.3. RECC project work flows and database status ... 193
RECC core rules: ... 193
RECC workflows, internally: ... 193
7.4. Interfaces from and to the ODYM-RECC model ... 195
From the scenario database (I) to ODYM-RECC (IV): ... 195
From the archetype model (II) to ODYM-RECC (IV): ... 196
From the LCIA (III) to ODYM-RECC (IV): ... 196
8. Outlook, future model expansion and development ... 197
8.1. Expanding the scope of the ODYM-RECC model ... 197
8.2. Expanding the capabilities of the ODYM-RECC model ... 197
8.3. Interface to other modelling frameworks ... 198
Integrated assessment models ... 198
8.4. ODYM-RECC FAQs ... 198
Supplementary References ... 199

Glossary

BAU Business-as-usual (scenario baseline)

EoL End-of-life, referring to a product after the end of its useful life: end-of-life product

LED Low energy demand, a scenario for low energy demand for decent living standards published by Grubler et al. [9].

ME Material efficiency, increasing the ratio of useful output/service by material input

ODYM Open Dynamic material systems model, a Python toolbox for dynamic material flow analysis [6]

ODYM-RECC Open Dynamic Material Systems Model for the Resource Efficiency-Climate Change Nexus

Primary material, primary production: Material produced from virgin (mineral) resources

RE Resource efficiency, increasing the ratio of useful output/service by resource input

RECC Resource efficiency and climate change mitigation

RES Resource efficiency strategy

Secondary material, secondary production: Material produced from scrap, both fabrication and postconsumer scrap

SSP Shared socioeconomic pathway, a comprehensive scenario storyline for future human and societal development, developed and used mostly by the climate change mitigation / integrated assessment modelling community.

Summary

ODYM-RECC model

The ODYM-RECC model (open dynamic material systems model for the resource efficiency and climate change mitigation project) is a modular depiction of major end-use sectors and the material cycles for the climate-relevant bulk materials [6] (https://github.com/YaleCIE/RECC-ODYM). Its system definition (Supplementary Figure 27) comprises the use phase of materials (in products) and the material cycle stages mining, primary production, manufacturing, waste management and scrap recovery, and remelting/recycling as well as an energy supply scenario.

ODYM-RECC generates a set of what-if scenarios [27] for the climate-relevant end-use sectors and bulk material cycles against different socioeconomic, technology deployment, and climate policy backgrounds. It does so by applying a mass-balanced framework for the material cycles [24]. It allows us to study the impacts of a broad spectrum of sustainable development strategies on the material cycles and identify trade-offs and constraints. It does not assess the likelihood of realisation of any of the scenarios studied but checks if mass balance constraints (e.g. by long product lifetimes or limited scrap supply) render some scenarios unfeasible from a material cycle point of view.

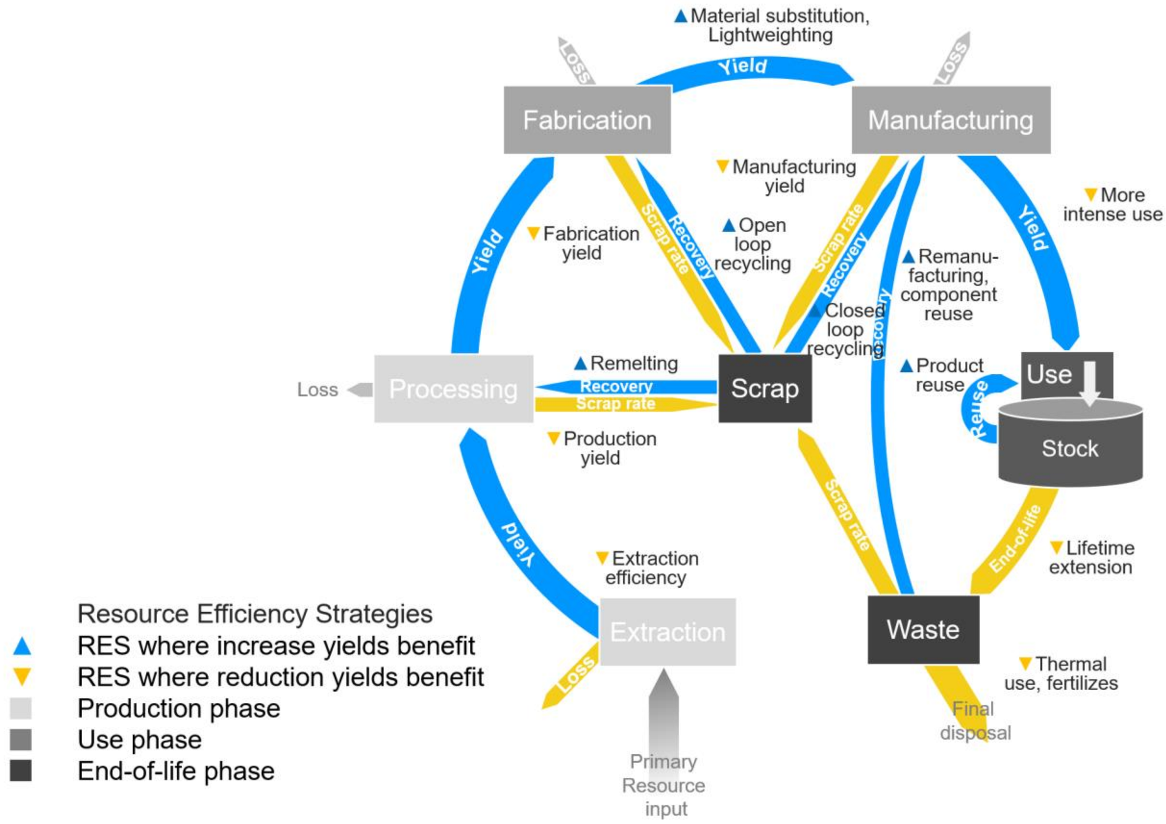

Supplementary Figure 27: System definition of ODYM-RECC assessment with processes and flows studied, resource efficiency strategies, and the modelling approaches taken for the computation of the material cycle response to resource efficiency. Inspired by Allwood et al. [40] and Reck and Graedel [41]. Figure drawn by Tomer Fishman for the RECC project.

ODYM-RECC is a multi-layer model depicting products, materials, chemical elements, energy flows, and emissions, with mass balance across all processes down to the individual chemical element. ODYM-RECC has six modules that quantify the system in Supplementary Figure 27 by translating a given service scenario into product stocks, inflows and outflows (module 'use phase UP', using stock-driven modelling [23], product outflows into scrap and recycled materials (module 'waste management and recycling WR', using parameter equations), product inflows into material demand and fabrication scrap (module 'manufacturing MF' using parameter equations), material demand into primary production and related impacts (module 'primary production PP', using environmental extension factors), and by determining the chemical element composition of al stocks and flows (module 'material-element composition ME', using mass balance). Finally, the energy consumption and environmental pressure and impact indicators are calculated (module 'energy and extensions EX').

For the RECC project, 35 data aspects (time, age-cohort, process, material, chemical element, waste/scrap, environmental extension, socioeconomic scenario…) were defined and each of the 90+ model parameters has a specific data model that links it to the data aspects. For example, the parameter for the product lifetime extension potential has the three aspects 'product', 'region', and 'scenario. The parameter for the future stock levels needed has the four aspects 'scenario', 'product', 'region', and 'time'. The resolution of each data aspect is defined in the model configuration file, a summary is given in Supplementary Table 7.

Supplementary Table 7: ODYM-RECC model and data resolution.

Model and data aspect	Resolution
Time	2016-2060 in steps of 1 year, results are reported for/by 2050.
Age-cohorts/Vintages	Vehicles: 1980-2060, residential and non-residential buildings: 1900-2060, appliances: 1971-2060, industry (electricity generation assets): 1986-2060.
Regions	For passenger vehicles and residential buildings: 20 countries and world regions, covering the entire world. For non-residential buildings and appliances: one aggregate global region. For industrial assets (electricity generation): 11 world regions.<br>Global North: OECD countries, countries of the former USSR, and China.<br>Global South: India, Africa, Latin America, Middle East, All other Asian countries
Products	6 passenger vehicle types and 24 archetypes, 13 residential building types and 52 archetypes, 24 detailed non-residential building types and 96 archetypes (Germany only), 4 aggregated non-residential building types, 18 electricity generation technologies, and 12 types of appliances.
Engineering materials	construction grade steel, automotive steel, stainless steel, cast iron, wrought Al, cast Al, copper electric grade, plastics, wood and wood products, zinc, concrete
Waste and scrap types	heavy melt, plate, and structural steel scrap; steel shred; Al extrusion scrap, auto rims, clean; Al old sheet and construction waste; Al old cast; copper wire scrap; construction waste, concrete, bricks, tiles, ceramics
Chemical elements	C, Al, Cr, Fe, Cu, Zn, 'other', traced through materials and waste
Energy carriers	Electricity, coal, hard coal, diesel, gasoline, natural gas, hydrogen, fuel wood

Service categories	Driving (vehicles), heating, cooling, domestic hot water (residential and non-residential buildings)
Scenarios	Socioeconomic: Low energy demand (LED), SSP1, SSP2<br>Climate policy: No policy after 2020 (reference scenario), 2 degrees Celsius (66%), corresponding to RCP2.6 forcing pathway.

The model parameters are linked to the system variables (stocks and flows shown in Supplementary Figure 27) via the model equations, which are grouped into the five ODYM-RECC modules. The parameters are divided into three groups: socioeconomic parameters such as future population, service demand, or intensity of operation of stocks (e.g. vehicle-km per year), technology parameters like energy efficiency of stock operation of the future emissions intensity of energy supply, and resource efficiency parameters describing both the potential for resource efficiency at the different stages of the system, and the speed of implementation of these potentials under different socioeconomic and climate policy scenarios.

Each RE strategy can be implemented separately or as part of a cascade of strategies. The model allows for calculating the impact of one strategy at a time (sensitivity analysis) or a bundle of strategies in different orders of implementation, each for different socioeconomic and climate policy scenarios.

The model and the corresponding input data and results database were released under a permissive license on https://github.com/YaleCIE/RECC-ODYM and on Zenodo:

RECC Global input database: https://doi.org/10.5281/zenodo.4671643

RECC Global results: https://zenodo.org/record/4698619

The ODYM-RECC Database

The ODYM-RECC v2.4 database contains 104 model parameters of two to six dimensions each. Parameters range from static values (direct emissions of combustion by MJ of energy carrier) to highly detailed highly uncertain datasets (e.g., the future energy carrier split of buildings by region, time, and operation mode (heating/cooling/hot water).

The ODYM-RECC database was compiled as a community effort involving a large number of experts. Data templates and project wide classifications were used to facilitate the compilation of the various types of information.

Depending on data availability, we applied several pathways of data compilation, which are listed and described in detail below.

- Extract mostly socioeconomic parameters from existing scenario models (scenario reference)
- Compile own plausible scenario estimates for socioeconomic parameters in line with the different scenario narratives where established model framework results are not available (group consensus scenarios)
- Extract process-, product, and material-specific data from the engineering and industrial ecology literature (bottom-up data)
- Extract quantitative estimates of resource efficiency strategy potentials, mostly related to prototypes and case studies, from the literature (strategy potentials)

- Simulate energy consumption and material composition of a number of building and vehicle archetypes with specialised software, which are then used as bottom-up product descriptions with and without implementation of RE strategies (archetype descriptions)

Scenario reference: For the socioeconomic parameters the Shared Socioeconomic Pathways (SSP) database and model results as well as available data from the World Energy Outlook and Energy Technology Perspectives models were used wherever possible, e.g., for future population, future GHG intensity of energy supply, or the drive technology mix for vehicles [28–32]. The data were extracted from available databases (like the SSP scenario database hosted at IIASA: https://www.iiasa.ac.at/web/home/research/researchPrograms/Energy/SSP_Scenario_Database.html) or shared by colleagues, then parsed and reviewed by the RECC team, then aggregated, disaggregated, and interpolated to fit the ODYM-RECC project-wide classification. For each parameter file the data gathering process is documented both in the respective template files in the RECC database (if only Excel was used), in custom scripts (for more comprehensive datasets) and in the data log files archived under https://github.com/YaleCIE/RECC-data.
Group consensus scenarios: For some parameters like the future stock levels or the split of residential buildings into different types no detailed SSP-consistent scenario calculation was available that we could refer to. Hence we assumed a set of plausible target values for a number of socioeconomic parameters in line with the storylines of the individual socioeconomic scenarios. This process is commonly used when translating broad storylines into high product and regional resolution and sector-specific parameters, cf. Riahi et al. [28] and Grübler et al. [9]. The target values for 2020, 2030, 2040, 2050, and 2060 chosen and the rationale for their choice are documented in scenario target tables, one for each parameter. From there, the target values are read, interpolated, smoothed with a moving average, and exported in ODYM format to be directly used in the ODYM-RECC model. The documentation of this process is available in Fishman et al. [4] and the documentation for the individual parameters is archived in https://github.com/YaleCIE/RECC-data.

The open model and data framework allow for third parties to modify the scenario assumptions and to run calculations with custom parameters and storylines.

Bottom-up data: For the energy intensity, emissions intensity, and material composition of products and processes detailed but representative product or process descriptions were compiled from the literature and available databases. These data include the material composition and specific energy consumption of vehicles and buildings, e.g., [33–35], the loss and recovery rates for the manufacturing and waste management industries e.g., [36,37], and the specific energy consumption and process emissions for the manufacturing, waste management, and primary material production industries [31,32,38,39]. While the data can be regarded as representative of current average global technology, their main limitation is that they are static and no information on their change under different socioeconomic and climate policy scenarios, in particular, is given. To become more realistic a scenario reference was made wherever possible (cf. above), e.g., for the changing GHG intensity of the supply of different energy carriers, for which a combination of MESSAGE IAM results and IEA Energy Technology Perspective results was used. Also, for the average GHG intensity of primary metal production a scenario analysis based on ecoinvent was calculated to take into account scenario-dependent changes of the GHG intensity of electricity generation.

Resource efficiency **Strategy potentials:** For some parameters, including the improvement potentials for fabrication scrap, end-of-life recovery efficiency of scrap, re-use of steel components in buildings, or product lifetime extension, previous estimates can be used [25]. For the more intense use of products,

the in-use stock curves for the scenario with the next smallest mitigation challenge were used, i.e., SSP1 values in the SSP2 scenario and LED values in the SSP1 scenario.

Archetype descriptions: For the final product categories residential buildings and vehicles, the product-specific simulation tools BuildME, GREET (https://greet.es.anl.gov/) and FASTSim (https://www.nrel.gov/transportation/fastsim.html) were used to derive model estimates for both the material composition and energy intensity of operation for different building and vehicle archetypes. For each of the nine building and six vehicle types four archetypes, representing maximal potential for change, were simulated: a standard product without special consideration of material efficiency, downsizing, or material substitution, a downsized product, a product with ambitious material substitution, and a downsized material-substituted product.

For a detailed description and definition of all model aspects, the classifications used for them, the system variables and parameters, the model equation and their division into modules and the data compilation, (dis)aggregation and formatting process, we refer to the ODYM-RECC model documentation.

The ODYM-RECC database is formatted in standardised spreadsheets and archived on Zenodo (dataset DOI https://doi.org/10.5281/zenodo.4671643), barring the confidential and licensed input data, which are available on request.

1. Background, introduction, and literature review

Global human extraction of biomass, minerals, and fossil fuels has risen to more than 90 Gt/yr and is directly associated with 50% of all human impact on the climate and 90% of all biodiversity loss and water stress [16].

Due to the sheer magnitude and ubiquity of human resource extraction and processing [15,16], their future environmental impacts and mitigation options need to be studied from a systems perspective [42]. Ambitious global dematerialization scenarios need to be formulated, their system-wide consequences explored, their political feasibility studied, and their impact mitigation opportunities lined out in detail.

Such assessments are already underway. For example, the 2015 World Energy Outlook by the IEA contains a material efficiency scenario [10], with the following central findings. First, on P26: "Changing product design, re-use and recycling (“material efficiency”) also offers huge potential for energy saving; for energy-intensive products such as steel, cement, plastics or aluminium, efficient use and re-use of materials can save more than twice as much energy as can be saved by efficiency measures in the production process to 2040." And on P387: "Achieving greater efficiency in the use of materials through light-weighting, longer life products, re-use and recycling, is an important complementary strategy to energy efficiency in energy-intensive industries, as the potential for energy savings is about twice as large."

To be able to study the system-wide impacts of material use and material efficiency and to robustly quantify the potential of the different mitigation options, the scientific community needs a push in dynamic material cycle modelling. The different resource efficiency, sufficiency, and circular economy strategies and their impact on material cycles, energy use, and environmental damage needs to be understood better, and prospective scenario modelling can make a central contribution to generate such knowledge [43]. Current established socioeconomic scenario models, in particular integrated assessment models, do not capture material cycles at the level of detail necessary to answer research question related to the linkage of resource efficiency and climate change [44]. The concurrent IRP assessment team states that there is “no known global forward-looking [built asset] model available” [18]. A recent OECD report comes to a similar conclusion: More detail and better connection between technology-detail (‘bottom-up’) and aggregated macroeconomic (‘top-down’) representations is needed [11].

1.1. Literature review: Previous approaches to modelling material efficiency on the large scale

Economic modelling

Global system wide repercussions of resource efficiency have been captured by a number of general equilibrium approaches. A list and review of recent approaches is given in Wining et al. [12]. It includes the work with computable general equilibrium models (CGE) of Böhringer and Rutherford [14], the EllenMcArthur Foundation and McKinsey, EXIOMOD, and GINFORS as approaches to assess resource efficiency in a GCE framework, as well as the econometric model E3ME and the mixed model framework GIAM/GTEM-C. Recent major additions to the literature are the Global Resource Outlooks

by the OEDC and the UN IRP, both published in 2019 and built upon a CGE framework [15,16]. For such works, CGE-based macro-economic models, such as GTEM-C are combined with physical accounts or physical sectoral models [17,45,46], including MEFISTO stock and flow framework [19].

A recent model review by the OECD [11] found that the material cycle processes relevant for quantifying the economy-wide impacts of material efficiency in a detailed manner are not described by these models, hence, such assessments can only give a rough estimate of future material use. They can neither be checked for physical correctness (do the service-providing products actually need that many materials for their production?), nor can the savings potential of the many different material efficiency strategies and policy options be assessed. Clearly, improvements are needed.

Physical detail needs to be added to macro-economic models. Wining et al. [12] and Schuhmacher and Sands [20] amend CGE models by adding detail about steelmaking, e.g., by disaggregating the steel sector into the primary and secondary production route. [21] use an MRIO approach to study the linkage between circular economy strategies, energy use, and emissions. They do not capture material flows and cycles themselves, as these are not covered by monetary IO models.

Biophysical modelling of material efficiency

The biophysical modelling approach uses engineering models as common in industrial ecology, such as prospective life cycle assessments or dynamic material flow analysis, to create a physical linkage between service provision and material flows (the so-called material stock-flow-service nexus) [1]. These approaches include much technological detail and estimations of the impact of a number of material efficiency strategies (‘bottom-up’). While the number of product-level life cycle assessments that include some kind of material efficiency or other circular economy strategies abounds (e.g., for material substitution in vehicles as reviewed by Kim and Wallington [47]), there are only some examples of detailed technology-based assessments of material efficiency at the large scale. These include a detailed assessment of material efficiency in the global steel cycle [25], a case study for reducing cement demand in the UK [48], and a study on the material efficiency-climate change mitigation link for the climate-relevant bulk materials in the EU [49]. Hertwich et al. [50] provide a comprehensive review of these studies and their findings. The high level of detail of such work allows for a robust estimation of the *technical potential* of the different strategies in the different sectors, taking into account system effects such as a changing quality of postconsumer scrap or export of excess secondary material to other sectors. Still, it is not clear what the economy-wide potential of such strategies would be, as costs are often not considered. More importantly, the economy-wide consequences of ambitious material efficiency, such as material-related rebound effects [51,52], are ignored, even by the studies that include costs, leading to potentially flawed (over-optimistic) policy recommendations.

Material efficiency was pushed (again) on the policy agenda [53] and later [40] defined six core material efficiency strategies: more intense use, light-weighting, lifetime extension, re-use, fabrication scrap reduction, and fabrication scrap diversion.

A first global assessment of these six material efficiency strategies was undertaken for the steel cycle [25]. Material efficiency in the steel cycle could reduce emissions from the steel sector by 50% in the future compared to present levels, and thus complements the spectrum of emissions mitigation potential with gigaton potential.

Future metal and material demand has been projected and studied from the perspective of different macro-level scenarios [54–62], but those assessments are not linked to resource efficiency, but represent a very important starting point for our work, as we can link our scenarios and data to these studies.

Combining economic and biophysical modelling

The MATTER project, which ran in the Netherlands between 1995 and 1999, aimed at establishing a link between material cycles and energy use and GHG scenarios produced by the MARKAL energy system model [63–66]. The main finding is that a material-related GHG emissions savings potential of up to 1 Gt exists for Western Europe, including strategies in waste management, material efficiency, and material substitution.

The scope of MATTER was limited to Western Europe, but for an assessment of global climate targets a global scope is needed. MATTER also ignored the coupling between material cycles. Back in the 90ies, the recent development in China, which produces now about half of many bulk metals globally, could not have been anticipated. Technology (dismantling and sorting) and policy (Paris Agreement, circular economy) have advanced significantly since then, and a refined modelling approach is needed now to incorporate the recent progress in resource policy and material flow analysis.

A GDP-driven steel cycle model is now part of the IMAGE integrated assessment framework [67,68], which contains all major steel-related technologies but which is not connected to the rest of the IMAGE scenarios, where buildings, vehicles, and consumer appliances are depicted in detail, thus lacking internal consistency.

Finally, there are some recent attempts to link material consumption to economy-wide models more directly. First, by converting sectoral output of CGE models into material flows by applying product material composition and prices [12,69], and second, by converting end-user demand for new products provided by energy system models into material flows by applying product material composition data [60,62]. These attempts are a step in the right direction, but the CGE approaches focus on single economic sectors only and do not consider material cycles and the mitigation potentials therein, and the energy system-based approaches only estimate final demand and currently do not consider the material cycle response.

1.2. Research gap

The research described above has led to ad-hoc models describing material systems in a partial and incomplete manner only. A holistic prospective assessment framework for metal cycles is currently lacking. The importance of metals for sustainable development was studied from different angles (cf. above), but these studies were all done in isolation, using only parts of the available data, too narrow system boundaries, and simplified scenario drivers.

Due to the scattered modelling approaches, the field of prospective metal cycle studies is severely underdeveloped (Pauliuk and Hertwich, 2016). **The link between prospective material cycle studies and climate policy assessments, especially by integrated assessment models, is almost completely lacking** (Pauliuk et al., 2017). The impact of the circular economy on the different material cycles, the very subject of the circular economy, cannot be quantified with the available tools. That lack of modelling capability is problematic as the system-wide benefits of material-specific strategies such as recycling and material efficiency cannot be correctly assessed. **The spectrum of GHG mitigation options is artificially (for practical and not for scientific reasons) narrowed down to technologies described by the incumbent integrated assessment models.**

It is the job of the modelling team of ODYM-RECC to contribute to filling that gap and to provide to the industrial ecology community a powerful scenario tool for the assessment of resource efficiency and other industrial ecology strategies from a systems perspective.

1.3. General terms and definitions:

1.3.1. Our scoping of the term resource efficiency

Resource efficiency is a very broad concept, roughly defined as “using the Earth's limited resources in a sustainable manner while minimising impacts on the environment. It allows us to create more with less and to deliver greater value with less input.”
(http://ec.europa.eu/environment/resource_efficiency/, accessed 2018-01-16)

In a wider meaning, resources include materials, biomass, and energy across the entire economy, including technical raw materials and primary energy, but also refined materials, products, and refined (secondary) energy. Sometimes, water is also included. That definition includes food and food products. It is understood as material and energy efficiency applied across all economic processes and consumption stages. Exergy is sometimes proposed as a common measure for this type of resource efficiency [70–72], but especially for materials quality in terms of physical properties is the desired outcome of the industry, and this objective cannot be captured by exergy efficiency.

In a more narrow sense, resources include material resources across all economic processes and consumption stages, but not water, food, and energy carriers. That means *engineering materials, including metals, construction materials and minerals, wood/timber, and man-made materials such as plastics*. In this context, resource efficiency is then understood as economy-wide material efficiency [53,73] (Supplementary Box 1).

Supplementary Box 1: RE strategy scope.

For the ODYM-RECC assessment resource efficiency is understood as economy-wide (engineering) material efficiency (ME), that means material efficiency across all industries and consumption stages. We also include energy efficiency and the impacts of material efficiency on energy use, as the link between materials and energy is particularly important when assessing the system-wide impacts of ME strategies from a life cycle perspective.

The system boundary of ODYM-RECC spans the entire industrial system from the environment-technosphere boundary to the services provided to final consumers, which is identical to the system scope of the life cycle inventory of products and services (service level described as functional unit to elementary flows). This overlap of system boundaries is crucial to the combination of the material cycle and product life cycle perspectives.

The resource efficiency scope ME includes all the 3R (reduce, reuse, recycle), 6R, 9R (rethink, reduce, reuse, repair, refurbish, remanufacture, repurpose, recycle, and recover) and other circular economy strategies.

1.3.2. Our scoping of the term material efficiency

The RECC project will investigate core material efficiency strategies in the use phase of products and the material cycles of bulk materials (cement, steel, plastics, …) [40]. The list of strategies considered and their definitions and implementations for residential buildings and vehicles is listed below in Supplementary Table 8.

Supplementary Table 8: The ten material efficiency strategies considered in ODYM-RECC V2.4.

Strategy	Buildings (residential and non-residential)	Vehicles
Using less material by light-weighting through improved design and/or downsizing, **ULD**	Optimized Design: Using less material by better design and engineering without loss in functionality	Segment shift from large vehicles (light trucks, sports utility vehicles) to smaller ones (passenger cars).
Less material through light-weighting by material substitution, **MSu**	Implementation differs for the two models. For buildings, materials with lower life-cycle emissions are being used. For vehicles, material is substituted to achieve less operational energy demand.	
	Wooden buildings have less life-cycle emissions than concrete or brick buildings. While other material options exist, wood is particularly effective because of the carbon sequestration. Regional limits to wood supply are taken into account.	Replacing steel with aluminum or high-strength steel (not considered here) reduces life-cycle emissions due to weight reduction and subsequent fuel savings in the use phase
Fabrication yield improvement, **FYI**	Fabrication yield improvements (FYI) reduces the amount of material scrap in the fabrication and manufacturing process, thereby lessening the demand for material input to the manufacturing sector.	
End-of-life recovery rate improvement, **EoL**	End-of-life recovery rate improvement (EoL) increases the share of materials salvaged as scrap from end-of-life products	
Fabrication scrap diversion, **FSD**	Large pieces of manufacturing scrap, like trimmings or cuttings, can be diverted into other manufacturing units for manufacturing smaller components from them. This avoids the remelting step and potentially reduces costs.	
Car-sharing, **CaS**	Does not apply.	Shift away from the personal car to the use of cars from a shared fleet
Ride-sharing, **RiS**	Does not apply.	Driving patterns where people with same or similar driving destinations share a ride. Different from ride-hailing, which is a modified taxi service.
More intensive use, **MIU**	MIU implies that fewer products are required to provide the same basic service. For buildings, peer-to-peer lodging is a potential strategy, in addition to steps such as increased household size/cohabitation, and a reduction of second homes.	Does not apply.

Product lifetime extension, **LTE**	Better design, increased repair, enhanced secondary markets.
Recovery, remanufacturing, and reuse of components, **ReU**	Replacing the production of spare parts or even primary products.

A detailed description of the implementation of the different strategies can be found in the model and data description chapters below.

1.4. Nomenclature and where to find what:

Project material

A general publicly available project description can be found here:
https://cie.research.yale.edu/project_main/resource-efficiency-climate-change
There are three public repositories for the project material:

1. A GitHub public page (https://github.com/YaleCIE/RECC-public) to share publicly available material like publications, documentation, posters, talks, and other.
2. The GitHub repository with the ODYM-RECC model code:
https://github.com/YaleCIE/RECC-ODYM
3. The database of the project, archived on Zenodo (dataset DOI https://doi.org/10.5281/zenodo.4671643).

Internally (for project team members), there is another repo mainly used for data documenation: https://github.com/YaleCIE/RECC-data as well as an internal shared folder \Dropbox\G7 RECC\, where the data, all project documents, and the main results are stored.

Model Framework

We distinguish between the model framework, which is general for dynamic MFA, and its application for this project.

The name of the general model framework is

ODYM - *Open Dynamic Material Systems Model*

The public repository on GitHub (https://github.com/indecol/odym/) hosts the software framework ODYM. A publication on ODYM is also available [6].

For our common project we use the acronym

RECC - *Resource efficiency and Climate Change Mitigation*

The service-material cycle-climate model used for this project is then

ODYM-RECC

The ODYM-RECC model is hosted on GitHub in an open repository: https://github.com/YaleCIE/RECC-ODYM

RECC database

All 104 ODYM-RECC v2.4 parameters are formatted into the same general data model [74] and are available as Excel templates, through which they are parsed by the model. The current ODYM-RECC database is stored on the common Dropbox folder \Dropbox\G7 RECC\Data. The database of the model version 2.4 with global coverage is archived on Zenodo (https://doi.org/10.5281/zenodo.4671643).

2. Research questions, and project structure

The ODYM-RECC research questions are listed below in Supplementary Box 2.

Supplementary Box 2: Research questions for ODYM-RECC, part IV of the model framework.

ODYM_RECC research questions

The following research questions are guiding model development, data gathering, and scenario analysis for ODYM-RECC:

RQ1) What is the impact of the different material efficiency strategies on material cycles, energy use, and GHG emissions for different socioeconomic scenarios until 2060?

RQ2) How large are the trade-offs and co-benefits of the different material efficiency strategies when implemented together?

RQ3) What socioeconomic or lifestyle changes translate directly into lower material use and what are the possible GHG savings until 2060?

RQ4) How big is the impact of material efficiency strategies on burden shifting across economic sectors and on the life cycle performance indicators of products and services?

Project structure

The RECC model framework and database consist of four modules, whose interaction is depicted in Supplementary Figure 28:

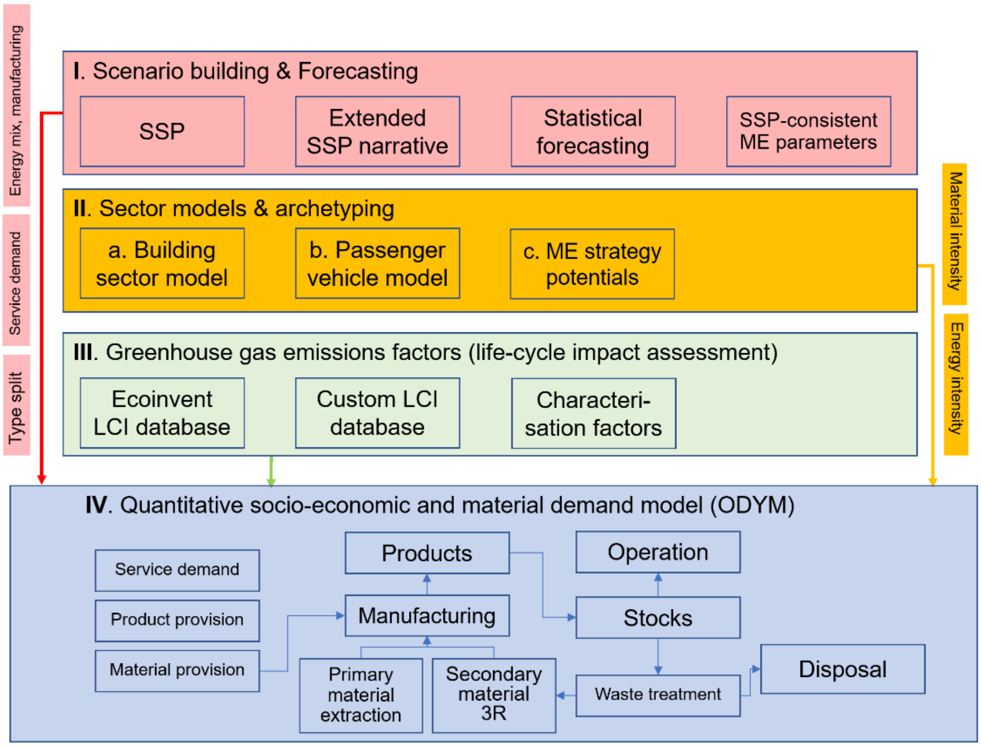

Supplementary Figure 28: RECC model framework and database, overall structure. Figure drawn by Niko Heeren, more detailed version available in Fishman et al [4]. The overall model consists of four key elements: I. Scenario formulation, II. Sector models and archetype description, III. Environmental impact assessment factors, and IV. Quantitative socio-economic material demand model (ODYM-RECC). Module I contains the data from the Shared-Socioeconomic Pathways (SSP) [28,75]. The interfaces between the ODYM-RECC model and the other parts are described in section 7.3.

The overall RECC project workflow follows the structure outlined in Supplementary Figure 29:

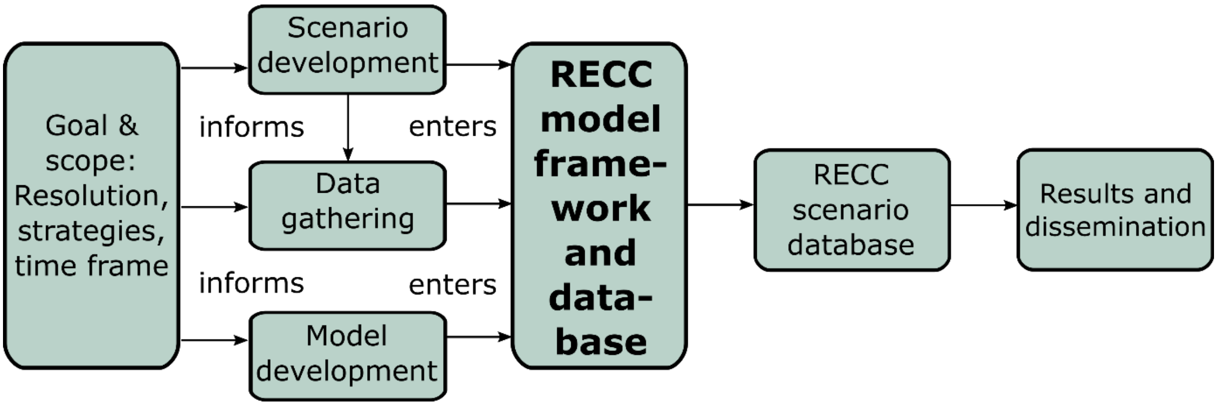

Supplementary Figure 29: ODYM-RECC workflow, aggregated. The ODYM-RECC model (part IV) of the project's model framework is where all raw, refined, and product-based data enter the large-scale scenario model and where the main results are calculated and exported to the different formats.

The main body of the ODYM-RECC documentation is structured as listed in Supplementary Box 3 below:

Supplementary Box 3: Chapter structure of this model documentation.

**In the subsequent chapters, the model framework is explained in detail.**
**Section 3:** System definition, assessment resolution, and time frame
**Section 4:** Model calibration, scenario description and development
**Section 5:** Data description and data gathering
**Section 6:** ODYM-RECC model description and development
**Section 7:** Working environment and work flow, interfaces between modules
**Section 8:** Outlook
**Appendix**

Model development prioritisation

With the given time frame and available resources, the following priorities/steps were chosen:

P1) Implement a generic description of future passenger vehicles and residential buildings for the major world regions until 2060 and calculate material cycle response to material efficiency strategies across system, cover climate-relevant bulk materials.

P2) Represent all G7 countries, China, and India separately.

P3) Provide a detailed and consistent scenario description for the multiple model parameters.

P4) Developed a detailed inventory of archetypes for vehicles and buildings to be scaled up.

P5) Refine model and database, consider carbon cycle and timber supply constraints, recycling limits, etc.

For the current model version 2.4, which was used for the case study with global scope, all steps above could be implemented.

3. System definition, model resolution, time frame

3.1. Project-wide system definition

The system definition of a material cycle is shown in Supplementary Figure 30. The more detailed ODYM-RECC system definition with process group numbers is shown in Supplementary Figure 31.

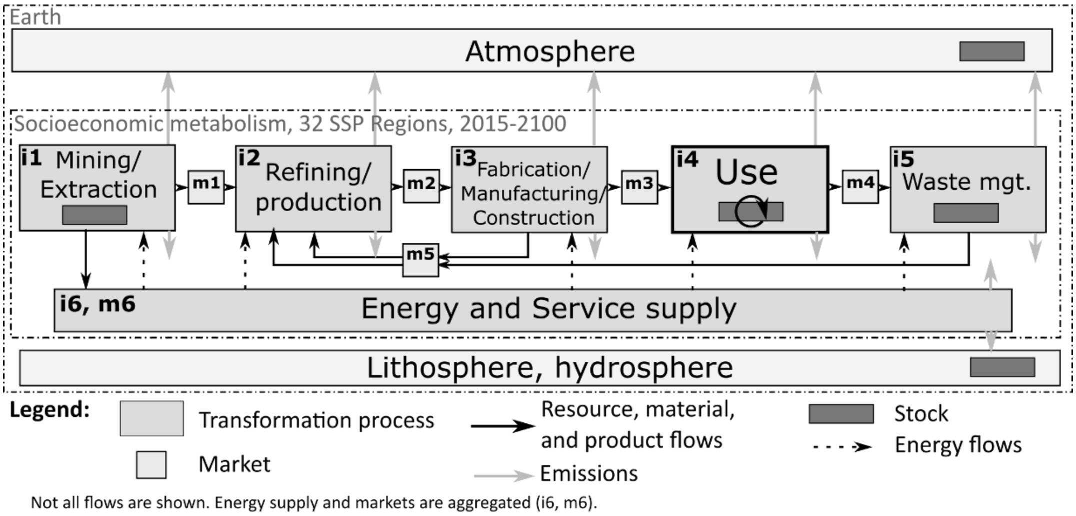

Supplementary Figure 30: Generic system definition of a material cycle with process group numbers. The process groups can have sub-indices, allowing us to distinguish between different product groups (m3), waste types (m4, m5), etc.

Based on the generic system definition, Supplementary Figure 31 provides an overview of the resource efficiency strategies covered for ODYM-RECC.

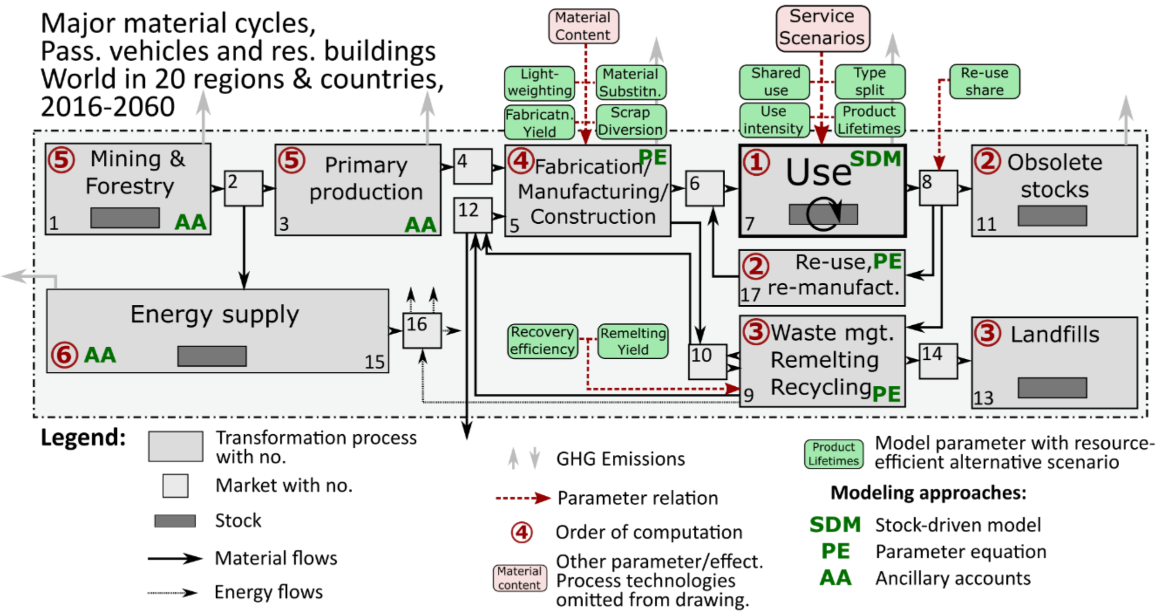

Supplementary Figure 31: System definition of ODYM-RECC assessment with model parameters, resource efficiency strategies, and the modelling approaches taken for the computation of the material cycle response to resource efficiency.

The mathematical representation of the different RE strategies is introduced in the model description chapter.

3.2. Main project scoping:

Starting point of the RECC assessment is the physical service level: m^2 of dwelling space and non-residential floor space and mobility in terms of passenger-km/yr. The GHG emissions associated with these two services are major contributors to the GHG balance in high income countries, next to industry (e.g. for Germany: 190 Mt for residential buildings, ca. 100 Mt for non-residential buildings (inkl. electricity), and 150 Mt for passenger vehicles out of the country's total emissions of ca. 900 Mt/yr, https://www.umweltbundesamt.de/en/indicator-greenhouse-gas-emissions) (Supplementary Figure 32).

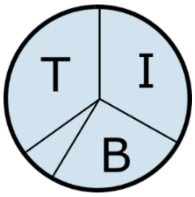

Supplementary Figure 32: The three major energy and material using sectors Transport (T), Buildings (B), and Industry (I). Infrastructure is part of the transport sector, together with vehicles. Industrial buildings are part of the industrial sector, but are currently not captured by the RECC assessment.

For the IRP RECC assessment the following services and service-providing stocks were considered:

RECC project, services and their links to stocks:

i) Shelter and thermal comfort, provided by **residential and non-residential buildings (m^2).**

ii) Mobility, provided by vehicles and traffic infrastructure, measured in vehicles, passenger-km and infrastructure-km. **The focus here lies on passenger vehicle transportation, (passenger-km / year).**

iii) Appliances, service **measured in pieces (per category).**

iv) Electricity generation capacity, **measured in GW of production capacity**.

Not included are:

v) Infrastructure and vi) Material production, manufacturing, and waste handling capacity, provided by industrial assets, measured in GWh/yr, Mt/yr, etc. and vii) Vehicles and transport modes other than passenger cars.

Services are linked to material cycles via the stock-flow-service nexus [1] (Supplementary Figure 33). The scheme starts with the energy service cascade to relate values to services to functions to products (and their operation) [2], stock-driven modelling to translate product in-use stock demand into production of new and recycling of old products [23], new and old products to material flows via dynamic material flow analysis (MFA) [24], and the material flows to the energy demand and related GHG emissions via environmental extensions as done in previous work [25,26].

Supplementary Fig. 33 (next page): Calculation scheme for the use phase (here shown as 'product stocks'). Stock levels are determined from historic stocks and scenarios following different storylines. The stock-driven model then determines the age-cohort decomposition of the in-use stock as well as product inflows and outflows and the associated material content. With the total stock broken down into different age-cohorts by the stock-driven model, the function and energy flows of the use phase can then be determined (cf. below) by applying the following parameters in turn: intensity of operation and intensity of use (for service flows) and energy intensity and energy carrier split (for energy use of the use phase). The indices are as follows (cf. RECC config table and RECC index table 3.1): t: time, c: age-cohort, r: region, g: good/commodity/product, S: scenario (SSP, RCP, and/or RE), V: service category, n: energy carrier, t_0: starting time of prospective assessment (2015). See also Tables 5.2-5.4 for an explanation of the different parameters. The red section of this figure is our interpretation and implementation of the energy service cascade [2].

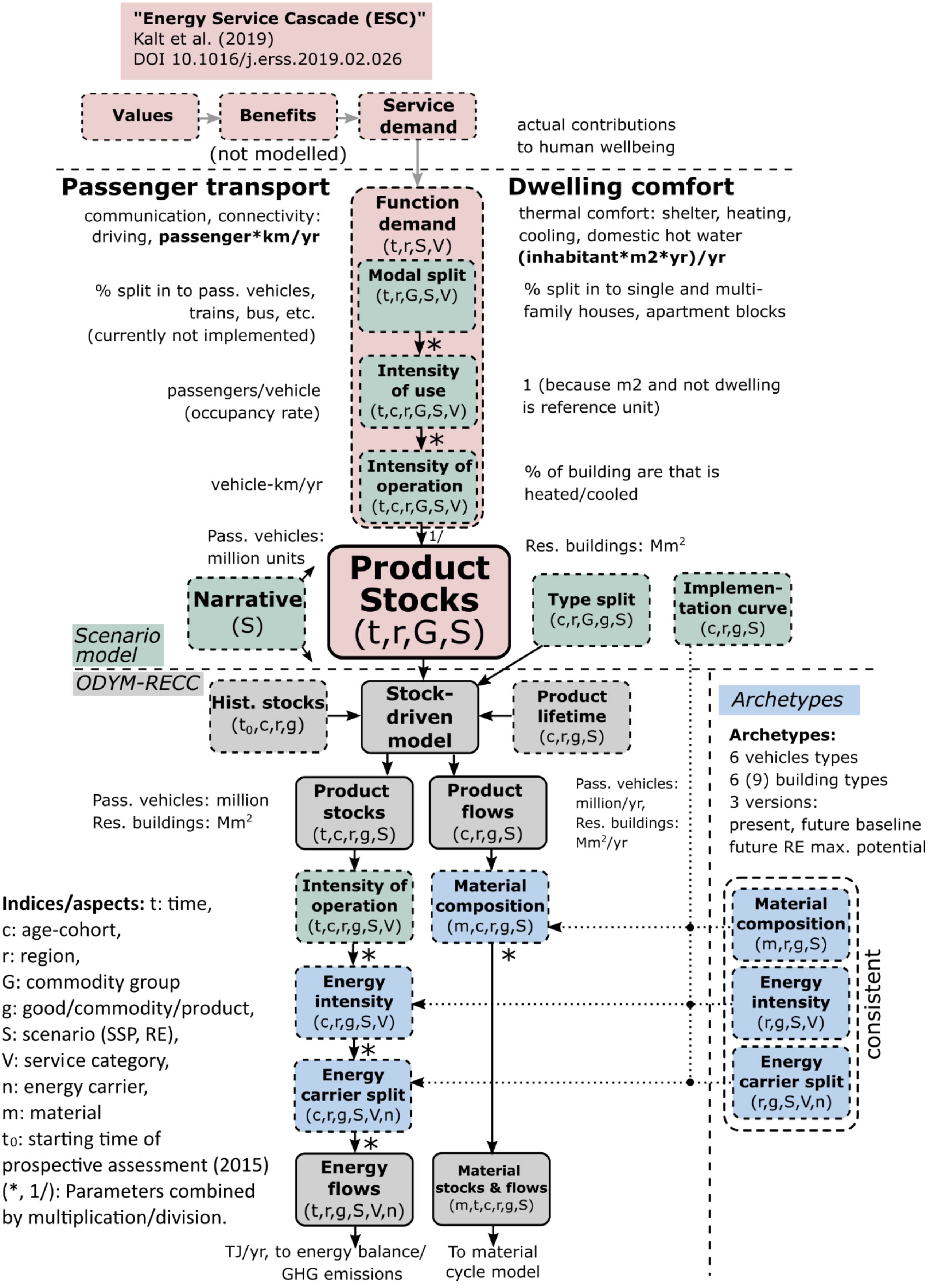
"Energy Service Cascade (ESC)"
Kalt et al. (2019)
DOI 10.1016/j.erss.2019.02.026
Values
Benefits
Service demand
(not modelled)
actual contributions to human wellbeing
Passenger transport
communication, connectivity: driving, passenger*km/yr
Function demand (t,r,S,V)
Dwelling comfort
thermal comfort: shelter, heating, cooling, domestic hot water (inhabitant*m2*yr)/yr
% split in to pass. vehicles, trains, bus, etc. (currently not implemented)
Modal split (t,r,G,S,V)
% split in to single and multi-family houses, apartment blocks
passengers/vehicle (occupancy rate)
Intensity of use (t,c,r,G,S,V)
1 (because m2 and not dwelling is reference unit)
vehicle-km/yr
Intensity of operation (t,c,r,G,S,V)
% of building are that is heated/cooled
1/
Pass. vehicles: million units
Res. buildings: Mm2
Product Stocks (t,r,G,S)
Narrative (S)
Type split (c,r,G,g,S)
Implemen-tation curve (c,r,g,S)
Scenario model
ODYM-RECC
Hist. stocks (t0,c,r,g)
Stock-driven model
Product lifetime (c,r,g,S)
Archetypes
Archetypes:
6 vehicles types
6 (9) building types
3 versions:
present, future baseline
future RE max. potential
Pass. vehicles: million
Res. buildings: Mm2
Product stocks (t,c,r,g,S)
Product flows (c,r,g,S)
Pass. vehicles: million/yr,
Res. buildings: Mm2/yr
Intensity of operation (t,c,r,g,S,V)
Material composition (m,c,r,g,S)
Material composition (m,r,g,S)
Energy intensity (c,r,g,S,V)
Energy intensity (r,g,S,V)
Energy carrier split (c,r,g,S,V,n)
Energy carrier split (r,g,S,V,n)
consistent
Energy flows (t,r,g,S,V,n)
Material stocks & flows (m,t,c,r,g,S)
TJ/yr, to energy balance/ GHG emissions
To material cycle model
Indices/aspects: t: time,
c: age-cohort,
r: region,
G: commodity group
g: good/commodity/product,
S: scenario (SSP, RE),
V: service category,
n: energy carrier,
m: material
t0: starting time of prospective assessment (2015)
(*, 1/): Parameters combined by multiplication/division.

3.3. Description of the aspects covered by ODYM-RECC

The elements of the system (processes with stocks, and flows) can be described along different aspects, such as time, age-cohort, regions, processes, materials, etc. [74]. First, all relevant aspects need to be introduced and then their resolution for the first assessment round is stated.

The ODYM-RECC *index table* [6] is part of the ODYM-RECC configuration excel file RECC_Config_V2_4.xlsx. The ODYM-RECC model framework covers the following aspects as specified in its index table (Supplementary Table 9).

Supplementary Table 9: Index table of ODYM-RECC, model version 2.4.

Aspect	Description	Dimension	Index letter
**Time**	Model time	Time	**t**
**Cohort**	age-cohorts	Time	**c**
**Element**	chemical elements	Element	**e**
**Unity**	trivial classification, 1 entry only	Unity	**u**
**Region32**	region of process or stock, region of origin (flow)	Region	**r**
**Region11**	region of process or stock, region of origin (flow)	Region	**l**
**Region5**	region of process or stock, region of origin (flow)	Region	**f**
**Region1**	region of process or stock, region of origin (flow)	Region	**o**
**MaterialProductionProcess**	Engineering material production processes	Process	**P**
**Engineering materials**	Engineering materials considered	Material	**m**
**ManufacturingProcess**	Manufacturing processes	Process	**F**
**Sectors**	Aggregated product groups: buildings, vehicles, …	Good_Product	**G**
**Good**	List of ALL goods and products considered	Good_Product	**g**
**Cars**	List of car types considered	Good_Product	**p**
**OtherVehicles**	List of other vehicles considered	Good_Product	**v**
**ResidentialBuildings**	List of residential building types considered	Good_Product	**B**
**NonresidentialBuildings**	List of nonresidential building types considered	Good_Product	**N**
**Infrastructure**	List of infrastructure considered	Good_Product	**I**
**Industry**	List of industry considered	Good_Product	**I**
**Appliances**	List of appliances considered	Good_Product	**A**
**WasteManagementIndustries**	Waste management industries	Process	**W**
**Waste_Scrap**	waste and scrap types considered	Material	**W**
**Energy**	Energy consumed	Energy carriers	**N**
**Scenario**	Scenerios considered (e.g., SSP)	Scenario	**S**
**Extensions**	Costs, emissions factors, social impacts	Extensions	**X**
**Scenario_RCP**	RCP scenarios	Scenario	**R**
**SSP_Population_model**	Population model used for SSP scenarios	Scenario	**M**
**ServiceType**	Different uses of building energy: heating, cooling, …	Extensions	**V**
**Archetype**	Product archetypes	Good_Product	**A**
**Custom**	Custom aspect, for calibration parameter	Unity	**C**
**Car_segments**	Segments of passenger vehicles	Good_Product	**S**
**Regions32goods**	List of goods with regional aggregation level 32	Good_Product	**T**
**Regions11goods**	List of goods with regional aggregation level 11	Good_Product	**L**
**Regions1goods**	List of goods with regional aggregation level 1	Good_Product	**O**
**Env. impact/pressure category**	Pressure indicator dimensions such as GWP etc.	Extensions	**x**

3.4. Resolution of model aspects.

For each of the model aspects defined in the aspect table a common classification that defines a certain resolution is used. The resolution of the most central aspects are listed here.

The information presented here is a summary only. The full info about the resolution of the RECC project is documented in the Master classification file, which is part of the project's database: **RECC_Classifications_Master_V2.0.xlsx**

Time and age-cohort, dimension: Time:

- The time frame is 1900-2060, as some historic data reach back to 1900 and before. The Actual modelling period is 2016 to 2060, where usually, results until 2050 are extracted for reporting and publication.

Regions, dimension: Region:

- The two end-use sectors passenger vehicles and residential buildings are implemented for 20 countries and world regions: (cf. RECC_Classifications_Master_V2.0.xlsx for details), Supplementary Table 10:

Supplementary Table 10: RECC Global regions and their aggregation

ODYM-RECC region (20)	Description	Global North	Global South	EU28
R32CAN	Canada	X		
R32CHN	China	X		
R32EU12-M	"New" EU countries, medium income	X		X
R32IND	India		X	
R32JPN	Japan	X		
R32USA	USA	X		
France	France	X		X
Germany	Germany	X		X
Italy	Italy	X		X
Poland	Poland	X		X
Spain	Spain	X		X
UK	UK	X		X (not in EU27)
Oth_R32EU15	Other "old" EU countries,	X		X
Oth_R32EU12-H	Other "new EU countries, high income	X		X
R5.2OECD_Other	Other OECD countries	X		
R5.2REF_Other	Countries of the former USSR	X		
R5.2ASIA_Other	Other Asian countries		X	
R5.2MNF_Other	Middle East and Northern African Countries		X	
R5.2SSA_Other	Sub-Saharan Africa Country		X	
R5.2LAM_Other	Latin-American Countries		X	

- The two end-use sectors appliances and non-residential buildings are implemented in a single, aggregate global region.

- The intermediate industrial sector 'electricity generation' is implemented for 11 world regions: (cf. RECC_Classifications_Master_V2.0.xlsx for details):
    - AFR
    - CPA
    - EEU
    - FSU
    - LAC
    - MEA
    - NAM
    - PAO
    - PAS
    - SAS
    - WEU

Eningering_Materials, dimension: Material:

- Construction grade steel
- Automotive steel
- stainless steel
- Cast iron
- Wrought Al
- Cast Al
- Copper electric grade
- Plastics
- Cement
- Wood and wood products
- Zinc
- Concrete
- Concrete aggregates

UsePhase, dimension: Process:

- Cf. Products resolution

Products, dimension: Good_Product:

- **Passenger vehicles:**
  Internal Combustion Engine, gasoline (ICEG)
  Internal Combustion Engine, diesel (ICED)
  Hybrid Electric Vehicles (HEV)
  Plugin Hybrid Electric Vehicles (PHEV)
  Battery Electric Vehicles (BEV)
  Fuel Cell Vehicles (FCV)

- **Residential buildings (SFH = single family house, MFH = multi-family house, RT = residential tower):**
  SFH_non-standard
  SFH_standard
  SFH_efficient

SFH_ZEB (zero energy building)
MFH_non-standard
MFH_standard
MFH_efficient
MFH_ZEB
RT_non-standard
RT_standard
RT_efficient
RT_ZEB
informal_non-standard

- **Nonresidential buildings (global):**
  nonres_agg_hotels
  nonres_agg_governmental
  nonres_agg_office
  nonres_agg_retail

- **Nonresidential buildings (Germany only):**
  nonres_offices_non_standard
  nonres_offices_standard
  nonres_offices_efficient
  nonres_offices_ZEB
  nonres_commercial_non_standard
  nonres_commercial_standard
  nonres_commercial_efficient
  nonres_commercial_ZEB
  nonres_education_non_standard
  nonres_education_standard
  nonres_education_efficient
  nonres_education_ZEB
  nonres_health_non_standard
  nonres_health_standard
  nonres_health_efficient
  nonres_health_ZEB
  nonres_hotels_restaurants_non_standard
  nonres_hotels_restaurants_standard
  nonres_hotels_restaurants_efficient
  nonres_hotels_restaurants_ZEB
  nonres_other_non_standard
  nonres_other_standard
  nonres_other_efficient
  nonres_other_ZEB

- **Appliances**
  Fan
  Air-cooler
  Air-conditioning
  Refridgerator
  Microwave
  Washing Machine
  Tumble dryer
  Dish washer
  Television
  VCR/DVD player

PC & Laptop computers
Other small appliances

- **Electricity generation**
  solar photovoltaic power plant
  concentrating solar power plant (CSP)
  wind power plant onshore
  wind power plant offshore
  hydro power plant
  nuclear power plant
  coal power plant
  coal power plant without abatement measures
  bio powerplant
  oil power plant
  geothermal power plant
  IGCC power plant
  light oil combined cycle
  gas combined cycle power plant
  advanced coal power plant with CCS
  coal power plant with CCS
  biomass power plant with CCS
  gas combined cycle power plant with CCS

EoL goods, dimension: Good_Product:

- Cf. Products resolution

Energy, dimension: Energy carriers:

- Electricity
- Coal, hard coal
- Diesel
- Gasoline
- Natural gas
- Hydrogen
- Fuel wood

SSP_Scenarios, dimension: Scenario:

- LED (low energy demand)
- SSP1 (Shared Socioeconomic Pathway 1)
- SSP2 (Shared Socioeconomic Pathway 2)

RCP_Scenarios, dimension: Scenario:

- RCP2.6
- Baseline (no new climate policy after 2020)

Env. extensions, dimension: Extensions:

- CO2 emisisons per main output
- CH4 emissions per main output
- N2O emissions per main output
- SF6 emisisons per main output

- GHG emissions
- GHG emissions, supply chain

Env. midpoints, dimension: Extensions:

- GWP 20/100/500
- GTP 20/100/500

Chemical Elements, dimension: Element:

- C
- Al
- Cr
- Fe
- Cu
- Zn
- 'Other' (sum of all elements not explicitly listed, for mass balance)

MaterialProductionProcess, dimension: Process:

- One (average) primary production process for each material.

ManufacturingProcess, dimension: Process:

- One average manufacturing process for each product/good

Waste management process, dimension: Process:

- One waste mgt. (dismantling, shredding, sorting) process to convert each of the 15 products into waste/scrap at the end of life, one re-melting process for each scrap category

Waste/scrap, dimension: Material:

- Heavy melt, plate, and structural steel scrap
- Steel shred
- Al extrusion scrap, auto rims, clean
- Al old sheet and construction waste
- Al old cast
- Copper wire scrap
- Construction waste, concrete, bricks, tiles, ceramics
- Thermoplastic waste
- Used wood

Car segments, Good_Product:

- microcar
- passenger car
- minivan_SUV
- light truck

4. Model calibration and scenario development

About half of the ODYM-RECC parameters (54 out of 104) is scenario-dependent, meaning, that their values need to be linked to an exogenous socioeconomic or climate policy storyline.

4.1. Scenario framing and model drivers

During the time frame of the RECC project we will not be able to establish a close connection to technology-rich IAMs or other comprehensive bottom-up models of the passenger vehicle and residential building sector to obtain detailed and authoritative drivers for material cycles. Moreover, the parameters needed here are often not considered by such models. We will hence need to implement a standalone but SSP-compatible assessment, and it was the task of the scenario team to add the relevant detail to the SSP and LED storyline. The scenario relevant aspects/parameter groups and the individual parameters are listed in Supplementary Table 11 below.

Supplementary Table 11: Broad scenario model parameter categories in the ODYM-RECC framework.

Group	Parameter
**D0) Present stock levels [no scenario]**	Current levels of in-use stocks and breakdown into age-cohorts EXOGENOUS
**D1) Future service level**	Total service need by category: m^2 of buildings, passenger-km delivered, EXOGENOUS
**D2) Future technology level**	Type of products that supply the services: vehicle types, building types, … EXOGENOUS
**D3) Future material stock level**	In-use stock of material by application, region, and scenario, ENDOGENOUS
**D4) Future material demand and EoL material supply**	Final material demand (in products) by region and scenario, supply of materials in EoL products by region and scenario., ENDOGENOUS
**D5) Resource efficiency strategies**	Cf. Figure 3.2, EXOGENOUS
**D6) Material industry response**	Technology setup and decision making in the mining, refining, manufacturing, and waste management sectors, ENDOGENOUS
**D7) Industry background response**	Electricity mix, carbon intensity of service, EXOGENOUS
**D8) Mining industry scenarios**	Bulk and companion metal production scenarios, extraction capacity development, brownfield exploration, greenfield exploration, and social and environmental impacts of future mining operations. NOT PART OF ODYM-RECC

D0) Present stock levels, EXOGENOUS

All model parameters with time series start at their present levels for the reference year 2015, the latest year where complete historic data were available. The 2015 in-use stocks represent a lock-in that partly determines future outflows and thus the potential for recycling and for the introduction of new technologies, especially for the vehicle fleet until 2030 and the building stock all throughout 2060. One therefore needs to know the current levels of in-use stocks, their lifetimes, and breakdown into age-cohorts.

D1) Future service levels and use phase parameters, EXOGENOUS

Starting point of the scenario modelling is the use phase, where services and the related *in-use stocks are described as a function of time*. Future per capita stock levels can be derived from

- The literature, both from the IE and the IAM communities
- a regression model using population, GDP, and urbanisation (only suitable for interpolation due to non-stationarity of time series)
- a detailed descriptive scenario for future service levels *(approach taken here)*

Irrespective of their origin, the future stock curves enter the stock-driven model as simple functions of the independent variable time and the aspects product group, region, and scenario. That approach increases the transparency of the approach, as we display those functions, explain how they were derived, and invite others to create their own functions.

In addition, the use phase parameters product lifetime and obsolete stock formation need to be quantified.

D2) Future technology levels, EXOGENOUS

Future technologies, e.g., the share of electric vehicle in transportation, can be derived as above from

- The literature, both from the Energy system modelling and the IAM communities *[approach taken for passenger vehicles]*
- a regression model using population, GDP, and urbanisation
- a detailed descriptive scenario for future service levels *[approach taken here for residential buildings]*

D3) Future material stock levels, ENDOGENOUS

Future material stock levels describe the material content of the products required to deliver services to end users, such as residential buildings and passenger vehicles.

Stock levels for individual buildings are determined by multiplying the product stock size with the respective material content.

The material composition is determined from available data for historic age-cohorts up to 2015 and from an archetype representation and mixing of different archetypes into average products for a given future years, region, and scenario.

D4) Future material demand and EoL material supply, ENDOGENOUS

The future final consumption of materials in products and the supply of materials for recovery in EoL products is the link between the use phase and the rest of the material cycle. It is determined endogenously in ODYM-RECC by solving a stock-driven model [23] (future stock curve and lifetime distribution determine product inflows and outflows to/from use phase), and multiplying those flows with the age-cohort and region-specific material composition of products yields the material demand (final consumption) and the available material in EoL products.

Since the sectors captured in ODYM-RECC do not comprise the entire economy but just a part of it (currently, only residential buildings and passenger vehicles are covered), this approach does not give the total future material demand, which is needed to determine global mining and production levels and the total extent of recycling.

D5) Resource efficiency strategies: Potentials and implementation patterns, EXOGENOUS

Ten resource efficiency strategies are within the scope of the rapid assessment for the G7, India, and China. They are defined in terms of model equations (section 6), then implemented in the software, and then quantified by scaling up reference/prototype implementation cases using implementation curves that indicate how quickly and to which extent the different prototypes will be used in the future.

D6) Material and waste mgt. industry response, ENDOGENOUS

Using process parameters, the ODYM-RECC model calculates the levels of re-use/remanufacturing and recycling/remelting. The potentials for reuse and recycling improvement enter this calculation, they are scenario-dependent.

D7) Industry background response (Energy mix etc.), EXOGENOUS

Changes in the future energy mix and carbon intensity of services determines the future life cycle impacts of products consumed. To estimate the climate impact of the different RE strategies we need to account for changes in the future energy mix, and we were able to obtain scenario results for the GHG intensity of energy supply for the different SSP scenarios from the MESSAGE team.

D8) Mining industry scenarios NOT PART OF ODYM-RECC

Based on future lifestyles, consumption patterns, and technology choices a certain amount of primary materials will be needed. Given estimates of the future extent of recycling one can then infer the amount of primary production needed, and develop mining exploration, extraction capacity development, and production scenarios for both bulk and companion/minor metals to advice mining developers and resource policy makers on which types of deposits are likely to be needed most in the future. This extension is outside the system boundary of the RECC project and not part of the assessment. Instead, the GHG emissions of primary production is modelled with a static mining process description.

4.2. Scenario development mechanisms

Starting point of RE implementation is the socioeconomic background provided by the shared socioeconomic pathways (SSP) scenario family, Supplementary Figure 34 [29,75].

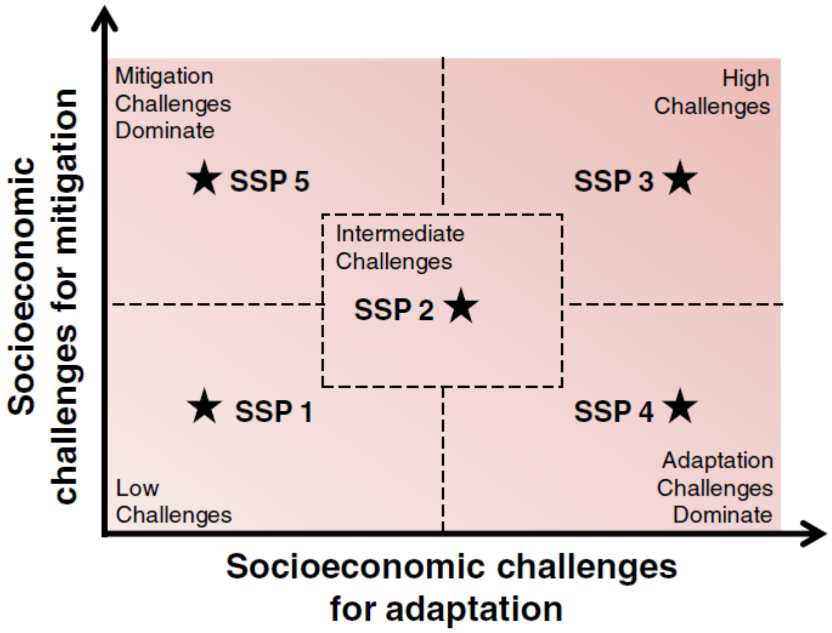

Supplementary Figure 34.: Space of challenges for mitigation of and adaptation to climate change. The challenge space is divided into five "domains" with one SSP located within each domain, represented by a star. **Image source:** O'Neill et al. [29].

The core SSP scenario drivers include: Population, urbanisation, and GDP (Supplementary Figure 35), and these are available from the IIASA scenario database for the 32 SSP regions. https://tntcat.iiasa.ac.at/SspDb/dsd?Action=htmlpage&page=about

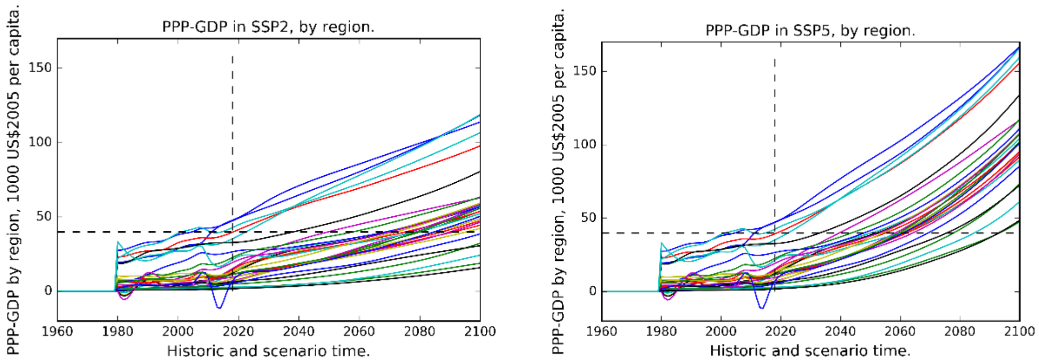

Supplementary Figure 35: PPP GDP per capita in SSP2 (left) and SSP5 (right), by region. Vertical dashed line: 2018, horizontal dashed line: 40000 US$2005 per capita. Negative values are contained in SSP database. Own plot. Note that in the current model setup, only the SSP1 and SSP2 scenarios/storylines are included. The ODYM-RECC model does NOT use GDP as a model driver/parameter.

Within the RECC team, scenario development focusses on three parameter domains with the relevant parameters, which are defined and described in the data section below, and the different ways to obtain the relevant parameters and to add detail to the scenario storylines.

1. **Socioeconomic parameters** (population, future service levels, building types split, intensity of operation of vehicles)
    a. From existing databases, such as the SSP scenario database or scenario work of the International Energy Agency IEA
    b. Expert consensus in line with the SSP storylines within the project team to identify plausible target values for the individual parameters for 2040, 2050, and 2060. These

values are then inserted into a *scenario target table* and interpolated to produce time series from 2015 to 2060.

2. **Technology parameters** (Energy carrier split of buildings, GHG intensity of energy supply)
   a. From existing model rungs, e.g., results from the MESSAGE model and work by the International Energy Agency IEA
   b. Scenario target table approach (cf. above, internal expert consensus)
   c. Own modelling effort (for the future GHG intensity of primary material production)
   d. Archetype descriptions for products and processes (detailed description of individual products to be scaled up) from own modelling efforts to describe vehicle and building prototypes with established engineering planning tools
3. **Resource and material efficiency parameters** (Reuse share, recovery rate improvements)
   a. From case studies and prototypes described in the literature

4.3. Running and evaluating the scenarios, material efficiency cascade

A large number of different scenario settings is possible, which allow the model user to answer a wide array of different research questions. The different sectors can be run together or separate, the same holds for different countries. The larger the scope of a model run, the higher the change that the secondary material available from the scrap supply will find a market inside the system boundary and doesn't have to be exported.

For each model run, the following information needs to be supplied in the ODYM-RECC config file or in the scenario list table RECC_ModelConfig_List_V2_4.xlsx, from which the config file is populated via the script ODYM_RECC_ScenarioControl_V2_4.py

- Which region(s) are included
- Which sector(s) are included
- Which products are included
- Which material efficiency strategies are included (any combination is possible).
- Whether building renovation, scrap export, scrap recycling credits, and energy efficiency improvements are included

For each model configuration, the model script then computes six socioeconomic-climate policy scenarios:

Socioeconomic:

- LED / SSP1 / SSP2

Climate policy:

- No new climate policy after 2020 (NoNewClimPol) / RCP2.6, leads to 400 ppm of atmospheric CO_2 by 2100, likely to reach 2°C target.

For each material efficiency strategy we define two implementation cases: One where the strategy is absent and one where it is implemented to an extent specified by the scenario target table or by the so-called implementation curve (parameter 3_SHA_RECC_REStrategyScaleUp_V3.3), that describes the ramp-up over time. To facilitate the interpretation of the results, the different material efficiency strategies are either considered one by one in a single-strategy sensitivity analysis or in a cascade. Supplementary Table 12 defines the sequence of ME strategies for which the scenarios are run in ODYM-RECC (ME strategies implementation cascade).

Supplementary Table 12. Material efficiency cascades and their breakdown into individual strategies. *) vehicles only. #) residential buildings only. The cascade is a sequence of model runs where additional strategies are added in each step as indicated below. For passenger vehicles, there are six, for residential buildings five steps in the material efficiency cascade

	**ME strategies implementation cascade**						
	**0 (Current ME levels)**	**1**	**2**	**3**	**4**	**5**	**6** (cars only)
End-of-life recovery rate improvement **(EoL)**		x	x	x	x	x	x
Fabrication yield improvement **(FYI)**		x	x	x	x	x	x
Fabrication scrap diversion **(FSD)**		x	x	x	x	x	x
Reuse **(ReU)**			x	x	x	x	x
Lifetime extension **(LTE)**			x	x	x	x	x
Material substitution **(MSU)**				x	x	x	x
Using less material by design / down-sizing **(ULD)**					x	x	x
Car-sharing * **(CaS)**						x	x
Ride-sharing for cars * **(RiS)**							x
More intensive use of floor space # **(MIU)**						x	n/a

The following list describes the different ME strategies in more detail. See also section 6 for the corresponding modelling equations.

(EoL): The 2015 values for the end-of-life recovery rate (fraction of material in end-of-life products that is recovered in form of scrap) increase gradually (by 2040, as specified in the ME strategy implementation curve) to new values specified by the EoL-recovery rate improvement parameter.

(FYI): The 2015 values for the fabrication yield factor (fraction of material entering into the manufacturing process that actually physically ends up in the product) increase gradually (by 2040, as

specified in the ME strategy implementation curve) to new values specified by the fabrication yield improvement parameter.

(FSD): A gradually (by 2040, as specified in the ME strategy implementation curve) increasing fraction of automotive steel fabrication scrap is not sent to remelting but used directly without remelting, as secondary material, e.g., to produce small steel parts from stampings, trimmings etc., as specified by the fabrication scrap diversion parameter.

(ReU): A gradually (by 2040, as specified in the ME strategy implementation curve) increasing fraction of material in EoL products (postconsumer products) is not sent to recycling/remelting but dismantled and reused directly, without remelting. This affects spare parts for cars, concrete slabs, and wooden beams, and the reuse potential is specified by the reuse potential parameter.

(LTE): The lifetime of new efficient buildings and electric vehicles is gradually extended, with a change over time as specified in the ME strategy implementation curve, and a maximum lifetime extension potential as specific by the product-specific lifetime extension parameter.

(MSU): The replacement of conventional materials by materials that lead to lower product life cycle emissions is modelled by mixing different product archetype descriptions. Here, engineering tools for vehicles and buildings were used to model the driving cycle and energy balance for different drive technologies and building types for different material use scenarios. These different high resolution product archetypes are then mixed together to form the average new product in a given future year. Archetype mixing is controlled by a number of scenario-consistent parameters for vehicle segment split, vehicle light-weighting share, and building light-weighting and downsizing shares.

(ULD): The implementation of ULD works in the same manner as the MSU strategy. For ULD, a vehicle and building 'downsizing parameter' controls the share of smaller car segments and light-weighted building types in the total use phase inflow.

(CaS): The car-sharing parameter denotes how many vehicle-km are delivered by shared use of cars, with the consequence that the annual kilometrage of those cars is twice as high as the default, leading to a smaller car fleet and higher turnover than a state with no car-sharing. The effect that car sharing use also reduces per capita passenger-km is not considered, as the exogenously specified passenger-km scenario driver is assumed fix.

(RiS): The ride-sharing parameter denotes how many passenger-km are delivered by shared rides, with the consequence that the occupancy rate of ride-sharing vehicles is 1.4 times the default, leading to a smaller car fleet than a state with no ride-sharing.

(MIU): The stock curve for future residential and nonresidential floor area gradually declines to 80% of the default value for the given scenario, modelled with spline interpolation until 2055, but does not fall below the values specified by the LED scenario.

4.4. Model calibration

The different parameters based on partly inconsistent historical data need to lead to correct results. They also need to fit the future scenario times series. To achieve both goals, the database needs to be calibrated, which is done by changing the most uncertain parameters from their literature values to values that will lead to correct model results for selected reference values. Results from non-calibrated model runs show a typical peak in the model year 2016 (Supplementary Figure 36), because the gap in stock resulting from the difference between actual data and scenarios is filled in that year.

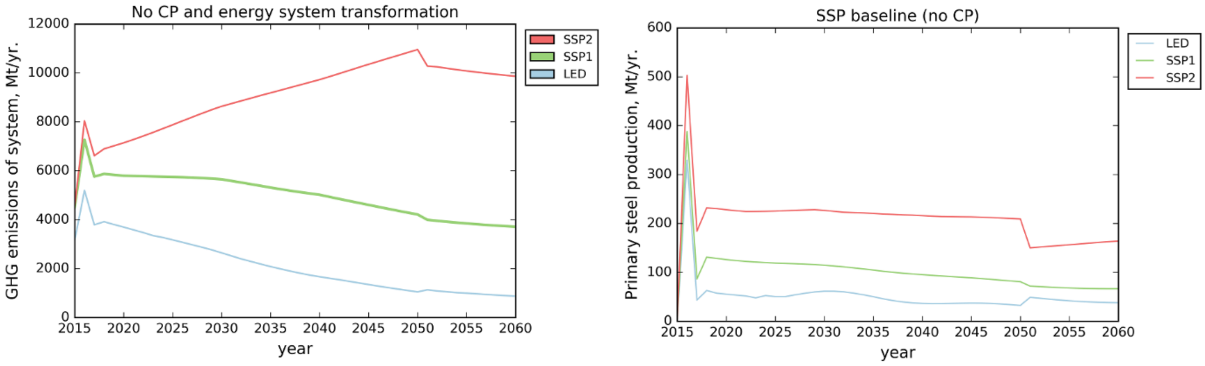

Supplementary Figure 36: Model result for non-calibrated historical data, with the characteristic 2016 peak in total emissions and material production.

The model calibration has two steps:

1) Stock calibration:

The 2015 stock per capita stock levels, which can be calculated from the stock parameter 2_S_RECC_FinalProducts_2015 and the population 2_P_RECC_Population_SSP_32R, need to be the starting points of the scenario curves. They need to be entered as 2015 values in the scenario target tables to make sure that the future stock curves have the right starting point.

2) Energy consumption calibration

Some of the 2015 statistical data in the RECC database need to be recalibrated to fit the reported energy demand. This procedure is documented in the parameter file 6_PR_Calibration. The calibration affects the use phase parameter kilometrage 3_IO_Vehicles_UsePhase and the use phase energy intensity of operation 3_EI_Products_UsePhase_passvehicles. For some regions, for example, the reported kilometrage and energy consumption (MJ/km) are too low to match the reported energy consumption. As a consequence, the vehicle and building energy intensity of operation are changed for most countries to reproduce the reported values for sectoral energy consumption, which is closely related to the GHG emissions statistics and therefore serves as reference for the calibration.

For details cf. the documentation in the ODYM-RECC parameter file 6_PR_Calibration_V2.4.xlsx.

3) Product lifetime calibration

The dynamic stock model computes the 2016 outflow from the historic stock. This outflow (EoL vehicles in million or demolished floor area in million m²) can be compared with statistical data, and, if the outflows are in strong disagreement with the literature values, the product lifetime of the historic age-cohorts can be adjusted to better translate into actual stock turnover. This led to an

adjustment of the average vehicle lifetime for Germany from 15 years to 14.5 years (see parameter file 3_LT_RECC_ProductLifetime_passvehicles) and of the residential building lifetime in some regions to values to 100 years and beyond (see parameter file 3_LT_RECC_ProductLifetime_resbuildings_V4.2).

4) Single product LCA

Not directly used for calibration, the computation of ODYM-RECC results for single product inflows can be used to check the model. Supplementary Figure 37 shows the 'dynamic LCA' of a single car, calculated with the standard parameter setting but overwriting the inflows, stock, and outflows of vehicles to simulate the life cycle of a single product. The simple dynamic LCA in ODYM-RECC was calculated by forcing the car inflow to 1 in 2020 and 0 else, and by changing stock and outflow accordingly to simulate a fixed lifetime of 15 years with standard km/yr.

The results (for Germany) are:

Share of production and EoL stages in life cycle GHG for gasoline vehicle produced in 2020: ca. 13%.

Share of production and EoL stages in life cycle GHG for BEV produced in 2020: ca. 39%.

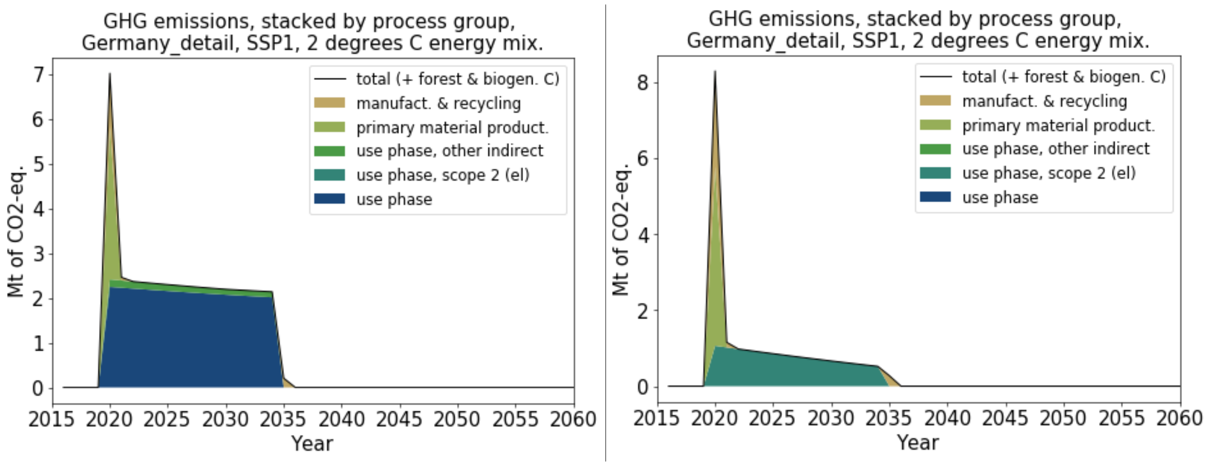

Supplementary Figure 37: Single vehicle dynamic LCI results for gasoline (left) and battery electric (right) vehicle. Note also the different emissions scopes: direct emissions (deep blue, left) vs. indirect emissions (right).

5. Data needs and data gathering

In this section, the 104 ODYM-RECC model parameters are defined and the main data sources and assumptions are listed. **In section 6, all parameters are connected to the system variables (stocks and flows in the model's system definition), and formally defined via the ODYM-RECC model equations.**

All parameters are defined in tuple format [74]:

parameter_value = function(aspect1, aspect2, aspect3, …)

The different aspects and their symbols are defined in the aspect table 3.1.

The parameter symbols are listed in the parameter definition tables below.

Each ODYM-RECC model parameter has a dataset ID, which consists of the following parts:

ParameterGroup_ParameterType_DescriptiveName_DataSetVersion

The general data model based on tuples or multi-dimensional arrays (data cubes), the six general parameter groups (Supplementary Table 13), and the assigned data types are adapted from the general data model for industrial ecology [74], which is documented as part of the industrial ecology data inventory under https://github.com/IndEcol/IE_data_commons

Supplementary Table 13: The ODYM-RECC parameter groups 1-6 and assigned parameter types with symbols F, S, IUS, …

ID	Name	Symbol	Description
1	**Flow**		**Objects flowing between processes**
	Flow	F	
2	**Stock**		**Objects residing as stocks in processes**
	Stock	S	General stock
	In-use stock	IUS	
	Population	P	
3	**Material/Product property**		**Intensive object properties**
	Lifetime	LT	
	Material composition	MC	
	Share	SHA	
	Price	PR	
	Intensity of use	IU	
	Specific energy consumption	EI	
4	**Process coefficient (intensive)**		**Intensive process properties**
	Yield coefficient	PY	
	Process extension	PE	
	Process factor (per capacity)	PF	
5	**Extensive process property**		**Extensive process properties such as capacity**
	Process capacity	CAP	
6	**General Ratio**		
	Per capita stock/flow	PCS<br>PCF	Any ratio between two system variables from the groups 1-5 above

For example, the ODYM-RECC parameter with ID *4_PY_MaterialProductionRemelting_V2.2* is the yield factor/coefficient of the remelting processes in version 2.2.

5.1. Description of the data gathering process

Data collection in RECC serves several purposes: First, to inventory datasets from the literature so that they can be easily reused by other team members. Second, inventoried datasets shall ultimately become part of the industrial ecology data commons (https://zenodo.org/communities/indecol/), [74]. Third, to link several inventoried datasets from the literature to quantify the different ODYM-RECC model parameters in the project-wide classification. All of these goals necessitate harmonization of data reproducibility and of data conversion. Therefore, we use a comprehensive procedure with standardized data files. Each model parameter will be documented in one data file, while the data can be of different sources.

Data collection in the RECC project happens in the following stages: (cf. also Supplementary Figure 38). The next paragraph provides further details.

1) Identification and inventory of data source: Document reference, web link, data license, dataset version, etc., that are to be recorded in a collection template

2) Data are extracted from original sources in their original resolution and stored in table or list format in the collection template.

3) Data are manually (sometimes via a script) converted to RECC resolution and format

4) Data conversion process is reviewed by assigned data reviewer

Repeat steps 1-4 until parameter dataset is finalized

5) Assign version number to dataset and include it in the RECC project database.

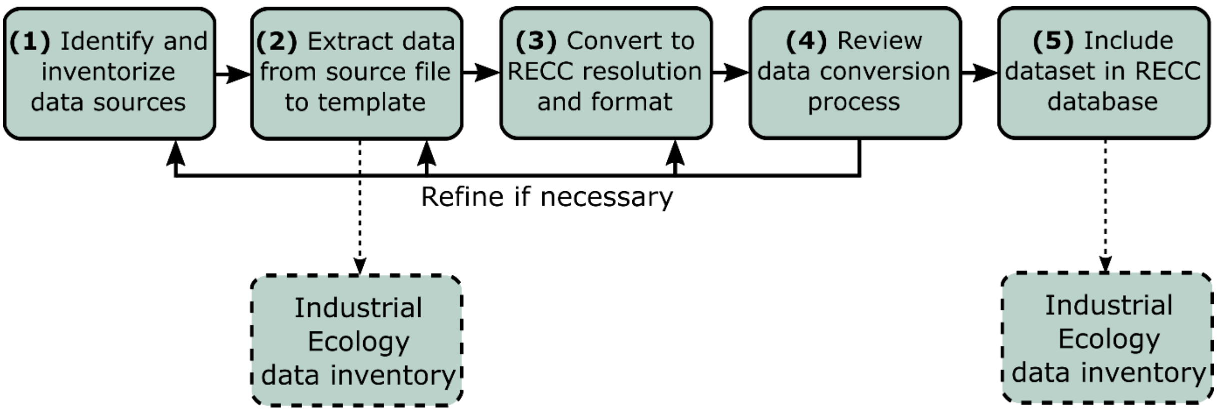

Supplementary Figure 38: RECC data collection scheme.

The data collection steps are described in detail:

1) Identification and inventory of data source: Document reference, web link, data license, dataset version, etc., that are to be recorded in a collection template. For this purpose, each template has a sheet ‘Raw Data’ (you can add more such sheets if necessary), and on this sheet, each raw data set,

from a single number to a larger table, is to be extracted from the literature and described (Supplementary Figure 39). As an alternative, larger datasets can be archived on Figshare or Zenodo, provided that you have permission to do that.

DATASET		
**Item**	**Description**	***Example***
Dataset Name	Serves as description	*Material composition of passenger vehicles, extracted from Hawkins et al. (2013) inventory.*
Dataset version	if any	*none*
Type of data	E.g., stock, flow, lifetime, product (	*product material composition*
Process scope	if any	*none*
Process resolution	if any	*none*
Product scope	if any	*passenger vehicles*
Product resolution	if any	*ICE vehicle, battery electric vehicle, fuel cell vehicle*
Material scope	if any	*metals, polymers, silica material, chemicals, other*
Material resolution	if any	*42 materials*
Regional scope	if any	*global*
Regional resolution	if any	*global*
Temporal scope	if any	*ca. 2000-2010*
Temporal resolution	if any	*ca. 2000-2010*
Semantic string example	"The copper content of average typ	*"The zinc content of average ICE vehicles, ca. 2000-2010 vintage, is 0.0998 kg / unit."*
Keywords	3-5 descriptive keywords	*product material composition; metal content; passenger vehicles*
Data provenance	Expert estimates, mass balance, pı	*industry data*
Type of source	publicly available dataset or report	*supplementary material of journal article*
Dataset format	Select 1 of the following:	*Excel spreadsheet*
Dataset license	if any	*CC-BY*
Main/first author or organisation		*Troy R. Hawkins*
Link to dataset	if any	*https://onlinelibrary.wiley.com/action/downloadSupplement?doi=10.1111%2Fj.1530-9290.2012.00532.x&attachmentId=168199307*
Link to accompanying report/paper	if any	*https://onlinelibrary.wiley.com/doi/abs/10.1111/j.1530-9290.2012.00532.x*
Suggested citation	DOI sufficient	*10.1111/j.1530-9290.2012.00532.x*
Access date	Data on which data were accessed	*04.06.2018*
Entry author	Your name	*Stefan Pauliuk*

Data/Unit/Uncertainty/Comment as tables or list

Up to you how to organise this best!

**Material**	**Unit**	**Vehicle type internal combustion engine**	**Vehicle type battery electric vehicle**	**Vehicle type fuel cell vehicle**	**Uncertainty**
steel	kg/unit	882.0902775	923.3628638	1158.098555	None
iron	kg/unit	115.8742446	20.81181057	20.81181057	None
aluminium	kg/unit	71.2499941	111.8435601	80.6365339	None
copper	kg/unit	23.87236603	67.53263096	38.91757181	None
magnesium	kg/unit	0.240403956	0.240403956	0.240403956	None
zinc	kg/unit	0.099790321	0.099790321	0.099790321	None

Supplementary Figure 39: Raw data inventory in RECC template. Upper section: Project-wide uniform description of metadata, dataset description, and system location. Lower part: Data as table or list in custom formatting.

In the example above the actual data are organized as a table. Another example for the data section of the raw data template is shown in Supplementary Figure 40.

Semantic string example	"The copper content of average typ	*"The aluminium content of average ICE vehicles, ca. 2010 vintage, is xxx kg / unit."*
Keywords	3-5 descriptive keywords	*product material composition; metal content; passenger vehicles*
Data provenance	Expert estimates, mass balance, pı	*industry data*
Type of source	publicly available dataset or report	*supplementary material of journal article*
Dataset format	Select 1 of the following:	*Excel spreadsheet*
Dataset license	if any	*all rights reserved*
Main/first author or organisation		*Roja Modaresi*
Link to dataset	if any	*https://pubs.acs.org/doi/suppl/10.1021/es502930w/suppl_file/es502930w_si_002.xlsx*
Link to accompanying report/paper	if any	*https://pubs.acs.org/doi/10.1021/es502930w*
Suggested citation	DOI sufficient	*10.1021/es502930w*
Access date	Data on which data were accessed	*04.06.2018*
Entry author	Your name	*Stefan Pauliuk*

Data/Unit/Uncertainty/Comment as tables or list

**Age-cohort**	**Car segment**	**Drive technology**	**Total car weight (kg)**	**Wrought Al**	**Cast Al**	**Cast Iron**	**Standard Steel**	**High Strength Steel**
1978	C/Average	Conventional Gasoline	1318.259545	8	19	197.738932	725.0427499	39.54778636
1985	C/Average	Conventional Gasoline	1136.392635	9	27	170.458895	545.4684647	79.54748443
1990	C/Average	Conventional Gasoline	1166.88749	11	34	175.033124	560.1059954	93.35099923
1995	C/Average	Conventional Gasoline	1198.851978	13	42	143.862237	539.48339	107.896678
2000	C/Average	Conventional Gasoline	1237.797215	17	54	123.779722	532.2528025	123.7797215
2005	C/Average	Conventional Gasoline	1272.333558	26	66	101.786685	534.3800942	152.6800269
2008	C/Average	Conventional Gasoline	1300.991374	30	72	104.07931	546.416377	208.1586198
2010	C/Average	Conventional Gasoline	1382.18852	33	76	110.575082	580.5191783	234.9720484

Supplementary Figure 40: Raw data inventory in RECC template, second example. Upper section: Meta data, dataset description, and system location. Lower part: Data as table or list.

Here the data are organised as table with a three-level row index and a single level column index.

While the metadata description follows a fixed form that will facilitate automatic processing later, the numerical data on the sheet 'Raw Data' can be organized at the discretion of the data collectors. Examples for data organisations include 2D tables, tables with multi-indices, list, and combinations thereof.

2) Data are extracted from original sources in their original resolution and stored in table or list format in the collection template. → Cf. point 1 above. There will be cases where this is not possible, e.g. database interfaces that need to be queried, very large databases, GIS data, etc. For the scope of the RECC project, however, dataset sizes were manageable with the given template structure.

3) Data are manually converted to RECC resolution and format

Ancillary calculations such as unit conversions, disaggregation, or aggregation of data are to be documented on the sheet 'Ancillary calculations' that is part of each parameter file. From there the individual parameter values are then copied or linked to the 'Values' table, which may require conversion of data format, data resolution, and data units, as this sheet comes with a pre-defined format common to all ODYM-RECC parameters and in the project-wide resolution. From the Values sheet the are read by the RECC model. Convert all values to the units that are already defined in the 'Units' sheet. On the 'Comment' sheet each individual value can be commented upon. If existent, uncertainties for each individual value should be recorded on the sheet 'Stats_array_string'. The use of stats_array_strings is explained in section 5.3 of the RECC model description. **Note:** You may use links and equations (Cell A = SheetXCellB * SheetYCellC) but make sure that these links only refer to other sheets within the same workbook. No links to other workbooks allowed, numbers from other workbooks need to be copied with the 'value only' option.

4) Data conversion process is reviewed by assigned data reviewer

Based on the review outcome, steps 1-4 are repeated until the parameter dataset is finalized.

5) Assign version number to dataset and include it in the RECC project database.

Once the dataset is regarded as final, it is assigned a version number and added to the RECC project database. All further editing and modification of the data needs to happen as part of a new version of that parameter to keep the database consistent.

5.2. ODYM-RECC parameters, complete list.

This subsection lists all 104 model parameters, their index structure and symbols and the major data sources. For each parameter, the complete list of literature data sources is contained in the 'ref' sheet of each parameter file. For the scenario parameters obtained from the target table interpolation, the data sources and assumptions are listed in the transport and building model documentations [76,77]. Additional information about parameter compilation can be found in the data log files under https://github.com/YaleCIE/RECC-data, where the compilation of the assumption and formatting of the data templates is documented.

5.2.1. Socioeconomic parameters

The ODYM-RECC basic scenario drivers population, PPP-GDP, and urbanisation are listed in Supplementary Table 14. Population and GDP were downloaded from the IIASA SSP database and brought to ODYM format. They are given for four aspects: time t, region r, SSP scenario S, and SSP Population/GDP model T.

Supplementary Table 14: The ODYM-RECC basic scenario drivers. The aspect/indices are introduced in the index table (Supplementary Table 9). For the scope of the parameters, we distinguish between historic or present base data (**H**), future scenario (**S**), future potentials (**P**), implementation of future potential (**I**).

Name	Symbol(s) and indices, dataset ID	Unit, Scope	Explanation/Example/Source (S)
Population	$P(M,t,\mathrm{r},\mathrm{S})$<br>2_P_RECC_Population_SSP_32R_V2.2	Million<br>**Scope:** H,S	**Ex:** P (2015, China, SSP1, IIASA-Pop) = 12300000<br>[78]
PPP-GDP	**(currently not used)**	Billion US$2005<br>**Scope:** H,S	**Ex:** GDP(2015, China, SSP1, IIASA-Pop) = 3450 bn Int$<br>[78]
Urbanisation rate	**(currently not used)**	%<br>**Scope:** H,S	**Ex:** U (2015, China, SSP1, IIASA-Pop) = 0.1<br>[78]

The ODYM-RECC socioeconomic parameters for the use phase are listed in Supplementary Table 15:

Supplementary Table 15: The ODYM-RECC basic parameters, use phase. Those that are affected by resource efficiency strategies are labelled in bold face. The indices are introduced in the index table (Supplementary Table 9). For the scope of the parameters, we distinguish between historic or present base data (**H**), future scenario (**S**), future potentials (**P**), implementation of future potential (**I**).

Name	Symbol(s) and indices	Unit, Scope	Explanation/Example/Source (S)
In use stock 2015	$S_0,\ S_{2015}(2015,\mathrm{c},p/B,r)$<br>2_S_RECC_FinalProducts_2015_passvehicles<br>2_S_RECC_FinalProducts_2015_resbuildings<br>2_S_RECC_FinalProducts_2015_nonresbuildings	Building stock: Million m^2<br>Vehicle stocks: 1<br>**Scope:** H	Starting value for In-use stock of buildings, infrastructure, and products. **Ex:** Stock(Residential buildings 4 storeys, 2010, China,) = 123 M m^2.<br>**(S):** Large number of sources, mostly international and national statistics and journal articles, listed in model files.
**Future stock levels**	$S_{fut}(t,\mathrm{r},G,\mathrm{S})$<br>2_S_RECC_FinalProducts_Future_resbuildings<br>2_S_RECC_FinalProducts_Future_NonResBuildings<br>2_S_RECC_FinalProducts_nonresbuildings_g (global aggregate)	Building stock: m^2/cap<br>**Scope:** S	Time series for future residential building stock per capita. **Ex:** FuturepCStock(Residential buildings, 2030, China, SSP1) = 30 m^2/cap<br>**(S):** Scenario target table
Future inflows	$F_{fut}(a,c,o,\mathrm{S},R)$<br>$F_{fut}(I,c,l,\mathrm{S},R)$<br>1_F_RECC_FinalProducts_appliances<br>1_F_RECC_FinalProducts_industry	Appliances: items/yr, industry: prod./ generation capacity /yr, e.g., GW/yr<br>**Scope:** S	Time series for future inflow of appliances and industry assets (here: electricity generation). Ex: F_fut(air conditioning units,2030,global,SSP1,RCP2.6) = 15 Million.<br>**(S):** Scenario target table
**Future passenger vehicle mobility**	$FUNCT_{exog\_fut}(t,\mathrm{r},G,\mathrm{S})$<br>1_F_Function_Future	Passenger-km/yr and per person<br>**Scope:** S	Time series for future passenger vehicle kilometres per person. **Ex:** PassVehkm(2030, USA, SSP1) = 20000<br>**(S):** Scenario target table
**Product lifetime**	$\tau(pr),\ \tau(Bcr)$<br>3_LT_RECC_ProductLifetime_passvehicles<br>3_LT_RECC_ProductLifetime_resbuildings<br>3_LT_RECC_ProductLifetime_NonResbuildings<br>3_LT_RECC_ProductLifetime_appliances<br>3_LT_RECC_ProductLifetime_industry	yr<br>**Scope:** H,S	Mean product lifetime, by cohort, product group, region, and scenario. **Ex:** Lifetime(Residential buildings 4 storeys, China, SSP1,2050) = 80yr<br>**(S):** previous work: [36,37,79]

	3_LT_RECC_ProductLifetime_nonresbuildings_g (global aggr.)		
**Intensity of operation, use phase**	$IO(V,r,t,S)$, $IO(c,B,V,r,S)$<br>3_IO_Buildings_UsePhase_Historic<br>3_IO_Buildings_UsePhase_Future_Heating<br>3_IO_Buildings_UsePhase_Future_Cooling<br>3_IO_NonResBuildings_UsePhase_V1.0<br>3_IO_Vehicles_UsePhase	Buildings: % of area that is heated or cooled, vehicles: km/yr<br>**Scope:** S	Denotes how intensively a product is used, e.g., how many km/yr a vehicle is driven. **Ex:** Share of residential building area that is heated (Germany, 2020, SSP1) = 96%.<br>**(S):** Historical data from a number of sources, cf. parameter files, 2015 values extrapolated into the future.
**Type split**	$TS(G,p,t,r,S)$,<br>$TS(G,B,r,t,S)$<br>3_SHA_TypeSplit_Buildings<br>3_SHA_TypeSplit_NonResBuildings<br>3_SHA_TypeSplit_Vehicles	1 (%), share of 6 vehicle and 9 building types in total new vehicles and buildings, resp.<br>**Scope:** S	Parameter splits good flows into different types of one good. **Ex:** Share of low energy multi-family houses in total new construction (Germany, 2020, SSP1) = 36%.<br>**(S):** Vehicles: IEA results [30], buildings: scenario target table.
**Vehicle occupancy rate**	$OR(G,r,t,S)$<br>6_MIP_VehicleOccupancyRate	1<br>**Scope:** S	Average occupancy (persons per vehicle) **Ex:** Vehicle occupancy (passenger vehicles, USA, 2015, SSP1) = 1.6.<br>**(S):** Transport model documentation
**Calibration parameter**	6_PR_Calibration	1	For each country/region, the calibration factors scale the 2015 and future vehicle annual kilometrage, the average MJ/km driven, and the average MJ/m^2/yr of building energy intensity to match national energy statistics.
**Obsolete Stock formation rate**	**(currently not used)**	1<br>**Scope:** H,S	Share of EoL products that pile up as obsolete stocks. **Ex:** Obsolete stocks (buildings residential, 2020, China, SSP1) = 20% (of all EoL buildings)

5.2.2. Technology parameters

The ODYM-RECC technology parameters for the use phase are listed in Supplementary Table 16:

Supplementary Table 16: The ODYM-RECC technology parameters, use phase. Those that are affected by resource efficiency strategies are labelled in bold face. The indices are introduced in the index table (Supplementary Table 9). For the scope of the parameters, we distinguish between historic or present base data (**H**), future scenario (**S**), future potentials (**P**), implementation of future potential (**I**).

Name	Symbol(s) and indices	Unit, Scope	Explanation/Example/Source (S)
**Product material composition**	$\mu(c,m,p/B,r)$<br>3_MC_RECC_Buildings<br>3_MC_RECC_NonResBuildings<br>3_MC_RECC_Vehicles<br>3_MC_RECC_Nonresbuildings_g (global aggr.)<br>3_MC_RECC_industry<br>3_MC_RECC_appliances	**kg/item, kg per m2,**<br><br>**Scope:** H	Material composition of products in regions and from age-cohorts (share of different engineering materials in goods). **Ex:** MaterialComposition(2040, EU, road infrastructure, asphalt) = 540 ton/km<br>**(S):** Only used for historic age-cohorts, various sources as documented in parameter files.<br>Future: Mix of different archetypes
Material demand of renovation	$\mu Rabs(c,m,B,r)$<br>$\mu Rrel(c,m,B,r)$<br>3_MC_RECC_Buildings_Renovation_Relative	**1, kg per m2,**<br><br>**Scope:** S	Material demand of building renovation, both in % of existing 3_MC (currently not used) and in absolute terms. **Ex:** μR(MFH,1930 cohort, USA, wood) = 12 kg/m2.<br>**(S):** Literature values from case studies.

	3_MC_RECC_Buildings_Renovation_Absolute		
**Product specific energy consumption**	$EI(c, p / B, V, n, r, S)$ 3_EI_Products_UsePhase_passvehicles 3_EI_Products_UsePhase_resbuildings 3_EI_Products_UsePhase_nonresbuildings	**MJ/km, MJ/m2/yr,** **Scope:** H	Specific operational energy consumption of products in regions and from age-cohorts. **Ex:** SpecEnergyConsumption(2000, USA, Internal Combustion Engine, gasoline (ICEG), Driving, all energy carriers, SSP1) = 2.7 MJ/km **(S):** Only used for historic age-cohorts, various sources as documented in parameter files. Future: Mix of different archetypes
Energy carrier split of products	$ECS(c, p, o, V, n, S)$, $ECS(V, R, r, n, t)$ 3_SHA_EnergyCarrierSplit_Buildings 3_SHA_EnergyCarrierSplit_NonResBuildings 3_SHA_EnergyCarrierSplit_Vehicles	**% (1)** **Scope:** H,S	Parameter that split the total energy consumption for operating buildings/vehicles into the individual energy carriers. **Ex:** EnergyCarrierSplit(2015, Hybrid Electric Vehicles (HEV), world, Driving, SSP1, gasoline) = 1 **(S):** Vehicles: by definition of vehicle type (drive technology dictates energy carrier), buildings: IEA parameter, [32]
Energy conversion efficiency buildings, historic	$BEChist(V, R, r, n, t)$ 4_TC_ResidentialEnergyEfficiency_Default	**% (1)** **Scope:** H	Energy conversion efficiency from useful energy delivered by building function to final energy delivered into building to. Current values. **Ex:** BEChist(Heating,OtherEU15,fuel wood, 2015) = 2.5. **(S):** Literature values from case studies.
Energy conversion efficiency buildings, future	$BECfut(V, R, r, n, t, S)$ 4_TC_ResidentialEnergyEfficiency_Scenario_Heating 4_TC_ResidentialEnergyEfficiency_Scenario_Cooling	**% (1)** **Scope:** S	Energy conversion efficiency from useful energy delivered by building function to final energy delivered into building to. Current values. **Ex:** BEChist(Heating,OtherEU15,fuel wood, 2050) = 0.4. **(S):** Scenario target table.
Material composition of archetypes	$MA(A, r, m)$, $MA(A, m)$ 3_MC_BuildingArchetypes 3_MC_VehicleArchetypes 3_MC_NonResBuildingArchetypes	**Mt/yr** **Scope:** P	Material composition of product archetypes. **Ex:** MA(USA, single-family house, standard design, cement) = 48,4 kg/m^2. **(S):** Building simulation module, Vehicle simulation module
Specific energy consumption of archetypes	$EIA(A, n)$, $EIA(A, V, r, n)$ 3_EI_BuildingArchetypes 3_EI_NonResBuildingArchetypes 3_EI_VehicleArchetypes	**Mt/yr** **Scope:** P	Specific energy consumption (use phase) of product archetypes. **Ex:** MA(ICEV-g_Minivan/SUV_Lightweight design, all energy carriers) = 2,4 MJ/km. **(S):** Building simulation module, Vehicle simulation module
Maximum building renovation potential	$MRP(r, c, B)$ $MRP(r, c, N)$ 3_SHA_MaxRenovationPotential_ResBuildings 3_SHA_MaxRenovationPotential_NonResBuildings	**%** **Scope:** P	Share of 2015 stock of resbuildings that can be renovated **Ex:** MRP(Germany,1960 cohort, office building standard) = 0.9. (Means that 90% of this cohort segment are available for renovation) **(S):** In line with Bürger et al. [80], DOI 10.1007/s12053-018-9660-6
Energy saving under building renovation	$ESP(r, S, B)$ $ESP(r, S, N)$	**%** **Scope:** P	Reduction in specific energy consumption of resbuildings, in %.

	3_SHA_EnergySavingsPot_Renovation_ResBuildings<br>3_SHA_EnergySavingsPot_Renovation_NonResBuildings		**Ex:** ESP(Germany,SSP1, office building standard) = 0.6. (Means that the specific energy consumption of this cohort segment can be reduced BY 60% (not down to 60%)<br>**(S):** In line with Bürger et al. [80], DOI 10.1007/s12053-018-9660-6
Implementation curve for building renovation	$ICBR(R,o,t,S)$<br>3_SHA_BuildingRenovationScaleUp	**1 (%)**<br>**Scope: S**	Curves that contain the ramp-up of the remaining building renovation potential in the 2015 stock.<br>Not implemented: Curves = 0.<br>**Ex:** ICBR(RCP2.6,World,2050,LED) = 1. (Means that in 2050, for the given scenario, the entire renovation potential indicated by *MRP(r,c,B)* will have been renovated to achieve the energy savings denoted by *ESP (r,S,B)*.<br>**(S):** Scenario target table, storyline extension.

The ODYM-RECC technology parameters for the material cycles are listed in Supplementary Table 17:

Supplementary Table 17: The ODYM-RECC technology parameters, material cycles. Those that are affected by resource efficiency strategies are labelled in bold face. The indices are introduced in the index table (Supplementary Table 9). For the scope of the parameters, we distinguish between historic or present base data (**H**), future scenario (**S**), future potentials (**P**), implementation of future potential (**I**).

Name	Symbol(s) and indices	Unit, Scope	Explanation/Example/Source (S)
**Fabrication yield**	$\lambda(m,w,g,F,t,o)$<br>4_PY_Manufacturing	**1 (%)**<br>**Scope:** H,S	Fabrication yield of materials into products, **Ex:** FabYield(steel, car manufacturing, 2010) = 78%.<br>**(S):** Previous work: [36,37,81]
**Remelting yield**	$RMY(w,m,e,W,t,o)$<br>4_PY_MaterialProductionRemelting	**1 (%)**<br>**Scope:** H,S	Remelting yield of scrap into secondary metals, **Ex:** RemYield(steel scrap, steel) = 97%.<br>**(S):** Previous work: [79,82] and industry information.
**Scrap recovery efficiency**	$\varphi(g,o,m,w,W)$<br>4_PY_EoL_RecoveryRate	**1 (%)**<br>**Scope:** H,S	Efficiency of recovering scrap w' from broad waste group w. **Ex:** RecEff(Copper, to Copper scrap, from E-Waste, EU) = 70%.<br>**(S):** Previous work: [36,37,81]
Elemental composition of materials, historic stocks	$MCe(m,e)$<br>3_MC_Elements_Materials_ExistingStock	**1 (%)**<br>**Scope:** H	Iron content of construction steel in historic (2015) stock is 0.999.<br>**(S):** Assumption, typical value
Elemental composition of materials, primary production	$MCp(m,e)$<br>3_MC_Elements_Materials_Primary	**1 (%)**<br>**Scope:** S	Al content of primary wrought aluminium is 0.99.<br>**(S):** Assumption, typical value

The ODYM-RECC basic parameters for the industry background and environmental mechanisms are listed in Supplementary Table 18:

Supplementary Table 18: The ODYM-RECC material cycle parameters, industry background and environmental mechanisms. Those that are affected by resource efficiency strategies are labelled in bold face. The indices are introduced in the index table (Supplementary Table 9). For the scope of the parameters, we distinguish between historic or present base data (**H**), future scenario (**S**), future potentials (**P**), implementation of future potential (**I**).

Name	Symbol(s) and indices	Unit, Scope	Explanation/Example/Source (S)
Process energy demand, manufacturing	$EIM(F,n,c,o)$<br>4_EI_ManufacturingEnergyIntensity	**MJ/item, MJ/m2**<br>**Scope:** H,S	Per unit-of-output energy demand of the different processes. **Ex:** Ext(Electricity demand, aluminium smelting, Brazil, 2020) = 13.5 GJ/ton<br>**(S):** Ecoinvent mostly
Process energy demand, primary production	$EIP(P,n,c,o)$<br>4_EI_ProcessEnergyIntensity	**MJ/ton**<br>**Scope:** H,S	Per unit-of-output energy demand of the different processes. **Ex:** Ext(Electricity demand, aluminium smelting, Brazil, 2020) = 13.5 GJ/ton<br>**(S):** Currently not used, as all primary production energy consumption is indirectly accounted for in the GHG parameter 4_PE_ProcessExtensions_V3.3.
Process energy demand, waste management	$EIW(w,n,c,o)$<br>4_EI_WasteMgtEnergyIntensity	**MJ/item, MJ/m2**<br>**Scope:** H,S	Per unit-of-output energy demand of the different processes. **Ex:** Ext(Electricity demand, aluminium smelting, Brazil, 2020) = 13.5 GJ/ton<br>**(S):** Ecoinvent mostly
Process energy demand, remelting	$EIRM(m,n,c,o)$<br>4_EI_RemeltingEnergyIntensity	**MJ/item, MJ/m2**<br>**Scope:** H,S	Per unit-of-output energy demand of the different processes. **Ex:** Ext(Electricity demand, aluminium smelting, Brazil, 2020) = 13.5 GJ/ton<br>**(S):** Ecoinvent mostly
Direct emissions	$GHGD(X,n)$<br>6_PR_DirectEmissions	**kg CO2-eq / MJ**<br>**Scope:** Constant	Direct GHG emissions of energy carrier combustion. **Ex:** GHGD(GWP100, Diesel) = 0.07 kg/MJ<br>**(S):** Standard values, recorded by Modaresi et al. [26]
Primary production GHG emissions	$GHGPP(P,X,o,t,S)$<br>4_PE_ProcessExtensions	**kg CO2-eq / kg**<br>**Scope:** S	Supply chain GHG emissions of primary material production. **Ex:** GHGPP(GWP100, aluminium smelting, 2040, SSP1) = 4.8 ton CO2eq /ton<br>**(S):** Scenario calculations with ecoinvent, scenario target table.
Energy and electricity supply GHG intensity, by region	$GHGE(X,n,S,R,r,t)$<br>4_PE_GHGIntensityEnergySupply	**t/GJ**<br>**Scope:** S	GHG intensity of energy supply. **Ex:** GHG(electricity, Soth-East Asia, 2040) = 4 kg CO2-eq /GJ<br>**(S):** MESSAGE IAM SSP model runs
Energy and electricity supply GHG intensity, global	$GHGW(X,n,S,R,o,t)$<br>4_PE_GHGIntensityEnergySupply_World	**t/GJ**<br>**Scope:** S	GHG intensity of energy supply. **Ex:** GHG(electricity, World, 2040) = 4.2kg CO2-eq /GJ<br>**(S):** MESSAGE IAM SSP model runs
Electricity supply GHG intensity, global backstop	$GHGBS(X,n,S,R,t)$<br>4_PE_GHGIntensityElectricitySupply_Backstop	**t/GJ**<br>**Scope:** S	GHG intensity of energy supply. **Ex:** GHG(electricity, World, 2050) = 20g CO2-eq / MJ<br>**(S):** [83]
Electricity generate per mass of wood waste burned	$ElWood(w,W,n)$<br>4_PE_ElectricityFromWoodCombustion	**GJ/t**<br>**Scope:** Constant	Electricity generate per mass of wood waste burned. **Ex:** ElWood(electricity, wood waste, waste mgt. industry) = 6.174 GJ/ton<br>**(S):** Literature value
Global warming potential of biomass storage	$GWPbio(c)$<br>6_MIP_GWP_Bio	**kg CO2-eq / kg**<br>**Scope:** S	Climate impact of biomass storage (in use phase) with subsequent incineration **Ex:** GWPbio(80) = -0.7 t CO2-eq / t<br>**(S):** [84]
Forest rotation period fuelwood	$FRPfuel(n)$<br>3_LT_ForestRotationPeriod_FuelWood	**yr**<br>**Scope:** Constant	Forest rotation period for timber is 20 yr.<br><br>**(S):** typical value

Forest rotation period timber	$FRPtimber(m)$ 3_LT_ForestRotationPeriod_Timber	**yr** **Scope:** Constant	Forest rotation period for timber is 75 yr. **(S):** typical value
CO2 per wood combusted	$CO2wood(X,m)$ 3_MC_CO2FromWoodCombustion	**1** **Scope:** Constant	CO2 per wood combusted = 1.83 kg (CO2) / kg (Wood) **(S):** typical value, stoichiometry
Heating value of wood per unit of carbon	$HHVwood(e,n)$ 3_EI_HeatingValueWoodPerCarbon	**MJ/kg** **Scope:** Constant	Higher heating value of (n=fuel wood) per (e = carbon) = 25 MJ/kg **(S):** typical value, thermal property
Cement content of concrete	$CCC(m,m^*)$ 3_MC_CementContentConcrete	**1** **Scope:** Constant	Content of (m=cement) in (m*=concrete) = 13% **(S):** typical value (Shanks et al. (2019))
CO_2 price index	**3_PR_RECC_CO2Price_SSP_32R (currently not used)**	US$2005/ton **Scope:** S	**Ex:** CO2Price(R32BRA, 2050, SSP4, RCP2.6) = 185 US$w005/ton
Characterisation factors for env. Mechanisms	$CF(x,X)$ 6_MIP_CharacterisationFactors	**Misc. Units** **Scope:** Constant	Characterisation factors for env. Mechanisms. **Ex**: GWP500 of N2O emissions per main output is 156 kg CO2-eq per kg. **(S):** Literature values

5.2.3. Resource efficiency parameters

The ODYM-RECC resource efficiency parameters are listed in Supplementary Table 19: The defining equations for these parameters are introduced in section 6 or in the transport model documentation.

Supplementary Table 19: The ODYM-RECC resource efficiency parameters. The indices are introduced in the index table (Supplementary Table 9). Some data sources are given directly below, but for some parameters, multiple data sources and assumptions were used, and some of the data sources link to the IEDC (http://www.database.industrialecology.uni-freiburg.de/), see the parameter files.

Name and strategy	Symbol(s), Unit	Explanation, reference case if not implemented	Example and data source
**Implementation curves of RE strategies** (cf. section 6.3.3)	$IC(R,o,t,S)$ Unit: 1 (%) 3_SHA_RECC_REStrategyScaleUp	Functions that contain the ramp-up of different resource efficiency potentials defined below. Not implemented: Curves = 0.	Complete ramp-up until 2040 assumed of a selected RES strategy is on. **(S):** SSP-consistent assumptions, scenario target tables.
**MIU: maximal MIU potential buildings**	$\Phi_{MIU}(G,o,S)$ 2_S_RECC_FinalProducts_Future_resbuildings_MIUPotential 2_S_RECC_FinalProducts_Future_nonresbuildings_MIUPotential	More service per unit of stock. Stock is reduced as a sufficiency strategy or following other, not considered economic or social incentives. Maximal reduction potential (share of existing stock) for more intense use of residential building (reduction of per capita floor space). Applies to buildings only. Not implemented: stock in m^2/cap not changed.	Reduce SSP1 and SSP2 stock levels by up to 20% in 2040, (smoothed linear ramp), but not lower than LED values. Implementation curves to not apply here! PHI(res. Buildings, World, SSP1) = 20% **(S):** SSP-consistent assumptions
**CaS: Car ownership change under cars-haring** (cf. transport model docu)	$COS(S,r)$ Unit: 1 6_MIP_CarSharing_Stock	Ratio of car ownership rate with vs. without participation in car-sharing	COS(SSP1,France) = 0.5 **(S):** Cf. transport model documentation

**RiS: Occupancy rate change under ride-sharing**<br>(cf. transport model docu)	$ORS(S,r)$ Unit: 1<br>6_MIP_RideSharing_Occupancy	Ratio of car occupancy with vs. without participation in ride-sharing: Global study: this parameter = 1, as the occupancy rate increases by 1 under ride-sharing (absol. increase)<br>Case study Germany: Relative factor, previous OR increases by factor 1.4.	ORS(SSP1,France) = 1.4<br>**(S):** Cf. transport model documentation
**Car-sharing**<br>(cf. transport model docu)	$CaS(G,o,t,S)$<br>Unit: 1 (%)<br>6_PR_CarSharingShare	Share of total passenger-vehicle-based passenger km that is delivered by shared cars<br>Not implemented: Parameter = 0.	SSP-consistent assumptions, scenario target tables. Up to 30%. For justification cf. the transport model docu!<br>Implementation curves to not apply here!<br>**(S):** SSP-consistent assumptions, scenario target tables.
**Ride-sharing**<br>(cf. transport model docu)	$RiS(G,o,t,S)$<br>Unit: 1 (%)<br>RiS<br>6_PR_RideSharingShare	Share of total passenger-vehicle-based passenger km that is delivered by shared rides (several persons sharing one car)<br>Not implemented: Parameter = 0.	SSP-consistent assumptions, scenario target tables. Up to 30%. For justification cf. the transport model docu!<br>Implementation curves to not apply here!<br>**(S):** SSP-consistent assumptions, scenario target tables.
**ULD: Product Downsizing**<br>(cf. transport and building model docu)	$DS(s,r,t,S)$, $DS(u,r,t,S)$<br>Unit: 1 (%)<br>3_SHA_DownSizing_Vehicles<br>3_SHA_DownSizing_Buildings<br>3_SHA_DownSizing_NonResBuildings	Share of new passenger vehicles/buildings that are built with leaner design (buildings) or of a smaller segment (vehicles). Mix of standard and down-sized archetypes	Share of lean-design buildings in UK, 2040, SSP1 is 65%.<br>**(S):** SSP-consistent assumptions, scenario target tables.<br>Implementation curves do not apply.
**ULD: Product Downsizing**<br>Direction of vehicle downsizing	$VDSD(r,S)$<br>X_FLAG_VehicleDownsizingDirection	**Unit: Bool.**<br>ULD for vehicles is modeled as segment shift. Depending on socioeconomics (r,S), the shift in the scenarios leads to smaller vehicles (like for US SSP1) or to larger vehicles (like India SSP2) on average.	This parameter then indicates which setting (2015) or future is the baseline for “no ULD”.<br>VDSD(India,SSP1) = True.<br>**(S):** Inspection of model results.
**MSu: Product Light-weighting**<br>(cf. transport and building model docu)	$MS(G,r,t,S)$, $MS(p,r,t,S)$<br>3_SHA_LightWeighting_Buildings<br>3_SHA_LightWeighting_NonResBuildings<br>3_SHA_LightWeighting_Vehicles	Share of new passenger vehicles/buildings that are built lighter by substituting materials. Mix of standard and down-sized archetypes	Share of material-substituted buildings in UK, 2040, SSP1 is 65%.<br>**(S):** SSP-consistent assumptions, scenario target tables.<br>Implementation curves do not apply.
**MSu: Reduction of cement content of concrete**	$CCCred(m)$<br>3_MC_CementContentConcrete	**Unit: 1**<br>**Scope:** Constant	Reduction potential for content of (m=cement) in concrete = 15%<br>**(S):** typical value (Shanks et al. (2019))
**LTE: Product lifetime extension**	$LTE(p,o,S)$, $LTE(B,r,S)$<br>Unit: 1 (%)<br>6_PR_LifeTimeExtension_passvehicles<br>6_PR_LifeTimeExtension_resbuildings<br>6_PR_LifeTimeExtension_nonresbuildings<br>6_PR_LifeTimeExtension_nonresbuildings_g (global aggregate)<br>6_PR_LifeTimeExtension_appliances<br>6_PR_LifeTimeExtension_industry	Longer product life.<br>Not implemented: Lifetimes stays at given value	**(S):** 90% for res. Buildings, 20 % for pass. Vehs., following Milford et al. (2013), DOI: 10.1021/es3031424<br>Implementation curves apply.

**Obsolete stock formation reduction**	Unit: 1 (%)	Fewer products going to obsolete stocks.	Not considered!
**ReU: Re-use of products and their components**	$ReU(m,B,o)$, $ReU(m,p,r,t,S)$<br>Unit: 1 (%)<br>Vehicles:<br>6_PR_ReUse_Veh<br>Buildings:<br>6_PR_ReUse_Bld<br>6_PR_ReUse_nonresBld	Share of materials in end-of-life products that gets reused or remanufactured without undergoing recycling<br>Not implemented: Re-use flow = 0	All vehicle values from scenario target table, documentation in transport model docu.<br>Buildings:<br>**(S):** Up to 29% for construction steel, following Milford et al. (2013), DOI: 10.1021/es3031424<br>Up to 27% for concrete (in concrete elements), estimated from Shanks et al. (2019), DOI 10.1016/j.resconrec.2018.11.002<br>Implementation curves apply.
**EoL: EoL recovery rate improvement**	$EoL(G,o,m,w,W)$<br>Unit: 1 (p.p.)<br>6_PR_EoL_RR_Improvement	Improvement of current EoL recovery rates of postconsumer scrap from EoL products entering waste mgt.	26 p.p. for automotive steel, 2 p.p. for cast iron, 8 p.p. for Al, 15 p.p. for Cu.<br>Implementation curves apply.<br>**(S):** Two main sources: Cullen Sankey work 2012 and World Steel Bulleting, for details cf. parameter files.
**FYI: Fabrication yield improvement**	$FYI(m,g,o,S)$<br>Unit: 1 (p.p.)<br>6_PR_FabricationYieldImprovement	Improvement of current fabrication yield loss rates	**(S):** 10 p.p. for automotive steel, following DOI 10.1021/es3031424<br>1.5 p.p. for concrete in construction, following DOI 10.1016/j.resconrec.2018.11.002<br>Implementation curves apply.
**FSD: Fabrication scrap diversion**	$FSD(m,w,o,S)$<br>Unit: 1 (p.p.)<br>6_PR_FabricationScrapDiversion	Share of fabrication scrap that is diverted into other manufacturing sectors instead of being remelted.	**(S):** Up to 80% of automotive steel fabrication scrap can be diverted, following DOI 10.1021/es3031424<br>Implementation curves apply.

The list below provides a summary of which parameters relate to the different material efficiency strategies. A number of strategies is modelled as technical potentials scaled up by an implementation curve, which is defined in the parameter **3_SHA_RECC_REStrategyScaleUp:** This parameter quantifies the extent to which a given industry RE strategy will be implemented (%). It applies to all industry RE strategy parameter and is dependent on time t, socioeconomic scenario S, and climate policy scenario R. In the current implementation (ODYM-RECC v2.4), a linear increase of the scale-up curve from 0% in 2019 to 100% in 2040 is assumed, followed by a splint interpolation to reduce the changes in the first derivative. This curve is applied to all regions and climate policy scenarios.

- **ULD: Using less material by design, reduction of cement content only.** *[3_SHA_RECC_REStrategyScaleUp, 3_SHA_CementContentReduction]*
- **LTE: Lifetime extension** *[3_SHA_RECC_REStrategyScaleUp, 6_PR_LifeTimeExtension_passvehicles, 6_PR_LifeTimeExtension_resbuildings, 6_PR_LifeTimeExtension_nonresbuildings, 6_PR_LifeTimeExtension_nonresbuildings_g, 6_PR_LifeTimeExtension_appliances, 6_PR_LifeTimeExtension_industry]*
- **ReU: Re-use, residential and non-residential buildings only** *[3_SHA_RECC_REStrategyScaleUp, 6_PR_ReUse_Bld,* 6_PR_ReUse_nonresBld*]*
- **FYI: Fabrication scrap reduction *(yield improvement)*** *[3_SHA_RECC_REStrategyScaleUp, 6_PR_FabricationYieldImprovement]*
- **EoL: Improved recovery efficiency of scrap from end-of-life (EoL) products** *[3_SHA_RECC_REStrategyScaleUp, 6_PR_EoL_RR_Improvement]*
- **FSD: Fabrication scrap diversion** *[3_SHA_RECC_REStrategyScaleUp, 6_PR_FabricationScrapDiversion]*

5.3. Numerical data, units, and uncertainty in ODYM-RECC

Numerical data are stored as float type, usually in *numpy* arrays, where each data aspect spans one array dimension.

Each system variable and each model parameter has a unit, and this unit is specified in the parameter files (either as global unit or for each individual value), and in the model code (for system variables). **Note:** At this development stage, the software does not verify the correct application of units, this is up to the model user. Special attention needs to be kept in situations where a single parameter has mixed units, e.g., the material composition of products, which is measured in kg/item for vehicles and kg/m^2 for buildings.

In the ODYM-RECC model **uncertainty of numerical values** is recorded using stats_array strings, a concept developed by Chris Mutel (http://stats-arrays.readthedocs.io/en/latest/). The type of uncertainty information for a numerical value is coded via Supplementary Table 20, and the parameters (if any) for each type are defined in the subsequent colums loc, scale, shape, min, and max.

Supplementary Table 20: The stats_array coding system. Taken from http://stats-arrays.readthedocs.io/en/latest/ and extended.

Name	ID	loc	scale	shape	min	max
undefined	**0***	static value				
No uncertainty	**1**	static value				
Lognormal	**2**	mu	sigma			
Normal	**3**	mu	sigma			
Uniform	**4**				min	max
Triangular	**5**	mode			min	max
Bernoulli	**6**	p			lower bound	upper bound
Discrete uniform	**7**				min	upper bound
Weibull	**8**	offset	lambda	k		
Gamma	**9**	offset	theta	k		
Beta	**10**	alpha	upper bound	beta		
Generalized extreme value	**11**	mu	sigma	xi		
Student's T	**12**	median	scale	nu		
low-mean-high	**13**	low	high			
	**14**					
	**15**					

*) or just ‘none’

The current list is available under:

http://www.database.industrialecology.uni-freiburg.de/uncertainty.aspx

In ODYM RECC the uncertainty information is coded as a string in the following format:

`'ID;loc;scale;shape;min;max'`

Empty fields are filled with 'none' or 'None'. References to the numerical value given for the data item are denoted with 'value' or 'Value'.

For example, a normally distributed value of mean 10 and standard deviation 1.5 has the stats_array string `'3;10;1.5;none;none;none'`.

A value with undefined or unkown uncertainty has the stats_array_string `'0;Value;none;none;none;none'`.

A uniformly distributed data item with lower bound 0 and upper bound 1 is denoted by `'4;none;none;none;0;1'`.

A value for which low and high alternatives are given is characterized by

`'13;low;high;none;none;none'` (where both high and low alternative are present),

`'13;low;none;none;none;none'` (where only low alternative is present),

`'13;none;high;none;none;none'` (where only high alternative is present),

`'13;0.8*value;1.2*value;none;none;none'` (where high and low alternative are taken as 80% and 120% of the given value).

5.4. ODYM-RECC parameter list, version numbers, and rationales

The following Supplementary Table 21 lists the 104 ODYM-RECC v2.4 parameters introduced above, lists the version and aspects used as well as the unit, and provides a rationale for the central parameters and points to the individual parameter files. For the scenario parameters obtained from the target table interpolation, the data sources and assumptions are listed in the transport and building model documentations [76,77].

Supplementary Table 21: The ODYM-RECC parameter list. Left: Parameter name // version number // aspect structure (cf. Table 9) // unit. Right: reference to parameter and (if applicable) rationale of parameter choice.

ODYM-RECC parameter	Reference and (if applicable) rationale of parameter choice
2_P_RECC_Population_SSP_32R<br>V2.2<br>MtrS<br>Million	See the ODYM-RECC v2.4 parameter file<br>2_P_RECC_Population_SSP_32R_V2.2.xlsx for details.<br>https://doi.org/10.5281/zenodo.4671643
2_S_RECC_FinalProducts_2015_<br>passvehicles<br>V1.3<br>tcpr<br>vehicles: million units. buildings:<br>billion m2	See the ODYM-RECC v2.4 parameter file<br>2_S_RECC_FinalProducts_2015_passvehicles_V1.3.xlsx for details.<br>https://doi.org/10.5281/zenodo.4671643

2_S_RECC_FinalProducts_2015_resbuildings<br>V1.2<br>tcBr<br>vehicles: million units. buildings: billion m2	See the ODYM-RECC v2.4 parameter file 2_S_RECC_FinalProducts_2015_resbuildings_V1.2.xlsx for details.<br>https://doi.org/10.5281/zenodo.4671643
2_S_RECC_FinalProducts_2015_nonresbuildings<br>V1.0<br>tcNr<br>vehicles: million units. buildings: million m2	See the ODYM-RECC v2.4 parameter file 2_S_RECC_FinalProducts_2015_nonresbuildings_V1.0.xlsx for<br>https://doi.org/10.5281/zenodo.4671643
1_F_Function_Future<br>V1.2<br>GrtS<br>inhabitant*m2*yr/yr and passenger-km/yr	See the ODYM-RECC v2.4 parameter file 1_F_Fuction_Future_V1.2.xlsx for details.<br>https://doi.org/10.5281/zenodo.4671643<br>Transport function (passenger vehicle operation) to be fulfilled by the entire fleet, from 2015 to 2100, for each region. See the corresponding section in the "Transport modeling documentation" [4,77].
1_F_RECC_FinalProducts_appliances<br>V1.0<br>ocSRa<br>Items/yr	See the ODYM-RECC v2.4 parameter file 1_F_RECC_FinalProducts_appliances_V1.0.xlsx for details.<br>https://doi.org/10.5281/zenodo.4671643<br>future and past inflow in use phase, appliances.
1_F_RECC_FinalProducts_industry<br>V1.0<br>lSRIc<br>GW/yr	See the ODYM-RECC v2.4 parameter file 1_F_RECC_FinalProducts_industry_V1.0.xlsx for details.<br>https://doi.org/10.5281/zenodo.4671643<br>future and past inflow in use phase, industrial assets (exogenous)
2_S_RECC_FinalProducts_nonresbuildings_g<br>V1.0<br>Nc<br>m²/yr	See the ODYM-RECC v2.4 parameter file 2_S_RECC_FinalProducts_nonresbuildings_g_V1.0.xlsx for details.<br>https://doi.org/10.5281/zenodo.4671643<br>future and past in-use stock scenarios for nonres. Buildings, global resolution

2_S_RECC_FinalProducts_Future_resbuildings<br>v2.3<br>StGr<br>buildings: m2 per person	See the ODYM-RECC v2.4 parameter file 2_S_RECC_FinalProducts_Future_resbuildings_v2.3.xlsx for details. https://doi.org/10.5281/zenodo.4671643<br>Per capita residential floor space tends to increase with GDP, but varies widely across countries at the same level of GDP, shaped by tradition, urban form, as well as land use and building regulations (International Energy Agency, 2016). The SSP scenarios don't detail the floor space in their documentation, and we formulate values which are consistent with the SSP storylines. In the SSP2 scenario and for the USA and Japan (for which relatively rich historical data exists), future per-capita floor space growth rates are an extension of historical rates to 2050 using a data-driven approach, This extends the methods of (Fishman et al., 2016) by incorporating GDP/cap and urbanization rates as drivers. Canada's growth rates are modeled using the USA's 2015-2050 growth rates, and likewise Germany, France, and Italy's growth rates are modeled using Japan's 2015-2050 growth rates, due to similar historical trajectories and current socioeconomic conditions. The LED scenario calls for a global convergence of floor space per capita of 30 m2/cap by 2050, but doesn't provide details by regions (Grubler et al., 2018). In our scenarios, most regions either contract or enlarge their floor area to reach this value by 2050, and do so more rapidly after 2030. The two exceptions are the USA, whose starting point at 2015 is significantly higher than the other modeled regions (nearly 70 m2/cap) and fails to reach the 30 m2/cap by 2050, only contracting to 43 m2/cap. In a similar fashion, India's lower per capita floor area values in 2015 compared to the others allows it to only reach 26 m2/cap by 2050. Details in Fishman et al. [4].
2_S_RECC_FinalProducts_Future_resbuildings_MIUPotential<br>V1.0<br>GoS<br>%	See the ODYM-RECC v2.4 parameter file 2_S_RECC_FinalProducts_Future_resbuildings_MIUPotential_V1.0.x https://doi.org/10.5281/zenodo.4671643<br>This parameter describes the maximum reduction potential for per-capita floor space. This potential is gradually seized over time, starting from the 2015 reference value. Based on various literature sources that describe scenarios for the reduction of per capita floor space (Rao and Baer 2012, DOI 10.3390/su4040656, Grubler et al. 2018, DOI 10.1038/s41560-018-0172-6) or material demand for residential buildings due to more intense use (Milford et al., 2013, DOI: 10.1021/es3031424) a value of 20% was chosen. Moreover, it is ensured that the resulting reduced per capita floor space does not fall below the scenario curve of the low energy demand scenario (LED), which, with a target value of 30 m2/cap, is the bottom line of the assessment. To that end, the actual floor space for each region and year is calculated as maximum(LEDvalue, value obtained from MIU implementation).

2_S_RECC_FinalProducts_Future_NonResBuildings<br>V1.0<br>GrtS<br>buildings: m2 per person	See the ODYM-RECC v2.4 parameter file 2_S_RECC_FinalProducts_Future_NonResBuildings_V1.0.xlsx for https://doi.org/10.5281/zenodo.4671643<br>Per capita non-residential floor space tends to increase with GDP, but varies widely across countries at the same level of GDP, shaped by tradition, urban form, as well as land use and building regulations (International Energy Agency, 2016). The SSP scenarios don't detail the floor space in their documentation, and we formulate values which are consistent with the SSP storylines. For Germany, 2015 stocks of all nonresidential buildings were at 21.3 m2/cap, which, under the LED scenario, will decrease to 20 m2/cap. For SSP1 and SSP2, we assumed future growth rates, leading to 23 and 28 m2/cap, respectively.
2_S_RECC_FinalProducts_Future_nonresbuildings_MIUPotential<br>V1.0<br>GoS<br>%	See the ODYM-RECC v2.4 parameter file 2_S_RECC_FinalProducts_Future_nonresbuildings_MIUPotential_V1.0.xlsx for details. https://doi.org/10.5281/zenodo.4671643<br>This parameter describes the maximum reduction potential for per-capita floor space. This potential is gradually seized over time, starting from the 2015 reference value. Based on various literature sources that describe scenarios for the reduction of per capita floor space (Rao and Baer 2012, DOI 10.3390/su4040656, Grubler et al. 2018, DOI 10.1038/s41560-018-0172-6) or material demand for residential buildings due to more intense use (Milford et al., 2013, DOI: 10.1021/es3031424) a value of 20% was chosen. Moreover, it is ensured that the resulting reduced per capita floor space does not fall below the scenario curve of the low energy demand scenario (LED), which, with a target value of 23 m2/cap, is the bottom line of the assessment. To that end, the actual floor space for each region and year is calculated as maximum(LEDvalue, value obtained from MIU implementation).
3_EI_Products_UsePhase_passvehicles<br>V1.2<br>cpVnrS<br>Vehicles: MJ/km. Buildings: MJ/m2/yr	See the ODYM-RECC v2.4 parameter file 3_EI_Products_UsePhase_passvehicles_V1.2.xlsx for details. https://doi.org/10.5281/zenodo.4671643
3_EI_Products_UsePhase_resbuildings<br>V1.3<br>cBVnrS<br>Vehicles: MJ/km. Buildings: MJ/m2/yr	See the ODYM-RECC v2.4 parameter file 3_EI_Products_UsePhase_resbuildings_V1.3.xlsx for details. https://doi.org/10.5281/zenodo.4671643
3_EI_Products_UsePhase_nonresbuildings<br>V1.0<br>cNVnrS<br>Vehicles: MJ/km. Buildings: MJ/m2/yr	See the ODYM-RECC v2.4 parameter file 3_EI_Products_UsePhase_nonresbuildings_V1.0.xlsx for details. https://doi.org/10.5281/zenodo.4671643
3_IO_Vehicles_UsePhase<br>v2.3<br>VrtS<br>vehicles: km/yr	See the ODYM-RECC v2.4 parameter file 3_IO_Vehicles_UsePhase_v2.3.xlsx for details. https://doi.org/10.5281/zenodo.4671643

6_MIP_VehicleOccupancyRate<br>V1.3<br>GrtS<br>1	See the ODYM-RECC v2.4 parameter file 6_MIP_VehicleOccupancyRate_V1.3.xlsx for details. https://doi.org/10.5281/zenodo.4671643<br>Occupany rates for vehicles in different regions from 2015-2100. The rates are assumed to be uniform for all vehicle archetypes. See the corresponding section in the "Transport modeling documentation" [4,77].
3_IO_Buildings_UsePhase_Historic<br>V1.3<br>cBVrS<br>share of m2 heated and cooled, historic age-cohorts	See the ODYM-RECC v2.4 parameter file 3_IO_Buildings_UsePhase_Historic_V1.3.xlsx for details. https://doi.org/10.5281/zenodo.4671643
3_IO_Buildings_UsePhase_Future_Heating<br>V1.0<br>GrtS<br>share of m2 heated, future age-cohorts	See the ODYM-RECC v2.4 parameter file 3_IO_Buildings_UsePhase_Future_Heating_V1.0.xlsx for details. https://doi.org/10.5281/zenodo.4671643
3_IO_Buildings_UsePhase_Future_Cooling<br>V1.0<br>GrtS<br>share of m2 cooled, future age-cohorts	See the ODYM-RECC v2.4 parameter file 3_IO_Buildings_UsePhase_Future_Cooling_V1.0.xlsx for details. https://doi.org/10.5281/zenodo.4671643
3_IO_NonResBuildings_UsePhase<br>V1.0<br>cNVrS<br>share of m2 heated and cooled, future age-cohorts	See the ODYM-RECC v2.4 parameter file 3_IO_NonResBuildings_UsePhase_V1.0.xlsx for details. https://doi.org/10.5281/zenodo.4671643
4_TC_ResidentialEnergyEfficiency_Default<br>V1.0<br>VRrnt	See the ODYM-RECC v2.4 parameter file 4_TC_ResidentialEnergyEfficiency_Default_V1.0.xlsx for details. https://doi.org/10.5281/zenodo.4671643
4_TC_ResidentialEnergyEfficiency_Scenario_Heating<br>V1.0<br>VRrntS	See the ODYM-RECC v2.4 parameter file 4_TC_ResidentialEnergyEfficiency_Scenario_Heating_V1.0.xlsx for details. https://doi.org/10.5281/zenodo.4671643
4_TC_ResidentialEnergyEfficiency_Scenario_Cooling<br>V1.0<br>VRrntS	See the ODYM-RECC v2.4 parameter file 4_TC_ResidentialEnergyEfficiency_Scenario_Cooling_V1.0.xlsx for details. https://doi.org/10.5281/zenodo.4671643
3_LT_RECC_ProductLifetime_passvehicles<br>V3.1<br>pr<br>yr	See the ODYM-RECC v2.4 parameter file 3_LT_RECC_ProductLifetime_passvehicles_V3.1.xlsx for details. https://doi.org/10.5281/zenodo.4671643

3_LT_RECC_ProductLifetime_resbuildings<br>V4.2<br>Brc<br>yr	See the ODYM-RECC v2.4 parameter file 3_LT_RECC_ProductLifetime_resbuildings_V4.2.xlsx for details. https://doi.org/10.5281/zenodo.4671643
3_LT_RECC_ProductLifetime_NonResbuildings<br>V1.0<br>Nrc<br>yr	See the ODYM-RECC v2.4 parameter file 3_LT_RECC_ProductLifetime_NonResbuildings_V1.0.xlsx for details. https://doi.org/10.5281/zenodo.4671643
3_LT_RECC_ProductLifetime_appliances<br>V1.0<br>a<br>yr	See the ODYM-RECC v2.4 parameter file 3_LT_RECC_ProductLifetime_appliances_V1.0.xlsx for details. https://doi.org/10.5281/zenodo.4671643
3_LT_RECC_ProductLifetime_industry_V1.0<br>I<br>yr	See the ODYM-RECC v2.4 parameter file 3_LT_RECC_ProductLifetime_industry_V1.0.xlsx for details. https://doi.org/10.5281/zenodo.4671643
3_LT_RECC_ProductLifetime_nonresbuildings_g_V1.0<br>Noc<br>yr	See the ODYM-RECC v2.4 parameter file 3_LT_RECC_ProductLifetime_nonresbuildings_g_V1.0.xlsx for details. https://doi.org/10.5281/zenodo.4671643
3_MC_RECC_Buildings<br>V1.2<br>cmBr<br>kg/m2	See the ODYM-RECC v2.4 parameter file 3_MC_RECC_Buildings_V1.2.xlsx for details. https://doi.org/10.5281/zenodo.4671643
3_MC_RECC_Vehicles<br>V1.1<br>cmpr<br>kg/unit	See the ODYM-RECC v2.4 parameter file 3_MC_RECC_Vehicles_V1.1.xlsx for details. https://doi.org/10.5281/zenodo.4671643
3_MC_RECC_NonResBuildings<br>V1.0<br>cmNr<br>kg/m2	See the ODYM-RECC v2.4 parameter file 3_MC_RECC_NonResBuildings_V1.0.xlsx for details. https://doi.org/10.5281/zenodo.4671643
3_MC_RECC_Nonresbuildings_g<br>V1.0<br>mN<br>kg/m2	See the ODYM-RECC v2.4 parameter file 3_MC_RECC_Nonresbuilding_g_V1.0.xlsx for details. https://doi.org/10.5281/zenodo.4671643
3_MC_RECC_industry<br>V1.1<br>Im<br>kg/m2	See the ODYM-RECC v2.4 parameter file 3_MC_RECC_appliances_V1.1.xlsx for details. https://doi.org/10.5281/zenodo.4671643
3_MC_RECC_appliances<br>V1.1<br>oam<br>kg/m2	See the ODYM-RECC v2.4 parameter file 3_MC_RECC_appliances_V1.1.xlsx for details. https://doi.org/10.5281/zenodo.4671643

3_MC_RECC_Buildings_Renovation_Relative<br>V1.0<br>cmBr<br>1	See the ODYM-RECC v2.4 parameter file<br>3_MC_RECC_Buildings_Renovation_Relative_V1.0.xlsx for details.<br>https://doi.org/10.5281/zenodo.4671643
3_MC_RECC_Buildings_Renovation_Absolute<br>V1.0<br>cmBr<br>kg/m2	See the ODYM-RECC v2.4 parameter file<br>3_MC_RECC_Buildings_Renovation_Absolute_V1.0.xlsx for details.<br>https://doi.org/10.5281/zenodo.4671643
3_MC_Elements_Materials_ExistingStock<br>V2.2<br>me<br>1 (kg/kg)	See the ODYM-RECC v2.4 parameter file<br>3_MC_Elements_Materials_ExistingStock_V2.2.xlsx for details.<br>https://doi.org/10.5281/zenodo.4671643
3_MC_Elements_Materials_Primary<br>V2.2<br>me<br>1 (kg/kg)	See the ODYM-RECC v2.4 parameter file<br>3_MC_Elements_Materials_Primary_V2.2.xlsx for details.<br>https://doi.org/10.5281/zenodo.4671643
3_PR_RECC_CO2Price_SSP_32R<br>V2.1<br>RtrS<br>US$2005/ton	See the ODYM-RECC v2.4 parameter file<br>3_PR_RECC_CO2Price_SSP_32R_V2.1.xlsx for details.<br>https://doi.org/10.5281/zenodo.4671643<br>(not used in current version of ODYM-RECC)
3_SHA_RECC_REStrategyScaleUp<br>V3.3<br>RotS<br>1	See the ODYM-RECC v2.4 parameter file<br>3_SHA_RECC_REStrategyScaleUp_V3.3.xlsx for details.<br>https://doi.org/10.5281/zenodo.4671643<br>This parameter quantifies the extent to which a given industry RE strategy will be implemented (%). It applies to all industry RE strategy parameter and is dependent on time t, socioeconomic scenario S, and climate policy scenario R. In the current implementation (ODYM-RECC v2.4), a spline-smoothed increase of the scale-up curve from 0% in 2019 to 100% in 2040 is assumed, followed by a spline interpolation to reduce the changes in the first derivative. This curve is applied to all regions and climate policy scenarios.
3_SHA_BuildingRenovationScaleUp<br>V1.0<br>RotS<br>1	See the ODYM-RECC v2.4 parameter file<br>3_SHA_BuildingRenovationScaleUp<br>_V1.0.xlsx for details.<br>https://doi.org/10.5281/zenodo.4671643<br>This parameter quantifies the extent to which a given renovation potential (MRP, in % of stock), will have been used in model year t (in %). In the current implementation (ODYM-RECC v2.4), a spline-smoothed increase of the scale-up curve from 0% in 2015 to 100%/80%/60% in 2060 is assumed for LED/SSP1/SSP2 for the NoNewClimPol scenario. For RCP2.6, the curves ramp up to 100% for LED, SSP1, and SSP2.
4_PE_GHGIntensityEnergySupply<br>V4.2<br>XnSRrt<br>kg of CO2-eq/MJ	See the ODYM-RECC v2.4 parameter file<br>4_PE_GHGIntensityEnergySupply_V4.2.xlsx for details.<br>https://doi.org/10.5281/zenodo.4671643

4_PE_GHGIntensityEnergySupply_World<br>V4.1<br>XnSRot<br>kg of CO2-eq/MJ	See the ODYM-RECC v2.4 parameter file<br>4_PE_GHGIntensityEnergySupply_World_V4.1.xlsx for details.<br>https://doi.org/10.5281/zenodo.4671643
4_PE_GHGIntensityElectricitySupply_Backstop<br>V1.2<br>XnSRt<br>kg of CO2-eq/MJ	See the ODYM-RECC v2.4 parameter file<br>4_PE_GHGIntensityElectricitySupply_Backstop_V1.2.xlsx for details.<br>https://doi.org/10.5281/zenodo.4671643
4_PE_ProcessExtensions<br>V3.4<br>PXotRS<br>kg/kg	See the ODYM-RECC v2.4 parameter file<br>4_PE_ProcessExtensions_V3.4.xlsx for details.<br>https://doi.org/10.5281/zenodo.4671643<br>This parameter describes the lifecycle kg of GHG emissions associated with production of 1 kg of the main construction and manufacturing materials considered in this study. Values are based on ecoinvent and recalculated for changing global electricity mix and assumptions on production efficiency (Vandepaer et al. 2019). Own assumptions for steel production (H2-based) and Al production were made and documented in the parameter file.
4_EI_ProcessEnergyIntensity<br>V2.2<br>PntoR<br>MJ/kg	See the ODYM-RECC v2.4 parameter file<br>4_EI_ProcessEnergyIntensity_V2.2.xlsx for details.<br>https://doi.org/10.5281/zenodo.4671643
4_EI_ManufacturingEnergyIntensity<br>V2.2<br>Fnco<br>MJ/kg	See the ODYM-RECC v2.4 parameter file<br>4_EI_ManufacturingEnergyIntensity_V2.2.xlsx for details.<br>https://doi.org/10.5281/zenodo.4671643
4_PY_EoL_RecoveryRate<br>v2.4<br>gomwW<br>%	See the ODYM-RECC v2.4 parameter file<br>4_PY_EoL_RecoveryRate_v2.4.xlsx for details.<br>https://doi.org/10.5281/zenodo.4671643
4_PY_Manufacturing<br>V2.3<br>mwgFto<br>1	See the ODYM-RECC v2.4 parameter file<br>4_PY_Manufacturing_V2.3.xlsx for details.<br>https://doi.org/10.5281/zenodo.4671643
4_PY_MaterialProductionRemelting<br>v2.2<br>wmeWto<br>1	See the ODYM-RECC v2.4 parameter file<br>4_PY_MaterialProductionRemelting_v2.2.xlsx for details.<br>https://doi.org/10.5281/zenodo.4671643
4_EI_WasteMgtEnergyIntensity<br>V1.1<br>wnco<br>MJ/kg	See the ODYM-RECC v2.4 parameter file<br>4_EI_WasteMgtEnergyIntensity_V1.1.xlsx for details.<br>https://doi.org/10.5281/zenodo.4671643
4_EI_RemeltingEnergyIntensity<br>V2.1<br>mnco<br>MJ/kg	See the ODYM-RECC v2.4 parameter file<br>4_EI_RemeltingEnergyIntensity_V2.1.xlsx for details.<br>https://doi.org/10.5281/zenodo.4671643

6_PR_EoL_RR_Improvement<br>v2.3<br>gomwW<br>percentage points	See the ODYM-RECC v2.4 parameter file 6_PR_EoL_RR_Improvement_v2.3.xlsx for details. https://doi.org/10.5281/zenodo.4671643
6_PR_LifeTimeExtension_passvehicles<br>V2.1<br>poS<br>1	See the ODYM-RECC v2.4 parameter file 6_PR_LifeTimeExtension_passvehicles_V2.1.xlsx for details. https://doi.org/10.5281/zenodo.4671643
6_PR_LifeTimeExtension_resbuildings<br>v2.3<br>BrS<br>1	See the ODYM-RECC v2.4 parameter file 6_PR_LifeTimeExtension_resbuildings_v2.3.xlsx for details. https://doi.org/10.5281/zenodo.4671643
6_PR_LifeTimeExtension_nonresbuildings<br>V1.1<br>Nr<br>1	See the ODYM-RECC v2.4 parameter file 6_PR_LifeTimeExtension_nonresbuildings_V1.1.xlsx for details. https://doi.org/10.5281/zenodo.4671643
6_PR_LifeTimeExtension_nonresbuildings_g<br>V1.0<br>No<br>1	See the ODYM-RECC v2.4 parameter file 6_PR_LifeTimeExtension_nonresbuildings_g_V1.0.xlsx for details. https://doi.org/10.5281/zenodo.4671643
6_PR_LifeTimeExtension_industry<br>V1.0<br>IlS<br>1	See the ODYM-RECC v2.4 parameter file 6_PR_LifeTimeExtension_industry_V1.0.xlsx for details. https://doi.org/10.5281/zenodo.4671643
6_PR_LifeTimeExtension_appliances<br>V1.0<br>aoS<br>1	See the ODYM-RECC v2.4 parameter file 6_PR_LifeTimeExtension_appliances_V1.0.xlsx for details. https://doi.org/10.5281/zenodo.4671643
6_PR_FabricationYieldImprovement<br>V2.1<br>mgoS<br>1	See the ODYM-RECC v2.4 parameter file 6_PR_FabricationYieldImprovement_V2.1.xlsx for details. https://doi.org/10.5281/zenodo.4671643
6_PR_FabricationScrapDiversion<br>V1.2<br>mwoS<br>1	See the ODYM-RECC v2.4 parameter file 6_PR_FabricationScrapDiversion_V1.2.xlsx for details. https://doi.org/10.5281/zenodo.4671643
6_PR_ReUse_Bld<br>V3.3<br>mBo<br>1	See the ODYM-RECC v2.4 parameter file 6_PR_ReUse_Bld_V3.3.xlsx for details. https://doi.org/10.5281/zenodo.4671643

6_PR_ReUse_Veh<br>V1.2<br>mprtS<br>1	See the ODYM-RECC v2.4 parameter file<br>6_PR_ReUse_Veh_V1.2.xlsx for details.<br>https://doi.org/10.5281/zenodo.4671643<br>Reuse rates for different materials (e.g., % of cast Al from reuse of vehicle components) in 6 vehicle archetypes, from 2015 to 2100, for each region. No further adjustment is made for weight options or size segments. See the corresponding section in the "Transport modeling documentation".
6_PR_ReUse_nonresBld<br>V1.2<br>mNo<br>1	See the ODYM-RECC v2.4 parameter file<br>6_PR_ReUse_nonresBld_V1.2.xlsx for details.<br>https://doi.org/10.5281/zenodo.4671643
6_PR_DirectEmissions<br>V1.2<br>Xn<br>kg of CO2-eq/MJ	See the ODYM-RECC v2.4 parameter file<br>6_PR_DirectEmissions_V1.2.xlsx for details.<br>https://doi.org/10.5281/zenodo.4671643
6_PR_CarSharingShare<br>V1.2<br>GotS<br>1	See the ODYM-RECC v2.4 parameter file<br>6_PR_CarSharingShare_V1.2.xlsx for details.<br>https://doi.org/10.5281/zenodo.4671643
6_PR_RideSharingShare<br>V2.0<br>GrtS<br>1	See the ODYM-RECC v2.4 parameter file<br>6_PR_RideSharingShare_V2.0.xlsx for details.<br>https://doi.org/10.5281/zenodo.4671643<br>Percentage of transport service demand fulfilled by ride sharing from 2015 to 2100, assumed to be identical for all regions and all archetypes. See the corresponding section in the "Transport modeling documentation".
3_SHA_TypeSplit_Vehicles<br>V3.0<br>GrRpt<br>%	See the ODYM-RECC v2.4 parameter file<br>3_SHA_TypeSplit_Vehicles_V3.0.xlsx for details.<br>https://doi.org/10.5281/zenodo.4671643<br>As part of the project-wide effort to link the ODYM-RECC parameters to existing scenarios, the IEA Energy Technology Perspectives results were used as they were available to us.<br>Data source: 2017 ETP. Fig. 5.3 in 'International Energy Agency (2017), Energy Technology Perspectives 2017, OECD/IEA, Paris'<br>Applied the following proxy settings: Scenario mapping:<br>Baseline scenario R aspect: Reference Technology Scenario<br>RCP2.6: Beyond 2°C Scenario (with significantly more EVs)
3_SHA_TypeSplit_Buildings<br>V1.3<br>BrtS<br>%	See the ODYM-RECC v2.4 parameter file<br>3_SHA_TypeSplit_Buildings_V1.3.xlsx for details.<br>https://doi.org/10.5281/zenodo.4671643<br>This parameter describes the split of residential buildings along two dimensions, category (single family housing, multifamily housing, informal) and energy efficiency standard (non-standard, standard, efficient, zero-energy-building). Projections are made in line with the socioconomic storylines, while the shares of multifamily housing is linked to the narrative on urbanisation and use intensity. Higher shares of efficienct buildings are assumed in the SSP1 and LED scenarios.

3_SHA_TypeSplit_NonResBuildings<br>V1.0<br>NrtS<br>%	See the ODYM-RECC v2.4 parameter file 3_SHA_TypeSplit_NonResBuildings_V1.0.xlsx for details.<br>https://doi.org/10.5281/zenodo.4671643<br>This parameter describes the split of nonresidential buildings along two dimensions, category (office, commerce, healt, education, hotels&restaurants, other) and energy efficiency standard (non-standard, standard, efficient, zero-energy-building). Projections are made in line with the socioconomic storylines, while the shares of the categories remain constant over time. Higher shares of efficienct buildings are assumed in the SSP1 and LED scenarios.
3_SHA_EnergyCarrierSplit_Vehicles<br>V1.1<br>cpoVnS<br>%	See the ODYM-RECC v2.4 parameter file 3_SHA_EnergyCarrierSplit_Vehicles_V1.1.xlsx for details.<br>https://doi.org/10.5281/zenodo.4671643
3_SHA_EnergyCarrierSplit_Buildings<br>v2.3<br>VRrnt<br>%	See the ODYM-RECC v2.4 parameter file 3_SHA_EnergyCarrierSplit_Buildings_v2.3.xlsx for details.<br>https://doi.org/10.5281/zenodo.4671643<br>As part of the project-wide effort to link the ODYM-RECC parameters to existing scenarios, the IEA Energy Technology Perspectives results, featuring a reference technology and a 2°C-compatible scenario, were used. These scenario results are from 2017 and were provided to us at the country level, from which we aggregated them to the regional resolution of the RECC assessment. The raw data report the total residential energy consumption by energy carrier, from which we calculated the share of the individual energy carriers in the total mix.
3_SHA_EnergyCarrierSplit_NonResBuildings<br>V1.0<br>VRrnt<br>%	See the ODYM-RECC v2.4 parameter file 3_SHA_EnergyCarrierSplit_NonResBuildings_V1.0.xlsx for details.<br>https://doi.org/10.5281/zenodo.4671643<br>As part of the project-wide effort to link the ODYM-RECC parameters to existing scenarios, the IEA Energy Technology Perspectives results, featuring a reference technology and a 2°C-compatible scenario, were used. These scenario results are from 2017 and were provided to us at the country level, from which we aggregated them to the regional resolution of the RECC assessment. The raw data report the total nonresidential energy consumption by energy carrier, from which we calculated the share of the individual energy carriers in the total mix.
3_MC_VehicleArchetypes<br>V2.0<br>Am<br>kg/unit, kg/m2	See the ODYM-RECC v2.4 parameter file 3_MC_VehicleArchetypes_V2.0.xlsx for details.<br>https://doi.org/10.5281/zenodo.4671643<br>material composition (in kg) of 48 vehicle archetypes (6 powertrains x 2 weight options x 4 size segments), assumed to be identical for all regions and all time. The values are derived from the material composition data in GREET2 vehicle cycle model. See the corresponding section in the "Transport modeling documentation".

3_EI_VehicleArchetypes<br>V4.0<br>An<br>MJ/km, MJ/m2/yr	See the ODYM-RECC v2.4 parameter file<br>3_EI_VehicleArchetypes_V4.0.xlsx for details.<br>https://doi.org/10.5281/zenodo.4671643<br>Fuel economy (in MJ/km) of 48 vehicle archetypes (6 powertrains x 2 weight options x 4 size segments), assumed to be identical for all regions and all time. The values are derived from "Future Automotive Systems Technology Simulator" (FASTSim). See the corresponding section in the "Transport modeling documentation".
3_MC_BuildingArchetypes<br>V1.2<br>Arm<br>kg/unit, kg/m2	See the ODYM-RECC v2.4 parameter file<br>3_MC_BuildingArchetypes_V1.2.xlsx for details.<br>https://doi.org/10.5281/zenodo.4671643<br>This parameter describes the material composition of building archetypes, in terms of mass per unit floor space (kg/m2). Values are calculated based on Taylor et al. 2015 and Heeren and Fishman 2019.
3_EI_BuildingArchetypes<br>V1.2<br>ArVn<br>MJ/km, MJ/m2/yr	See the ODYM-RECC v2.4 parameter file<br>3_EI_BuildingArchetypes_V1.2.xlsx for details.<br>https://doi.org/10.5281/zenodo.4671643<br>This parameter describes the energy intensity per unit of floor area. It covers energy services of three major energy end-uses in residential buildings: space heating, space cooling, and domestic hot water. Values are defined for each of the building 'types' defined in 3_SHA_TypeSplit_Buildings, and are based on a simluation of energy consumption using energyplus and based on archetypes from Taylor et al. 2015.
3_MC_NonResBuildingArchetype<br>s<br>V1.0<br>Arm<br>kg/unit, kg/m2	See the ODYM-RECC v2.4 parameter file<br>3_MC_NonResBuildingArchetypes_V1.0.xlsx for details.<br>https://doi.org/10.5281/zenodo.4671643<br>This parameter describes the material composition of building archetypes, in terms of mass per unit floor space (kg/m2). Values are calculated based on Taylor et al. 2015 and Heeren and Fishman 2019. Multi-familiy residential building material composition data are used as proxy for the different nonresidential building types.
3_EI_NonResBuildingArchetypes<br>V1.0<br>ArVn<br>MJ/km, MJ/m2/yr	See the ODYM-RECC v2.4 parameter file<br>3_EI_NonResBuildingArchetypes_V1.0.xlsx for details.<br>https://doi.org/10.5281/zenodo.4671643<br>This parameter describes the energy intensity per unit of floor area. It covers energy services of three major energy end-uses in non-residential buildings: space heating, space cooling, and domestic hot water. Values are defined for each of the building 'types' defined in 3_SHA_TypeSplit_NonResBuildings, and are based on a simluation of energy consumption using energyplus and based on archetypes from Taylor et al. 2015. Here: Multi-family houses of different energy standards are used as proxy for the heating energy demand figures, and for cooling and hot water demand, the latest available figures from ca. 2014 were applied to all future years, simulating a continued demand for cooling loads and hot water.
3_SHA_DownSizing_Vehicles<br>V2.3<br>srtS<br>%	See the ODYM-RECC v2.4 parameter file<br>3_SHA_DownSizing_Vehicles_V2.3.xlsx for details.<br>https://doi.org/10.5281/zenodo.4671643<br>market share of each size segment of vehicles in the production (inflows) of new vehicles each year, from 2015 to 2100, for each region. See the corresponding section in the "Transport modeling documentation".

8_FLAG_VehicleDownsizingDirection<br>V1.0<br>rS<br>Bool	See the ODYM-RECC v2.4 parameter file 8_FLAG_VehicleDownsizingDirection_V1.0.xlsx for details. https://doi.org/10.5281/zenodo.4671643 Flag is set so that either base case or scenario case lead lower GHG emissions (shift towards smaller segments)
3_SHA_LightWeighting_Vehicles<br>V1.3<br>prtS<br>%	See the ODYM-RECC v2.4 parameter file 3_SHA_LightWeighting_Vehicles_V1.3.xlsx for details.<br>https://doi.org/10.5281/zenodo.4671643<br>Market share of lightweighted vehicles of each powertrain in the production (inflows) of new vehicles each year, from 2015 to 2100, for each region. See the corresponding section in the "Transport modeling documentation".
3_SHA_DownSizing_Buildings<br>V1.3<br>urtS<br>%	See the ODYM-RECC v2.4 parameter file 3_SHA_DownSizing_Buildings_V1.3.xlsx for details.<br>https://doi.org/10.5281/zenodo.4671643<br>This parameter describes the share of new constructions which are considered in the lightweighting Material Efficiency Strategy.
3_SHA_LightWeighting_Buildings<br>v2.2<br>GrtS<br>%	See the ODYM-RECC v2.4 parameter file 3_SHA_LightWeighting_Buildings_v2.2.xlsx for details.<br>https://doi.org/10.5281/zenodo.4671643<br>This parameter describes the share of new residential buildings which is built a predominantly timber-frame structure, as opposed to concrete. It is based on the construction styles most often implemented in each country currently (e.g. US, Canada, and Japan start with already high shares of 'lightweight' buildings). Generally, countries which do not currently have a large share of lightweighted buildings are projected build more timber-frame structures so that the share of lightweighted buildings in new construction reaches 85% (LED), 50% (SSP1), and 10% (SSP2) in 2050, while countries with already high shares generally remain stable or slightly increase the share of lightweighted buildings to 95% in 2050
3_SHA_DownSizing_NonResBuildings<br>V1.0<br>urtS<br>%	See the ODYM-RECC v2.4 parameter file 3_SHA_DownSizing_NonResBuildings_V1.0.xlsx for details.<br>https://doi.org/10.5281/zenodo.4671643<br>This parameter describes the share of new constructions which are considered in the lightweighting Material Efficiency Strategy.
3_SHA_LightWeighting_NonResBuildings<br>V1.0<br>GrtS<br>%	See the ODYM-RECC v2.4 parameter file 3_SHA_LightWeighting_NonResBuildings_V1.0.xlsx for details.<br>https://doi.org/10.5281/zenodo.4671643<br>This parameter describes the share of new nonresidential buildings which is built a predominantly timber-frame structure, as opposed to concrete. It is based on the construction styles most often implemented in each country currently (e.g. US, Canada, and Japan start with already high shares of 'lightweight' buildings). Generally, countries which do not currently have a large share of lightweighted buildings are projected build more timber-frame structures so that the share of lightweighted buildings in new construction reaches 85% (LED), 50% (SSP1), and 10% (SSP2) in 2050, while countries with already high shares generally remain stable or slightly increase the share of lightweighted buildings to 95% in 2050

6_PR_Calibration<br>v2.4<br>Cr<br>ratios	See the ODYM-RECC v2.4 parameter file 6_PR_Calibration_v2.4.xlsx for details. https://doi.org/10.5281/zenodo.4671643
6_MIP_CarSharing_Stock<br>V1.0<br>Sr<br>1	See the ODYM-RECC v2.4 parameter file 6_MIP_CarSharing_Stock_V1.0.xlsx for details.<br>https://doi.org/10.5281/zenodo.4671643<br>Ratio of per capita passenger vehicle stock with vs. without carsharing to describe the reduction of vehicle stock due to car-sharing in different regions, assumed to be identical for all time. The rates are assumed to be uniform for all vehicle archetypes. See the corresponding section in the "Transport modeling documentation".
6_MIP_RideSharing_Occupancy<br>V1.1 (for RECC Germany: V1.0)<br>Sr<br>1	See the ODYM-RECC v2.4 parameter file 6_MIP_RideSharing_Occupancy_V1.1.xlsx for details.<br>https://doi.org/10.5281/zenodo.4671643<br>Occupancy rates for ride-sharing vehicles in different regions, assumed to be identical for all time. The rates are assumed to be uniform for all vehicle archetypes. See the corresponding section in the "Transport modeling documentation".<br>Global study: this parameter = 1, as the occupancy rate increases by 1 under ride-sharing (absol. increase)<br>Case study Germany: Relative factor, previous OR increases by factor 1.4.
6_MIP_GWP_Bio<br>V1.0<br>c<br>1	See the ODYM-RECC v2.4 parameter file 6_MIP_GWP_Bio_V1.0.xlsx for details. https://doi.org/10.5281/zenodo.4671643
3_SHA_MaxRenovationPotential_ResBuildings<br>V1.1<br>rcB<br>1	See the ODYM-RECC v2.4 parameter file 3_SHA_MaxRenovationPotential_ResBuildings_V1.1.xlsx for details.<br>https://doi.org/10.5281/zenodo.4671643<br>Potential estimates are in line with Bürger et al. 2018, DOI 10.1007/s12053-018-9660-6
3_SHA_MaxRenovationPotential_NonResBuildings<br>V1.0<br>rcN<br>1	See the ODYM-RECC v2.4 parameter file 3_SHA_MaxRenovationPotential_NonResBuildings_V1.0.xlsx for details. https://doi.org/10.5281/zenodo.4671643<br>Potential estimates are in line with Bürger et al. 2018, DOI 10.1007/s12053-018-9660-6
3_SHA_EnergySavingsPot_Renovation_ResBuildings<br>V1.1<br>rSB<br>1	See the ODYM-RECC v2.4 parameter file 3_SHA_EnergySavingsPot_Renovation_ResBuildings_V1.1.xlsx for details. https://doi.org/10.5281/zenodo.4671643<br>Potential estimates are in line with Bürger et al. 2018, DOI 10.1007/s12053-018-9660-6
3_SHA_EnergySavingsPot_Renovation_NonResBuildings<br>V1.0<br>rSN<br>1	See the ODYM-RECC v2.4 parameter file 3_SHA_EnergySavingsPot_Renovation_NonResBuildings_V1.0.xlsx for details. https://doi.org/10.5281/zenodo.4671643<br>Potential estimates are in line with Bürger et al. 2018, DOI 10.1007/s12053-018-9660-6

6_MIP_CharacterisationFactors<br>V1.0<br>xX<br>misc. units	See the ODYM-RECC v2.4 parameter file<br>6_MIP_CharacterisationFactors_V1.0.xlsx for details.<br>https://doi.org/10.5281/zenodo.4671643
4_PE_ElectricityFromWoodComb<br>ustion<br>V1.0<br>wWn<br>GJ/ton	See the ODYM-RECC v2.4 parameter file<br>4_PE_ElectricityFromWoodCombustion_V1.0.xlsx for details.<br>https://doi.org/10.5281/zenodo.4671643
3_LT_ForestRotationPeriod_Fuel<br>Wood<br>V1.0<br>n<br>yr	See the ODYM-RECC v2.4 parameter file<br>3_LT_ForestRotationPeriod_FuelWood_V1.0.xlsx for details.<br>https://doi.org/10.5281/zenodo.4671643
3_LT_ForestRotationPeriod_Tim<br>ber<br>V1.0<br>m<br>yr	See the ODYM-RECC v2.4 parameter file<br>3_LT_ForestRotationPeriod_Timber_V1.0.xlsx for details.<br>https://doi.org/10.5281/zenodo.4671643
3_MC_CO2FromWoodCombusti<br>on<br>V1.0<br>xm<br>1	See the ODYM-RECC v2.4 parameter file<br>3_MC_CO2FromWoodCombustion_V1.0.xlsx for details.<br>https://doi.org/10.5281/zenodo.4671643
3_EI_HeatingValueWoodPerCarb<br>on<br>V1.0<br>en<br>MJ/kg	See the ODYM-RECC v2.4 parameter file<br>3_EI_HeatingValueWoodPerCarbon_V1.0.xlsx for details.<br>https://doi.org/10.5281/zenodo.4671643
3_MC_CementContentConcrete<br>V1.0<br>mm<br>1	See the ODYM-RECC v2.4 parameter file<br>3_MC_CementContentConcrete_V1.0.xlsx for details.<br>https://doi.org/10.5281/zenodo.4671643
3_SHA_CementContentReductio<br>n<br>V1.0<br>m<br>misc. units	See the ODYM-RECC v2.4 parameter file<br>3_SHA_CementContentReduction_V1.0.xlsx for details.<br>https://doi.org/10.5281/zenodo.4671643

6. The ODYM-RECC model

This section contains and describes the ODYM-RECC model setup and model equations that transform the above-listed parameters into the system variables (material and product stocks and flows). The model is comprehensive in its scope but still, many important system linkages are not implemented in ODYM-RECC 2.4, including a detailed depiction of the waste management cascade and an assessment of the costs of the different ME strategies.

6.1. Theoretical foundation of ODYM-RECC

Our starting point is that ODYM-RECC described the material aspects and system linkages of socioeconomic metabolism and in-use stocks as the biophysical layer of human society as complex self-reproducing system [85].

- The functioning of social systems requires humans to organize energy and material flows for their own bodies' reproduction and the reproduction of the built up in-use stocks, i.e., socioeconomic metabolism.
- The particular way in which socioeconomic metabolism is operated determines the system's environmental impacts.
- Basic laws of natural science (thermodynamics, constancy of matter) also apply to social and economic systems and are to be respected [86], also when modelling substitution between materials and other production factors.

In a complex self-reproducing system including humans it is not possible to capture all linkages, not even all relevant linkages. Many important linkages (changes in attitude, political situation, new technologies) have to be omitted from the model or represented in a stylized manner only. Supplementary Table 22 list the *system linkages* that are captured and those that are not captured.

Supplementary Table 22: System linkages captured and not captured by ODYM-RECC.

System linkage	Degree of capture
Wellbeing and service demand	Not covered
Service demand and stocks	Partly covered. Stocks serve as proxy for services in the building sector, and their the intensity of use is modelled. Passenger vehicle transport is modelled as service flow of annual passenger-km by vehicles
Stock-flow	Fully covered by dynamic stock model (population balance model), stock-driven approach
Flow-material	Fully covered by material intensity and material substitution parameters
Flow-waste/scrap	Fully covered by detailed description of waste management industries
Scrap-material	Fully covered
Material-alloy-element	Partly covered (chemical elements are considered but no evaluation or constraints regarding this linkage)
Product life cycles	Partly covered by material cycle foreground model and partly by extension for energy and services.

Our main motivation for this approach, instead of using an economic model, is twofold: First, the higher resolution and biophysical consistency that a biophysical model offers, and second: the nature of the strategy implementation, i.e., whether it is implemented via economic incentives, regulations, or lifestyle changes, is yet unclear. Our approach allows to explore the sociometabolic consequences of a certain implementation pattern without prescribing the nature of its implementation.

6.2. Reference to methods and software used.

Once we have either a product consumption or a product stock demand we can use the established and available dynamic MFA routines to

+ determine product inflow and outflow, and the material composition of these flows (using product material composition data and product lifetimes) [23,87], Python code available.

+ determine the optimal response of the waste management industries to the end-of life product flows (using EoL recovery efficiencies and waste management process descriptions), Python code available and running for simple case, unresolved nonlinear constraint for the case where alloying elements are considered. [88–90] *[not implemented]*

+ determine the resulting level of primary production, (using the process inventories of the primary metal producers, the available scrap supply, and metal demand from manufacturing).

+ determine the resulting mining output (using available mining inventories by the Monash colleagues), mining exploitation routine is still under development and *not implemented*. [91,92]

+ determine the impact of material efficiency on the metal cycles (using scenarios for resource and material efficiency). [25,26,36]

+ estimate energy demand from the metal cycles and mining operation for comparison with other scenario results (using process inventory data).

+ quantify the resulting environmental impact and GHG emissions savings from the different resource efficiency strategies (using scenarios for resource and material efficiency and applying them across the modelled system)

+ IO model scenario building and prospective hybrid LCA [83], *not implemented*.

6.3. Basic ODYM-RECC modules and model equations

The model aspects and the resolution (classification items) of ODYM-RECC v2.4 are listed above. Here we define the system variables, model equations and modules, and the model parameters for the basic version v2.4, without the consideration of costs, optimisation, and rebound effects.

The ODYM-RECC model equations are based on the system definition in Supplementary Figure 41.

The modules that have been implemented in v2.4 are listed below.

The generic system definition in Supplementary Figure 41 provides an overview of the processes, flows, stocks, and resource efficiency strategies covered for ODYM-RECC v2.4.

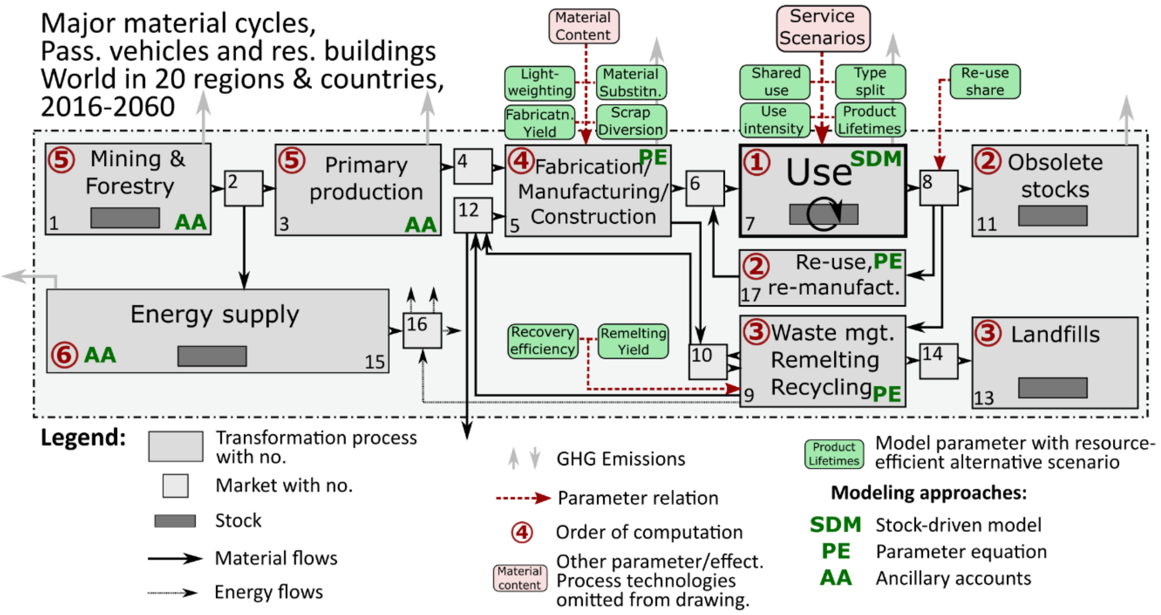

Supplementary Figure 41: System definition of ODYM-RECC assessment with model parameters, resource efficiency strategies, and the modelling approaches taken for the computation of the material cycle response to resource efficiency. This figure is identical to the system definition shown further up above and is repeated here for convenience.

6.3.1. ODYM-RECC modules, overview

The design principle of ODYM-RECC is modular to facilitate update of parts, versioning, testing, and code management. The different modules are built on the underlying ODYM software framework [6].

Supplementary Table 23: The ODYM-RECC modules

Module and function	Layers	coverage	Comment/feature
**Use phase UP:** translate in-use stock targets into commodity inflows and outflows (stock-driven model)	Product layer	All regions and products defined	Split product groups (pass. Vehicles) into individual product types
**Waste management and recycling WR:** Determine amount of re-used products and recycled materials	Products, materials, chem. elements	All regional waste streams aggregated to one global flow, treatment by global industry modelled	Fabrication scrap is buffered for one year, thus the elemental composition is known for all flows.
**Manufacturing MF:** Determine use of secondary material, primary production necessary, and fabrication scrap	Products, materials, chem. elements	One global industry for each product group	
**Primary production PP:** calculate energy demand, ore/concentrage/resource demand, and emissions of primary material production	Materials, chem. elements	One global industry for each material	
**Mining and refining: MR:** calculate energy demand, resource demand, and emissions of mining and refining operations	Minerals, chem. elements	[planned: mine-specific where data are available]	*Currently not part of ODYM-RECC*
**Material and element breakdown ME:** Determine element composition of materials	Materials, chem. elements	All materials covered	Determines the average element composition of the materials used in

			manufacturing, the final consumption of materials in products, and the in-use stock.
**Energy consumption and environmental extensions EX:** Calculate the energy consumption by energy carrier for all processes and the relevant environmental extensions, such as GHG emissions.	Energy carriers, env. pressure and impact categories	All processes in the system definition, all energy carriers selected, region-specific emissions factors for energy supply	

Calculation order (cf. also Supplementary Figure 41): ODYM-RECC first calculates the use phase model UP for all regions, products and years. The modules WR, MF, PP, and ME are solved in a year-by-year loop, because the element composition of materials needs to be determined for all previous years before the waste management module can be solved (solution depends on element composition of materials, e.g., copper content of steel). The MR module is currently not part of ODYM-RECC, the emissions factors for primary production used cover the supply chain including mining. The EX module is called last.

6.3.2. System variables

The ODYM-RECC system variables are listed in Supplementary Table 24. The entire model is run for a specific socioeconomic and climate policy scenario (SSP/RCP), and the two related indices, S and R, apply to all system variables and there therefore omitted here.

Supplementary Table 24: The ODYM-RECC system variables, as defined in the system definition Supplementary Figure 41. The variable aspects are case sensitive: S denotes the socioeconomic scenario, s the car segments, R the climate policy scenario, r the 32 SSP regions, etc. For convenience reasons, the material flows are listed as they are defined in the ODYM-RECC model Python code.

Name	Symbol(s)	Explanation
**Function flow from use phase**	$FUNCT_{exog\_fut}(V,t,c,r,g)$	Shelter provided in area-degree-person-hours, transport provided in passenger-km. The subscript "exog" denotes that this variable is exogenously specified.
**Material stocks**	Cf. definition of ODYM-RECC stock dictionary in list below this table	In-use stock of buildings, infrastructure, and products, losses at different stages of the system.
**Material flows**	Cf. definition of ODYM-RECC flow dictionary in list below this table	All material flows
**Energy flows**	$E_{x\_y}(\mathrm{n,t,...})$	Energy flows to operate the different processes in the system
**Emissions flows**	$GHG_{x\_y}(\mathrm{t,...})$	Emissions flows from the different processes in the system, for ODYM-RECC v2.4: GHG only

The ODYM-RECC flow list:

The flows and stocks with sub-indices _Nl and _No represent the flows between processes that are defined at a different regional resolution than the default: 11 regions for Nl and one aggregate world region for No.

```
RECC_System.FlowDict['F_0_1']    = msc.Flow(Name='CO2 uptake', P_Start=0, P_End=1, Indices='t,e', Values=None, Uncert=None, Color=None, ID=None, UUID=None)

RECC_System.FlowDict['F_1_2']    = msc.Flow(Name='harvested wood', P_Start=1, P_End=2, Indices='t,e', Values=None, Uncert=None, Color=None, ID=None, UUID=None)

RECC_System.FlowDict['F_2_3']    = msc.Flow(Name='timber consumed by sawmills', P_Start=2, P_End=3, Indices='t,m,e', Values=None, Uncert=None, Color=None, ID=None, UUID=None)

RECC_System.FlowDict['F_2_7']    = msc.Flow(Name='wood fuel use', P_Start=2, P_End=7, Indices='t,e', Values=None, Uncert=None, Color=None, ID=None, UUID=None)
# This flow is directly routed to the use phase.

RECC_System.FlowDict['F_7_0']    = msc.Flow(Name='wood fuel use direct emissions', P_Start=7, P_End=0, Indices='t,e', Values=None, Uncert=None, Color=None, ID=None, UUID=None)

RECC_System.FlowDict['F_0_3']    = msc.Flow(Name='ore input', P_Start=0, P_End=3, Indices='t,m,e', Values=None, Uncert=None, Color=None, ID=None, UUID=None)

RECC_System.FlowDict['F_3_4']    = msc.Flow(Name='primary material production' , P_Start = 3, P_End = 4, Indices = 't,m,e', Values=None, Uncert=None, Color = None, ID = None, UUID = None)

RECC_System.FlowDict['F_4_5']    = msc.Flow(Name='primary material consumption' , P_Start = 4, P_End = 5, Indices = 't,m,e', Values=None, Uncert=None, Color = None, ID = None, UUID = None)

RECC_System.FlowDict['F_5_6']    = msc.Flow(Name='manufacturing output' , P_Start = 5, P_End = 6, Indices = 't,o,g,m,e', Values=None, Uncert=None, Color = None, ID = None, UUID = None)

RECC_System.FlowDict['F_6_7']    = msc.Flow(Name='final consumption', P_Start=6, P_End=7, Indices='t,r,g,m,e', Values=None, Uncert=None, Color=None, ID=None, UUID=None)

RECC_System.FlowDict['F_6_7_Nl']  = msc.Flow(Name='final consumption Nl', P_Start=6, P_End=7, Indices='t,l,L,m,e', Values=None, Uncert=None, Color=None, ID=None, UUID=None)

RECC_System.FlowDict['F_6_7_No']  = msc.Flow(Name='final consumption No', P_Start=6, P_End=7, Indices='t,o,O,m,e', Values=None, Uncert=None, Color=None, ID=None, UUID=None)

RECC_System.FlowDict['F_7_8']     = msc.Flow(Name='EoL products' , P_Start = 7, P_End = 8, Indices = 't,c,r,g,m,e', Values=None, Uncert=None,  Color = None, ID = None, UUID = None)

RECC_System.FlowDict['F_7_8_Nl']  = msc.Flow(Name='EoL products Nl' , P_Start = 7, P_End = 8, Indices = 't,c,l,L,m,e', Values=None, Uncert=None, Color = None, ID = None, UUID = None)

RECC_System.FlowDict['F_7_8_No']  = msc.Flow(Name='EoL products No' , P_Start = 7, P_End = 8, Indices = 't,c,o,O,m,e', Values=None, Uncert=None, Color = None, ID = None, UUID = None)

RECC_System.FlowDict['F_8_0']     = msc.Flow(Name='obsolete stock formation' , P_Start = 8, P_End = 0, Indices = 't,c,r,g,m,e', Values=None, Uncert=None, Color = None, ID = None, UUID = None)
```

```
RECC_System.FlowDict['F_8_0_Nl']  = msc.Flow(Name='obsolete stock formation Nl' , P_Start = 8, P_End = 0, Indices = 't,c,l,L,m,e', Values=None, Uncert=None, Color = None, ID = None, UUID = None)

RECC_System.FlowDict['F_8_0_No']  = msc.Flow(Name='obsolete stock formation No' , P_Start = 8, P_End = 0, Indices = 't,c,o,O,m,e', Values=None, Uncert=None, Color = None, ID = None, UUID = None)

RECC_System.FlowDict['F_8_9']     = msc.Flow(Name='waste mgt. input' , P_Start = 8, P_End = 9, Indices = 't,r,g,m,e', Values=None, Uncert=None, Color = None, ID = None, UUID = None)

RECC_System.FlowDict['F_8_9_Nl']  = msc.Flow(Name='waste mgt. input Nl' , P_Start = 8, P_End = 9, Indices = 't,l,L,m,e', Values=None, Uncert=None, Color = None, ID = None, UUID = None)

RECC_System.FlowDict['F_8_9_No']  = msc.Flow(Name='waste mgt. input No' , P_Start = 8, P_End = 9, Indices = 't,o,O,m,e', Values=None, Uncert=None, Color = None, ID = None, UUID = None)

RECC_System.FlowDict['F_8_17']    = msc.Flow(Name='product re-use in' , P_Start = 8, P_End = 17, Indices = 't,c,r,g,m,e', Values=None, Uncert=None, 
Color = None, ID = None, UUID = None)

RECC_System.FlowDict['F_8_17_Nl'] = msc.Flow(Name='product re-use in Nl' , P_Start = 8, P_End = 17, Indices = 't,c,l,L,m,e', Values=None, Uncert=None, Color = None, ID = None, UUID = None)

RECC_System.FlowDict['F_8_17_No'] = msc.Flow(Name='product re-use in No' , P_Start = 8, P_End = 17, Indices = 't,c,o,O,m,e', Values=None, Uncert=None, Color = None, ID = None, UUID = None)

RECC_System.FlowDict['F_17_6']    = msc.Flow(Name='product re-use out' , P_Start = 17, P_End = 6, Indices = 't,c,r,g,m,e', Values=None, Uncert=None, 
Color = None, ID = None, UUID = None)

RECC_System.FlowDict['F_17_6_Nl'] = msc.Flow(Name='product re-use out' , P_Start = 17, P_End = 6, Indices = 't,c,l,L,m,e', Values=None, Uncert=None, Color = None, ID = None, UUID = None)

RECC_System.FlowDict['F_17_6_No'] = msc.Flow(Name='product re-use out' , P_Start = 17, P_End = 6, Indices = 't,c,o,O,m,e', Values=None, Uncert=None, Color = None, ID = None, UUID = None)

RECC_System.FlowDict['F_9_10']    = msc.Flow(Name='old scrap' , P_Start = 9, P_End = 10, Indices = 't,r,w,e', Values=None, Uncert=None, 
Color = None, ID = None, UUID = None)

RECC_System.FlowDict['F_9_10_Nl'] = msc.Flow(Name='old scrap Nl' , P_Start = 9, P_End = 10, Indices = 't,l,w,e', Values=None, Uncert=None, Color = None, ID = None, UUID = None)

RECC_System.FlowDict['F_9_10_No'] = msc.Flow(Name='old scrap No' , P_Start = 9, P_End = 10, Indices = 't,o,w,e', Values=None, Uncert=None, Color = None, ID = None, UUID = None)

RECC_System.FlowDict['F_5_10']  = msc.Flow(Name='new scrap' , P_Start = 5, P_End = 10, Indices = 't,o,w,e', Values=None, Uncert=None, 
Color = None, ID = None, UUID = None)

RECC_System.FlowDict['F_10_9']  = msc.Flow(Name='scrap use' , P_Start = 10, P_End = 9, Indices = 't,o,w,e', Values=None, Uncert=None, Color = None, ID = None, UUID = None)

RECC_System.FlowDict['F_9_12']  = msc.Flow(Name='secondary material production' , P_Start = 9, P_End = 12, Indices = 't,o,m,e', Values=None, Uncert=None, Color = None, ID = None, UUID = None)

RECC_System.FlowDict['F_10_12'] = msc.Flow(Name='fabscrapdiversion' , P_Start = 10, P_End = 12, Indices = 't,o,m,e', Values=None, Uncert=None, Color = None, ID = None, UUID = None)
```

```
RECC_System.FlowDict['F_12_5']  = msc.Flow(Name='secondary material consumption' , P_Start = 12, P_End = 5, Indices = 't,o,m,e', Values=None, Uncert=None, Color = None, ID = None, UUID = None)

RECC_System.FlowDict['F_12_0']  = msc.Flow(Name='excess secondary material' , P_Start = 12, P_End = 0, Indices = 't,o,m,e', Values=None, Uncert=None, Color = None, ID = None, UUID = None)

RECC_System.FlowDict['F_9_0']   = msc.Flow(Name='waste mgt. and remelting losses' , P_Start = 9, P_End = 0, Indices = 't,e', Values=None, Uncert=None, Color = None, ID = None, UUID = None)
```

The ODYM-RECC stock and stock change list:

```
RECC_System.StockDict['dS_0']   = msc.Stock(Name='System environment stock change', P_Res=0, Type=1, Indices = 't,e', Values=None, Uncert=None, ID=None, UUID=None)

RECC_System.StockDict['dS_1t']  = msc.Stock(Name='Forestry stock change, timber', P_Res=1, Type=1, Indices = 't,e', Values=None, Uncert=None, ID=None, UUID=None)

RECC_System.StockDict['S_1t']   = msc.Stock(Name='Forestry carbon stock, fuel wood', P_Res=1, Type=0, Indices = 't,c,e', Values=None, Uncert=None, ID=None, UUID=None)

RECC_System.StockDict['dS_1f']  = msc.Stock(Name='Forestry stock change, fuel wood', P_Res=1, Type=1, Indices = 't,e', Values=None, Uncert=None, ID=None, UUID=None)

RECC_System.StockDict['S_1f']   = msc.Stock(Name='Forestry carbon stock, timber', P_Res=1, Type=0, Indices = 't,c,e', Values=None, Uncert=None, ID=None, UUID=None)

RECC_System.StockDict['S_7']    = msc.Stock(Name='In-use stock', P_Res=7, Type=0, Indices = 't,c,r,g,m,e', Values=None, Uncert=None, ID=None, UUID=None)

RECC_System.StockDict['S_7_Nl'] = msc.Stock(Name='In-use stock', P_Res=7, Type=0, Indices = 't,c,l,L,m,e', Values=None, Uncert=None, ID=None, UUID=None)

RECC_System.StockDict['S_7_No']  = msc.Stock(Name='In-use stock', P_Res=7, Type=0, Indices = 't,c,o,O,m,e', Values=None, Uncert=None, ID=None, UUID=None)

RECC_System.StockDict['dS_7']   = msc.Stock(Name='In-use stock change', P_Res=7, Type=1, Indices = 't,c,r,g,m,e', Values=None, Uncert=None, ID=None, UUID=None)

RECC_System.StockDict['dS_7_Nl'] = msc.Stock(Name='In-use stock change', P_Res=7, Type=1, Indices = 't,c,l,L,m,e', Values=None, Uncert=None, ID=None, UUID=None)

RECC_System.StockDict['dS_7_No'] = msc.Stock(Name='In-use stock change', P_Res=7, Type=1, Indices = 't,c,o,O,m,e', Values=None, Uncert=None, ID=None, UUID=None)

RECC_System.StockDict['S_10']   = msc.Stock(Name='Fabrication scrap buffer', P_Res=10, Type=0, Indices = 't,c,o,w,e', Values=None, Uncert=None, ID=None, UUID=None)

RECC_System.StockDict['dS_10']  = msc.Stock(Name='Fabrication scrap buffer change', P_Res=10, Type=1, Indices = 't,o,w,e', Values=None, Uncert=None, ID=None, UUID=None)

RECC_System.StockDict['S_12']   = msc.Stock(Name='secondary material buffer', P_Res=12, Type=0, Indices = 't,o,m,e', Values=None, Uncert=None, ID=None, UUID=None)

RECC_System.StockDict['dS_12']  = msc.Stock(Name='Secondary material buffer change', P_Res=12, Type=1, Indices = 't,o,m,e', Values=None, Uncert=None, ID=None, UUID=None)
```

6.3.3. General description of resource efficiency strategies

The target values for a number of RE strategies are directly formulated in the expert group consensus approach and entered into the scenario target tables [4] and are documented there. For most of the ME strategies in the material industries and also lifetime extension, a homogenous presentation of the future rollout of resource efficiency strategies is needed. Therefore, we split each strategy representation into two parameters: a) the maximum potential and b) the extent to which the maximum potential is seized. The maximal potential is determined by literature review, expert interviews, and estimations; it is scenario-independent. The scenario- and time-dependent implementation levels are modelled in a stylized manner, by two-parameter implementation curves (Supplementary Figure 42).

A main motivation for this approach, instead of an economic model, is that the nature of the strategy implementation, i.e., whether it is implemented via economic incentives, regulations, or lifestyle changes, is yet unclear. Our approach allows to explore the sociometabolic consequences of a certain implementation pattern and estimate the technical potential, without prescribing or implying the nature of its implementation.

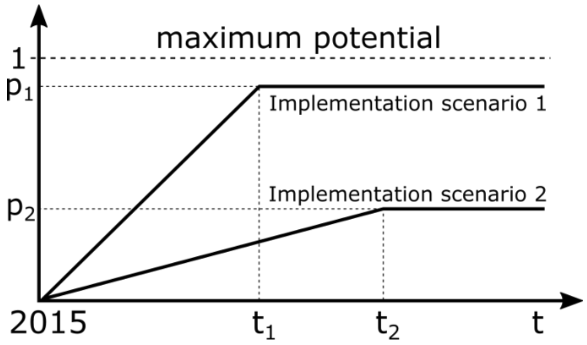

Supplementary Figure 42: Implementation curve for a resource efficiency strategy, with the total implementation level (in %) and the time of full implementation.

Example: Lifetime extension: With $\tau_0(c,r,g)$ being the BAU (business as usual) product lifetime for future age-cohorts of products, the maximum lifetime extension potential can (and will) be defined as percent increase from the base value. For each material efficiency scenario S, the actual product lifetime, $\tau_{act}(c,r,g,S)$, is then determined as the sum of the original and the product of implementation curve *IC*, maximal implementation potential ϕ, and base lifetime:

$$\tau_{act}(c,r,g,S) = \tau_0(c,r,g) + IC_\tau(\mathrm{t=c,r,g},S)\cdot\Phi_\tau(\mathrm{r,g})\cdot\tau_0(c,r,g) \quad (1)$$

For already existing age-cohorts we have to decide whether those are affected and if so, how the remaining lifetime scales with ϕ. Similar equations are defined below for all ME parameters.

The model equations below are formulated for the parameters without explicit scenario indices. If a scenario is to be calculated, the baseline parameters are simply replaced by their scenario values, and for each RE-relevant parameter, we specify how exactly the maximal implementation potential ϕ is defined (e.g., whether it is a maximum percentage increase or an absolute increase).

The RE scale-up curve is applied to all regions and climate policy scenarios. The following strategies are modelled this way:

- **ULD: Using less material by design, reduction of cement content only.**
  [3_SHA_RECC_REStrategyScaleUp, 3_SHA_CementContentReduction]

- **LTE: Lifetime extension** *[3_SHA_RECC_REStrategyScaleUp, 6_PR_LifeTimeExtension_passvehicles, 6_PR_LifeTimeExtension_resbuildings, 6_PR_LifeTimeExtension_nonresbuildings, 6_PR_LifeTimeExtension_nonresbuildings_g, 6_PR_LifeTimeExtension_appliances, 6_PR_LifeTimeExtension_industry]*
- **ReU: Re-use, residential and non-residential buildings only** *[3_SHA_RECC_REStrategyScaleUp, 6_PR_ReUse_Bld,* 6_PR_ReUse_nonresBld*]*
- **FYI: Fabrication scrap reduction *(yield improvement)*** *[3_SHA_RECC_REStrategyScaleUp, 6_PR_FabricationYieldImprovement]*
- **EoL: Improved recovery efficiency of scrap from end-of-life (EoL) products** *[3_SHA_RECC_REStrategyScaleUp, 6_PR_EoL_RR_Improvement]*
- **FSD: Fabrication scrap diversion** *[3_SHA_RECC_REStrategyScaleUp, 6_PR_FabricationScrapDiversion]*

Below the basic model equations and the different resource efficiency strategies (RES) are introduced, cf. also Supplementary Figure 41.

6.3.4. The use phase module (UP)

The use phase in ODYM-RECC can run as both, an inflow-driven and a stock-driven model, the latter meaning that the starting point for all future material cycle modelling are the exogenous trajectories for the in-use stock $S_7(t,\mathrm{r},G,\mathrm{S}) = S_{fut}(t,\mathrm{r},G,\mathrm{S})$, which is calculated from the future per-capita stock (lower case *s*) and the population.

$$S_7(t,r,G,S) = P(t,r,S) \cdot s_7(t,r,G,S) \quad (2)$$

For some stocks, the physical stock unit is also the unit of the service, e.g., m^2 of residential building space, and for others, the intensity of use needs to be factored in, e.g., for vehicles (km/yr). For passenger transport, the future annual passenger-km are converted to vehicle-km first, using the occupancy rate, and then to stocks, using kilometrage (Fig. 3.4), cf. also the transport model docu. We determine the future per capita service flows (vehicles) and residential building stocks from the starting value in 2015 combined with the target values in the scenario target table. To avoid model artefacts the value of the extrapolation function in the year 2015 must be equal to the actual stock in that year:

$$S_{2015}(2015,c,G,r) = S_7(2015,r,G,S) \quad (3)$$

We now introduce the model equations in the order they are implemented and executed in the ODYM-RECC model script. All system variables are scenario-dependent, and the index *S* is therefore omitted below.

Passenger vehicles: translating service into stock, implementing car-sharing and ride-sharing: With the carsharing and ridesharing parameters the total passenger-km are divided into four sections: First, we divided into passenger-vehicle mobility provided by privately owned vs carsharing cars and second, we divided into cars with normal occupancy rate and ride-shared cars (model parameters *CaS* and *RiS*). While the carsharing sector has a different vehicle kilometrage than privately owned cars, the ridesharing sector has a different occupancy rate, all described by the model parameters *COS* and *ORS*. From these ratios and parameters the total vehicle-km driven are calculated and by dividing them by the annual kilometrage, the required per capita vehicle stock $s_7(t,r,G)$ for G = 0 is calculated.

The modelling approach is documented in detail below. For details regarding the data used, please check the transport model documentation.

Step I: Convert passenger-km into vehicle-km: For the ODYM-RECC scenarios, we assume that the number of total passenger-km travelled per capita and year, $FUNCT_{exog\_fut}$, is given as exogenous parameter, after having considered total transport demand (sufficiency) and modal split. The following accounting equation for any given subset *x* of the total vehicle stock links vehicle stock s_x with annual kilometrage vkm_x, the occupancy rate OR_x (passengers per vehicle, driver only counts if he/she also benefits from travel service), and the delivered passenger-km PKM_x. It holds for each sector and for the total stock:

$$PKM_x = OR_x \cdot \mathrm{VKM}_x \cdot \mathrm{s}_x \quad (4)$$

The total car fleet as well as the total person-km can now be split in to the four sectors:

- Mobility by privately-owned and not shared vehicle stock (index $_0$)
- Mobility by car-shared but not ride-shared vehicle stock (index $_{CaS}$)
- Mobility by ride-shared but not car-shared vehicle stock (index $_{RiS}$)
- Mobility by car-shared and ride-shared vehicle stock (index $_{CaS+RiS}$)

We break down the total per capital vehicle stock and delivered PKM into these sectors, assuming that there are only two split parameters, one for car-sharing, and one for ride-sharing, that apply to the entire transportation demand. In other words, the share of ride-shared passenger-km is the same for privately owned and car-sharing cars, and vice versa, the share of car-shared passenger-km is the same for privately owned and ride-sharing cars:

$$\begin{aligned} PKM &= PKM_0 + PKM_{CaS} + PKM_{RiS} + PKM_{CaS+RiS} \\ &= OR_0 \cdot VKM_0 \cdot \mathrm{s}_0 \\ &+ OR_{CaS} \cdot VKM_{CaS} \cdot \mathrm{s}_{CaS} \\ &+ OR_{RiS} \cdot VKM_{RiS} \cdot \mathrm{s}_{RiS} \\ &+ OR_{CaS+RiS} \cdot VKM_{CaS+RiS} \cdot \mathrm{s}_{CaS+RiS} \end{aligned} \quad (5)$$

With the following model approaches and assumptions for *OR* and *VKM*:

$$\begin{aligned} OR_0 &= OR_{CaS} \\ OR_{CaS+RiS} &= OR_{RiS} =: \begin{cases} ORS + OR_0, \text{ global study} \\ ORS \cdot OR_0, \text{ Germany study} \end{cases} \end{aligned} \quad (6)$$

Here, *ORS* is the increment (global study) or factor (Germany case study) by which *OR* increases under ride-sharing (current value: 1.4, cf. transport model docu). It is converted to the subsequently used ORS_{act}:

$$ORS_{act} = \begin{cases} \dfrac{ORS + OR_0}{OR_0}, \text{ global study} \\ ORS, \text{ Germany study} \end{cases} \quad (7)$$

And for the kilometrage:

$$\begin{aligned} VKM_0 &= \mathrm{VKM}_{RiS} \\ VKM_{CaS+RiS} &= \mathrm{VKM}_{CaS} =: \frac{VKM_0}{COS} \end{aligned} \quad (8)$$

Here, *COS* is the factor by which car ownership decreases under car-sharing (current value: 2.0, cf. transport model docu), and, as a consequence, the utilisation or annual kilometrage of each car-sharing car goes up by a factor of *1/COS*.

From these definitions and simplifications, it follows:

$$PKM = \mathrm{OR}_0 \cdot \mathrm{VKM}_0 \cdot \left(s_0 + COS^{-1} \cdot s_{CaS} + ORS \cdot s_{RiS} + ORS_{act} \cdot COS^{-1} \cdot s_{CaS+RiS} \right) \quad (9)$$

With the following definitions for the ride-sharing and car sharing-based PKM in the total PKM:

$$CaS := \frac{PKM_{CaS} + PKM_{CaS+RiS}}{PKM} \quad (10)$$

and

$$RiS := \frac{PKM_{RiS} + PKM_{CaS+RiS}}{PKM} \quad (11)$$

and the assumption that these shares are homogenous across all sectors (e.g., the share of ride-shared PKM in the car-shared PKM is the same as the share of ride-shared PKM in the total PKM etc. pp), we can write:

$$\begin{aligned} PKM &= 1 \cdot PKM \\ &= 1 \cdot 1 \cdot PKM \\ &= \left(CaS + 1 - CaS \right) \cdot \left(RiS + 1 - RiS \right) \cdot PKM \\ &= \left[CaS \cdot RiS + \left(1 - CaS\right) \cdot RiS + \mathrm{CaS} \cdot \left(1 - RiS\right) + \left(1 - CaS\right) \cdot \left(1 - RiS\right) \right] \cdot PKM \end{aligned}$$

(12)

where each of the terms denotes the PKM delivered by one of the four sector of the stock listed above.

Hence, we can calculate the size of the stock sectors directly from equating the respective terms:

$$\begin{aligned} s_0 &= \left(1 - CaS\right) \cdot \left(1 - RiS\right) \cdot PKM \;/\; \left(OR_0 \cdot VKM_0\right) \\ s_{CaS} &= COS \cdot CaS \cdot \left(1 - RiS\right) \cdot PKM \;/\; \left(OR_0 \cdot VKM_0\right) \\ s_{RiS} &= \left(1 - CaS\right) \cdot RiS \cdot PKM \;/\; \left(ORS_{act} \cdot OR_0 \cdot VKM_0\right) \\ s_{CaS+RiS} &= COS \cdot CaS \cdot RiS \cdot PKM \;/\; \left(ORS_{act} \cdot OR_0 \cdot VKM_0\right) \end{aligned} \quad (13)$$

Note, that these calculations work both at the per capita stock and at the total stock level. Here, per capita stock levels are calculated and later multiplied with the scenario-specific population parameter.

From this result, we can calculate the total stock needed as the sum of the sectors, the resulting average VKM, and the resulting average OR:

$$\begin{aligned}
s_{fut} &= s_0 + s_{CaS} + s_{RiS} + s_{CaS+RiS} \\
VKM_{fut} &= \left[s_0 + COS^{-1} \cdot s_{CaS} + s_{RiS} + COS^{-1} \cdot s_{CaS+RiS} \right] \cdot VKM_0 / s_{fut} \\
OR_{fut} &= SERV_{exog_fut} / \left[s_{fut} \cdot VKM_{fut} \right] \\
PKM &\equiv SERV_{exog_fut} \\
OR_0 &\equiv OR_{exog}(t) \\
VKM_0 &\equiv IO_{exog}(t)
\end{aligned} \tag{14}$$

Finally, the model parameters are linked to the following datasets: The future *PKM* is given by the exogenous parameter $SERV_{exog\_fut}$, and the future baseline (no RiS) occupancy rate and the future baseline (no CaS) vehicle kilometrage are specified via the scenario target table approach as well (via $OR_{exog}(t)$ and $IO_{exog}(t)$). The results s_{fut} and VKM_{fut} enter the subsequent ODYM-RECC model calculations as parameter time series.

Also, the lifetime distribution needs to be modified. Assuming a constant total vehicle kilometrage over the entire vehicle lifetime, the lifetime of car sharing cars scales with the *COS* parameter. Hence, there will be a bi-modal lifetime distribution, which we can simplify by calculating the resulting average new lifetime τ_{eff} from the original lifetime τ_0 according to the share of car-sharing cars in the fleet, which is also an ODYM-RECC parameter

$$\tau_{eff} = \tau_0 \cdot s_{fut}^{-1} \cdot \left(s_0 + COS \cdot s_{CaS} + s_{RiS} + COS \cdot s_{CaS+RiS} \right) \tag{15}$$

The setup described above allows us to apply car-sharing and ride-sharing as to independent strategies and to calculate the effect of either of them not being implemented.

Residential and nonresidential buildings: More intense use of floorspace: Unlike service sufficiency, which leads to a reduction of stocks due to lower service demand, a more intense use of products means that total service demand remains constant but is achieved with smaller stocks. Examples include car-sharing, shared office spaces, denser urban form, parents moving to smaller apartments when their kids move out, and a higher occupancy rate in public transport. With the more intense use potential ϕ_{MIU} defined as the maximum share of the original stock that can be reduced, resulting new stock is then given below:

$$\begin{aligned}
s_{7_MIU}(t,r,G) &= \left(1 - IC_{MIU}(t) \cdot \Phi_{IU}(G)\right) \cdot s_7(t,r,G) \\
S_{7_MIU}(t,r,G) &= P(t,r) \cdot s_{7_MIU}(t,r,G)
\end{aligned} \tag{16}$$

Here, IC_{MIU} is the ramp-up curve for the more intense use of the building stock. Unlike the material-related ME strategies, which are ramped up with a function that is a sequence of linear changes, the more intense use of buildings needs a smoother curve as sudden changes in the derivative of the

stock curve cause jumps in the material flows. Hence, for IC_{MIU} a slower ramp up by 2050 and a subsequent splint interpolation is applied.

All products: Product lifetime extension: The lifetime of new and existing products is prolonged, due to more robust design that allows for easier exchange for parts that wear down quicker than the structural components, or that change more rapidly than the latter due to changes in consumer preference (fashion) or safety standards.

a) Future age-cohorts:

$$\tau_{act}(c,r,g) = \left(1+\Phi_{\tau}(\mathrm{r,g})\right)\cdot\tau_0(c,r,g), \quad \text{for } c \geq 2016 \quad (17)$$

Here, a scenario-independent lifetime extension of future ago-cohorts is modelled, which acknowledges that the lifetime extension of newly produced products only will have a measurable effect in the longer run, after the average product lifetime will have passed. The probability density function of a product leaving the stock (pdf_{act}) is then determined according to which lifetime distribution model is set, and with the average lifetime equal to τ_{act}.

b) Past (historic) age-cohorts: For the historic age-cohorts, whose post-2016-phase out is modelled, it is assumed that the mean lifetime shifts gradually according to

$$\tau_{act}(c,r,g) = \left(1+LC(c)\cdot\Phi_{\tau}(\mathrm{r,g})\right)\cdot\tau_0(c,r,g), \text{ for } c < 2016 \quad (18)$$

Here, *LC(c)* is a linear curve from 0 for the 1900 age-cohort (*c=0*) to 1 for the 2015 age-cohort. This approach acknowledges that the potential for changing the mean product lifetime is largest for recent age-cohorts. Older age-cohorts, especially for buildings, are largely preserved as they are already on the 'long tail' of their lifetime distribution.

Passenger vehicles and buildings: Stock-driven model: With the parameters prepared according to the equations above, we can apply a stock-driven model [23], which is implemented as part of ODYM in the class `dynamic_stock_model` [6]. The computations are done model year by model year, starting with the historic stock in the first year. First, the stock from the last model year is transferred to the present year (ageing).

$$S_7^*(t,c,r,g)) = S_7(t-1,c,r,g) \quad (19)$$

Then, the outflow of the existing stock is computed and subtracted from the preliminary stock S_7*, and corrected for lifetime extension of historic age-cohorts. Here, pdf_{act} is the probability of discard from stock calculated from the lifetime after including lifetime extension.

$$\begin{aligned} F_{7\_8}(t,\mathrm{c},r,g) &= S_7^*(t,c,r,g)\cdot pdf_{act}(t-c,r,g) \\ F_{7\_8}(t,r,g) &= \sum_c F_{7\_8}(t,\mathrm{c},r,g) \qquad (20) \\ S_7(t,c,r,g) &= S_7^*(t,c,r,g) - F_{7\_8}(t,\mathrm{c},r,g) \end{aligned}$$

In a stock-driven model, the total current stock must equal the exogenously specified value. The inflow (apparent consumption) necessary to maintain and expand the stock is obtained and added as youngest age-cohort to the existing stock. Below, we sum up over all products *g* belonging to a certain product group/sector *G*:

$$F_{6\_7}(t,G,r) = S_{7\_MIU}(t,G,r) - \sum_{c,g \in G} S_7(t,c,r,g)/1yr$$
$$S_7(t,c=t,G,r) = F_{6\_7}(t,G,r) \cdot 1\,yr \quad (21)$$

The inflow of total products (index G) is split into different types (of vehicles, buildings, etc., index g) with the type split:

$$F_{6\_7}(t,r,g) = TS(t,r,G,g) \cdot F_{6\_7}(t,r,G) \quad (22)$$

Product material composition and energy use in use phase: Products can be light-weighted by better design, downsizing, or different material choices and substitution. Two strategies that change the material composition of products are considered: A reduction of weight per product via downsizing, i.e., smaller vehicles, and a reduction via material substitution. e.g., aluminium for steel in vehicles or timer for concrete in buildings.

A number of vehicle archetypes was simulated. For the six vehicle types, there are four segments (microcar, passenger car, minivan/SUV, and light truck) that come in two versions each: one with conventional material choice and one with a material substituted design. 6 * 4 * 2 = 48 archetypes in total.

A number of building archetypes was simulated, there are four archetypes for each building type: one for a standard building, one for a lightweight design, one for a material substituted, and one for a lightweight design and material-substituted archetype. In addition, the building archetypes are region-dependent to account for different climates and building conventions.

The different archetypes are then scaled up using the share of downsized and light-weighted prototypes, respectively, as shown in the equations below. The ULD and MSu strategies are used to model a switch to different archetypes of products by changing the mix of archetypes and calculate the resulting changes in material composition and operational energy consumption of the *average* product from a given age-cohort:

+ Vehicles:

$$\begin{aligned} &\mu(c,m,p,r) = \\ &\sum_s DS(G,s,r,c=t) \cdot MS(G,r,c=t) \cdot MA_{LWE}(s,p,m) + \\ &\sum_s DS(G,s,r,c=t) \cdot \left(1 - MS(G,r,c=t)\right) \cdot MA_{conv}(s,p,m) \end{aligned} \quad (23)$$

$$\begin{aligned} &EI(c,p,n,r) = \\ &\sum_s DS(G,s,r,c=t) \cdot MS(G,r,c=t) \cdot EIA_{LWE}(s,p,n) + \\ &\sum_s DS(G,s,r,c=t) \cdot \left(1 - MS(G,r,c=t)\right) \cdot EIA_{conv}(s,p,n) \end{aligned} \quad (24)$$

In the equations above, G is the sector that p belongs to, and MA_{conv} is the material composition of the conventionally designed archetypes without material substitution, and MA_{LWE} the MC of the material-substituted archetypes, same for the EIA parameters.

+ Buildings (residential and nonresidential):

$$\begin{aligned}
&\mu(c,m,B,r)= \\
&DS(G,r,c=t)\cdot MS(G,r,c=t)\cdot MA_{LWE_MSu}(B,r,m)+ \\
&DS(G,r,c=t)\cdot\left(1-MS(G,r,c=t)\right)\cdot MA_{LWE}(B,r,m)+ \\
&\left(1-DS(G,r,c=t)\right)\cdot MS(G,r,c=t)\cdot MA_{MSu}(B,r,m)+ \\
&\left(1-DS(G,r,c=t)\right)\cdot\left(1-MS(G,r,c=t)\right)\cdot MA_{conv}(B,r,m)
\end{aligned} \quad (25)$$

$$\begin{aligned}
&EI(c,n,V,B,r)= \\
&DS(G,r,c=t)\cdot MS(G,r,c=t)\cdot EIA_{LWE_MSu}(B,r,V,n)+ \\
&DS(G,r,c=t)\cdot\left(1-MS(G,r,c=t)\right)\cdot EIA_{LWE}(B,r,V,n)+ \\
&\left(1-DS(G,r,c=t)\right)\cdot MS(G,r,c=t)\cdot EIA_{MSu}(B,r,V,n)+ \\
&\left(1-DS(G,r,c=t)\right)\cdot\left(1-MS(G,r,c=t)\right)\cdot EIA_{conv}(B,r,V,n)
\end{aligned} \quad (26)$$

In the equations above, G is the sector that B belongs to, and MA_{conv} is the material composition of the conventionally designed archetypes without material substitution and lightweight design, MA_{MSu} is the material composition of the material-substituted archetypes, MA_{LWE} is the material composition of the lightweight design archetypes, and MA_{LWE_MSu} the MC of the material-substituted *and* lightweight design archetypes, same for the EIA parameters.

While the material composition is known from the scenario parameters at the start of the model run, the elemental composition of materials needs to be determined from the available waste flows and their remelting, together with the required primary production to satisfy total material demand.

That means that the material composition for chemical elements together (recorded under element 0, 'all'), can be calculated from the total material composition parameter

$$\mu(c,r,g,m,e=0,S) \quad (27)$$

at any point in the model, whereas the elemental breakdown needs to be determined after the material cycle have been closed at the total mass level. Hence, a loop over all future model years is programmed.

Different vintages of materials and different flows in the system have different chemical element composition of the materials they contain. For example, the element composition of the materials entering manufacturing (primary production and secondary materials) each have their own element composition, and the composition of the newly manufactured goods is the mass-weighted average of the two input values.

If re-use of products is present, the element composition of the final consumption flow is different of the manufacturing outflow material composition. Also here, a weighted average is computed to ensure the mass balance at the chemical element level also here.

With the product material composition parameters calculated above, the product flows can be converted to material flows at any time during the model run, e.g.:

$$F_{6\_7}(t,r,g,m)=\mu(t,g,r,m)\cdot F_{6\_7}(t,r,g) \quad (28)$$

Modelling of building renovation: ODYM-RECC v2.4 contains a simplified representation of renovation/refurbishment of residential and non-residential buildings to lower energy standards. This mechanism was implemented to allow us to create realistic scenarios for future energy consumption and GHG, and it enables us to apply building lifetime extension also to historic age-cohorts (cf. above).

The implementation of building renovation changes the specific energy consumption parameter *EI* to lower values, using three parameters: the Maximum building renovation potential (*MRP*), the Energy saving under building renovation parameter (*ESP*), and the building renovation implementation curves *ICBR*. Through renovation, the *EI* parameter becomes time-dependent, which is then considered in the subsequent equations where the total energy demand is calculated. The equation below is written for the residential building types *B*, and the same equation is implemented also for the non-residential building types *N*.

$$\begin{aligned} EI(t,c,n,V,B,r,S,R) &= EI(c,n,V,B,r,S,R)\cdot \\ &(1-MRP(r,c,B)\cdot ESP(r,S,B)\cdot ICBR(R,o=0,t,S)) \end{aligned} \quad (29)$$

Here, *o* is the index for the global aggregate region, its only value is 0 (for ‘global’).

The change of material composition over time due to renovation is calculated as follows:

$$\begin{aligned} \mu(t,c,m,B,r) &= \mu(c,m,B,r)\cdot\left(1+MRP(r,c,B)\cdot ICBR(o=0,t)\cdot \mu Rrel(c,m,B,r)\right)+ \\ &MRP(r,c,B)\cdot ICBR(o=0,t)\cdot \mu Rabs(c,m,B,r) \end{aligned} \quad (30)$$

Because the material composition of building now changes with time and is not constant for a given age-cohort anymore, the material inflows into the use phase have to be re-calculated using the mass balance, where *diff* is the discrete difference:

$$F_{6\_7}(t,r,B,m) = \sum_c diff\,(S_7(t,c,r,B,m)) + F_{7\_8}(t,c,r,B,m) \quad (31)$$

Industrial assets and appliances, inflow-driven model: Unlike for passenger vehicles and buildings, for the two sectors industrial assets (electricity generation and appliances) the annual inflow of new products is given from other scenario modelling projects [60,61]. With the probability of discard pdf_{flow} (calculated after applying lifetime extension if activated), the lifetime model is used to determine both the accumulation of in-use stocks and the generation of EoL products:

$$F_{6\_7}(c,r,g) \equiv F_{Fut}(c,r,g) \quad (32)$$

$$\begin{aligned} F_{7\_8}(t,c,r,g) &= F_{6\_7}(c,r,g)\cdot pdf_{flow}(t-c,r,g) \\ S_7(t,c,r,g) &= F_{6\_7}(t,c,r,g)\cdot\left(1-\sum_{c\le t'\le t} pdf_{flow}(t'-c,r,g)\right) \\ S_7(t,r,g) &= \sum_c S_7(t,c,r,g) \end{aligned} \quad (33)$$

With the given product material composition parameters the product flows can be converted to material flows at any time during the model run, e.g.:

$$F_{7\_8}(t,c,r,g,m) = \mu(c,g,r,m) \cdot F_{7\_8}(t,c,r,g) \quad (34)$$

6.3.5. The waste management and recycling module (WR)

Obsolete stock formation and obsolete stock formation reduction [currently not implemented]: A fraction of the products and buildings that leaves the use phase is not made available for reuse or material recovery. These obsolete stocks are determined with a dedicated parameter and a corresponding reduction strategy

$$F_{8\_0}(t,r,g,) = \left(1 - IC_{OBS}(\mathrm{t,r,g}) \cdot \Phi_{OBS}(\mathrm{r,g})\right) \cdot OBS(\mathrm{t,r,g}) \cdot F_{7\_8}(t,r,g) \quad (35)$$

Re-use of end-of-life (EoL) products: A fraction of the available end-of-life products can be re-used, which is modelled with a re-use factor diverting products away from waste management and re-inserting them back into the market for final products:

$$F_{8\_17}(t,r,g) = IC_{\mathrm{Re}Use}(t,r,g) \cdot \Phi_{\mathrm{Re}Use}(r,g) \cdot \left(F_{7\_8}(t,r,g) - F_{8\_0}(t,r,g)\right) \quad (36)$$

The complement of the obsolete stock formation and re-use,

$$F_{8\_9}(t,r,g,m) = F_{7\_8}(t,r,g,m) - F_{8\_0}(t,r,g,m) - F_{8\_17}(t,r,g,m) \quad (37)$$

is sent to the waste management industries for treatment. Analog equations apply to the sectors with 11 and one world region (appliances and aggregate nonresidential buildings: index o and industry (electricity generation): index l).

Waste management is modelled as a cascade: first, scrap is extracted from the end-of-life (EoL) products that are sent to the waste management industries. This is modelled by a simple factor end-of-life recovery rate.

$$F_{9\_10}(t,w,e) = \sum_{g,m,W,r} EoL\_RR(g,r,m,w,W) \cdot F_{8\_9}(t,g,r,m,e) \quad (38)$$

At this point, the element composition of the flows is still known, since only EoL products with historic or previously determined (earlier in the for loop over t) age-cohort are contained in this flow.

End-of-life recovery rate improvement: The current EoL-RR values can be improved by better dismantling and sorting. This effect is modelled by a separate RE strategy:

$$EoL\_RR(g,r,m,w,W) = EoL\_RR(g,r,m,w,W) + IC_{EoL}(t,r,g) \cdot \Phi_{EoL}(g,r,m,w,W) \quad (39)$$

Here, the improvement potential Φ_{EoL} is measured in percentage points by definition, so that it can be directly added to the baseline value.

Moreover, since waste and scrap can be traded, the regional dimension is no longer considered here and is collapsed. In the equations, *r* is thus not shown as aspect for the material flows at global scale, but in the model, the values are assigned to the region ‘World’ with index letter *o*.

The fabrication scrap flow from last year, which is buffered as stock on the scrap market, is added to the resulting old scrap flow (but quality differences are kept by distinguishing between the different scrap and material classes *w* and *m*). The sum of these flows is then sent to re-melting (also part of process 9), from where the recycled material flow is determined by the parameter re-melting yield RMY:

$$F_{9\_12}(t,m,e) = \sum_{W,w} RMY(w,m,e,W,t) \cdot \left(F_{9\_10}(t,w,e) + F_{5-10}(t-1,w,e) \right) \quad (40)$$

From the above equation, it also becomes clear why the introduction of the time lag for the scrap flow simplifies the computation: As the elemental composition of the fabrication scrap of last year is already known, one can directly compute the elemental composition of the secondary material produced during the current year.

6.3.6. Link to function provision, energy consumption, and environmental extensions/pressures (module EX)

To link the stocks to function provision type V and use phase energy consumption the model follows the scheme shown in Fig. 3.4. The following equations are used, and the parameters therein are explained in Table 6.4 below. Below is the general equation for linking a stock to a function provided:

$$F_{S7}(t,c,r,g,V) = IU(t,c,r,g,V) \cdot IO(t,c,r,g,V) \cdot S_7(t,c,r,g) \quad (41)$$

For passenger vehicles, the *intensity of operation (IO)* parameter denotes the annual kilometrage, and for buildings, IO denotes the share of the built-up area that provides building services: heating, cooling, and domestic hot water generation, all at a standard level for which average specific energy consumption is reported.

For passenger vehicles, the *intensity of use (IU)* parameter denotes the occupancy rate (average number of people per car) and for buildings, IU denotes the number of building occupants enjoying a certain number degree-days of thermal comfort if the service unit is thermal comfort, and 1 if the service unit is simply m^2 of living space, all per m^2.

The direct energy consumption is then determined by multiplying the specific energy consumption (*energy intensity EI,* energy intensity of service type V) of operating the products to the intensity of use of the stock. Then, the result is multiplied with *ECS, the energy carrier split* of energy consumption for delivering service type V into energy carrier n. The *EI* parameter is time-dependent in the case where building renovation is considered. Else, it is only age-cohort dependent. For vehicles, the following equation applies:

$$E_{16\_7}(n,t,r,g,V) = \sum_{c} ECS(n,c,r,g,V) \cdot EI((t),c,r,g,V) \cdot IO(t,c,r,g,V) \cdot S_7(t,c,r,g)$$

(42)

For building, an additional calculation step is necessary to consider the conversion efficiency from final energy (i.e., energy delivered to the building like electricity or natural gas) to useful energy (i.e., energy delivered for building function like heat in heated air). Therefore, the parameter 4_TC_ResidentialEnergyEfficiency (here written as building energy conversion *BEC*) was introduced. The calculation is not straight forward because from EI, we only know the useful energy demand for

all energy carriers. This *EI(all)* then needs to be multiplied with the energy carrier split for useful energy, which is unknown as the given *ECS* is for final energy:

$$\begin{aligned} E_{16\_7}(n) &= BEC(n)\cdot ECS_{useful}(n)\cdot E_{useful}(all) \\ E_{16\_7}(n) &= ECS_{final}(n)\cdot E_{16\_7}(all) \end{aligned} \qquad (43)$$

These equations are resolved for *ECS(useful)* as follows, with *Anc(n)* as ancillary quantity:

$$\begin{aligned} Anc(n) &:= ECS_{final}(n)/BEC(n) \\ ECS_{useful}(n) &= Anc(n)/\sum_n Anc(n) \end{aligned} \qquad (44)$$

The final equation for buildings is then:

$$E_{16\_7}(n,t,r,g,V) = \sum_c \begin{matrix} BEC(n,r,t,V)\cdot ECS_{useful}(n,t,r,V)\cdot \\ EI((t),c,r,g,V)\cdot IO(t,c,r,g,V)\cdot S_7(t,c,r,g) \end{matrix} \qquad (45)$$

In the model code, the product BEC x ECS is pre-multiplied and normalized, leading to a factor *3_SHA_EnergySupply_Buildings (ESB(n))*, which is calculated by scaling the resulting *ECSuseful* to directly yield the final energy flow *FinalEnergy* per 1 MJ of useful energy demanded and applied as follows:

$$\begin{aligned} & with \\ & \sum_n Anc(n) =: U \\ & and \\ & FinalEnergy = \sum_n BEC(n)\cdot ECS_{useful}(n) \\ & we\ find \\ & ESB(n) = ECS_{useful}(n)\cdot FinalEnergy \\ & = ECS_{useful}(n)\cdot \sum_n BEC(n)\cdot ECS_{useful}(n) \\ & = ECS_{final}(n)/(U\cdot BEC(n))\cdot \sum_n BEC(n)\cdot ECS_{final}(n)/(U\cdot BEC(n)) \\ & = ECS_{final}(n)/(U\cdot BEC(n))\cdot \sum_n ECS_{final}(n)/U \\ & = \frac{ECS_{final}(n)}{BEC(n)\cdot U^2} \end{aligned} \qquad (46)$$

For vehicles, EI is measured in MJ/km driven, for building services in kWh per m^2 and year. Energy Flow E_{16_7} is then multiplied with the scenario-specific emissions factors to obtain the use phase carbon footprint.

Supplementary Table 25: Coupling between stock S_7, function provision F_{S7}, and energy consumption E_{16_7}.

Sector	Function flow unit and description	Intensity of operation (IO) unit and description	Intensity of use (IU) unit and description	Energy intensity unit and description	Product stock unit and description
**Passenger vehicles**	Passenger-km/yr	km/vehicle/year	Passengers per vehicle	MJ/km	Vehicles (product of vehicle ownership and population)
**Residential buildings**	Person-comfort m^2*yr /yr	Share of built area that provides services: heating, cooling, hot water access	1	kWh/m^2/yr	m^2 of residential buildings (product of per capita floor space and population)
**Non-residential buildings**	Comfort m^2*yr /yr	Share of built area that provides services: heating, cooling, hot water access	1	kWh/m^2/yr	m^2 of non-residential buildings (product of per capita floor space and population)

Process emissions, direct emissions, and indirect emissions of energy supply are considered by defining appropriate emissions and energy intensity factors. These are multiplied to the material and service flows, e.g., the energy flow into manufacturing and the related GHG emissions of its supply:

$$E_{16\_5}(n,t,o) = \sum_{g,r} EIM(F = g, n, t = c, o) \cdot F_{5\_6}(t,r,g) \quad (47)$$

$$GHG_{5i}(X,t) = \sum_{n,o(=0)} GHGW(X,n,o,t) \cdot E_{16\_5}(n,t,o) \quad (48)$$

Here, the subindex 'i' denotes the indirect emissions scope. Analog equations apply to the other processes (waste management and remelting, primary material production) and the other emissions types: direct combustion and process emissions. In particular, for energy supply, we use results from the MESSAGE IAM plus a backstop parameter for low carbon electricity (minimum supply chain CO_2 emissions as we do not consider negative emissions technologies here).

$$\begin{aligned}
GHGE_{act}(X,n,r,t) &= \max(GHGE(X,n,r,t), GHGBS(X,n,t)) \\
GHGW_{act}(X,n,o,t) &= \max(GHGW(X,n,o,t), GHGBS(X,n,t)) \\
GHG_{P,i}(X,t) &= \sum_{n,o(=0)} GHGE_{act}(X,n,r,t) \cdot E_{16_P}(n,t,r) \\
GHG_{P,i}(X,t) &= \sum_{n,o(=0)} GHGW_{act}(X,n,o,t) \cdot E_{16_P}(n,t,o)
\end{aligned} \quad (49)$$

Here, the sub-indices 'P,i' denote the indirect emissions scope of a process P (use phase, manufacturing, etc.)

Environmental pressures, characterisation factors: From the GHG flows the different pressure indicators (here: global warming metrics (GWP 100/500, GTP 100/500)) are determined by multiplication with the characterisation factors (here: for emissions from a process P):

$$\text{Pressure}_{\text{P}}(x) = \sum_{X} CF(x, X) \cdot GHG_P(X) \quad (50)$$

6.3.7. Manufacturing (MF module) and the closure of the recycling loop

Merger of the different regional scopes: ODYM-RECC allows the user to depict different end-use sectors with different regional resolutions. That means that the use phase, re-use and waste management flows (F_{x_7}, F_{7_x}, F_{x_17}, F_{17_x}, F_{x_9}, F_{9_10}) come in three versions with aspects r, l, and o, resp. For the total use of scrap by the recycling processes (F_{10_9}) they are merged into a global aggregate, and the region-specific demand for manufactured goods (F_{6_7}) is aggregated into a global manufacturing output (F_{5_6}).

The manufacturing process is described by two parameters: the manufacturing yield and the energy demand of manufacturing.

In the simplest case, the total material demand of manufacturing/construction (process 5), *F_x_5* is determined from manufacturing yield λ, material content μ, and product demand *F_5_6*:

$$F_{x\_5}(m,t,o) = \sum_{g,r} \frac{1}{\sum_{w} \lambda(m,w,g,F=g,t,o)} \cdot \mu(m,c=t,r,g) \cdot F_{5\_6}(t,r,g)$$

(51)

Resource efficiency in manufacturing: Several resource efficiency strategies apply (Fig. 3.4): Material substitution, which is depicted by exogenous scenarios; light-weighting of products, also depicted by exogenous scenarios; fabrication yield improvement, depicted by a resource efficiency parameter applied to the fabrication yield, and fabrication scrap diversion, which is modelled by a new flow *F_10_12* consisting of part of the fabrication scrap that is assumed to have a quality and workability that makes it a suitable input to other manufacturing sectors.

The change of the manufacturing yield is modelled in the same manner as the other RE strategies affecting process parameters.

The primary production is now determined from the mass balance, assuming that all available secondary material is used first. No rebound effects of recycling [51,52] are considered here.

$$F_{3\_4}(m,t,o) = F_{x\_5}(m,t,o) - F_{12\_5}(m,t,o) \quad (52)$$

If there is excess supply of secondary material for the sectors studied (e.g. more construction steel cascaded from EoL vehicle steel than needed in new residential buildings), the affected elements of *F_3_4* are set to zero and the excess secondary material is exported from the system via the flow *F_12_0* instead.

6.3.8. Link to material composition of products and materials (ME module)

One central feature of ODYM is that it can work at different layers: Material, product, chemical element, etc. Because of the service perspective, the product stocks are modelled first. With the use

phase inflow and outflow of products known, one can add the material composition of products and the element content of materials, e.g.:

$$F_{8\_17}(t,r,g,m,e) = \mu(r,g,m,e) \cdot F_{8\_17}(t,r,g) \quad (53)$$

Check also the section **Product material composition and energy use in use phase** above, where the determination of the material composition parameter μ from archetype data is explained for convenience reasons.

The determination of the element composition of materials is calculated after the primary production *F_3_4* and the scrap export *F_12_0* have been determined. After this correction (the export of excess scrap), and since the element composition of both flows on the right side of the last equation is known, one can now also calculate the breakdown of the total material flows into individual chemical elements:

$$F_{x\_5}(m,t,e,o) = F_{3\_4}(m,t,e,o) + F_{9\_12}(m,t,e,o) + F_{10\_12}(m,t,e,o) - F_{12\_0}(m,t,e,o)$$

(54)

From that equation, the manufacturing output *F_5_6* and the fabrication scrap *F_5_10* can be broken down into individual chemical elements as well. The scrap contained in the latter flow will then be recycled in the next model year:

To simplify the computation of the material loops, it is assumed that all fabrication scrap is sorted and remelted in the following year, meaning that the time for a material passing through the recycling loop for fabrication scrap is one year. Internal scrap in remelting, so called home scrap, is not included as a separate flow, but indirectly via the loss rates and the energy consumption.

6.3.9. The primary material production (PP module)

Primary production (F_3_4) is determined as the amount of material required to fill the gap between demand from manufacturing and supply of secondary material from within the sector. With the exception of steel, the associated supply chain energy demand is not calculated in model version 2.3. Instead, the entire supply chain emissions *GHG_3* are calculated by multiplying *F_3_4* with *GHGPP*. For the four types of steel and the RCP 2.6 scenario, a gradual shift from coke-based primary steel to direct reduced hydrogen-based steel production is modelled, assuming a linear shift from 0% in 2030 to 100 % in 2070. For the share of steel production that is hydrogen based a certain amount of electricity (for machine operation and hydrogen production) is listed in the energy parameter 4_EI_ProcessEnergyIntensity.

For wood production, sustainable regrowth is assumed with a rotation period of 70 yr (timber) and 30 years (fuel wood) [84]. A carbon balance for forestry is established:

$$\begin{aligned} &F_{2\_3}(t, m = timber, e = 'C') = F_{3\_4}(t, m = timber, e = 'C') \\ &F_{2\_7}(t, e = 'C') = F_{15\_7}(t, n = 'woodfuel') / HHVwood \\ &F_{1\_2}(t, e = 'C') = F_{2\_7}(t, e = 'C') + F_{2\_3}(t, m = timber, e = 'C') \end{aligned} \quad (55)$$

In each year, the forest carbon C stock *S₁(e=C)* is reduced accordingly to deliver *F_1_2*. Timber and fuel wood have different rotation periods and are therefore described by two separate stocks. The regrowth (*F_0_1* sequestered by re-growing trees and added to the forest carbon stock) is modelled

with a simple forest growth model using the cumulative distribution function of the normal distribution with inflection point at 50% of the rotation period.

$$\begin{aligned}
&\text{Regrowth_fuel(t)} \quad = scipy.stats.norm.cdf\,(t, FRPfuel/2, FRPfuel/4)\\
&\text{Regrowth_wood(t)} = scipy.stats.norm.cdf\,(t, FRPwood/2, FRPwood/4)\\
&F_{0_1}(e='C', c, t, fuel) = F_{2_7}(c, e='C')\cdot\left(\text{Regrowth_fuel}(t-c)-\text{Regrowth_fuel}(t-1-c)\right)\\
&F_{0_1}(e='C', c, t, wood) = F_{2_3}(c, e='C')\cdot\left(\text{Regrowth_wood}(t-c)-\text{Regrowth_wood}(t-1-c)\right)\\
&F_{0_1}(e='C', t) = \sum_{c,\, fuel, wood} F_{0_1}(e='C', c, t, fuel/wood)
\end{aligned}$$

(56)

Here the third and fourth equation lines give the uptake of atmospheric carbon in year t for a harvest at vintage c for fuel or wood, and the bottom line shows that the total uptake *F_0_1(t)* is the sum over both harvest years c and use types (timber/fuel wood).

The fuel wood is burned in the year of harvest and the corresponding CO_2 released to the atmosphere:

$$GHG_{15}(t, woodfuel) = GHGD(X='CO2', n='fuelwood')\cdot F_{1\_2}(t, m=woodfuel, e='all') \quad (57)$$

This equation is part of the calculation of direct emissions from the use phase. The timber flows are stored in the use phase until they are discarded at end-of life. The non-recycled manufacturing waste and the EoL timber are assumed to be combusted in the waste mgt. industries and in some scenarios, electricity generation from wood combustion is modelled, which then substitutes 'regular' electricity from the grid.

$$\begin{aligned}
&GHG_{9_0}(t, m=wood) = \frac{44}{12}\cdot F_{9_0}(t, e='C')\\
&E_9(t, n=electricity) = \frac{Elwood}{CO2wood\cdot 12/44}\cdot F_{9_0}(t, e='C')
\end{aligned} \quad (58)$$

Above, the process chain 9-13-14 is abbreviated by modelling these flows as a single flow F_{9_0}. By this accounting of carbon stocks and flows in the system the actual carbon update and harvest in forests and the actual emissions from wood waste combustion are quantified and the need of aggregate factors like GWPbio [84] is not necessary in this time-explicit and large-scale modelling framework.

6.3.10. Mining industries (MR module)

Impacts from mining are currently included in the supply chain emissions parameter for primary production, *GHGPP*. Further detail on future mining, including a deposit-specific exploration and production model [93–95], is currently under development by the colleagues whose work is cited here, but not published yet.

6.3.11. Socioeconomic impacts

Socioeconomic impacts, like labour demand, costs, or value added in the different industrial processes modelled are not implemented yet.

6.4. Sensitivity analysis and scenarios

In prospective modelling, one needs to be clear about the purpose and the storyline behind each model run. We distinguish between sensitivity analysis (impact of single or combined parameter variation(s) on model outcomes) and scenarios (a set of parameter variations combined into a storyline). The scenarios are constrained by mass balance, resource availability, and stock inertia, but at this stage, we do not regard some scenarios as more likely to happen than others. All scenarios depicted represent possible futures from a biophysical point of view, and an assessment of their likelihood (how realistic they are) is beyond the scope of our work.

In the sensitivity analysis, we quantify the impact of variations in parameters on model outcome, one by one. This procedure helps us the understand model behaviour better and it allows us to identify the key model parameters for the material efficiency-climate change mitigation link. The parameter variations include both epistemic (we actually don’t know the true parameter value) and aleatory (the parameter value takes different values for different members of the sample) uncertainty [96]. For the parameter product lifetime, for example, the epistemic uncertainty is analysed by changing the mean value of the lifetime distribution, and the aleatory uncertainty is analysed by changing the standard deviation of the lifetime distribution.

In the scenario analysis, we run a socioeconomic scenario several times and add the different ME strategies, the so-called ME strategy cascade, cf. Table 4.2 and the explanations there.

7. Modelling environment, work flow, and interfaces

In this section the setup of the ODYM-RECC working environment is described and it is explained how the user can run custom scenarios.

7.1. Modelling environment: Software, database, and sharing

To set up the working environment for the ODYM-RECC model, four elements are necessary:

0. A local copy of the ODYM model framework for dynamic MFA
1. A local copy of the RECC model
2. A local copy of the RECC database (or path to the Dropbox repo)
3. A local result folder

Ad 1) The ODYM-RECC assessment is based on the software framework and database structure of ODYM. The model classes and functions of ODYM are hosted on the open source platform GitHub under the label of the already existing organisation Industrial Ecology: https://github.com/IndEcol/ODYM

It can be copied from there, either by direct download or by the `git clone` command.

Ad 2) The ODYM-RECC model and config files are shared via the (currently private) repo https://github.com/YaleCIE/RECC-ODYM

It can be copied from there, either by direct download or by the `git clone` command.

Ad 3) The data, in the format required for the model, are exchanged via Dropbox and – to the extent the licences allow – will be made openly available upon publication on Zenodo so that anyone with the sufficient computer skills will be able to replicate all scientific claims made. For example, the complete dataset for the ODYM-RECC v2.4 Global coverage model version is available under a permissive license via https://doi.org/10.5281/zenodo.4671643

The internal project data archive folder is \Dropbox\G7 RECC\Data\RECC_Database\CURRENT\

Ad 4) A local result folder needs to be created.

An example of the folder structure of ODYM-RECC is shown in Supplementary Figure 43.

In the RECC model main folder, also the working directory, a local git-ignored file “RECC_Paths.py” must be present that contains the following paths (see also Supplementary Figure 44):

- **odym_path**, points to local ODYM copy (Note that ODYM is not a package yet as it is still at an experimental stage)
- **data_path**, points to the local copy of the RECC project database.
- **results_path**, points to folder where model results are stored.

The main RECC_Model folder, which is also the working directory, contains the model configuration file RECC_Config_V2_4.xlsx and the different model scripts, each of which is configured in RECC_Config_V2_4.xlsx.

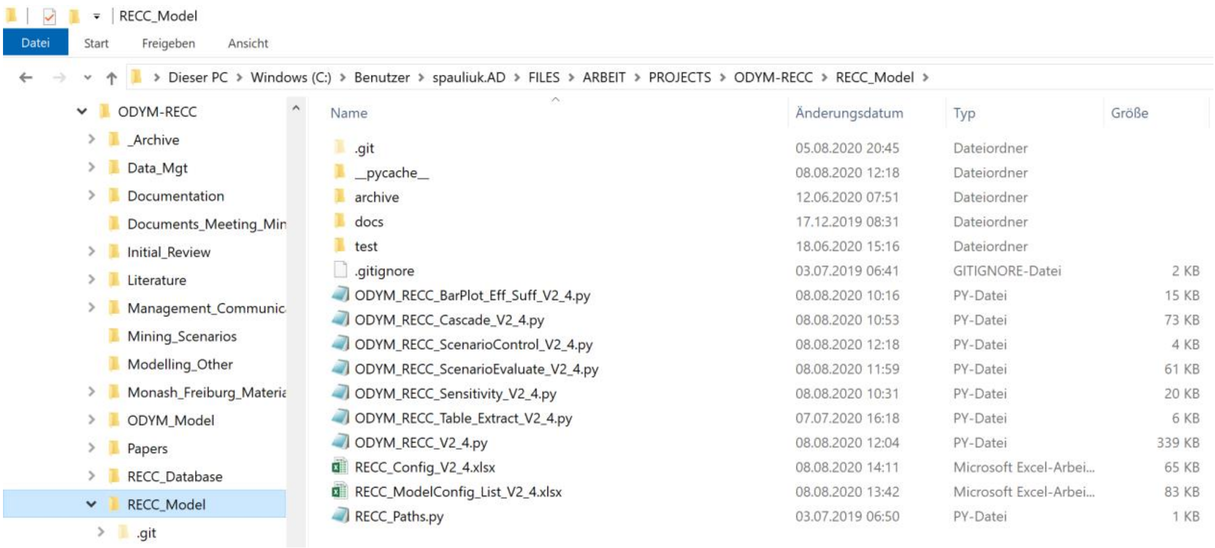

Supplementary Figure 43: Example of folder structure of ODYM-RECC. There need to be four folders: one with the ODYM model, one with the RECC model (which is also the working directory), one with the RECC database and one result folder.

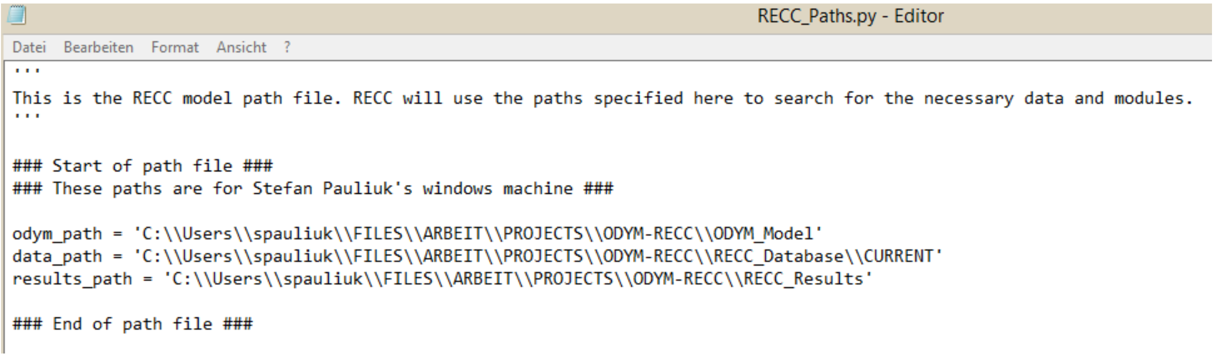


```
'''
This is the RECC model path file. RECC will use the paths specified here to search for the necessary data and modules.
'''

### Start of path file ###
### These paths are for Stefan Pauliuk's windows machine ###

odym_path = 'C:\\Users\\spauliuk\\FILES\\ARBEIT\\PROJECTS\\ODYM-RECC\\ODYM_Model'
data_path = 'C:\\Users\\spauliuk\\FILES\\ARBEIT\\PROJECTS\\ODYM-RECC\\RECC_Database\\CURRENT'
results_path = 'C:\\Users\\spauliuk\\FILES\\ARBEIT\\PROJECTS\\ODYM-RECC\\RECC_Results'

### End of path file ###
```

Supplementary Figure 44: The RECC path file setup. This example contains absolute MS-Windows paths but relative paths and LINUX are possible as well, as they will be combined with Python's operating system independent os.join() method.

When called, the RECC model script will determine its location in the directory tree of your computer and from there, look for the path file and the config file. From the path file the location of the data, the ODYM modules, and the result folder is determined.

7.2. Running the ODYM-RECC model

To run the RECC model, there are a number of options as described below. First, we list the available scripts and functions in the main model folder:

- **ODYM_RECC_V2_4.py:** Main model script, is organised as a function so that it can be called by the scenario control script for batch processing. If you want to add or change features of ODYM-RECC, create a git branch and convert the main model script to script mode (by commenting out the function definition and un-tabbing the main code).
- **ODYM_RECC_ScenarioControl_V2_4.py:** This script is used to run a larger number of model configurations defined in RECC_ModelConfig_List_V2_4.xlsx. For each model run, it will load

the model configuration parameters defined in RECC_ModelConfig_List_V2_4.xlsx, write them to the model config file RECC_Config_V2_4.xlsx, and run the main script ODYM_RECC_V2_4.py
- **ODYM_RECC_ScenarioEvaluate_V2_4.py:** This script is used to evaluate a larger number of model configurations defined in RECC_ModelConfig_List_V2_4.xlsx. After ODYM_RECC_ScenarioControl_V2_4.py has completed, the resulting list with the result folders of the individual model runs needs to be copied from the workspace and stored in RECC_ModelConfig_List_V2_4.xlsx, and ODYM_RECC_ScenarioEvaluate_V2_4.py can then be called to read the different results, call the single-sector evaluation scripts for each model region (ODYM_RECC_Cascade….py and ODAM_RECC _Sensitivity….py, cf. below), and write the result overview to an excel file.
- **ODYM_RECC_Cascade….py and ODYM_RECC_Sensitivity….py:** These scripts create a number of evaluation tables and plots for a given sector and given regions. They are called by ODYM_RECC_ScenarioEvaluate_V2_4.py

To run ODYM-RECC, follow these steps:

1. Pull or clone latest model version from GitHub (https://github.com/YaleCIE/RECC-ODYM) to your local working directory.
2. Create a path file with your local paths and add it to the model folder. An example can be found on \Dropbox\G7 RECC\Modeling\ODYM-RECC and in Supplementary Figure 44.
3. Copy the project database from Zenodo or Dropbox (\Dropbox\G7 RECC\Data\RECC_Database\CURRENT\) into the data folder specified in the path file.

For single model runs:

4. Open the model config file RECC_Config_V2_4.xlsx and define the model run parameters in the Config_Manual sheet, then specify the name 'Config_Manual' in cell D4 of the Config sheet. Comment out the function definition of the main script and run the main script ODYM_RECC_V2_4.py. The results will be stored in a single folder and be available in the workspace of the programming environment after the model run.

For multiple model runs:

5. Open the RECC_ModelConfig_List_V2_4.xlsx file and specify the model configurations you want to run by modifying existing model config lists or creating a new list (must use exact same structure as template provided)
6. Open the RECC config file and modify parameters that are not listed in RECC_ModelConfig_List_V2_4.xlsx.
7. Open the model script caller ODYM_RECC_ScenarioControl_V2_4.py, specify the sheet with the model configs from RECC_ModelConfig_List_V2_4.xlsx that you want to run, and press F5. The main model script will now be called with new configurations as many times as there are model configs in RECC_ModelConfig_List_V2_4.xlsx.
8. Copy the list `ResultsFolders` created by Python to the sheet "Evaluate_RECC_Cascade" in RECC_ModelConfig_List_V2_4.xlsx (or copy-paste from the generated Excel file ResultFolders.xls), save, and run the scenario evaluation control script ODYM_RECC_ScenarioEvaluate_V2_4.py, which will in turn call the scenario comparison and sensitivity scripts ODYM_RECC_Cascade_V2_4.py and ODYM_RECC_Sensitivity_V2_4.py as many times as needed.

7.3. RECC project work flows and database status

[For internal use and reference only!] To keep the project manageable and the workflow productive a set of rules is necessary.

RECC core rules:

- Do not modify or delete any files in the main database \Dropbox\G7 RECC\Data\RECC_Database\CURRENT\ without talking to the person responsible for each file.
- Do not push changes to the RECC model repo (https://github.com/YaleCIE/RECC-ODYM), create pull requests instead!

RECC workflows, internally:

Responsibilities: \Dropbox\G7 RECC\Data\admin\overview.xlsx

Data management scheme: \Dropbox\G7 RECC\Data\README.docx

The current status of the ODYM-RECC model database as well as the grouping of the different parameters is shown in Supplementary Table 26 below.

Supplementary Table 26: The ODYM-RECC parameters and their aspects/index structure, ODYM-RECC v2.4. For the ***italic*** parameters, own scenario modelling was carried out and applied.

Parameter_Name	Version	Index structure	Unit
2_P_RECC_Population_SSP_32R	V2.2	**MtrS**	Million
2_S_RECC_FinalProducts_2015_passvehicles	V1.3	**tcpr**	vehicles: million units. buildings: billion m2
2_S_RECC_FinalProducts_2015_resbuildings	V1.2	**tcBr**	vehicles: million units. buildings: billion m2
2_S_RECC_FinalProducts_2015_nonresbuildings	V1.0	**tcNr**	vehicles: million units. buildings: billion m2
1_F_Function_Future	V1.2	**GrtS**	inhabitant*m2*yr/yr and passenger-km/yr
1_F_RECC_FinalProducts_appliances	V1.0	**ocSRa**	items/yr
1_F_RECC_FinalProducts_industry	V1.0	**lSRIc**	GW/yr
2_S_RECC_FinalProducts_nonresbuildings_g	V1.0	**Nc**	m2/yr
2_S_RECC_FinalProducts_Future_resbuildings	V2.3	**StGr**	vehicles: cars per person, buildings: m2 per person
2_S_RECC_FinalProducts_Future_resbuildings_MIUPotential	V1.0	**GoS**	%
2_S_RECC_FinalProducts_Future_NonResBuildings	V1.0	**GrtS**	vehicles: cars per person, buildings: m2 per person
2_S_RECC_FinalProducts_Future_nonresbuildings_MIUPotential	V1.0	**GoS**	%
3_EI_Products_UsePhase_passvehicles	V1.2	**cpVnrS**	Vehicles: MJ/km. Buildings: MJ/m2/yr
3_EI_Products_UsePhase_resbuildings	V1.3	**cBVnrS**	Vehicles: MJ/km. Buildings: MJ/m2/yr
3_EI_Products_UsePhase_nonresbuildings	V1.0	**cNVnrS**	Vehicles: MJ/km. Buildings: MJ/m2/yr
3_IO_Vehicles_UsePhase	V2.3	**VrtS**	vehicles: km/yr
3_IO_Buildings_UsePhase_Historic	V1.3	**cBVrS**	buildings: share of area heated/cooled/DHW-supplied
3_IO_Buildings_UsePhase_Future_Heating	V1.0	**GrtS**	1
3_IO_Buildings_UsePhase_Future_Cooling	V1.0	**GrtS**	1
3_IO_NonResBuildings_UsePhase	V1.0	**cNVrS**	buildings: share of area heated/cooled/DHW-supplied
4_TC_ResidentialEnergyEfficiency_Default	V1.0	**VRrnt**	1
4_TC_ResidentialEnergyEfficiency_Scenario_Heating	V1.0	**VRrntS**	1
4_TC_ResidentialEnergyEfficiency_Scenario_Cooling	V1.0	**VRrntS**	1
6_MIP_VehicleOccupancyRate	V1.3	**GrtS**	1
3_LT_RECC_ProductLifetime_passvehicles	V3.1	**pr**	yr
3_LT_RECC_ProductLifetime_resbuildings	V4.2	**Brc**	yr
3_LT_RECC_ProductLifetime_NonResbuildings	V1.0	**Nrc**	yr

3_LT_RECC_ProductLifetime_appliances	V1.0	**a**	yr
3_LT_RECC_ProductLifetime_industry	V1.0	**I**	yr
3_LT_RECC_ProductLifetime_nonresbuildings_g	V1.0	**Noc**	yr
3_MC_RECC_Vehicles	V1.1	**cmpr**	kg/unit
3_MC_RECC_Buildings	V1.2	**cmBr**	kg/m2
3_MC_RECC_NonResBuildings	V1.0	**cmNr**	kg/m2
3_MC_RECC_Nonresbuildings_g	V1.0	**mN**	kg/m2
3_MC_RECC_industry	V1.1	**Im**	kt/GW
3_MC_RECC_appliances	V1.1	**oam**	g/item
3_MC_RECC_Buildings_Renovation_Relative	V1.0	**cmBr**	1
3_MC_RECC_Buildings_Renovation_Absolute	V1.0	**cmBr**	kg/m2
3_MC_Elements_Materials_ExistingStock	V2.2	**me**	1 (kg/kg)
3_MC_Elements_Materials_Primary	V2.2	**me**	1 (kg/kg)
3_PR_RECC_CO2Price_SSP_32R	V2.1	**RtrS**	US$2005/ton
3_SHA_RECC_REStrategyScaleUp	V3.3	**RotS**	1
3_SHA_BuildingRenovationScaleUp	V1.0	**RotS**	1
4_PE_GHGIntensityEnergySupply	V4.2	**XnSRrt**	kg of CO2-eq/MJ
4_PE_GHGIntensityEnergySupply_World	V4.1	**XnSRot**	kg of CO2-eq/MJ
4_PE_GHGIntensityElectricitySupply_Backstop	V1.2	**XnSRt**	kg of CO2-eq/MJ
4_PE_ProcessExtensions	V3.4	**PXotRS**	kg/kg
4_EI_ProcessEnergyIntensity	V2.2	**PntoR**	MJ/kg
4_EI_ManufacturingEnergyIntensity	V2.2	**Fnco**	MJ/kg
4_PY_EoL_RecoveryRate	V2.4	**gomwW**	%
4_PY_Manufacturing	V2.3	**mwgFto**	1
4_PY_MaterialProductionRemelting	V2.3	**wmeWto**	1
4_EI_WasteMgtEnergyIntensity	V1.1	**wnco**	MJ/kg
4_EI_RemeltingEnergyIntensity	V2.1	**mnco**	MJ/kg
6_PR_EoL_RR_Improvement	V2.3	**gomwW**	percentage points
6_PR_LifeTimeExtension_passvehicles	V2.1	**poS**	1
6_PR_LifeTimeExtension_resbuildings	V2.3	**BrS**	1
6_PR_LifeTimeExtension_nonresbuildings	V1.1	**Nr**	1
6_PR_LifeTimeExtension_nonresbuildings_g	V1.0	**No**	1
6_PR_LifeTimeExtension_appliances	V1.0	**aoS**	1
6_PR_LifeTimeExtension_industry	V1.0	**IlS**	1
6_PR_FabricationYieldImprovement	V2.1	**mgoS**	1
6_PR_FabricationScrapDiversion	V1.2	**mwoS**	1
6_PR_ReUse_Bld	V3.3	**mBo**	1
**6_PR_ReUse_Veh**	V1.2	**mprtS**	1
6_PR_ReUse_nonresBld	V1.2	**mNo**	1
6_PR_DirectEmissions	V1.2	**Xn**	kg of CO2-eq/MJ
**6_PR_CarSharingShare**	V1.2	**GotS**	1
**6_PR_RideSharingShare**	V2.0	**GrtS**	1
3_SHA_TypeSplit_Vehicles	V3.0	**GrRpt**	%
**3_SHA_TypeSplit_Buildings**	V1.3	**BrtS**	%
**3_SHA_TypeSplit_NonResBuildings**	V1.0	**NrtS**	%
3_SHA_EnergyCarrierSplit_Vehicles	V1.1	**cpoVnS**	%
3_SHA_EnergyCarrierSplit_Buildings	V2.3	**VRrnt**	%
3_SHA_EnergyCarrierSplit_NonResBuildings	V1.0	**VRrnt**	%
3_MC_VehicleArchetypes	V2.0	**Am**	kg/unit, kg/m2
3_MC_BuildingArchetypes	V1.2	**Arm**	kg/unit, kg/m2
3_MC_NonResBuildingArchetypes	V1.0	**Arm**	kg/unit, kg/m2
3_EI_VehicleArchetypes	V4.0	**An**	MJ/km, MJ/m2/yr
3_EI_BuildingArchetypes	V1.2	**ArVn**	MJ/km, MJ/m2/yr
3_EI_NonResBuildingArchetypes	V1.0	**ArVn**	MJ/km, MJ/m2/yr
3_SHA_DownSizing_Vehicles	V2.3	**srtS**	%
3_SHA_LightWeighting_Vehicles	V1.3	**prtS**	%
3_SHA_DownSizing_Buildings	V1.3	**urtS**	%
3_SHA_LightWeighting_Buildings	V2.2	**GrtS**	%
3_SHA_DownSizing_NonResBuildings	V1.0	**urtS**	%

3_SHA_LightWeighting_NonResBuildings	V1.0	**GrtS**	%
6_PR_Calibration	V2.4	**Cr**	ratio
6_MIP_CarSharing_Stock	V1.0	**Sr**	1
6_MIP_RideSharing_Occupancy	V1.1	**Sr**	1 (For RECC Germany: V1.0)
6_MIP_GWP_Bio	V1.0	**c**	1
4_PE_ElectricityFromWoodCombustion	V1.0	**wWn**	GJ/ton
3_LT_ForestRotationPeriod_FuelWood	V1.0	**n**	yr
3_LT_ForestRotationPeriod_Timber	V1.0	**m**	yr
3_MC_CO2FromWoodCombustion	V1.0	**Xm**	1
3_EI_HeatingValueWoodPerCarbon	V1.0	**en**	MJ/kg
3_MC_CementContentConcrete	V1.0	**mm**	1
3_SHA_CementContentReduction	V1.0	**m**	1
3_SHA_MaxRenovationPotential_ResBuildings	V1.1	**rcB**	%
3_SHA_MaxRenovationPotential_NonResBuildings	V1.0	**rcN**	%
3_SHA_EnergySavingsPot_Renovation_ResBuildings	V1.1	**rSB**	%
3_SHA_EnergySavingsPot_Renovation_NonResBuildings	V1.0	**rSN**	%
6_MIP_CharacterisationFactors	V1.0	**xX**	mics. Units
8_FLAG_VehicleDownsizingDirection	V1.0	**rS**	Bool

7.4. Interfaces from and to the ODYM-RECC model

From the scenario database (I) to ODYM-RECC (IV):

Most ODYM-RECC parameters are scenario-dependent (cf. Supplementary Table 26). Some parameters, like population or GDP (not used by ODYM-RECC v2.4) are obtained directly from the SSP database at http://www.iiasa.ac.at/web/home/research/researchPrograms/Energy/SSP_Scenario_Database.html while other need to be compiled by the RECC project team based on the SSP storylines. The parameters for which own scenario work needs to be done are highlighted in italics in Supplementary Table 26.

All other parameters currently labelled as scenario depended are taken from other databases (4_PE_GHGIntensityEnergySupply from SSP/MESSAGE scenario database), set constant or modelled with an LCA scenario tool (4_EI_ProcessEnergyIntensity, 4_EI_WasteMgtEnergyIntensity, etc.), or are determined by the scale-up of the RE strategies (6_PR_MoreIntenseUse, 6_PR_FabricationYieldImprovement), etc. For the latter group, there is no SSP dependency but scenario-independent potentials, and the only scenario-dependent RE parameter will be 3_SHA_RECC_REStrategyScaleUp.

GHG emissions per MJ produced for the different energy carriers and 11 world regions are available from the MESSAGE results database [28]. In addition, data for hydrogen production were used from the IEA WEO model [30]. To reflect that the CURRENT electricity mix in the individual countries differs from the regional total modelled by MESSAGE, we introduced a linear interpolation from today's g CO_2/kWh for each country to the aggregate regional number from MESSAGE in 2040. This way, we can reflect current and mid-term regional difference but assume ultimate convergence to the regional average as electricity supply becomes more international to facilitate the integration of renewable sources of electricity.

From the archetype model (II) to ODYM-RECC (IV):

Supplementary Table 27 lists the interface between the archetype model (II) and the ODYM-RECC scenario model (IV). ODYM-RECC does not deal with archetypes, just the average of each building type for each age-cohort, which, for future age-cohorts, can be represented as mix of different archetypes/prototypes, as explained in the section 'Product material composition and energy use in use phase' above. That means that the archetypes defined by the product modelling teams are mixed together (e.g., x % standard and 100%-x% alternative, where x is time- and scenario-dependent) when entering the calculations.

Supplementary Table 27: ODYM-RECC parameters for which scenarios are derived from mixing different archetypes within the RECC project.

Parameter name	Description	Resolution of parameter
3_MC_RECC_Buildings	Future material composition of residential buildings	Age-cohort x material x building type x region
3_MC_RECC_NonResBuildings	Future material composition of non-residential buildings	Age-cohort x material x building type x region
3_MC_RECC_Vehicles	Future material composition of vehicles	Age-cohort x material x vehicle type x region
3_EI_Products_UsePhase_passvehicles	Energy intensity, MJ/km, of vehicles in use	Age-cohort x product type x service x energy carrier x region x scenario
3_EI_Products_UsePhase_resbuildings	Energy intensity, MJ/m2/yr, for buildings in use	Age-cohort x product type x service x energy carrier x region x scenario
3_EI_Products_UsePhase_nonresbuildings	Energy intensity, MJ/m2/yr, for buildings in use	Age-cohort x product type x service x energy carrier x region x scenario

From the LCIA (III) to ODYM-RECC (IV):

The ODYM-RECC model produces scenarios for energy demand in all industrial processes and the use phase in Supplementary Figure 41. It also contains parameters from the LCIA part III for process and direct GHG emissions (aspect X) so that currently, the GHG emissions of the entire system are computed within ODYM-RECC ('GHG emissions' part in model IV). Other extensions can be added to the X aspect as well.

For each model run, the material and energy flows are exported in table format so that other assessments can be made.

8. Outlook, future model expansion and development

The ODYM-RECC model will be expanded both in terms of regional and sectoral scope and in terms of modelling capability.

8.1. Expanding the scope of the ODYM-RECC model

P1) The following extensions are currently planned:

+ Consider infrastructure, other transport, and a detailed representation of non-residential buildings, as well as all climate-relevant bulk materials, using the results of existing assessments and previous work [57–59].

P2) Model a sufficiently large fraction of the total use of a metal in detailed ODYM-RECC sectors and then scale up to total demand using empirical relationships. **This is first planned for copper and possible with ODYM-RECC 2.4.**

P3) The second priority is to make our scenarios consistent with other prominent macro-scale assessments, such as the SSP scenario runs of IAMs or the work done for the Global Resources Outlook [16].

8.2. Expanding the capabilities of the ODYM-RECC model

Further incorporating basic economic accounts and effects into ODYM-RECC would be a major breakthrough, as the model and scenarios would gain substantial 'socioeconomic credibility'. The theory of physical production functions seems underdeveloped. Winning et al. [12] list the cost structures of material producing sectors (a column of the A-matrix, aggregated to 10-15 categories). We could go further in the aggregation of inputs and include KLEMS-accounts for each process covered by the model. The KLEMS accounts can be scaled for different scenarios to estimate future costs, economic impact, and labour input of the material cycles. In a second step, the KLEMS accounts can enter an optimisation model. A future combination with a CGE model should be planned for as a further model integration step.

There is ongoing work on decomposing LCI data and incorporating them into IAMs [97,98]. This approach could be a blueprint for more systematically linking life cycle thinking with material cycle modelling and subsequent indicator development and needs further investigation. Also, the issue of variability of emissions over time and the question of dynamic characterisation factors needs more investigation [99,100].

Finally, we plan to link the ODYM-RECC primary production scenario to a mining supply module to have consistent metal demand-mining supply models at the global scale, with copper as the first case study [93–95].

8.3. Interface to other modelling frameworks

Integrated assessment models

ODYM is developed as a self-contained standalone prospective modelling framework. Like all prospective models that involve social systems, ODYM needs a set of exogenous parameters to run, and these parameters need to follow a certain scenario storyline. ODYM-RECC scenarios share their storylines with integrated assessment models (IAMs) by using the Shared Socioeconomic Pathways as exogenous scenarios. Different degrees of coupling tightness between ODYM and IAMs are applied:

In the most basic case, where sufficient detail from the available IAM scenarios is absent, the only shared parameters are the main SSP scenario drivers population, GDP per capita, and urbanisation. Within the RECC team, we then used the scenario target tables to add detail to the storylines, cf. the scenario modelling docu. Regression models similar to the ones use by, e.g., GCAM and IMAGE, to build scenarios for the service delivered by buildings, transport, and industrial output, were only used in rare cases. For example, regression-based ARIMAX model forecasts are used for the USA and Japan for floor space per capita in the SSP2 scenario, as here, sufficiently long time series were present and the SSP2 storyline can be seen as a continuation of historic trends.

For this approach, exact comparability to other scenario model results will not be achieved, only the broad model drivers will be the same. This coupling mode is the initial running mode for the ODYM-RECC as the time frame of the first assessment is too narrow to successfully establish a close interaction with IAMs.

In a tighter coupling option, ODYM will service demand directly from IAM results. In the simplest case, this would just mean that the regression model equations are replaced by formatted IAM model output. Such approach has already been lined out for the five materials Cu, Co, Li, Nd, and and Ta, where IMAGE model output for the use phase was converted to material in-use stocks, inflows, and outflows of the use phase [60,61]. Also, the implementation of the material cycle consequences of electricity generation installation from MESSAGE is already under way. This approach only covers the sectors that are well represented in the IAM, while other sectors are not considered. That means that for the full material cycle picture a large-scale scenario target table, regression, or upscaling model will be needed in parallel to the sector-specific assessment done in coupling to the IAM.

8.4. ODYM-RECC FAQs

Q: What kind of computer is needed to run ODYM-RECC v2.4.? **A:** A normal PC or laptop is sufficient, as long as it has a large enough working memory. For a single-region run, at least 12 GB RAM are required, and for running multiple regions in one go, like the G7 (seven regions) or EU28 (nine regions in the project's classification), 32 GB are required. For a single region, 35 product groups selected, and a not too fancy Windows laptop with 32 GB RAM, the main model script takes 20-40 seconds to run through.

Q: Do I have to use the data formatting templates when running ODYM models? **A:** The ODYM functions can be used without the database structure and the parameter files. Data can be read using custom-made routines. For reproducible group work a more professional setup is necessary, however, and the data formatting templates were developed to simplify data parsing (all data files are parsed

by a single routine) and to prepare for the storage of data as data packages. For the ODYM-RECC model, all data come in the ODYM templates v0.2.

Q: Can I use the RECC project database without using the ODYM-RECC model framework? **A:** Yes, no problem. The model framework and the database are two interlinked but separate things.

Q: Can I use the ODYM-RECC model without using the RECC project database? **A:** No. ODYM-RECC needs a certain set of data, and these data are stored in the RECC project database.

Q: Why is ODYM-RECC open access? **A:** Because we believe that open science creates a positive pressure to do better work, because external expertise and feedback can be obtained and incorporated easy, and because we believe that future industrial ecology scenario modelling needs to be more collaborative to progress more quickly and make increasingly relevant contributions to tackling pressing sustainability challenges.

Q: How can I contribute to the development of ODYM-RECC? **A:** Both the database and the model need extension. Please check the model repo wiki on https://github.com/YaleCIE/RECC-ODYM for pending tasks and open issues!

Supplementary References

1. Haberl, H., Wiedenhofer, D., Erb, K. H., Görg, C. & Krausmann, F. The material stock-flow-service nexus: A new approach for tackling the decoupling conundrum. *Sustain.* **9**, 1049 (2017).
2. Kalt, G., Wiedenhofer, D., Görg, C. & Haberl, H. Energy Research & Social Science Conceptualizing energy services : A review of energy and well-being along the Energy Service Cascade. *Energy Res. Soc. Sci.* **53**, 47–58 (2019).
3. Edelenbosch, O. Y., van Vuuren, D. P., Blok, K., Calvin, K. & Fujimori, S. Mitigating energy demand sector emissions: The integrated modelling perspective. *Appl. Energy* **261**, 114347 (2020).
4. Fishman, T. *et al.* A comprehensive set of global scenarios of housing, mobility, and material efficiency for material cycles and energy systems modelling. *J. Ind. Ecol.* **25**, 305–320 (2021).
5. Wolfram, P., Tu, Q., Heeren, N., Pauliuk, S. & Hertwich, E. G. Material efficiency for immediate climate change mitigation of passenger vehicles. *J. Ind. Ecol.* **25**, 494–510 (2021).
6. Pauliuk, S. & Heeren, N. ODYM - An Open Software Framework for Studying Dynamic Material Systems - Principles, Implementation, and Data Structures. *J. Ind. Ecol.* **24**, 446–458 (2020).
7. Pauliuk, S. *et al.* Linking Service Provision to Material Cycles – A New Framework for Studying the Resource Efficiency-Climate Change Nexus (RECC). *J. Ind. Ecol.* **25**, 260–273 (2021).
8. Hertwich, E. G., Lifset, R. J., Pauliuk, S. & Heeren, N. *Resource Efficiency and Climate Change: Material Efficiency Strategies for a Low-Carbon Future.* (2020). doi:10.5281/ZENODO.3542680
9. Grubler, A. *et al.* A low energy demand scenario for meeting the 1.5 °C target and sustainable development goals without negative emission technologies. *Nat. Energy* **3**, 515–527 (2018).
10. IEA. *World Energy Outlook 2015*. (2015).
11. McCarthy, A., Dellink, R. & Bibas, R. *The Macroeconomics of the Circular Economy Transition: A Critical Review of Modelling Approaches OECD Environment Working Papers, No. 130*. (2018). doi:http://dx.doi.org/10.1787/af983f9a-en
12. Winning, M., Calzadilla, A., Bleischwitz, R. & Nechifor, V. Towards a circular economy : insights based on the development of the global ENGAGE-materials model and evidence for the iron and steel industry. *Int Econ Econ Policy* **14**, 383–407 (2017).
13. Balistreri, E. J., Böhringer, C. & Rutherford, T. F. Carbon policy and the structure of global trade. *World Econ.* **41**, 194–221 (2018).
14. Böhringer, C. & Rutherford, T. F. Combining bottom-up and top-down. *Energy Econ.* **30**, 574–596 (2008).
15. OECD. *Global Material Resources Outlook to 2060 – Economic drivers and environmental consequences*. (2019).
16. UNEP-IRP. *Global Resources Outlook 2019: Natural Resources for the Future We Want.* (2019).

17. Schandl, H. *et al.* Decoupling global environmental pressure and economic growth: scenarios for energy use, materials use and carbon emissions. *J. Clean. Prod.* **132**, 45–56 (2016).
18. Hatfield-Dodds, S., Obersteiner, M. & Schandl, H. *UNEP-IRP (International Resource Panel): Terms of Reference. Developing and demonstrating scenario modelling of integrated resource management*. (International Resource Panel, 2017).
19. Lennox, J. A., Turner, G., Hoffman, R. & Mcinnis, B. Modeling Basic Industries in the Australian Stocks and Flows Framework. *J. Ind. Ecol.* **8**, 101–120 (2005).
20. Schumacher, K. & Sands, R. D. *Where Are the Industrial Technologies in Energy-Economy Models ? – An Innovative CGE Approach for Steel Production in Germany. DIW Discussion paper 605*. (2006).
21. Cooper, S. J. G. *et al.* Thermodynamic insights and assessment of the 'circular economy'. *J. Clean. Prod.* **162**, 1356–1367 (2017).
22. Haberl, H. *et al.* A systematic review of the evidence on decoupling of GDP, resource use and GHG emissions, part II: synthesizing the insights. *Environ. Res. Lett.* **15**, 65003 (2020).
23. Müller, D. B. Stock dynamics for forecasting material flows - Case study for housing in The Netherlands. *Ecol. Econ.* **59**, 142–156 (2006).
24. Brunner, P. H. & Rechberger, H. *Practical Handbook of Material Flow Analysis. 2nd edition*. (CRC Press, 2016).
25. Milford, R. L., Pauliuk, S., Allwood, J. M. & Müller, D. B. The Roles of Energy and Material Efficiency in Meeting Steel Industry CO2 Targets. *Environ. Sci. Technol.* **47**, 3455–3462 (2013).
26. Modaresi, R., Pauliuk, S., Løvik, A. N. & Müller, D. B. Global carbon benefits of material substitution in passenger cars until 2050 and the impact on the steel and aluminum industries. *Environ. Sci. Technol.* **48**, 10776–10784 (2014).
27. Börjeson, L., Höjer, M., Dreborg, K.-H., Ekvall, T. & Finnveden, G. Scenario types and techniques: Towards a user's guide. *Futures* **38**, 723–739 (2006).
28. Riahi, K. *et al.* The shared socioeconomic pathways and their energy, land use, and greenhouse gas emissions implications: An overview. *Glob. Environ. Chang.* **42**, 153–168 (2017).
29. O'Neill, B. C. *et al.* A new scenario framework for climate change research: the concept of shared socioeconomic pathways. *Clim. Change* **122**, 387–400 (2014).
30. OECD/IEA. *Energy Technology Perspectives 2010*. (IEA, 2010).
31. IEA. *Energy Technology Perspectives*. (International Energy Agency, 2015).
32. OECD/IEA. *Energy Technology Perspectives*. (2017).
33. Hawkins, T. R., Singh, B., Majeau-Bettez, G. & Strømman, A. H. Comparative Environmental Life Cycle Assessment of Conventional and Electric Vehicles. *J. Ind. Ecol.* **17**, 53–64 (2013).
34. Reyna, J. L. & Chester, M. V. The Growth of Urban Building Stock. *J. Ind. Ecol.* **19**, 524–537 (2015).
35. Marcellus-Zamora, K. A., Gallagher, P. M., Spatari, S. & Tanikawa, H. Estimating Materials Stocked by Land-Use Type in Historic Urban Buildings Using Spatio-Temporal Analytical Tools. *J. Ind. Ecol.* **20**, 1025–1037 (2016).
36. Pauliuk, S., Milford, R. L., Müller, D. B. & Allwood, J. M. The Steel Scrap Age. *Environ. Sci. Technol.* **47**, 3448–3454 (2013).
37. Liu, G., Bangs, C. E. & Müller, D. B. Stock dynamics and emission pathways of the global aluminium cycle. *Nat. Clim. Chang.* **2**, 338–342 (2012).
38. Wernet, G. *et al.* The ecoinvent database version 3 (part I): overview and methodology. *Int. J. Life Cycle Assess.* **21**, 1218–1230 (2016).
39. OECD/IEA. *Energy Technology Perspectives : Scenarios and Strategies to 2050*. (2010).
40. Allwood, J. M. *et al. Sustainable Materials: With Both Eyes Open*. (UIT Cambridge, UK, 2012).
41. Reck, B. K. & Graedel, T. E. Challenges in metal recycling. *Science* **337**, 690–5 (2012).
42. Liu, J. *et al.* Systems integration for global sustainability. *Science (80-.).* **347**, 12588321–12588329 (2015).
43. Pauliuk, S. & Hertwich, E. G. Prospective models of society's future metabolism - What industrial ecology has to contribute. in *Taking Stock of Industrial Ecology* (eds. Clift, R. & Duckmann, A.) 21–43 (Springer, Netherlands., 2016).
44. Pauliuk, S., Arvesen, A., Stadler, K. & Hertwich, E. G. Industrial ecology in integrated assessment models. *Nat. Clim. Chang.* **7**, 13–20 (2017).
45. Hatfield-Dodds, S. *et al.* Assessing global resource use and greenhouse emissions to 2050, with ambitious resource efficiency and climate mitigation policies. *J. Clean. Prod.* **144**, 403–414 (2017).
46. Hatfield-Dodds, S. *et al.* Australia is 'free to choose' economic growth and falling environmental pressures. *Nature* **527**, 49–53 (2015).
47. Kim, H. C. & Wallington, T. J. Life-cycle energy and greenhouse gas emission benefits of lightweighting in automobiles: review and harmonization. *Environ. Sci. Technol.* **47**, 6089–97 (2013).
48. Shanks, W. *et al.* How much cement can we do without? Lessons from cement material flows in the UK. *Resour. Conserv. Recycl.* **141**, 441–454 (2019).
49. Enkvist, P.-A. & Klevnäs, P. *The Circular Economy a Powerful Force for Climate Mitigation Transformative - innovation for prosperous and low-carbon industry*. (2018).
50. Hertwich, E. G. *et al.* Material efficiency strategies to reducing greenhouse gas emissions associated with buildings, vehicles, and electronics—a review. *Environ. Res. Lett.* **14**, 043004 (2019).
51. Zink, T. & Geyer, R. Circular Economy Rebound. *J. Ind. Ecol.* **21**, 593–602 (2017).
52. Hertwich, E. G. Consumption and the Rebound Effect: An Industrial Ecology Perspective. *J. Ind. Ecol.* **9**, 85–98 (2005).
53. Allwood, J. M., Ashby, M. F., Gutowski, T. G. & Worrell, E. Material efficiency: A white paper. *Resour. Conserv.*

Recycl. **55**, 362–381 (2011).
54. Hatayama, H., Daigo, I., Matsuno, Y. & Adachi, Y. Outlook of the world steel cycle based on the stock and flow dynamics. *Environ. Sci. Technol.* **44**, 6457–63 (2010).
55. Elshkaki, A. & Graedel, T. E. Dynamic analysis of the global metals flows and stocks in electricity generation technologies. *J. Clean. Prod.* **59**, 260–273 (2013).
56. Elshkaki, A., Graedel, T. E., Ciacci, L. & Reck, B. K. Copper demand, supply, and associated energy use to 2050. *Glob. Environ. Chang.* **39**, 305–315 (2016).
57. van der Voet, E., van Oers, L., Verboon, M. & Kuipers, K. Environmental Implications of Future Demand Scenarios for Metals Methodology and Application to the Case of Seven Major Metals. *J. Ind. Ecol.* **23**, 141–155 (2019).
58. Schipper, B. W. *et al.* Resources , Conservation & Recycling Estimating global copper demand until 2100 with regression and stock dynamics. *Resour. Conserv. Recycl.* **132**, 28–36 (2018).
59. Elshkaki, A., Graedel, T. E., Ciacci, L. & Reck, B. K. Resource Demand Scenarios for the Major Metals. *Environ. Sci. Technol.* **52**, 2491–2497 (2018).
60. Deetman, S., Pauliuk, S., Van Vuuren, D. P., van der Voet, E. & Tukker, A. Scenarios for Demand Growth of Metals in Electricity Generation Technologies, Cars, and Electronic Appliances. *Environ. Sci. Technol.* **52**, 4950–4959 (2018).
61. Deetman, S. *et al.* Modelling global material stocks and flows for residential and service sector buildings towards 2050. *J. Clean. Prod.* **245**, 118658 (2020).
62. Watari, T. *et al.* Resources , Conservation & Recycling Total material requirement for the global energy transition to 2050 : A focus on transport and electricity. *Resour. Conserv. Recycl.* **148**, 91–103 (2019).
63. Gielen, D. J., Gerlagh, T. & Bos, A. J. M. *MATTER 1.0 - A MARKAL Energy and Materials System Model Characterisation*. (1998).
64. Gielen, D. J. Materialising dematerialisation. Integrated energy and materials systems engineering for greenhouse gas emission mitigation. (TU Delft, 1999).
65. Groenendaal, B. J. & Gielen, D. J. *The future of the petrochemical industry: A MARKAL-MATTER analysis*. (ECN, Energy Research Centre of the Netherlands, 1999).
66. Kram, T. *et al. The MATTER project: Integrated energy and materials systems engineering for GHG emission mitigation*. (ECN, Energy Research Centre of the Netherlands, 2001).
67. Stehfest, E. *et al. Integrated Assessment of Global Environmental Change with IMAGE 3.0 - Model description and policy applications*. (2014).
68. van Ruijven, B. J. *et al.* Long-term model-based projections of energy use and CO2 emissions from the global steel and cement industries. *Resour. Conserv. Recycl.* **112**, 15–36 (2016).
69. Cao, Z., Liu, G., Zhong, S., Dai, H. & Pauliuk, S. Integrating Dynamic Material Flow Analysis and Computable General Equilibrium Models for Both Mass and Monetary Balances in Prospective Modeling : A Case for the Chinese Building Sector. *Environ. Sci. Technol.* **53**, 224–233 (2018).
70. Ayres, R. U., Ayres, L. W. & Masini, A. An Application of Exergy Accounting to Five Basic Metal Industries. Eco-Efficiency in Industry and Science, vol 19. in *Sustainable Metals Management.* (eds. von Gleich, A., Ayres, R. U. & Gößling-Reisemann, S.) (Springer, 2006).
71. Wu, J., Wang, R., Pu, G. & Qi, H. Integrated assessment of exergy, energy and carbon dioxide emissions in an iron and steel industrial network. *Appl. Energy* **183**, 430–444 (2016).
72. Gutowski, T. G. *et al.* Thermodynamic Analysis of Resources Used in Manufacturing Processes. *Environ. Sci. Technol.* **43**, 1584–1590 (2009).
73. Worrell, E., Allwood, J. M. & Gutowski, T. The Role of Material Efficiency in Environmental Stewardship. *Annu. Rev. Environ. Resour.* **41**, 575–598 (2016).
74. Pauliuk, S., Heeren, N., Hasan, M. M. & Müller, D. B. A general data model for socioeconomic metabolism and its implementation in an industrial ecology data commons prototype. *J. Ind. Ecol.* **23**, 1016–1027 (2019).
75. van Vuuren, D. P. *et al.* The Shared Socio-economic Pathways: Trajectories for human development and global environmental change. *Glob. Environ. Chang.* **42**, 148–152 (2017).
76. Heeren, N. *et al. Annex C of the UN-IRP report 'Resource Efficiency and Climate Change - Material Efficiency Strategies for a Low-Carbon Future' Supplementary material for the in-depth industrial ecology assessment*. (2020).
77. Wolfram, P., Tu, Q., Hertwich, E. G. & Pauliuk, S. *Documentation of the transport-sector model v1.0 within the RECC model framework.* (2020). doi:10.5281/zenodo.3631938
78. KC, S. & Lutz, W. The human core of the shared socioeconomic pathways: Population scenarios by age, sex and level of education for all countries to 2100. *Glob. Environ. Chang.* **42**, 181–192 (2014).
79. Pauliuk, S., Kondo, Y., Nakamura, S. & Nakajima, K. Regional distribution and losses of end-of-life steel throughout multiple product life cycles—Insights from the global multiregional MaTrace model. *Resour. Conserv. Recycl.* **116**, 84–93 (2017).
80. Bürger, V., Hesse, T., Köhler, B., Palzer, A. & Engelmann, P. German Energiewende — different visions for a (nearly) climate neutral building sector in 2050. *Energy Effic.* **12**, 73–87 (2019).
81. Glöser, S., Soulier, M. & Tercero Espinoza, L. a. Dynamic analysis of global copper flows. Global stocks, postconsumer material flows, recycling indicators, and uncertainty evaluation. *Environ. Sci. Technol.* **47**, 6564–72 (2013).
82. Nakamura, S., Kondo, Y., Nakajima, K., Ohno, H. & Pauliuk, S. Quantifying Recycling and Losses of Cr and Ni in Steel Throughout Multiple Life Cycles Using MaTrace-Alloy. *Environ. Sci. Technol.* **51**, 9469–9476 (2017).
83. Hertwich, E. G. *et al.* Integrated life-cycle assessment of electricity-supply scenarios confirms global environmental benefit of low-carbon technologies. *Proc. Natl. Acad. Sci.* **112**, 6277–6282 (2015).
84. Guest, G., Cherubini, F. & Strømman, A. H. Global Warming Potential of Carbon Dioxide Emissions from Biomass

Stored in the Anthroposphere and Used for Bioenergy at End of Life. *J. Ind. Ecol.* **17**, 20–30 (2013).
85. Fischer-Kowalski, M. & Weisz, H. Society as Hybrid between Material and Symbolic Realms: Toward a Theoretical Framework of Society-Nature Interaction. *Adv. Hum. Ecol.* **8**, 215–251 (1999).
86. Ayres, R. U. & Kneese, A. Production, Consumption, and Externalities. *Am. Econ. Rev.* **59**, 282–297 (1969).
87. van der Voet, E., Kleijn, R., Huele, R., Ishikawa, M. & Verkuijlen, E. Predicting future emissions based on characteristics of stocks. *Ecol. Econ.* **41**, 223–234 (2002).
88. Kondo, Y. & Nakamura, S. Waste input–output linear programming model with its application to eco-efficiency analysis. *Econ. Syst. Res.* **17**, 393–408 (2005).
89. Løvik, A. N., Modaresi, R. & Müller, D. B. Long-Term Strategies for Increased Recycling of Automotive Aluminum and Its Alloying Elements. *Environ. Sci. Technol.* **48**, 4257–4265 (2014).
90. Gaustad, G., Olivetti, E. & Kirchain, R. Toward Sustainable Material Usage : Evaluating the Importance of Market Motivated Agency in Modeling Material Flows. *Environ. Sci. Technol.* **45**, 4110–4117 (2011).
91. Northey, S. A., Mohr, S., Mudd, G. M., Weng, Z. & Giurco, D. Modelling future copper ore grade decline based on a detailed assessment of copper resources and mining. *Resour. Conserv. Recycl.* **83**, 190–201 (2014).
92. Northey, S. A. *et al.* The exposure of global base metal resources to water criticality, scarcity and climate change. *Glob. Environ. Chang.* **44**, 109–124 (2017).
93. Northey, S. A., Mohr, S., Mudd, G. M., Weng, Z. & Giurco, D. Modelling future copper ore grade decline based on a detailed assessment of copper resources and mining. *Resour. Conserv. Recycl.* **83**, 190–201 (2014).
94. Norgate, T. E. & Haque, N. Energy and greenhouse gas impacts of mining and mineral processing operations. *J. Clean. Prod.* **18**, 266–274 (2010).
95. Mudd, G. M., Jowitt, S. & Weng, Z. A detailed assessment of global Cu reserveand resource trends and worldwide Cu endowments. *Econ. Geol.* **108**, 1163–1183 (2013).
96. Laner, D., Rechberger, H. & Astrup, T. F. Systematic Evaluation of Uncertainty in Material Flow Analysis. *J. Ind. Ecol.* **18**, 859–870 (2014).
97. Arvesen, A., Luderer, G., Pehl, M., Leon, B. & Hertwich, E. G. Environmental Modelling & Software Deriving life cycle assessment coef fi cients for application in integrated assessment modelling. *Environ. Model. Softw.* **99**, 111–125 (2018).
98. Pehl, M. *et al.* Understanding future emissions from low-carbon power systems by integration of life-cycle assessment and integrated energy modelling. *Nat. Energy* **2**, 939–945 (2017).
99. Levasseur, A., Lesage, P., Margni, M., Deschenes, L. & Samson, R. Considering Time in LCA : Dynamic LCA and Its Application to Global Warming Impact Assessments. *Environ. Sci. Technol.* **44**, 3169–3174 (2010).
100. Levasseur, A., Lesage, P., Margni, M. & Samson, R. Biogenic Carbon and Temporary Storage Addressed with Dynamic Life Cycle Assessment. *J. Ind. Ecol.* **17**, 117–128 (2013).
